# Synthesis and Antiproliferative Activity against Melanoma Cells of New Heterocyclic Hybrids Based on Pyridine and Pyrimidine Scaffolds

**DOI:** 10.2174/0109298673370617250330151330

**Published:** 2025-06-10

**Authors:** Magdalena Perużyńska, Radosław Birger, Tomasz J. Idzik, Zofia M. Myk, Magdalena M. Lubowicz, Łukasz Struk, Jacek G. Sośnicki, Patrycja Kłos, Dariusz Chlubek, Marek Droździk

**Affiliations:** 1 Department of Experimental and Clinical Pharmacology, Pomeranian Medical University in Szczecin, Powstanców Wielkopolskich 72, 70-111, Szczecin, Poland;; 2 Department of Organic and Physical Chemistry, Faculty of Chemical Technology and Engineering, West Pomeranian University of Technology in Szczecin, Piastów 42, 71-065, Szczecin, Poland;; 3 Center for Advanced Materials and Manufacturing Process Engineering (CAMMPE), Piastów 42, 71-065, Szczecin, Poland;; 4 Department of Biochemistry and Medical Chemistry, Pomeranian Medical University in Szczecin, Powstanców Wielkopolskich, 72, 70-111, Szczecin, Poland

**Keywords:** Pyridines, pyrimidines, heterocycles, anticancer agents, antiproliferative agents, ADMET, melanoma, drug development

## Abstract

**Background:**

Over 85% of biologically active compounds are heterocycles or contain heterocyclic groups, underscoring their vital importance in contemporary drug development. Among them, nitrogen-containing derivatives, such as pyridines and pyrimidines, are considered privileged structures in approved drugs or are extensively studied due to their promising therapeutic effects.

**Objective:**

In the current work, we would like to verify the hypothesis that incorporating heterocyclic pharmacophores into derivatives of pyrimidine-2(1*H*)-thione (PMT), 2-pyridone (P), pyridine-2(1*H*)-thione (PT), dihydropyrimidine-2(1*H*)-thione (DHPMT), dihydropyridin-2(1*H*)-one (DHP), and dihydropyridine-2(1*H*)-thione (DHPT) rings enhances antitumor activity.

**Methods:**

A range of novel pyridine- and pyrimidine-based compounds were synthesized and assessed for their anticancer properties against the melanoma A375 cell line. The two most potent compounds (**16b** and **29**) were then chosen for further evaluation of their effects on non-cancerous human dermal fibroblasts, cancer cell apoptosis, cell cycle phase distribution, and tubulin polymerization. Furthermore, *in silico* analyses were performed to assess the pharmacokinetics, toxicity, drug-likeness, and molecular target of the selected compounds.

**Results:**

Among the 33 compounds tested, pyridine analogs **16b** and **29** demonstrated the strongest antiproliferative activity (with IC_50_ values of 1.85 ± 0.44 µM and 4.85 ± 1.67 µM, respectively) and selectivity (SI=65.08 and SI> 100, respectively) against cancer cells. Additional studies revealed that compound **16b**, which features a thiophene ring at the C-5 position and a 3,4,5-trimethoxyphenyl (TMP) group, showed the most promising cell cycle arrest and tubulin polymerization inhibition (IC_50_=37.26 ± 10.86 µM), resulting in cancer cell apoptosis. *In silico* ADMET analysis confirmed the drug- likeness of the synthesized compounds.

**Conclusion:**

This research reinforced the significance of heterocyclic rings as valuable pharmacophores. Additionally, it highlighted the antiproliferative and antimitotic potential of modified pyridine derivatives.

## 1 INTRODUCTION

According to the latest definition, the term “cancer” refers to a disease characterized by the uncontrolled proliferation of transformed cells. Healthy cells transform into cancer cells as a result of the genetic and epigenetic changes that accumulate within a population of cells, leading to the lethal phenotype [1]. Mattiuzzi and Lippi, referring to the Disability-Adjusted Life Years (DALY), indicate cancer as the highest clinical, social, and economic burden, even ahead of ischemic heart disease and stroke [2]. In 2022, nearly 20 million new cancer cases and 9.7 million cancer-related deaths were reported worldwide. Estimates show that approximately one in five people will develop cancer in their lifetime, and one in nine men and one in twelve women will die from it [3]. The significant death rate from cancer, coupled with the limited effectiveness of available therapies and adverse effects of current treatments, has prompted the exploration of novel, more efficient anticancer strategies. Extensive research in this field may be related to the synthesis of new drugs [4], the development of new methods of targeted drug delivery [5], or the search for therapies that combine various treatment modalities [6].

The current paper provides information about new small molecules with anticancer properties, based on pyridine and pyrimidine core, equipped mainly with heterocyclic rings. It is estimated that more than 85% of biologically active compounds are heterocycles or contain heterocyclic groups. Among them, compounds containing nitrogen atoms (like pyridines and pyrimidines) have particular importance. Research on US FDA (The United States Food and Drug Administration) databases revealed that about 60% of unique small- molecule drugs, comprised nitrogen based heterocycles, emphasizing their structural significance in drug design and discovery [7]. Moreover, pyridine and pyrimidine derivatives show promising anticancer activity [8]. There are some of the approved anticancer drugs with pyridine pharmacophores: sorafenib, regorafenib, vismodegib, and crizotinib, and medications containing pyrimidine moiety: gemcitabine, 5-fluorouracil, and floxuridine [9]. A comprehensive analysis by Dwivedi *et al.* found that 33% of the 54 pyridine-containing drugs approved by the US FDA between 2014 and 2023 were anticancer drugs [10].

Our previously published data showed that dihydro- derivatives of pyridine-2(1*H*)-thione (DHPT) and pyrimidine-2(1*H*)-thione (DHPMT) revealed promising antiproliferative effects, including cell cycle arrest at mitosis, disturbed mitotic spindle formation and inhibition of tubulin polymerization [11]. Further study revealed that the introduction of the thiophene ring to the structure of 3,4-dihydropyridine-2(1*H*)-thione contributed to increased antiproliferative activity and selectivity against cancer cells [12]. Nowadays, the design of hybrids has proven to be a promising approach in drug discovery [13]. This technique involves merging two or more pharmacophores of bioactive scaffolds to develop a drug with improved affinity and activity compared to its parent molecules. Hybrid anticancer drugs are of significant interest due to their potential to overcome the pharmacokinetic limitations of conventional anticancer drugs. Consequently, pyridine hybrids with various heterocyclic moieties have shown biological significance as antiproliferative agents [14].

In the current work, we would like to verify hypothesis assuming that introduction of heterocyclic pharmacophores into pyrimidine-2(1*H*)-thione (PMT), 2-pyridone (P), pyridine-2(1*H*)-thione (PT), dihydropyrimidine-2(1*H*)-thione (DHPMT), dihydropyridin-2 (1*H*)-one (DHP) and dihydropyridine-2(1*H*)-thione (DHPT) rings derivatives increase antitumor activity. For this purpose, among a broad spectrum of heterocyclic ring systems, thiophene [15], benzothiophene, furan [16], triazole [17], indole [18], and benzoxazole/benzothiazole [19, 20] rings were mainly selected. We incorporated an additional heterocyclic ring into the base pyridine and pyrimidine structures through coupling and addition reactions. The combination of heterocyclic rings *via* coupling reactions is a common method in synthesizing bioactive compounds [21, 22]. Nevertheless, there are limited examples of regioselective addition of organolithium and organomagnesium heterocyclic reagents to pyrimidine-2(1*H*)-thiones, as reported by Sośnicki *et al.* [23]. Besides, over the last decade numerous tubulin inhibitors functionalized with trimethoxyphenyl (TMP) group have been discovered as potent anticancer agents [24], we decided also to apply this functionality in some cases as substituents. It is widely documented that individual enantiomers of dihydropyrimidinethiones exhibit greater biological activity compared to their racemic mixtures. Therefore, in addition to synthesis of racemic hybrid compounds, we aimed to synthesize a 4,5-diaryl derivative of 3,4-dihydropyridine-2(1*H*)-thione in a form of single enantiomer or enriched amount of one enantiomer. Implementing the above experimental plan several series of PMT, P, PT, DHPMT, DHP, and DHPT derivatives were synthesized, followed by the antiproliferative and antitubulin studies.

## 2 MATERIALS AND METHODS

### 2.1 Chemical Part

Details of the synthesis and spectral data of the compounds are provided in Supplementary Materials (Chemical part). Copy of ^1^H and ^13^C NMR spectra (Fig. **S2.1**-**51**) are provided in the Supplementary Materials.

### 2.2 WST- 1 Assay

The A375 malignant melanoma cell line (ECACC 88113005, Salisbury, UK) was procured from the European Collection of Authenticated Cell Cultures (ECACC). The primary culture of human dermal fibroblasts (HDF) was derived from skin according to the protocol approved by the Ethical Committee of Pomeranian Medical University in Szczecin (KB-0012/02/18). The detailed protocol of fibroblast isolation, authentication, and cell culture conditions was previously described [25]. Both cell lines were cultivated in a humidified incubator under conditions of 5% CO_2_ and a temperature of 37°C, using Dulbecco’s modified Eagle’s medium (DMEM) with 4500 mg/L glucose (Sigma-Aldrich Merck Group, St. Louis, MO, USA) enriched with 10% heat-inactivated fetal bovine serum (FBS, EURx, Gdańsk, Poland), 2 mM L-glutamine (Sigma-Aldrich Merck Group, St. Louis, MO, USA), and penicillin-streptomycin solution (Sigma-Aldrich Merck Group, St. Louis, MO, USA). Regular assessments for the presence of mycoplasma were conducted using the MycoAlert^®^ Mycoplasma Detection Kit (Lonza, Rockland, ME, USA).

The evaluation of the antiproliferative activity of the compounds was performed using the Cell Proliferation Reagent WST-1 assay (Roche, Germany). This assay relies on the reduction of tetrazolium salt to formazan, with the final amount of formazan proportional to the number of viable cells. In our study, A375 cells (2×10^3^) and HDF (3×10^3^) were initially seeded into 96-well plates and cultured in a humidified atmosphere of 5% CO_2_ at 37°C. 24 hours later, the culture medium was replaced with a medium containing the tested compounds for a 48-hour incubation period at specified final concentrations. Tested compounds were dissolved in dimethyl sulfoxide (DMSO, Sigma-Aldrich Merck Group, St. Louis, MO, USA), ensuring that the final DMSO concentration did not exceed 0.2%. For control purposes, cells without the tested compounds, which contained only the vehicle (DMSO), were used. Additionally, a blank sample consisting of tested compounds in a medium (without cells) was included. After the 48-hour incubation period, the WST-1 reagent was introduced and allowed to incubate with the cells for 30 minutes. The absorbance was subsequently measured at 450 nm with background correction at 620 nm using a microplate reader (Infinite 200 Pro, Tecan, Männedorf, Switzerland). Cell viability was calculated according to the following formula: [(Atest–Ablank)/ (Acontrol − Ablank)] × 100%. These measurements were conducted in triplicate for at least three independent experiments.

In the screening step, 10 µM solutions of all 33 compounds were tested on A375 cells. Only compounds that satisfy pre-determined criteria (decreased cell proliferation to at least 50%) will progress to further study to estimate the inhibitory concentration causing 50% growth inhibition (IC_50_). Selected compounds (**2c**, **18e**, **16b**, **29**) were tested at concentrations of 0.1, 1, 10, 50, and 100 µM. The IC_50_ values were determined using an online calculator (AAT Bioquest, Inc., Quest Graph™ IC_50_ Calculator (v.1). Retrieved from https://www.aatbio.com/tools/IC_50_-calculator-v1, accessed on: 2022, December 12).

To assess the selectivity of the most active compounds (**16b** and **29**), we calculated the selectivity index (SI), which is calculated as the ratio of the IC_50_ for non-cancer cells to the IC_50_ for cancer cells. For that purpose, non-tumor HDF cells were used and the concentrations of tested compounds were increased to 200 µM (0.4% DMSO) and 500 µM (1% DMSO). Appropriate controls were prepared for higher concentrations of DMSO. However, the IC_50_ values and SI for **29** need to be estimated because, at 500 µM, the HDF viability was still 85.92 ± 7.47%, while at higher concentrations, compound precipitation was observed.

### 2.3 Apoptosis Assay and Live-cell Imaging

In this study, A375 cells were initially seeded into 24-well plates at a density of 1×10^4^ cells per well. After 24 hours, the cell culture medium was carefully removed, and a new medium containing **16b** and **29** at varying final concentrations (1, 5, and 10 µM) was added, followed by an additional 48-hour incubation. Cells in a medium containing 0.2% DMSO served as the control group. After the treatment period, cells were washed with phosphate-buffered saline (PBS), and both the cell medium and PBS wash were retained. The cells were then detached using trypsin and centrifuged. The resulting cell pellet was suspended in a fresh medium after discarding the supernatant. An equivalent volume of Muse Annexin V & Dead Cell Reagent (Luminex Corporation, Austin, TX, USA) was added and thoroughly mixed through repeated pipetting. The staining procedure was conducted for 20 minutes at room temperature in the absence of light. The Muse Cell Analyzer (Luminex Corporation, Austin, TX, USA) was utilized for analysis. This assay detects phosphatidylserine (PS) on apoptotic cell surfaces using annexin V-PE (phycoerythrin) binding. A 7-AAD (7-aminoactinomycin D) marker was used to assess cell membrane integrity. The dual staining approach enabled the identification of four distinct cell groups: viable (Annexin V-PE− and 7-AAD−), early apoptotic (Annexin V-PE+ and 7-AAD−), late apoptotic/dead (Annexin V-PE+ and 7-AAD+), and primarily nuclear debris (Annexin V-PE− and 7-AAD+). The findings were based on at least three separate experimental runs.

For live-cell imaging, A375 cells were seeded into 96-well plates at a density of 2×10^3^ cells per well and allowed to grow for 24 h before imaging. Next, the cell culture medium was carefully removed, and new medium containing **16b** (at 2 µM and 10 µM) and **29** (at 5 µM and 10 µM) was added and the cell culture plate was placed in the imaging chamber of Agilent BioTek Lionheart FX automated microscope (Agilent Technologies, Inc., Santa Clara, CA, USA) equipped with a Plan Fluorite 20x/0.75 objective (Olympus, Nishi-Shinjuki, Tokyo, Japan), and a FLIR Blackfly BFLY-U3-23S6M camera (Teledyne FLIR, Wilsonville, OR, USA). The temperature in the imaging chamber was set to 37°C and CO_2_ level to 5%. The bright-field images were acquired every 5 minutes for 48 hours. Lower tested concentrations were close to IC_50_ values (1.85 ± 0.44 µM and 4.85 ± 1.67 µM for **16b** and **29**, respectively), 10 µM is the highest concentration tested during apoptosis assay.

### 2.4 Cell Cycle

For cell cycle analysis, A375 cells were initially plated in 6-well plates at a density of 1×10^5^ cells per well and allowed to culture for 24 hours. Following this incubation period, the culture medium was replaced with fresh medium containing **16b** and **29** at final concentrations of 1, 5, and 10 µM, after which the cells were incubated for an additional 5 hours. For control purposes, cells cultured in a medium containing 0.2% DMSO without treatment were utilized. The experimental procedure involved several steps: initially, the cells were rinsed with PBS, and then collected *via* trypsinization. The resulting cell pellet underwent another PBS wash before being fixed with 70% cold ethanol (Chempur, Piekary Śląskie, Polska) at -20°C for at least 3 hours. Before analysis, the ethanol-fixed cells were centrifuged, washed again with PBS, and then treated with Muse Cell Cycle Reagent (Luminex Corporation, Austin, TX, USA), which includes propidium iodide (PI) and RNAse. This staining process was carried out for 30 minutes at room temperature in the absence of light. The assay relies on PI-based staining of DNA content to differentiate and quantify the percentage of cells in the G0/G1, S, and G2/M phases of the cell cycle. The analysis was performed using the Muse Cell Analyzer (Luminex Corporation, Austin, TX, USA). The obtained findings were derived from a minimum of three independent experiments.

### 2.5 Cell Free Tubulin Polymerization Assay

The tubulin polymerization process was evaluated using a tubulin (>99% pure) and fluorescence-based tubulin polymerization kit (Cytoskeleton, Denver, CO, USA). The effects of compounds **16b** and **29** (at final concentrations of 1, 10, 50, 100 µM) on tubulin polymerization were evaluated in cell-free conditions. Paclitaxel (PTX) and vinblastine (VBL) (3 µM) were used as references, and DMSO (0.2%) was used as the vehicle control. This assay tracks the process of tubulin polymerization through an increase in fluorescence resulting from the addition of a fluorescence reporter to microtubules as polymerization occurs. After incubating the tested compounds at 37°C for 1 minute, the icy tubulin reaction mixture (2 mg/mL tubulin, 1.0 mM GTP, 15% glycerol in buffer containing 80 mM PIPES, 2.0 mM MgCl_2_, 0.5 mM EGTA, pH 6.9, and 10 µM fluorescent reporter) was added to the samples, which were then mixed and monitored using a spectrophotometric microplate reader (Infinite 200 Pro, Tecan) at 1-minute intervals for 90 minutes at 37°C (excitation: 360 nm, emission: 450 nm). The software installed on the plate reader was used to compute the maximum slope (Vmax) of the growth phase, measured in RFU/min, as outlined in the manufacturer's instructions. Each polymerization curve's Vmax was then standardized against the control curve's Vmax. The IC_50_ values were determined using an online tool (AAT Bioquest, Inc., Quest Graph™ IC_50_ Calculator ((v.1). Retrieved from https://www.aatbio.com/tools/IC_50_-calculator-v1, accessed on: 2024, May 6).

### 2.6 Quantitative Cell-based Tubulin Depolymerization and Stabilization Assays

In a white 96-well plate, A375 cells (4×10^3^) were planted and maintained under standard conditions for 48 hours. To assess depolymerization [26], the cells were subsequently exposed to **16b** and **29** at various concentrations (1, 5, 10, 25, 50, and 100 µM) for 30 minutes. A 0.2% DMSO solution served as the negative control, while combretastatin-4 CA-4 (1 µM) was used as the positive control. The findings are presented as the percentage of remaining microtubules (MTs), with 100% corresponding to cells treated with 0.2% DMSO. For stabilization assay [27] cells were treated for 90 minutes with **16b** and **29** at the same concentrations. Then all cells were treated with 0.5 µM of CA-4 for 30 minutes. Additional wells including cells treated with DMSO (0.2%) without CA-4 were also included. Results are expressed as % of MTs resistant to CA-4 induced depolymerization, with 100% corresponding to cells treated with DMSO (0.2%) without CA-4 and 0% corresponding to cells treated with DMSO (0.2%) and CA-4. PTX at 1 µM served as a positive control inhibitor.

Following the removal of the culture medium, according to the previously described protocol [26], cells underwent permeabilization for 10 minutes at 37°C. This permeabilization process was carried out using OPT buffer (80 mM Pipes, 1 mM EGTA, 1 mM MgCl2, 0.5% Triton X-100, and 10% glycerol, pH 6.8). Subsequently, the cells were fixed overnight at room temperature using a 4% formaldehyde solution (Sigma-Aldrich Merck Group, St. Louis, MO, USA) diluted in PBS. A thorough washing step was performed, involving three rinses with PBS containing 0.1% Tween-20. For immunostaining, a primary mouse anti-α-tubulin monoclonal antibody (Sigma-Aldrich Merck Group, St. Louis, MO, USA) was utilized. This antibody was diluted to a concentration of 1:5000 in a solution comprising PBS with 2% bovine serum albumin (BSA, Sigma-Aldrich Merck Group, St. Louis, MO, USA) and 0.1% Tween-20 (Bio-Rad, Hercules, CA, USA). The antibody was incubated with the cells for 30 minutes at room temperature. Following this, three additional washes were performed. In the subsequent step, a secondary mouse IgGκ BP conjugated to horseradish peroxidase (HRP) (Santa Cruz Biotechnology, Dallas, TX, USA) was diluted 1:2000 in a solution comprising PBS with 2% BSA and 0.1% Tween-20, and it was allowed to incubate with the cells for a duration of 30 minutes. The cells were then subjected to another wash with PBS. Finally, an chemiluminescence (ECL) substrate (Western Bright Sirius Chemiluminescent Detection Kit, Advansta, San Jose, CA, USA) was added to each well. The luminescence was measured after 2 minutes using a spectrophotometric microplate reader (Infinite 200 Pro, Tecan, Männedorf, Switzerland).

### 2.7 ADMET Properties

The online *in silico* prediction model ADMETlab 2.0 was used to determine ADMET attributes such as absorption, distribution, metabolism, excretion, toxicity, and physicochemical properties of all chemicals. Drug similarity properties were assessed using Lipinski's rule of five, Pfizer rule, GSK rule and Golden Triangle. The tested compounds were drawn using the JMSE editor available on the ADMETlab 2.0.

### 2.8 Molecular Docking

Molecular docking was carried out using CB- Dock2 online platform [28] (https://cadd.labshare.cn/cb-dock2/, accessed on 9 January 2025) and the best binding model was selected out of 5 proposed using the most negative binding energy (Vina score) as it indicates strongest binding. Tubulin-Colchicine: Stathmin-like domain complex crystal structure (PDB ID: 1SA0) was aquired from RCSB PDB [29]. Chemical structures of tested compounds were drawn using JMSE editor embedded in CB-Dock webserver.

### 2.9 Statistical Analysis

The results have been expressed as the mean ± standard deviation (SD). To evaluate differences between the cells treated with compounds and the control (non-treated) cells, as well as variations between the tumor cells (A375) and the primary culture of HDF, the t-student test was employed. A *p*-value of less than 0.05 was regarded as statistically significant. Statistical analysis was conducted using Statistica 13.3 software (Statsoft, Tulsa, OK, USA).

## 3 RESULTS AND DISCUSSION

### 3.1 Chemical Part

#### 3.1.1 Synthesis of the Studied Compounds

**Scheme 1:**
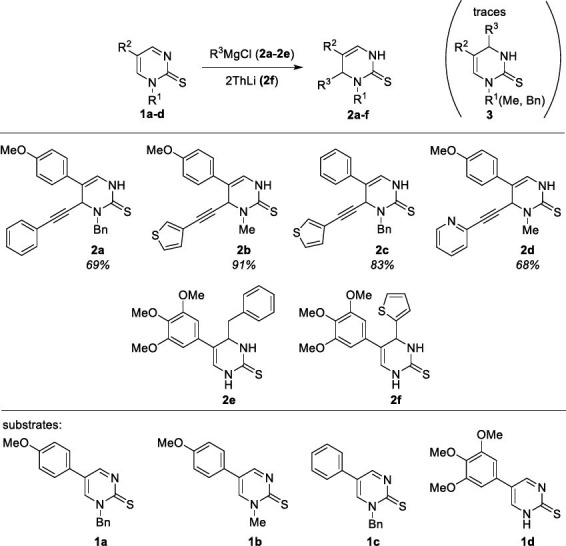
Synthesis of functionalized 3,4-dihydropyrimidine-2(1*H*)-thiones (DHPT) **2a**-**2f**.

Functionalized dihydropyrimidinethione derivatives (DHPMT), depicted in Scheme **1**, were obtained by the addition of magnesium or lithium reagent to pyrimidinethione **1** in a similar method we described earlier [30]. However, in contrast **2e** and **2f** were obtained by addition of benzylmagnesium chloride and 2-thienyllithium to *N*H pyrimidine-2-thione **1c**, respectively, the introduction of alkynyl moiety to *N*-Me(Bn) substituted substrates, leading regioselectively to compounds **2a**-**2d**, was carried out using (arylethynyl)magnesium chloride prepared from corresponding terminal alkyne and *i-*PrMgCl. However, as far as regioselectivity is concerned, it was found that the addition took place at the C4 position (next to the substituted nitrogen atom), which is the opposite result to the addition regioselectivity, previously observed for aryl Grignard compounds, because in former cases addition took place at carbon atom adjacent to an unsubstituted nitrogen atom. Observed regioselectivity was evidenced by coupling constants analysis and Overhauser effects observed in ^1^H,^1^H NOESY spectrum of **2b** (Supplementary materials Fig. **S1**).

**Scheme 2:**
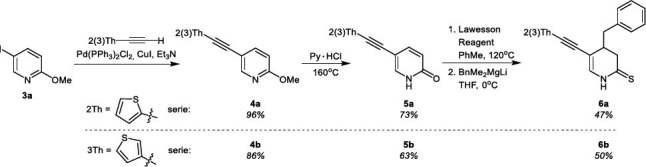
Synthesis of 5-functionalized *N*H 3,4-dihydropyridine-2(1*H*)-thione (DHPT) **6a** and **6b**.

Further studies comprised the syntheses of 5-thienylalkynyl, 4-benzyl functionalized 3,4-dihydropyridino-2-thiones (DHPT) **6a**, **6b**. These compounds were synthesized by a four-step procedure, summarized in Scheme **2**, starting from 5-iodo-2-methoxypyridine (**3a**) and appropriate thienylalkynes, followed by transformation to 2-pyridones **5** and corresponding 2-thiopyridones, which finally were used in the addition of lithium benzyldimethylmagnesiate [31] to obtain compounds **6**. The reaction products of each stage were obtained with good yields. However, it should be noted that due to the instability of the 2-thiopyridone (PT) derivatives (**5a** and **5b**), they were used for the synthesis of compounds **6a** and **6b** immediately after purification.

**Scheme 3:**
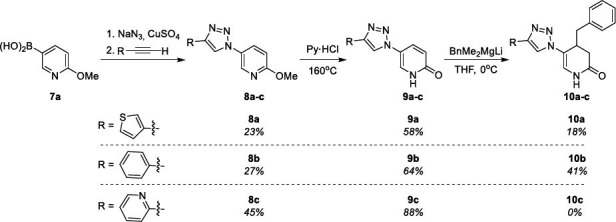
Synthesis of 5-triazole functionalized pyridin-2(1*H*)-ones (P) **8** and dihydro pyridin-2(1*H*)-ones (DHP) **9**-**10**.

**Scheme 4:**
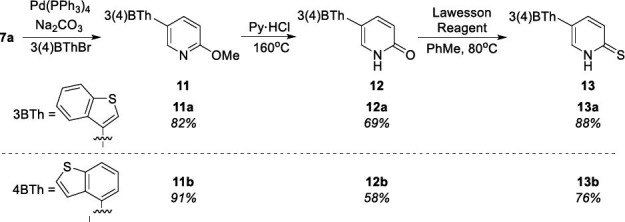
Synthesis of 3(4)-benzo[*b*]thiophen-4-yl functionalized compounds **11**-**13**.

Synthesis of 5-triazole functionalized 2-pyridine derivatives **8**-**10** is presented in Scheme **3**. It comprises the initial synthesis of 5-triazole-2-methoxypyridine derivatives **8** from boronic acid **7a**, which were converted into the corresponding *N*H pyridin-2(1*H*)-ones **9**, which were in turn treated with lithium benzyldimethylmagnesiate giving products **10a** and **10b**. The negative result for derivative **10c** shows that the pyridyl substituent prevents the addition reaction.

(6-Methoxypyridin-3-yl)boronic acid (**7a**) was used for the synthesis of 5-(benzo[*b*]thiophen-3-yl)- and 5-(benzo[*b*]thiophen-4-yl)-functionalized *N*H 2-pyridine-2(1*H*)-thiones (PT) **13a** and **13b** starting from Suzuki-Miyaura cross-coupling reaction [32] leading to **11a**/**11b**, following by transformations to *N*H 2-pyridones (P) **12a**/**12b** in pyridine hydrochloride at 160°C [33], ending with thionation by applying Lawesson reagent [34] (Scheme **4**).

**Scheme 5:**
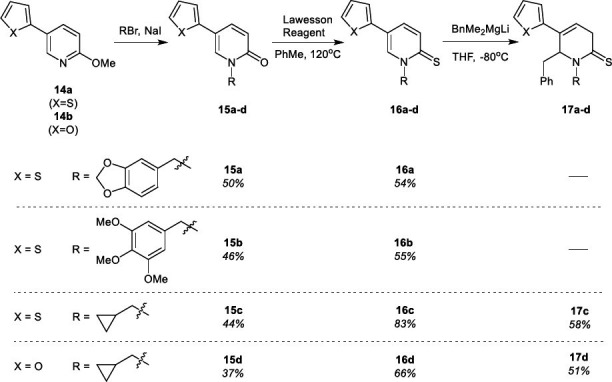
Synthesis of 5-(thiophen-2-yl) and 5-(furan-2-yl) derivatives of pyridin-2(1*H*)-ones (P) (**15a-d**), pyridine-2(1*H*)-thiones (PT) (**16a-d**) and 3,6-dihydropyridine-2(1*H*)-thiones (DHPT) (**17c**,**d**).

Synthesis of 5-(2-thiophene or 2-furan)-functionalized *N*-substituted **PT** derivatives **16a**-**c** and **DHPT** derivatives **17c** and **17d** was achieved by standard procedures described by us earlier, starting from 5-functionalized 2-methoxypyridines **14** [35]. The sequence of reactions illustrated on Scheme **5** comprised synthesis of pyridin-2(1*H*)-ones **15a-d,** according to the modified Bowman and Bridges procedure [36], followed by thionation using Lawesson reagent, leading to pyridine-2(1*H*)-thiones **16a-d**, two of which were finally converted into **DHPT** derivatives **17c** and **17d**.

**Scheme 6:**
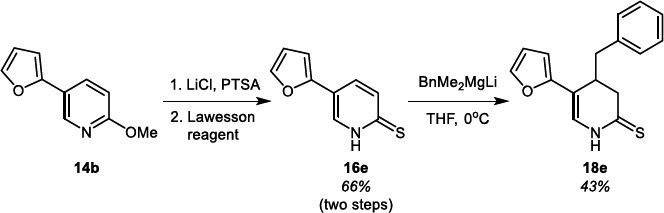
Synthesis of compound **18e.**

**Scheme 7:**
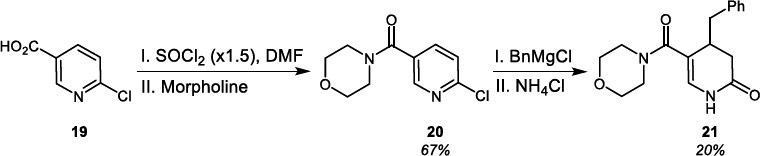
Synthesis of compound **21.**

The synthesis of 5-furyl, *N*H, 4-benzyl functionalized DHPT compound **18e** is depicted in Scheme **6**. We also attempted to synthesize *N*H DHP derivative **21** (Scheme **7**) due to the addition of BnMgCl to the 2-chloropyridine derivative **20** followed by hydrolysis. This route, based on the procedure described earlier [37], turned out to be not very efficient, as product **21** was obtained in a 20% yield.

**Scheme 8:**
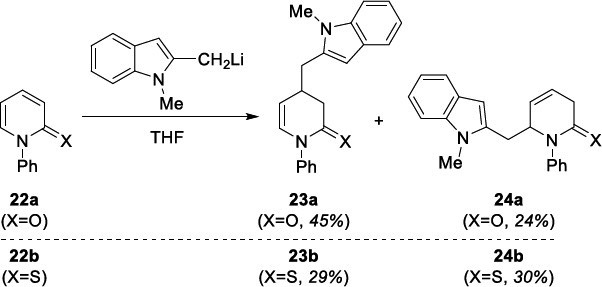
Introduction of indole ring into 2-(thio)pyridine.

**Scheme 9:**
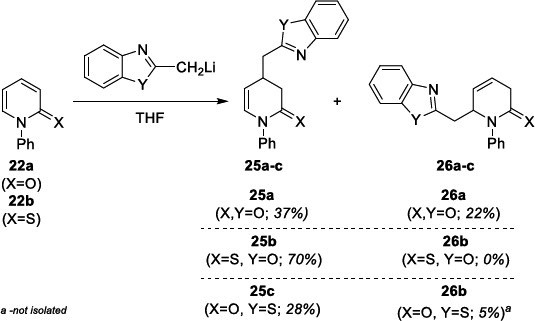
Introduction of benzoxazole/benzothiazole ring into 2-pyridone (P) and 2-thiopyridone (PT).

Searching for a new possibility of introducing heterocyclic rings into the 2-(thio)pyridone ring, we made a successful attempt to add lithiated derivatives of 1,2-dimethylindole (Scheme **8**), 2-methyloxazole and 2-methylthiazole (Scheme **9**) to N-Ph substituted pyridin-2(1*H*)-one **22a** and 2-pyridone-2(1*H*)-thione **22b**. As a result, the syntheses of indolilomethyl functionalized DHP(T) products **23** and **24** were obtained in good total yields, however in low regioselectivity (Scheme **8**). In the case of the addition of lithiated 2-methylbenzox(thi)azole to **22**, the reactions proceeded in various yields and selectivity. However, the synthesis of benzoxazole and 3,4-dihydropyridine-2(1*H*)-thione hybride **25b** was the most effective (Scheme **9**).

**Scheme 10:**
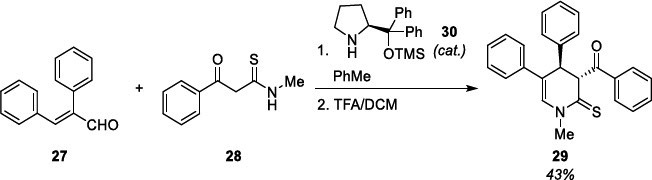
Synthesis of optically active 4,5-diphenyl-substituted DHPT derivative **29.**

The above-described compounds were obtained in the form of racemates. However, the need to obtain an optically active derivative prompted us finally to attempt to synthesize an optically active 4,5-diaryl derivative of 3,4-dihydropyrimidinothione (DHPT) by cyclization using an optically active pyrrolidine catalyst, following the reaction described for the cinnamaldehyde [38]. In our attempt, we used 2-phenylcinnamaldehyde (**27**) and *N*-methyl-3-oxo-3-phenylpropanethioamide [39] (**28**) as reagents and (S)-2-(diphenyl((trimethylsilyl)oxy)-methyl)pyrrolidine (**30**) as catalyst at the presence of benzoic acid in toluene, following by treatment with TFA in DCM (Scheme **10**). Product **29** was obtained in 43% yield and was characterized with specific rotation [α]20D = -52.58 deg (MeOH). Details of the synthesis of the compounds are provided in the Supplementary Materials (Chemical part).

### 3.2 Screening Results

All compounds were tested initially at a single concentration (10 µM) against melanoma A375 cells, which were defined as the most sensitive against pyridine analogs in our previous study [12]. The WST-1 assay was used to evaluate the antiproliferative activity and the results are summarized in Fig. (**1**) and Table **S1**. Only compounds that satisfactory pre-determined threshold inhibition criteria (decreased cell proliferation to at least 50%) were progressed to further study to estimate the concentration causing 50% growth inhibition (IC_50_), (Figs. **S2** and **S3**). As shown in Table **1** compounds **16b** and **29** showed the highest inhibition of A375 cells growth with IC_50_ values of 1.85 ± 0.44 µM and 4.85 ± 1.67 µM, respectively. Among them, compound **16b** that bears tiophene ring at C-5 and 3,4,5-trimethoxyphenyl (TMP) moiety constituting a part of N-3,4,5-trimethoxybenzyl substituent, displayed the most potent activity. Our recently published data have also revealed that among tested 22 pyridine analogs, the compound with benzyl group at C-4 and a thiophene ring at C-5 of a 3,4-dihydropyridine-2(1*H*)-thione (S22) showed the most promising anticancer activity (IC_50_ equal 1.71 ± 0.58 µM) and selectivity (SI = 21.09) against melanoma A375 cells [12]. Both **16b** and **29** belong to pyridine analogs. Our previously published data also revealed that 4-benzyl-5-phenyl-3,4-dihydropyridine-2(1*H*)-thione showed higher antiproliferative activity than its pyrimidine bioisoster and a prototype agent monastrol [11]. The two most active compounds (**16b** and **29**) were selected to further biological evaluation.

At first, the WST-1 assay was also used to calculate the selectivity index (SI), which is defined as the ratio of IC_50_ values for normal cells to IC_50_ values for cancer cells. Cancer A375 cell line and non-tumor human dermal fibroblasts (HDF) were used for that purpose. According to the “selectivity criteria” (SI > 10) [40] compounds **16b** as well as **29** could be considered selective (Table **1**). However, the IC_50_ values and SI for **29** need to be estimated because, at 500 µM, the HDF viability was still 85.92 ± 7.47%, while at higher concentrations precipitation was observed. The results from DHF cells (WST-1 assay) are shown in Supplementary Materials (Fig. **S4**). For potential drugs, selectivity towards cancer cells is as important a parameter as potency, because it increases the chances of reducing potential side effects of the therapy. As shown by Nagender *et al.* among all the screened novel hydrazone and azole functionalized pyrazolo [3,4-b]pyridine derivatives, compound **10d** with trifluoromethylthio substituent in the phenyl ring demonstrated the most promising activity (IC_50_= 3.2 ± 0.11 µM) and selectivity (SI= 24.03) against A549 lung cancer cells [41].

### 3.3 Apoptosis Assay and Live-cell Imaging

Apoptosis is widely recognized as a key mechanism of drug-induced cell death. Therefore, in the next step, to evaluate this process, an apoptotic marker was assessed using flow cytometry. Representative individual experiments, which feature cell populations divided into quadrants dot plots based on annexin V-PE/7-AAD staining, are illustrated in Fig. (**S5**). For improved clarity, Fig. (**2**) displays only the population of annexin-positive cells (both early and late apoptotic), revealing a significant increase in the number of apoptotic cells in a concentration-dependent manner. Based on the obtained results we can conclude that compound **16b** is more effective in inducing cancer cell apoptosis. Namely, the percentage of annexin-positive cells increased from 4.20 ± 0.57% (control) to 18.43 ± 2.90% and 30.50 ± 1.27% in the case of respectively 5 µM and 10 µM of **16b**, whereas the percentage of annexin-positive cells increased from 4.23 ± 0.35% (control) to 8.28 ± 1.80% and 21.38 ± 1.55% after treatment with **29** at 5 µM and 10 µM, respectively. The cytotoxicity of cells treated with **16b** and **29** was unexpectedly lower than proliferation inhibition according to WST-1 assay. This might be explained by the fact that compounds have a more potent antiproliferative effect rather than a direct cytotoxic effect. This relationship has been observed before with a benzofuran microtubule inhibitor, which was more effective at inhibiting cell proliferation than causing cytotoxicity [42].

Simultaneously with apoptosis assay, we analyzed the effect of compounds **16b** (at 2 µM and 10 µM) and **29** (at 5 µM and 10 µM), using time-lapse microscopy (Supplementary Materials movies 1-6). Lower tested concentrations were close to IC_50_ values (1.85 ± 0.44 µM and 4.85 ± 1.67 µM for **16b** and **29**, respectively). In control (with 0.2% DMSO) A375 cells divided intensively to reach confluence after 48 hours of treatment (Movie 1). In the case of **16b** treated cells (at 2 µM), cells also divided, but less effectively, divisions lasted longer, and finally, the confluence was lower than that in the control well (Movie 2). When melanoma cells were treated with **16b** at 10 µM, arrested mitosis resulted in mitotic slippage, leading to apoptosis or formation of binucleated cells (Movie 3). The videomicroscopy analysis of the cells treated with compound **29** at 5 µM (Movie 4) revealed finally more normal cells in the field of view than in the case of the same concentration of **16b**, which is consistent with the results from the apoptosis study. However, during 48 hour time-lapse video of **29** treated cells, we were able to observe more cases of programmed cells death with characteristic apoptotic bodies and fianlly more spherical cells (Movie 5). It is difficult to determine whether the rounded cells formed from apoptotic bodies are actually dead cells or whether we are dealing with the onset of anastasis. Anastasis is one of the resistance mechanisms of cancer cells, which may return to their initial morphology and proliferation activity, after cessation of exposure to toxic agents [43, 44]. Combretastatin-4 (CA-4) at 1 µM, used as positive control, caused the formation of characteristic protrusions, indicating changes in the organization of the cytoskeleton leading to apoptosis with typical apoptotic bodies (Movie 6).

### 3.4 Cell Cycle Distribution

In the consideration of prominent antiproliferative activity of selected compounds, the effect of **16b** and **29** on cell cycle progression using propidium iodide (PI) staining in A375 cells was examined. As illustrated in Fig. (**3**), after 5 h incubation only compound **16b**, in concentration depended manner, inhibited cell cycle at G2/M phase. The percentage of A375 cells in the G2/M phase increased from 31.58 ± 2.52% in the control to 33.79 ± 0.46%, 39.33 ± 5.27% and 44.52 ± 4.00% for 1 µM, 5 µM and 10 µM **16b**, respectively. Representative individual experiments, are shown in Fig. (**S6**). Most of pyridine based compounds, including coumarin hybrids with pyridine and fused pyridine moieties [45], pyridine-chalcone derivatives [46], pyrazolo [3,4-b]pyridine-bridged derivatives of CA-4 [47], pyridine-based hydroxamates [48] target mitosis at G2/M phase.

### 3.5 Cell Free Tubulin Polymerization Assay

**Fig. (4):**
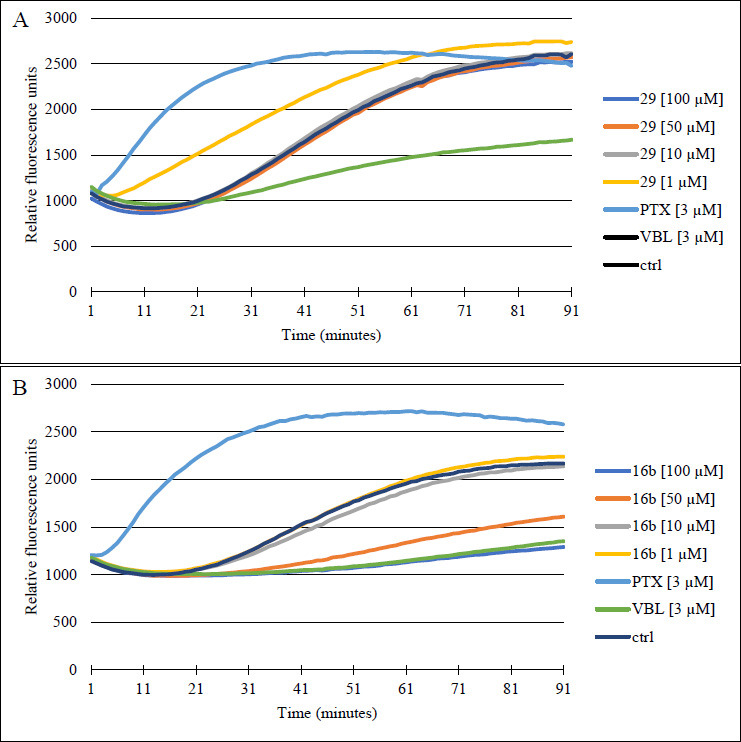
Effects of **16b** (**A**) and **29** (**B**) derivatives on tubulin polymerization in cell-free conditions. Increased relative fluorescence units indicate tubulin polymerization. Paclitaxel (PTX) and vinblastine (VBL) were used as references, while DMSO (0.2%) served as a vehicle control. The results shown are the mean of three independent experiments. Error bars have been omitted for clarity (chart containing error bars has been included in the Supplementary Materials Fig. **S7**).

To assess the direct interaction between compounds **16b**, **29** and tubulin, we evaluated their capacity to interfere with pure tubulin under cell-free conditions using a fluorescence-based tubulin polymerization assay. We compared the activity of these compounds to paclitaxel (PTX), vinblastine (VBL), and 0.2% DMSO, which served as references and a negative control, respectively. As shown in Figs. (**4** and **S7**), 0.2% DMSO (represented by black curves) had no direct effect on spontaneous tubulin self-assembly. This was evident from the presence of three distinct stages of microtubule formation: nucleation, growth, and steady-state inhibition. In comparison to the control, PTX (3 µM) reduced the duration of the nucleation phase and increased the Vmax (maximum slope values for the growth phase). Conversely, VBL (3 µM) suppressed tubulin polymerization, resulting in decreased Vmax and reduced final polymer mass of protein. The overlapping curves of of negative control (black line) and compound **29** at all tested concentrations (1-100 µM) proved that this derivative is not interacting with tubulin (Fig. **4B**). Whereas, overlapping curves of VBL - well-characterized microtubule-destabilizing agent and compound **16b** at 100 µM indicated that **16b** acted as tubulin polymerization inhibitors (Fig. **4A**). These observations were confirmed by IC_50_ values: 37.26 ± 10.86 µM, > 100 µM for **16b** and **29**, respectively. The obtained results for compound **16b** are not surprising because this agent, significantly arrested cells in the G2/M phase, and has the 3,4,5-trimethoxylphenyl group, which are typical features of pyridine-based tubulin polymerization inhibitors [47]. As reported by Li *et al.* the presence of TMP is necessary to maintain the anticancer activity of CA-4 analogues, while the second aromatic ring can be replaced by different groups to improve the potency and pharmacokinetic properties of a parent drug [24]. However, the current study revealed that inhibition of tubulin assembly by **16b**, observed only at the highest tested concentration, indicates that it is less active than the reference drug. Surprisingly, our previously reported 5-(thiophen-2-yl)-3,4-dihydropyridine-2(1*H*)-thione (S22) derivative, with the additional 4-benzyl group and without TMP, demonstrated comparable antitubulin activity (IC_50_=26.82 ± 15.21 µM).

We also found that higher concentrations of compound **16b** are necessary to observe its effect on pure tubulin than those above mentioned to obtain a cellular effect. Current observations are in line with our previously published data. The IC_50_ values determined against A375 using WST-1 assay for pyridine analogs S1, S22 were equal, *i.e*. 4.33 ± 1.00 µM and 1.71 ± 0.58 µM, respectively, and concentrations of these compounds required to inhibit tubulin assembly by 50% were also equal, *i.e*. 10.77 ± 3.83 µM and 26.82 ± 15.21 µM, respectively [12]. Similarly, Kamal *et al.* reported that IC_50_ for reference CA-4 varied from 0.008 to 0.072 µM (depending on cell line) according to MTT assay, and was equal 1.08 ± 0.33 µM in fluorescence-based *in vitro* tubulin polymerization assay [49]. Whereas Zheng *et al.* showed that compound **4h** did not affect tubulin polymerization even at 10 µM, in spite of the fact this pyridine-linked CA-4 analogue arrested cells in the G2/M phase as effectively as CA-4 and showed high cytotoxicity (IC_50_=0.0031- 0.089 µM) [50].

### 3.6 Quantitative Cell-based Tubulin Depolymerisation/Stabilization Assay

Proceeding to search for mechanisms of the antiproliferative activity of compounds **16b** and **29** we evaluated the effects of the tested compounds on the microtubular network of interphase cells. Various concentrations of compounds **16b** and **29** were applied to the melanoma cells, and their ability to destabilize microtubules was assessed after 30 minutes of treatment. To eliminate free tubulin dimers, while preserving intact microtubules, a permeabilization buffer (OPT buffer) was used. As shown in Fig. (**5A**), none of the compounds was able to depolymerize microtubules as effectively as the reference CA-4. In the next step, we verified the ability of our compounds to stabilize the microtubular network and prevent depolymerization caused by CA-4 (0.5 µM). Tubulin depolymerization agents like CA-4 or nocodazole bind to free tubulin dimers and prevent their incorporation into microtubules, leading to a progressive loss of the polymerized microtubule network. However, stabilized microtubules with slow dynamics have reduced exchanges of their tubulin content with the free tubulin pool and thus, they are less sensitive to drug-induced depolymerization, as described previously [27]. As shown in Fig. (**5B**), our compounds did not protect tubulin polymers against the depolymerizing effect of CA-4. Even at the highest tested concentrations (100 µM), our compounds did not show activity comparable to the positive control- known stabilizing agent – PTX at 1 µM. The aforementioned results together with the results of cell cycle analysis and cell free tubulin polymerization assay suggest that the compound **16b** is characterized by a mitotic-specific mechanism of action, which might potentially contribute to decreased side effects compared with reference drugs.

### 3.7 ADMET Analysis

The ADMET properties of compounds **16b** and **29** were calculated through the online web server ADMETlab 2.0 and are presented in detail in Tables **S2** and **S3**. According to Dulsat *et al.*, ADMETlab provides the best prediction accuracy and precision, among 18 tested free web servers [51]. In the past ten years, approximately 90% of drug developments have been unsuccessful due to inadequate pharmacokinetic profiles. Specifically, these failures can be attributed to a lack of clinical efficacy (40-50%), unmanaged toxicity (30%), and insufficient drug-like properties (10-15%). This data emphasizes how important is ADMET evaluation in the early stages of drug development. The essential factors that characterize a drug's pharmacokinetic profile include: physicochemical properties, absorption, distribution, metabolism, elimination, and toxicity [51].

Four complementary rules were used to evaluate the drug-likeness properties of tested compounds. These include: Lipinski's rule-of-five, Pfizer rule, GSK rule and Golden Triangle. Both compounds met the criteria of Lipinski’s rule of five: molecular weight (MW) < 500 Da, octanol-water partition coefficient (log P) < 5, hydrogen bond donors < 5, and hydrogen bond acceptors < 10 [52]. According to the Pfizer rule both compounds with logP > 3 and topological polar surface area (TPSA) < 75 are about 2.5 times more likely to be toxic than to be clean [53]. In the case of the GSK rule, only **16b** met its criteria, which are log P < 4 and MW < 400, which indicates that it has a more favorable ADMET profile [54]. Finally, **16b** and **29** with MW 373.08 g/mol and 383.13 g/mol and logD (the logP of compounds at physiological pH = 7.4) 3.662 and 4.319, respectively, did not satisfy Golden Triangle. This rule assumes that only compounds meeting both criteria: MW in the range of 50-200 and logD between -2 and 5 correspond to compounds reflecting optimal permeability and good metabolic stability [55] [56].

The initial phase of ADMET (absorption) is significantly influenced by the solubility of compounds in water, and is expressed as logS: -5.439, -5.166 mol/L for **16b** and **29**, respectively. Other parameters, that allow to determine absorption efficiency: human intestinal absorption (HIA) and Caco-2 permeability confirm that both compounds can efficiently penetrate the intestinal membranes. Moreover, the efflux produced by P-gp activity was excluded, since neither compound **16b** nor **29** are its substrates. As for the distribution phase, plasma protein binding (PPB), volume of distribution (Vd) and blood-brain barrier penetration (BBB) were evaluated. PPB plays a crucial role in determining drug safety, as compounds with > 90% protein-binding value tend to have a narrow therapeutic index. The PPB values equal 96.45% and 97.74%, for **16b** and **29** respectively, suggest high protein binging (and such small volume of distribution, high interaction potential, and possibly narrow therapeutic index). The calculated BBB values (0.271 and 0.873 for **16b** and **29**, respectively) indicate probability of being BBB positive (suggesting ability to cross BBB, to which lack of P-gp transport can contribute) with **16b** better BBB penetration, which may result in potential central nervous system (CNS) side effects. Based on the drug's moderate clearance (5-15 mL/min/kg) and relatively long half-life, infrequent dosing were suggested. In the case of compound **29**, toxicology evaluations revealed potential hepatotoxicity (drug-induced liver injury, DILI) and lung injury (respiratory toxicology), which may require special attention in further clinical practice.

### 3.8 Molecular Docking

**Fig. (6):**
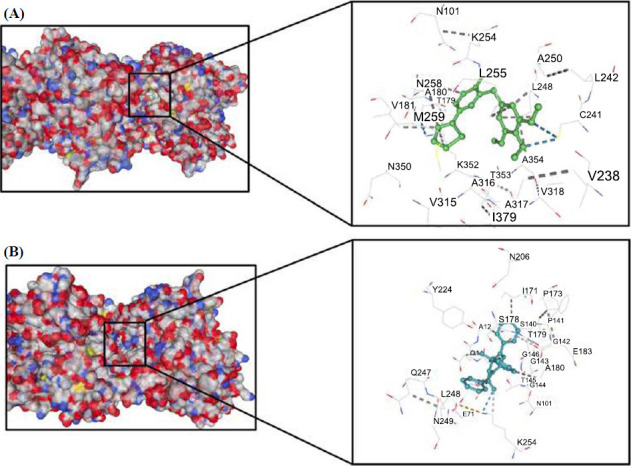
Proposed binding modes of compound **16b** (**A** green) and **29** (**B** blue) in the pocket located between the α-tubulin and β-tubulin interface. Grey dashed lines indicate hydrophobic contact and blue/light blue dashed lines indicate potential hydrogen bonds. Nitrogen atoms are colored blue, oxygen atoms red, and sulfur atoms yellow.

Molecular docking revealed that the best pose for compounds **16b** and **29** is situated between the α- and β-subunit of tubulin dimers with binding energies of -7.9 kcal/mol and -9.6 kcal/mol respectively. As shown in Fig. (**6A**) The compound **16b** mostly occupied a pocket within the β-subunit with the TMP group interacting with multiple amino acid residues (Leu248, Ala250, Leu255) and forming two hydrogen bonds with sulfur atom of Cys241 similar to colchicine that is known tubulin depolymerization agent [57]. This residue has been previously described as an important target for colchicine binding site inhibitors (CBSIs) [58, 59]. Whereas, the pyridine core and thiophene ring of **16b** interacted with amino acid residues located on both subunits (α: Ala180, Val181; β: Asn258, Lys352*).* The pyridine core also formed a weak hydrogen bond with the oxygen of β Asn258 and the pyridine core (light blue dashed line). On the other hand, compound **29** bonded more closely to α-subunit interacting with multiple amino acid residues of this chain (Gln11, Phe141, Ile171, Ala180), but also forming a potential hydrogen bond with β Lys254 nitrogen atom (Fig. **6B**), which is surprising considering the above results of the cell-free tubulin polymerization assay (IC_50_ for 29 >100µM). Nevertheless, the obtained molecular docking results were consistent with our previously published data, indicating that the colchicine-binding site of tubulin may be the molecular target of dihydropyridine-2(1*H*)-thiones [12]. However, a comprehensive analysis of pyridine-containing drugs revealed that most of these compounds primarily affect kinases and enzymes involved in cell cycle progression, proliferation, cellular catabolism, cell survival, and apoptosis [10]. While the possibility of compound **16b** having a complex mechanism of action (impacting both kinases and tubulin, similar to tirbanibulin [60]) cannot be dismissed, additional research is necessary to confirm this hypothesis.

## CONCLUSION

In conclusion, a series of new pyridine and pyrimidine-thione-based derivatives were synthesized and tested for their anticancer properties against melanoma A375 cell lines. Among all screened 33 compounds, the **16b** and **29** belonging to pyridine analogs, showed promising antiproliferative activity, confirming the importance of pyridine-2(1*H*)-thione (PT) and 3,4-dihydropyridine-2(1*H*)-thione (DHPT) rings as a valuable pharmacophore. Further studies revealed that compound **16b**, bearing a thiophene ring at the C-5 and 3,4,5-trimethoxyphenyl (TMP) moiety, displayed the most promising antimitotic and antitubulin activity probably related to the colchicine binding site on tubulin. Computational ADMET analysis confirmed the drug-likeness of the synthesized compounds. Moreover, both compounds exhibited enhanced selectivity towards A375 cancer cells compared to normal fibroblasts, suggesting a possible lower toxicity and wider therapeutic index than those of conventional drugs. Additionally, the mitotic-specific activity of compound **16b** might potentially contribute to decreased side effects compared to classical microtubule- targeted agents.

## Figures and Tables

**Fig. (1):**
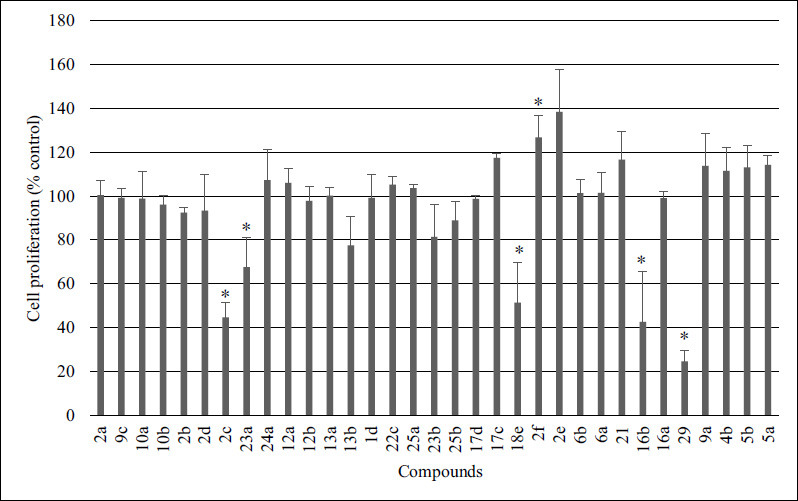
Screening study. The proliferation of melanoma A375 cells after 48 h of treatment with a single concentration (10 µM) of compounds, determined by WST-1 assay. The data are presented as average ± standard deviation, derived from a minimum of three separate experiments; * *p* < 0.05 *vs* ctrl (Student’s *t*-test).

**Fig. (2):**
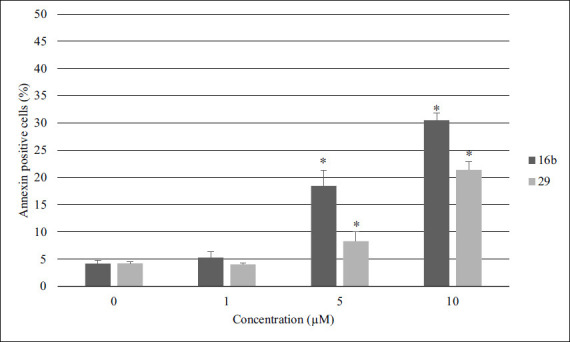
The impact of specific concentrations of compounds **16b** and **29** on triggering programmed cell death in melanoma cell lines was evaluated after a 48-hour exposure period. Following treatment, cells underwent staining with annexin V-PE and 7-AAD before analysis using a Muse Cell Analyzer. Results are presented as the average ± standard deviation, derived from a minimum of three separate experiments, *p < 0.05 *vs* ctrl (Student’s *t*-test).

**Fig. (3):**
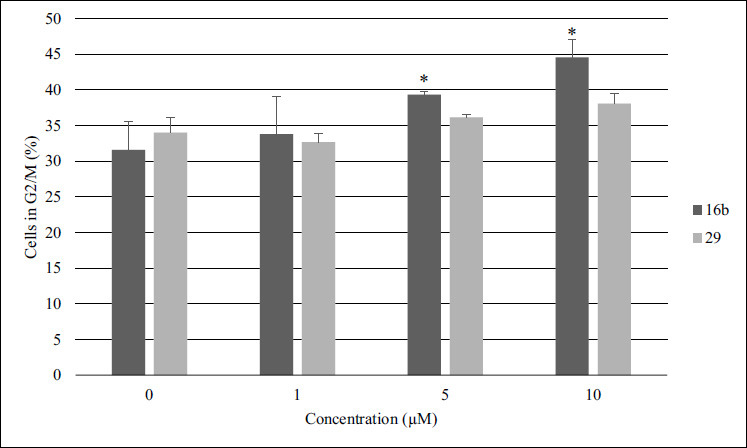
A375 cells exposed to various concentrations of compounds **16b** and **29** for 5 hours. The cells were then stained with PI and examined using a Muse Cell Analyzer to determine the proportion in the G2/M phase. Results are presented as the average and standard deviation derived from a minimum of three separate experiments; *p < 0.05 *vs* ctrl (Student’s *t*-test).

**Fig. (5):**
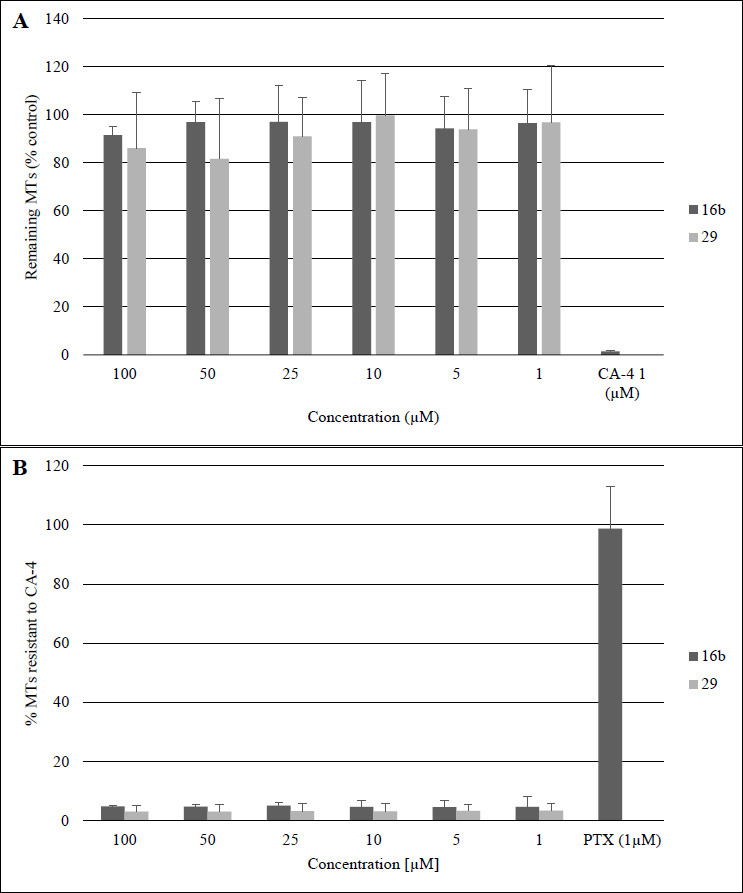
The impact of compounds **16b** and **29** on microtubule stability in melanoma A375 cells was evaluated under two conditions. (**A**) First, the destabilizing effect was assessed after a 30-minute exposure to various concentrations of these compounds. The outcome is presented as the percentage of remaining microtubules (MTs), with cells treated using 0.2% DMSO serving as the 100% baseline. (**B**) Second, the stabilizing influence was examined following a 90-minute treatment with the same compounds at specified concentrations. In this case, results are shown as the percentage of MTs resistant to CA-4-induced depolymerization. The 100% mark corresponds to cells treated with 0.2% DMSO without CA-4, while 0% represents cells exposed to both DMSO and CA-4. The findings are displayed as mean values with standard deviations, derived from a minimum of three separate experiments; * *p* < 0.05 *vs* cells treated with 0.2% DMSO (for destabilization assay) and * *p* < 0.05 *vs* cells treated with DMSO (0.2%) and CA-4 (for stabilization assay) determined by Student’s *t*-test.

**Table 1:** The IC_50_ values (µM) and selectivity index (SI) for chosen compounds, as determined by the WST-1 assay following 48 hours of treatment.

**Compounds**	**A375**	**HDF ^1^**	**SI ^2^**
2c	59.30 ± 9.62	nd ^3^	nd ^3^
18e	10.13 ± 0.75	nd ^3^	nd ^3^
16b	1.85 ± 0.44	120.40 ± 8.42	65.08
29	4.85 ± 1.67	> 500	> 100

**Note:** ^1^ HDF – normal human dermal fibroblasts

^2^Selectivity index: IC_50_ normal cells/IC_50_ cancer cell

^3^Not determined.

## Data Availability

The data and supportive information is available within the article.

## LIST OF ABBREVIATIONS

PS: Phosphatidylserine
PBS: Phosphate-buffered Saline
ECACC: European Collection of Authenticated Cell Cultures
HDF: Human Dermal Fibroblasts
TMP: 3,4,5-trimethoxyphenyl
DALY: Disability-adjusted Life Years
