# Supplementary material for: Synthesis and Antiproliferative Activity against Melanoma Cells of New Heterocyclic Hybrids Based on Pyridine and Pyrimidine Scaffolds
Source: Curr Med Chem. 2025 Jun 10;33(5):983–1003. doi: 10.2174/0109298673370617250330151330 (PMC13223435; doi:10.2174/0109298673370617250330151330)
Supplement: Supplementary file 1 [file CMC-33-5-983_SD1.pdf]

Supplementary Material

Synthesis and Antiproliferative Activity against Melanoma Cells of New Heterocyclic Hybrids Based on Pyridine and Pyrimidine Scaffolds

Magdalena Perużyńska[1,*], Radosław Birger[1], Tomasz J. Idzik[2], Zofia M. Myk[2], Magdalena M. Lubowicz[2], Łukasz Struk[2,3], Jacek G. Sośnicki[2], Patrycja Kłos[4], Dariusz Chlubek[4] and Marek Droździk[1]

[1]Department of Experimental and Clinical Pharmacology, Pomeranian Medical University in Szczecin, Powstanców Wielkopolskich 72, 70-111, Szczecin, Poland; [2]Department of Organic and Physical Chemistry, Faculty of Chemical Technology and Engineering, West Pomeranian University of Technology, Piastów 42, 71-065, Szczecin, Poland; [3]Center for Advanced Materials and Manufacturing Process Engineering (CAMMPE), Piastów 42, 71-065, Szczecin, Poland; [4]Department of Biochemistry and Medical Chemistry, Pomeranian Medical University in Szczecin, Powstanców Wielkopolskich 72, 70-111, Szczecin, Poland

Supplementary materials 1.

Table S1. Screening results of antiproliferative activities of compounds at 10 µM against melanoma A375 cells, determined using WST-1 assay after 48 h of treatment. Bolded compounds which satisfactory pre-determined threshold inhibition criteria (decreased cell proliferation to at least 50%) were progressed to the further study. Data are expressed as the mean ± SD from three independent experiments.

Compound	Cell proliferation (% control)	Compound	Cell proliferation (% control)	Compound	Cell proliferation (% control)
2a	100.45 ± 6.85	13a	100.14 ± 3.65	2e	138.34 ± 19.49
9c	99.2 ± 4.4	13b	77.48 ± 13.05	6b	101.32 ± 6.05
10a	98.82 ± 12.55	1d	99.23 ± 10.77	6a	101.42 ± 9.41
10b	96.09 ± 4.11	22c	105.16 ± 3.72	21	116.54 ± 13.01
2b	92.43 ± 2.51	25a	103.66 ± 1.5	**16b**	**42.75 ± 22.88**
2d	93.28 ± 16.57	23b	81.39 ± 14.88	16a	99.1 ± 3.1
**2c**	**44.76 ± 6.68**	25b	88.89 ± 8.81	**29**	**24.73 ± 4.73**
23a	67.63 ± 13.31	17d	98.71 ± 1.41	9a	113.77 ± 14.49
24a	107.24 ± 14.17	17c	117.33 ± 2.07	4b	111.43 ± 10.80
12a	105.92 ± 6.52	**18e**	**51.43 ± 18.48**	5b	113.11 ± 9.77
12b	97.83±6.41	2f	126.73±9.99	5a	114.19±4.12

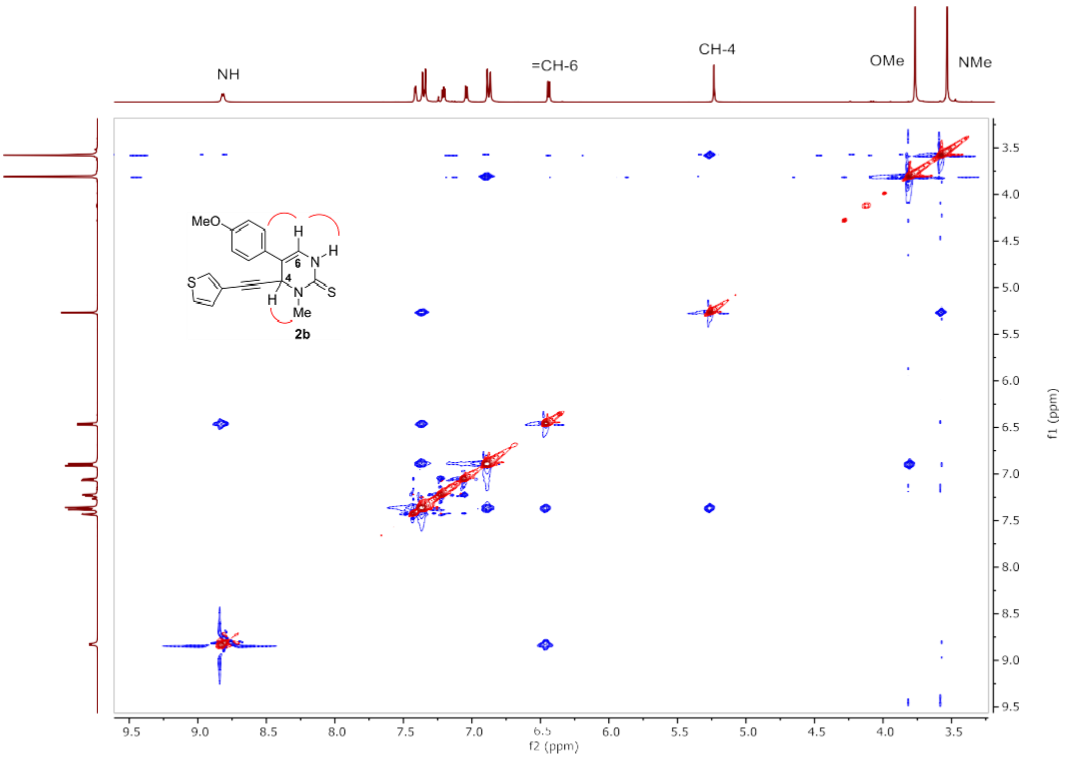

Figure S1. ^{1}H,^{1}H NOESY of compound **2b**

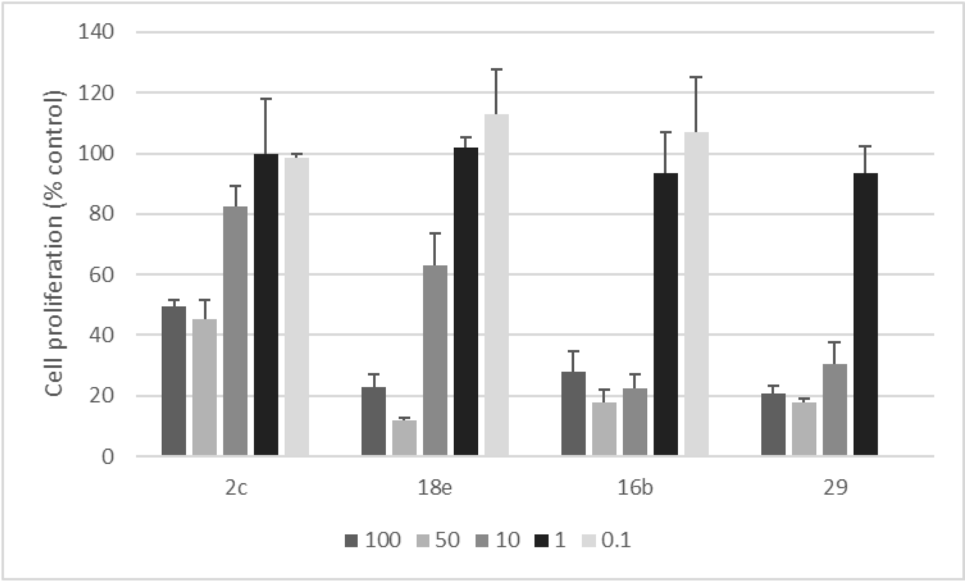

Figure S2. Proliferation of melanoma A375 cells after 48 h of treatment with selected compounds, determined by WST-1 assay. The data are presented as mean and SD from three independent experiments.

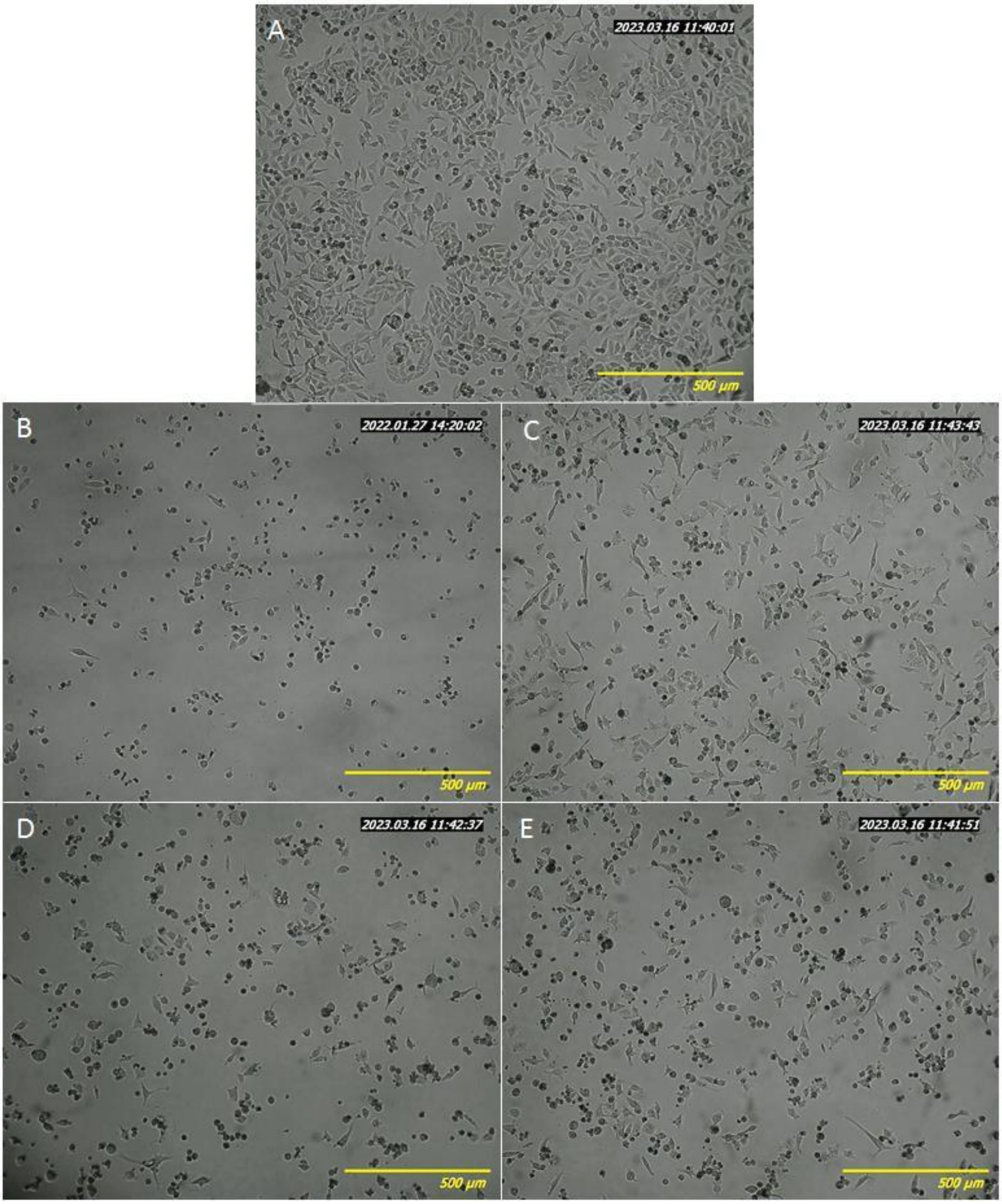

Figure S3. Optical microscopy images of melanoma A375 cells after 48 h incubation with 0.2% DMSO (A) and 10 µM of **2c** (B), **18e** (C), **16b** (D), **29** (E).

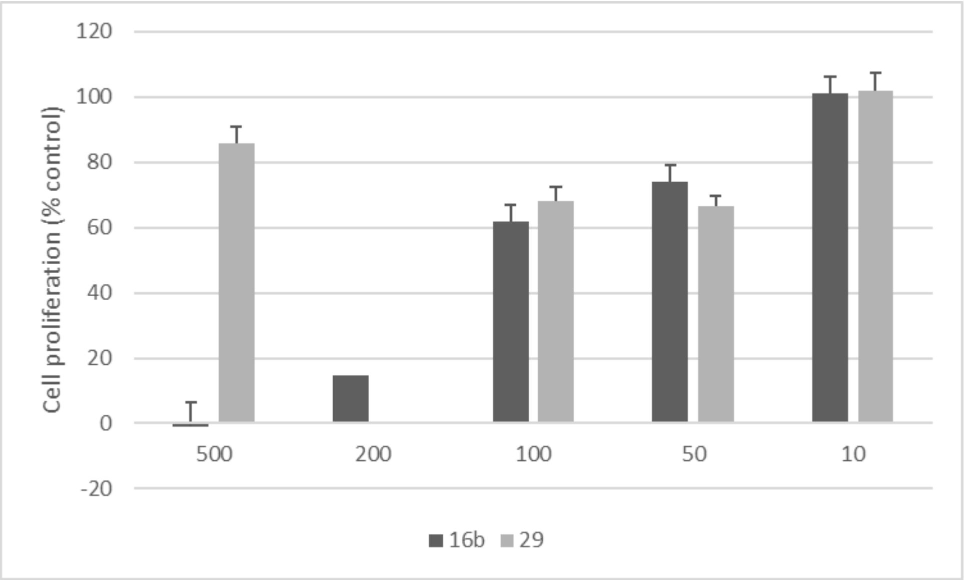

Figure S4. Proliferation of normal HDF cells after 48 h of treatment with selected compounds, determined by WST-1 assay (determined in order to estimate the selectivity index). The data are presented as mean and SD from three independent experiments.

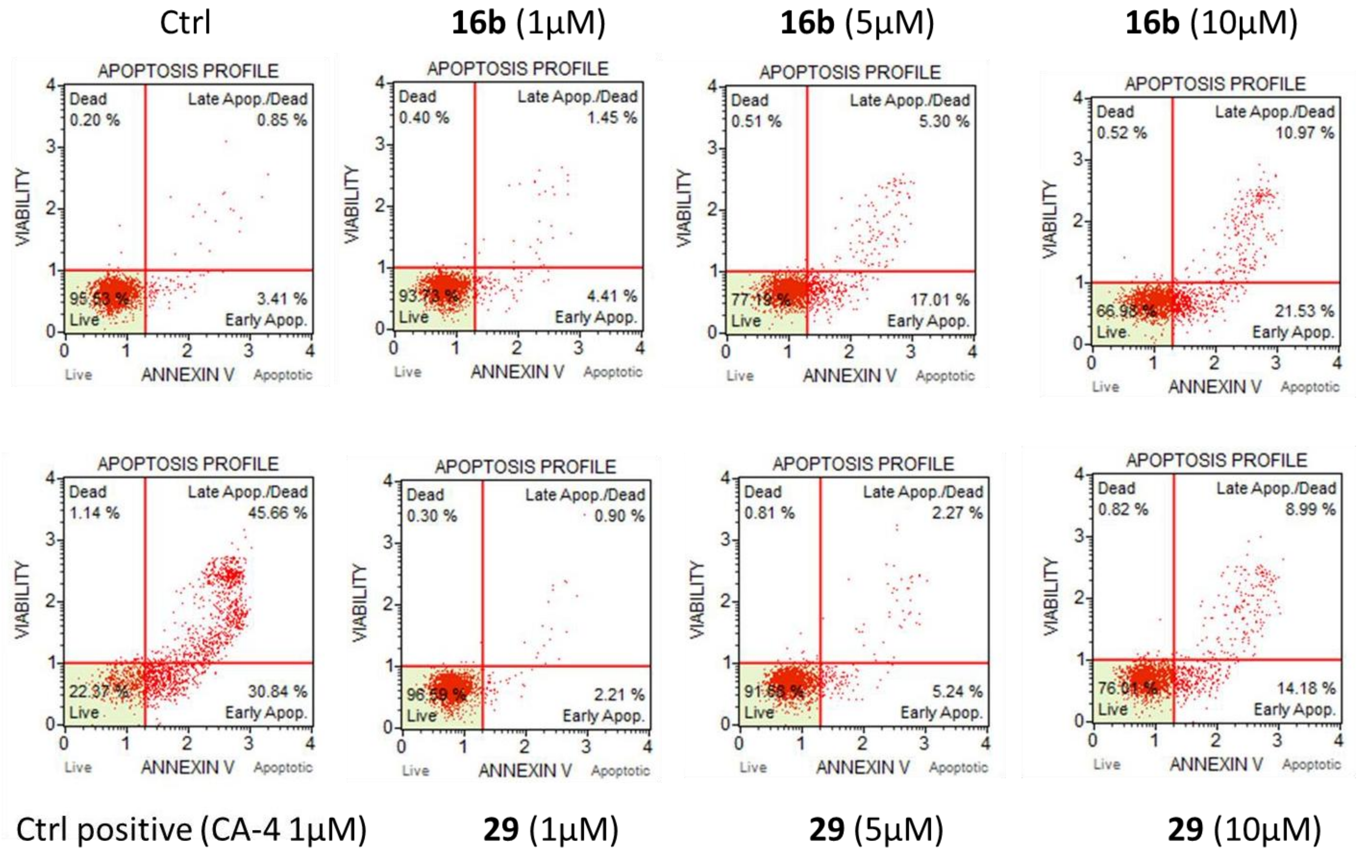

Figure S5. Representative scatter diagrams of melanoma cells exposed to indicated concentrations of **16b** and **29** for 48 h, subsequently stained with annexin V-PE and 7-AAD and analyzed by Muse Cell Analyzer.

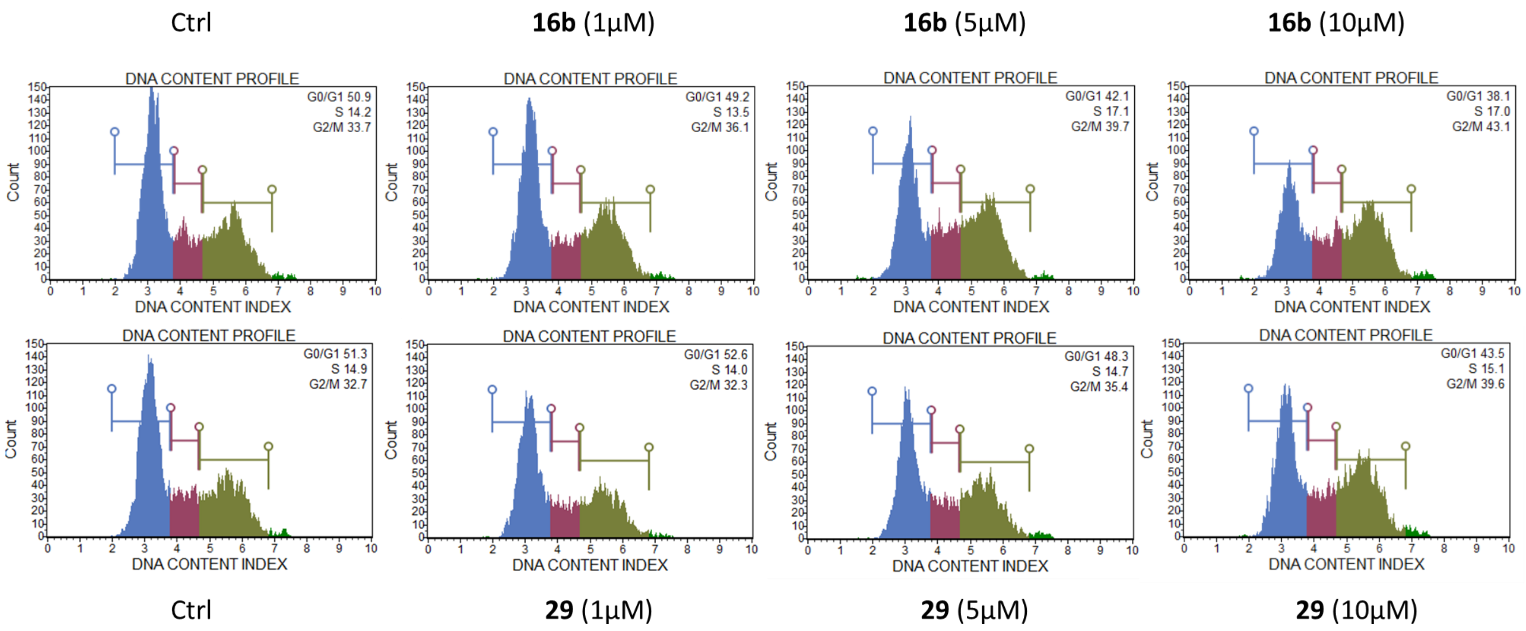

Figure S6. Representative individual experiment of cell cycle phase distribution of A375 cells exposed to indicated concentrations of compounds **16b** and **29** for 5 h subsequently stained with PI analyzed by Muse Cell Analyzer.

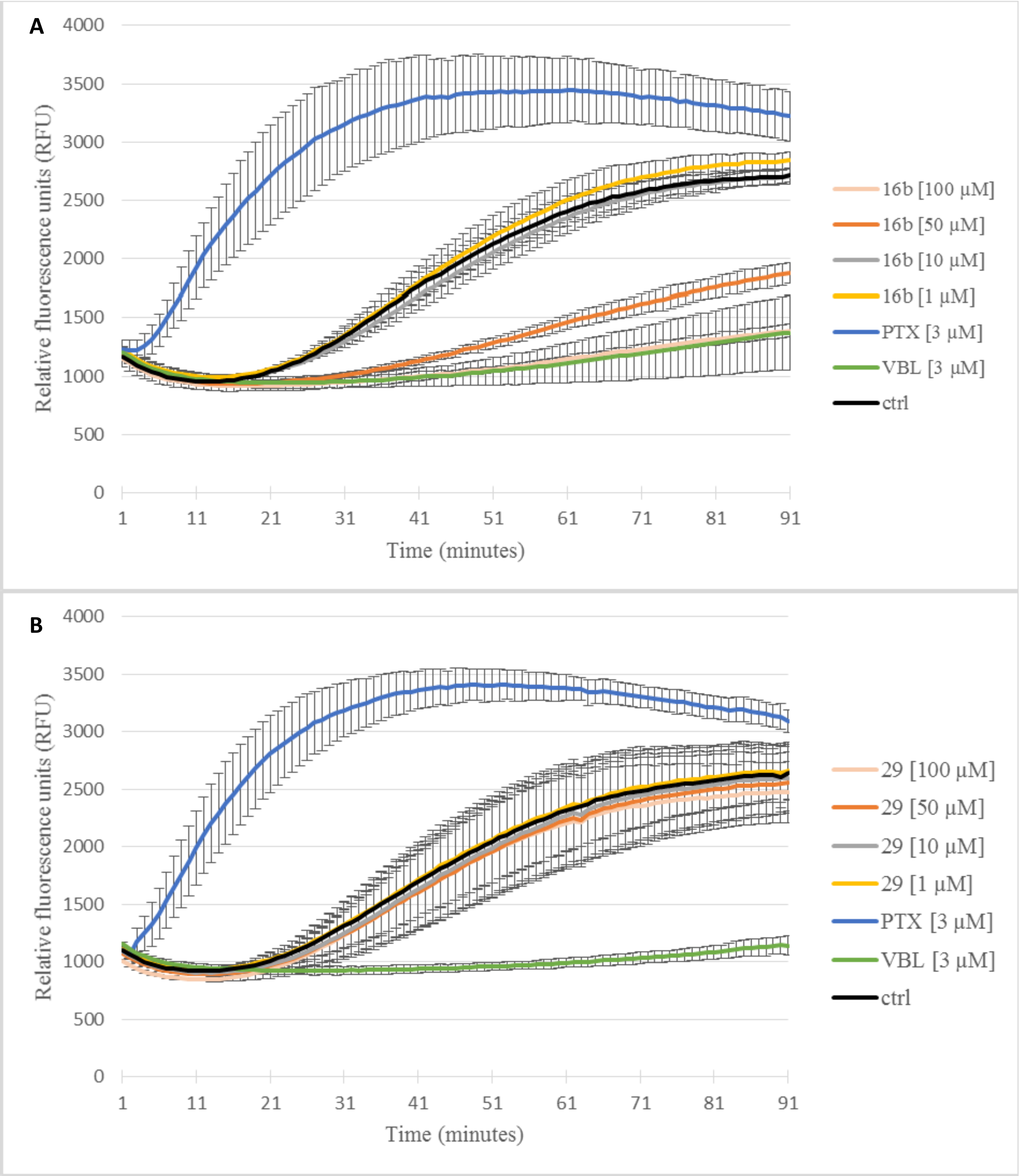

Figure S7. Effects of **16b** (A) and **29** (B) derivatives on tubulin polymerization in cell-free conditions. Increased relative fluorescence units indicate tubulin polymerization. Paclitaxel (PTX) and vinblastine (VBL) were used as references, while DMSO (0.2%) served as a vehicle control. The chart contains error bars representing the variability in the data from three independent experiments.

Table S2. Detailed ADMET properties of compound **16b** generated through online web server ADMETlab 2.0. The empirical-based decision states of each property are visually represented with different colored dots. Decision: green – excellent, yellow – medium, red – poor.

ADMETlab 2.0

COc1cc(cc(c1OC)OC)Cn1cc(ccc1=S)c1cccs1

1. Physicochemical Property

Property	Value	Comment
Molecular Weight	373.08	Contain hydrogen atoms. Optimal:100~600
Volume	364.805	Van der Waals volume
Density	1.023	Density = MW / Volume
nHA	4	Number of hydrogen bond acceptors. Optimal:0~12
nHD	0	Number of hydrogen bond donors. Optimal:0~7
nRot	6	Number of rotatable bonds. Optimal:0~11
nRing	3	Number of rings. Optimal:0~6
MaxRing	6	Number of atoms in the biggest ring. Optimal:0~18
nHet	6	Number of heteroatoms. Optimal:1~15
fChar	0	Formal charge. Optimal:-4 ~4
nRig	18	Number of rigid bonds. Optimal:0~30
Flexibility	0.333	Flexibility = nRot /nRig
Stereo Centers	0	Optimal: ≤ 2
TPSA	32.62	Topological Polar Surface Area. Optimal:0~140
logS	-5.439	Log of the aqueous solubility. Optimal: -4~0.5 log mol/L
logP	3.478	Log of the octanol/water partition coefficient. Optimal: 0~3
logD	3.662	logP at physiological pH 7.4. Optimal: 1~3

2. Medicinal Chemistry

Property	Value	Decision	Comment
QED	0.568	● (red)	■ A measure of drug-likeness based on the concept of desirability;<br>■ Attractive: > 0.67; unattractive: 0.49~0.67; too complex: < 0.34
SAscore	2.465	● (green)	■ Synthetic accessibility score is designed to estimate ease of synthesis of drug-like molecules.<br>■ SAscore ≥ 6, difficult to synthesize; SAscore <6, easy to synthesize

Fsp3	0.211	●	■ The number of sp3 hybridized carbons / total carbon count, correlating with melting point and solubility.<br>■ Fsp^3 ≥0.42 is considered a suitable value.
MCE-18	17.0	●	■ MCE-18 stands for medicinal chemistry evolution.<br>■ MCE-18≥45 is considered a suitable value.
NPscore	-0.905	-	■ Natural product-likeness score.<br>■ This score is typically in the range from –5 to 5. The higher the score is, the higher the probability is that the molecule is a NP.
Lipinski Rule	Accepted	●	■ MW ≤ 500; logP ≤ 5; Hacc ≤ 10; Hdon ≤ 5<br>■ If two properties are out of range, a poor absorption or permeability is possible, one is acceptable.
Pfizer Rule	Rejected	●	logP > 3; TPSA < 75<br>Compounds with a high log P (>3) and low TPSA (<75) are likely to be toxic.
GSK Rule	Accepted	●	■ MW ≤ 400; logP ≤ 4<br>■ Compounds satisfying the GSK rule may have a more favorable ADMET profile
Golden Triangle	Accepted	●	■ 200 ≤ MW ≤ 50; -2 ≤ logD ≤ 5<br>■ Compounds satisfying the Golden Triangle rule may have a more favorable ADMET profile.
PAINS	0 alerts	-	Pan Assay Interference Compounds, frequent hitters, Alpha-screen artifacts and reactive compound.
ALARM NMR	3 alerts	-	Thiol reactive compounds.
BMS	0 alerts	-	Undesirable, reactive compounds.
Chelator Rule	0 alerts	-	Chelating compounds.

3. Absorption

Property	Value	Decision	Comment
Caco-2 Permeability	-4.502	●	Optimal: higher than -5.15 Log unit
MDCK Permeability	1.7e-05	●	■ low permeability: < 2 × 10^{-6} cm/s<br>■ medium permeability: 2–20 × 10^{-6} cm/s<br>■ high passive permeability: > 20 × 10^{-6} cm/s
Pgp-inhibitor	0.983	●	■ Category 1: Inhibitor; Category 0: Non-inhibitor;<br>■ The output value is the probability of being Pgp-inhibitor
Pgp-substrate	0.0	●	■ Category 1: substrate; Category 0: Non-substrate;<br>■ The output value is the probability of being Pgp-substrate
HIA	0.005	●	■ Human Intestinal Absorption<br>■ Category 1: HIA+(HIA < 30%); Category 0: HIA-(HIA < 30%); The output value is the probability of being HIA+

$F_{20\%}$	0.004	●	■ 20% Bioavailability<br>■ Category 1: $F_{20\%}$+ (bioavailability < 20%); Category 0: $F_{20\%}$- (bioavailability ≥ 20%); The output value is the probability of being $F_{20\%}$+
$F_{30\%}$	0.163	●	■ 30% Bioavailability<br>■ Category 1: $F_{30\%}$+ (bioavailability < 30%); Category 0: $F_{30\%}$- (bioavailability ≥ 30%); The output value is the probability of being $F_{30\%}$+

4. Distribution

Property	Value	Decision	Comment
PPB	96.45%	●	■ Plasma Protein Binding<br>■ Optimal: < 90%. Drugs with high protein-bound may have a low therapeutic index.
VD	0.856	●	■ Volume Distribution<br>■ Optimal: 0.04-20L/kg
BBB Penetration	0.271	●	■ Blood-Brain Barrier Penetration<br>■ Category 1: BBB+; Category 0: BBB-; The output value is the probability of being BBB+
Fu	1.668%	●	■ The fraction unbound in plasms<br>■ Low: <5%; Middle: 5~20%; High: > 20%

5. Metabolism

Property	Value	Comment
CYP1A2 inhibitor	0.916	■ Category 1: Inhibitor; Category 0: Non-inhibitor;<br>■ The output value is the probability of being inhibitor.
CYP1A2 Substrate	0.953	■ Category 1: Substrate; Category 0: Non-substrate;<br>■ The output value is the probability of being substrate.
CYP2C19 Inhibitor	0.967	■ Category 1: Inhibitor; Category 0: Non-inhibitor;<br>■ The output value is the probability of being inhibitor.
CYP2C19 Substrate	0.606	■ Category 1: Substrate; Category 0: Non-substrate;<br>■ The output value is the probability of being substrate.
CYP2C9 inhibitor	0.927	■ Category 1: Inhibitor; Category 0: Non-inhibitor;<br>■ The output value is the probability of being inhibitor.
CYP2C9 Substrate	0.854	■ Category 1: Substrate; Category 0: Non-substrate;<br>■ The output value is the probability of being substrate.
CYP2D6 inhibitor	0.31	■ Category 1: Inhibitor; Category 0: Non-inhibitor;<br>■ The output value is the probability of being inhibitor.
CYP2D6 Substrate	0.913	■ Category 1: Substrate; Category 0: Non-substrate;<br>■ The output value is the probability of being substrate.
CYP3A4 inhibitor	0.934	■ Category 1: Inhibitor; Category 0: Non-inhibitor;<br>■ The output value is the probability of being inhibitor.
CYP3A4 Substrate	0.877	■ Category 1: Substrate; Category 0: Non-substrate;<br>■ The output value is the probability of being substrate.

6. Excretion

Property	Value	Decision	Comment
CL	8.691	●	■ Clearance<br>■ High: >15 mL/min/kg; moderate: 5-15 mL/min/kg; low: <5 mL/min/kg
$T_{1/2}$	0.235	-	■ Category 1: long half-life ; Category 0: short half-life;<br>■ long half-life: >3h; short half-life: <3h<br>■ The output value is the probability of having long half-life.

7. Toxicity

Property	Value	Decision	Comment
hERG Blockers	0.194	●	■ Category 1: active; Category 0: inactive;<br>■ The output value is the probability of being active.
H-HT	0.077	●	■ Human Hepatotoxicity<br>■ Category 1: H-HT positive(+); Category 0: H-HT negative(-);<br>■ The output value is the probability of being toxic.
DILI	0.759	●	■ Drug Induced Liver Injury.<br>■ Category 1: drugs with a high risk of DILI; Category 0: drugs with no risk of DILI. The output value is the probability of being toxic.
AMES Toxicity	0.362	●	■ Category 1: Ames positive(+); Category 0: Ames negative(-);<br>■ The output value is the probability of being toxic.
Rat Oral Acute Toxicity	0.027	●	■ Category 0: low-toxicity; Category 1: high-toxicity;<br>■ The output value is the probability of being highly toxic.
FDAMDD	0.737	●	■ Maximum Recommended Daily Dose<br>■ Category 1: FDAMDD (+); Category 0: FDAMDD (-)<br>■ The output value is the probability of being positive.
Skin Sensiti zation	0.315	●	■ Category 1: Sensitizer; Category 0: Non-sensitizer;<br>■ The output value is the probability of being sensitizer.
Carcinogen city	0.093	●	■ Category 1: carcinogens; Category 0: non-carcinogens;<br>■ The output value is the probability of being toxic.
Eye Corrosion	0.003	●	■ Category 1: corrosives ; Category 0: noncorrosives<br>■ The output value is the probability of being corrosives.
Eye Irritation	0.016	●	■ Category 1: irritants ; Category 0: nonirritants<br>■ The output value is the probability of being irritants.

Respiratory Toxicity	0.355	●	■ Category 1: respiratory toxicants; Category 0: respiratory nontoxicants<br>■ The output value is the probability of being toxic.

8. Environmental toxicity

Property	Value	Comment
Bioconcentration Factors	2.867	■ Bioconcentration factors are used for considering secondary poisoning potential and assessing risks to human health via the food chain.<br>■ The unit is –log10[(mg/L)/(1000*MW)]
IGC_{50}	4.519	■ Tetrahymena pyriformis 50 percent growth inhibition concentration<br>■ The unit is –log10[(mg/L)/(1000*MW)]
$LC_{50}FM$	6.315	■ 96-hour fathead minnow 50 percent lethal concentration<br>■ The unit is –log10[(mg/L)/(1000*MW)]
$LC_{50}DM$	6.2	■ 48-hour daphnia magna 50 percent lethal concentration<br>■ The unit is –log10[(mg/L)/(1000*MW)]

9. Tox21 pathway

Property	Value	Decision	Comment
NR-AR	0.132	●	■ Androgen receptor<br>■ Category 1: actives ; Category 0: inactives;<br>■ The output value is the probability of being active.
NR-AR-LBD	0.162	●	■ Androgen receptor ligand-binding domain<br>■ Category 1: actives ; Category 0: inactives;<br>■ The output value is the probability of being active.
NR-AhR	0.733	●	■ Aryl hydrocarbon receptor<br>■ Category 1: actives ; Category 0: inactives;<br>■ The output value is the probability of being active.
NR-Aromatase	0.838	●	■ Category 1: actives ; Category 0: inactives;<br>■ The output value is the probability of being active.
NR-ER	0.523	●	■ Estrogen receptor<br>■ Category 1: actives ; Category 0: inactives;<br>■ The output value is the probability of being active.
NR-ER-LBD	0.047	●	■ Estrogen receptor ligand-binding domain<br>■ Category 1: actives ; Category 0: inactives;<br>■ The output value is the probability of being active.
NR-PPAR-Gamma	0.937	●	■ Peroxisome proliferator-activated receptor gamma<br>■ Category 1: actives ; Category 0: inactives;<br>■ The output value is the probability of being active.
SR-ARE	0.896	●	■ Antioxidant response element<br>■ Category 1: actives ; Category 0: inactives;<br>■ The output value is the probability of being active.
SR-ATAD5	0.825	●	■ ATPase family AAA domain-containing protein 5<br>■ Category 1: actives ; Category 0: inactives;<br>■ The output value is the probability of being active.

SR-HSE	0.808		■ Heat shock factor response element<br>■ Category 1: actives ; Category 0: inactives;<br>■ The output value is the probability of being active.
SR-MMP	0.51		■ Mitochondrial membrane potential<br>■ Category 1: actives ; Category 0: inactives;<br>■ The output value is the probability of being active.
SR-p53	0.741		■ Category 1: actives ; Category 0: inactives;<br>■ The output value is the probability of being active.

10. Toxicophore Rules

Property	Value	Comment
Acute Toxicity Rule	0 alerts	■ 20 substructures<br>■ acute toxicity during oral administration
Genotoxic Carcinogenicity Rule	0 alerts	■ 117 substructures<br>■ carcinogenicity or mutagenicity
NonGenotoxic Carcinogenicity Rule	0 alerts	■ 23 substructures<br>■ carcinogenicity through nongenotoxic mechanisms
Skin Sensitization Rule	2 alerts	■ 155 substructures<br>■ skin irritation
Aquatic Toxicity Rule	0 alerts	■ 99 substructures<br>■ toxicity to liquid(water)
NonBiodegradable Rule	1 alerts	■ 19 substructures<br>■ non-biodegradable
SureChEMBL Rule	0 alerts	■ 164 substructures<br>■ MedChem unfriendly status

Table S3. Detailed ADMET properties of compound **29** generated through online web server ADMETlab 2.0. The empirical-based decision states of each property are visually represented with different colored dots. Decision: green – excellent, yellow – medium, red – poor.

ADMETlab 2.0

CN1C=C(c2ccccc2)[C@@H](C(C1=S)C(=O)c1ccccc1)c1ccccc1

1. Physicochemical Property

Property	Value	Comment
Molecular Weight	383.13	Contain hydrogen atoms. Optimal:100~600
Volume	413.389	Van der Waals volume
Density	0.927	Density = MW / Volume
nHA	2	Number of hydrogen bond acceptors. Optimal:0~12
nHD	0	Number of hydrogen bond donors. Optimal:0~7
nRot	4	Number of rotatable bonds. Optimal:0~11
nRing	4	Number of rings. Optimal:0~6
MaxRing	6	Number of atoms in the biggest ring. Optimal:0~18
nHet	3	Number of heteroatoms. Optimal:1~15
fChar	0	Formal charge. Optimal:-4 ~4
nRig	26	Number of rigid bonds. Optimal:0~30
Flexibility	0.154	Flexibility = nRot /nRig
Stereo Centers	2	Optimal: ≤ 2
TPSA	20.31	Topological Polar Surface Area. Optimal:0~140
logS	-5.166	Log of the aqueous solubility. Optimal: -4~0.5 log mol/L
logP	4.875	Log of the octanol/water partition coefficient. Optimal: 0~3
logD	4.319	logP at physiological pH 7.4. Optimal: 1~3

2. Medicinal Chemistry

Property	Value	Decision	Comment
QED	0.433	●	■ A measure of drug-likeness based on the concept of desirability; ■ Attractive: > 0.67; unattractive: 0.49~0.67; too complex: < 0.34
SAscore	3.227	●	■ Synthetic accessibility score is designed to estimate ease of synthesis of drug-like molecules. ■ SAscore ≥ 6, difficult to synthesize; SAscore <6, easy to synthesize

Fsp3	0.12	🔴	■ The number of sp3 hybridized carbons / total carbon count, correlating with melting point and solubility.<br>■ Fsp^3 ≥0.42 is considered a suitable value.
MCE-18	66.0	🟢	■ MCE-18 stands for medicinal chemistry evolution.<br>■ MCE-18≥45 is considered a suitable value.
NPscore	-0.247	-	■ Natural product-likeness score.<br>■ This score is typically in the range from –5 to 5. The higher the score is, the higher the probability is that the molecule is a NP.
Lipinski Rule	Accepted	🟢	■ MW ≤ 500; logP ≤ 5; Hacc ≤ 10; Hdon ≤ 5<br>■ If two properties are out of range, a poor absorption or permeability is possible, one is acceptable.
Pfizer Rule	Rejected	🔴	logP > 3; TPSA < 75<br>Compounds with a high log P (>3) and low TPSA (<75) are likely to be toxic.
GSK Rule	Rejected	🔴	■ MW ≤ 400; logP ≤ 4<br>■ Compounds satisfying the GSK rule may have a more favorable ADMET profile
Golden Triangle	Accepted	🟢	■ 200 ≤ MW ≤ 50; -2 ≤ logD ≤ 5<br>■ Compounds satisfying the Golden Triangle rule may have a more favorable ADMET profile.
PAINS	0 alerts	-	Pan Assay Interference Compounds, frequent hitters, Alpha-screen artifacts and reactive compound.
ALARM NMR	1 alerts	-	Thiol reactive compounds.
BMS	0 alerts	-	Undesirable, reactive compounds.
Chelator Rule	0 alerts	-	Chelating compounds.

3. Absorption

Property	Value	Decision	Comment
Caco-2 Permeability	-4.767	🟢	Optimal: higher than -5.15 Log unit
MDCK Permeability	3.9e-05	🟢	■ low permeability: $< 2 \times 10^{-6}$ cm/s<br>■ medium permeability: $2–20 \times 10^{-6}$ cm/s<br>■ high passive permeability: $> 20 \times 10^{-6}$ cm/s
Pgp-inhibitor	0.989	🔴	■ Category 1: Inhibitor; Category 0: Non-inhibitor;<br>■ The output value is the probability of being Pgp-inhibitor
Pgp-substrate	0.0	🟢	■ Category 1: substrate; Category 0: Non-substrate;<br>■ The output value is the probability of being Pgp-substrate
HIA	0.003	🟢	■ Human Intestinal Absorption<br>■ Category 1: HIA+(HIA < 30%); Category 0: HIA-(HIA < 30%); The output value is the probability of being HIA+

$F_{20\%}$	0.039	🟢	■ 20% Bioavailability<br>■ Category 1: $F_{20\%}+$ (bioavailability < 20%); Category 0: $F_{20\%}-$ (bioavailability ≥ 20%); The output value is the probability of being $F_{20\%}+$
$F_{30\%}$	0.001	🟢	■ 30% Bioavailability<br>■ Category 1: $F_{30\%}+$ (bioavailability < 30%); Category 0: $F_{30\%}-$ (bioavailability ≥ 30%); The output value is the probability of being $F_{30\%}+$

4. Distribution

Property	Value	Decision	Comment
PPB	97.74%	🔴	■ Plasma Protein Binding<br>■ Optimal: < 90%. Drugs with high protein-bound may have a low therapeutic index.
VD	1.317	🟢	■ Volume Distribution<br>■ Optimal: 0.04-20L/kg
BBB Penetration	0.873	🔴	■ Blood-Brain Barrier Penetration<br>■ Category 1: BBB+; Category 0: BBB-; The output value is the probability of being BBB+
Fu	1.969%	🔴	■ The fraction unbound in plasms<br>■ Low: <5%; Middle: 5~20%; High: > 20%

5. Metabolism

Property	Value	Comment
CYP1A2 inhibitor	0.873	■ Category 1: Inhibitor; Category 0: Non-inhibitor;<br>■ The output value is the probability of being inhibitor.
CYP1A2 substrate	0.368	■ Category 1: Substrate; Category 0: Non-substrate;<br>■ The output value is the probability of being substrate.
CYP2C19 inhibitor	0.921	■ Category 1: Inhibitor; Category 0: Non-inhibitor;<br>■ The output value is the probability of being inhibitor.
CYP2C19 substrate	0.314	■ Category 1: Substrate; Category 0: Non-substrate;<br>■ The output value is the probability of being substrate.
CYP2C9 inhibitor	0.936	■ Category 1: Inhibitor; Category 0: Non-inhibitor;<br>■ The output value is the probability of being inhibitor.
CYP2C9 substrate	0.235	■ Category 1: Substrate; Category 0: Non-substrate;<br>■ The output value is the probability of being substrate.
CYP2D6 inhibitor	0.042	■ Category 1: Inhibitor; Category 0: Non-inhibitor;<br>■ The output value is the probability of being inhibitor.
CYP2D6 substrate	0.108	■ Category 1: Substrate; Category 0: Non-substrate;<br>■ The output value is the probability of being substrate.
CYP3A4 inhibitor	0.55	■ Category 1: Inhibitor; Category 0: Non-inhibitor;<br>■ The output value is the probability of being inhibitor.
CYP3A4 substrate	0.514	■ Category 1: Substrate; Category 0: Non-substrate;<br>■ The output value is the probability of being substrate.

6. Excretion

Property	Value	Decision	Comment
CL	6.331	●	■ Clearance<br>■ High: >15 mL/min/kg; moderate: 5-15 mL/min/kg; low: <5 mL/min/kg
$T_{1/2}$	0.114	-	■ Category 1: long half-life ; Category 0: short half-life;<br>■ long half-life: >3h; short half-life: <3h<br>■ The output value is the probability of having long half-life.

7. Toxicity

Property	Value	Decision	Comment
hERG Blockers	0.023	●	■ Category 1: active; Category 0: inactive;<br>■ The output value is the probability of being active.
H-HT	0.057	●	■ Human Hepatotoxicity<br>■ Category 1: H-HT positive(+); Category 0: H-HT negative(-);<br>■ The output value is the probability of being toxic.
DILI	0.973	●	■ Drug Induced Liver Injury.<br>■ Category 1: drugs with a high risk of DILI; Category 0: drugs with no risk of DILI. The output value is the probability of being toxic.
AMES Toxicity	0.573	●	■ Category 1: Ames positive(+); Category 0: Ames negative(-);<br>■ The output value is the probability of being toxic.
Rat Oral Acute Toxicity	0.868	●	■ Category 0: low-toxicity; Category 1: high-toxicity;<br>■ The output value is the probability of being highly toxic.
FDAMDD	0.851	●	■ Maximum Recommended Daily Dose<br>■ Category 1: FDAMDD (+); Category 0: FDAMDD (-)<br>■ The output value is the probability of being positive.
Skin Sensiti zation	0.421	●	■ Category 1: Sensitizer; Category 0: Non-sensitizer;<br>■ The output value is the probability of being sensitizer.
Carcinogen city	0.528	●	■ Category 1: carcinogens; Category 0: non-carcinogens;<br>■ The output value is the probability of being toxic.
Eye Corrosion	0.003	●	■ Category 1: corrosives ; Category 0: noncorrosives<br>■ The output value is the probability of being corrosives.
Eye Irritation	0.014	●	■ Category 1: irritants ; Category 0: nonirritants<br>■ The output value is the probability of being irritants.

Respiratory Toxicity	0.948	●	■ Category 1: respiratory toxicants; Category 0: respiratory nontoxicants<br>■ The output value is the probability of being toxic.

8. Environmental toxicity

Property	Value	Comment
Bioconcentration Factors	2.621	■ Bioconcentration factors are used for considering secondary poisoning potential and assessing risks to human health via the food chain.<br>■ The unit is –log10[(mg/L)/(1000*MW)]
IGC_{50}	5.045	■ Tetrahymena pyriformis 50 percent growth inhibition concentration<br>■ The unit is –log10[(mg/L)/(1000*MW)]
$LC_{50}FM$	6.519	■ 96-hour fathead minnow 50 percent lethal concentration<br>■ The unit is –log10[(mg/L)/(1000*MW)]
$LC_{50}DM$	4.932	■ 48-hour daphnia magna 50 percent lethal concentration<br>■ The unit is –log10[(mg/L)/(1000*MW)]

9. Tox21 pathway

Property	Value	Decision	Comment
NR-AR	0.074	●	■ Androgen receptor<br>■ Category 1: actives ; Category 0: inactives;<br>■ The output value is the probability of being active.
NR-AR-LBD	0.805	●	■ Androgen receptor ligand-binding domain<br>■ Category 1: actives ; Category 0: inactives;<br>■ The output value is the probability of being active.
NR-AhR	0.978	●	■ Aryl hydrocarbon receptor<br>■ Category 1: actives ; Category 0: inactives;<br>■ The output value is the probability of being active.
NR-Aromatase	0.931	●	■ Category 1: actives ; Category 0: inactives;<br>■ The output value is the probability of being active.
NR-ER	0.912	●	■ Estrogen receptor<br>■ Category 1: actives ; Category 0: inactives;<br>■ The output value is the probability of being active.
NR-ER-LBD	0.672	●	■ Estrogen receptor ligand-binding domain<br>■ Category 1: actives ; Category 0: inactives;<br>■ The output value is the probability of being active.
NR-PPAR-gamma	0.97	●	■ Peroxisome proliferator-activated receptor gamma<br>■ Category 1: actives ; Category 0: inactives;<br>■ The output value is the probability of being active.
SR-ARE	0.939	●	■ Antioxidant response element<br>■ Category 1: actives ; Category 0: inactives;<br>■ The output value is the probability of being active.
SR-ATAD5	0.878	●	■ ATPase family AAA domain-containing protein 5<br>■ Category 1: actives ; Category 0: inactives;<br>■ The output value is the probability of being active.

SR-HSE	0.953	●	■ Heat shock factor response element<br>■ Category 1: actives ; Category 0: inactives;<br>■ The output value is the probability of being active.
SR-MMP	0.977	●	■ Mitochondrial membrane potential<br>■ Category 1: actives ; Category 0: inactives;<br>■ The output value is the probability of being active.
SR-p53	0.86	●	■ Category 1: actives ; Category 0: inactives;<br>■ The output value is the probability of being active.

10. Toxicophore Rules

Property	Value	Comment
Acute Toxicity Rule	0 alerts	■ 20 substructures<br>■ acute toxicity during oral administration
Genotoxic Carcinogenicity Rule	0 alerts	■ 117 substructures<br>■ carcinogenicity or mutagenicity
NonGenotoxic Carcinogenicity Rule	1 alerts	■ 23 substructures<br>■ carcinogenicity through nongenotoxic mechanisms
Skin Sensitization Rule	1 alerts	■ 155 substructures<br>■ skin irritation
Aquatic Toxicity Rule	0 alerts	■ 99 substructures<br>■ toxicity to liquid(water)
NonBiodegradable Rule	0 alerts	■ 19 substructures<br>■ non-biodegradable
SureChEMBL Rule	1 alerts	■ 164 substructures<br>■ MedChem unfriendly status

Chemical part

Melting points were determined on a Boetius hot stage apparatus. ^{1}H and ^{13}C NMR spectroscopic measurements were performed on a Bruker DPX 400 Avance III HD spectrometer, operating at 400.2 and 100.6 MHz, respectively. TMS was used internal standard, $\delta_{H,C}$ = 0 ppm). For NMR analyses MestReNova (version: 12.0.4) program was used. For detailed peak assignments, 2D spectra were acquired using Bruker software (^{1}H,^{1}H DFQCOSY; ^{13}C,^{1}H COSY; ^{1}H,^{13}C HMBC, ^{1}H,^{1}H NOESY). The standard abbreviation for multiplicities were used (s = singlet, d =doublet, t = triplet, q =quartet, quint = quintet, m = multiplet, sxt = sextet, spt = septet, etc. Gas chromatography-mass spectrometry (GC-MS) measurements were carried out on a Hewlett-Packard instrument model HP 6890 equipped with a mass detector HP 5973 and on an Agilent 7820A GC system equipped with a mass (Agilent 5977E MSD) detector. HRMS analyses (ESI+) were performed on a Agilent 6546 LC/Q-TOF using acetonitrile or mixture of acetonitrile/methanol as solvent. The measurement of specific rotation $[\alpha]_D^{20}$ was performed on A.KRÜSS Polarimeter P8000-P from A.KRÜSS Optronic

GmbH at 20 ±0.1 °C and in 589 nm wavelength. Enantiomeric excess was established by HPLC Waters 996 system equipped with chiral column (*R*, *R*) WHELK-O 1 5/100.

n-BuLi (2.5 M in hexane), *sec*-BuLi (1.4 M in cyclohexane), MeLi (3.1 M in diethoxymethane), *i*-PrMgCl (2.0 M in THF), 2-Thienyllithium (1.0 in THF/Hexanes) was purchased from Sigma-Aldrich. Lawesson Reagent was purchased from Fluorochem. 5-iodo-2-methoxypyridine was purchased from Angene. Aryl acetylenes and (6-methoxypyridin-3-yl)boronic acid were purchased from AmBeed. 1,2-dimethylindole was purchased from Ark Pharm, Inc. BnMgCl (1.4 M in THF), 2-methyloxazole, 2-methylbenzoxazole were purchased from Thermo Scientific.

Pyrimidines **1b** and **1c** were obtained according to the procedure described earlier (Ge and Hartwig, 2012).
Anhydrous toluene and THF were purified by distillation over sodium metal under argon prior to use. Products were purified by flash column chromatography on silica gel (63-200 μm, Merck) using appropriate solvents.

Procedure for the synthesis of compounds **2a-2d** (Scheme 1)
To a solution of arylacetylene (1.4 mmol, 2.0 equiv.) in 10 mL of THF at 0°C *i*-PrMgCl (0.73 mL, 1.47 mmol, 2.0 M in THF, 2.1 equiv.) was added and stirred at this temperature for 1 h. Solution of 1-substituted-5-arylpyrimidine-2(1*H*)-thione (0.7 mmol, 1.0 equiv.) in 10 mL of dry THF, cooled to 0°C, was transferred to the obtained solution of (arylethynyl)magnesium chloride. The reactions were carried out for 1 hour at 0°C (for product **2a**); for 1 hour at 0°C and 17 hours at room temperature (for products **2b** and **2c**), and 1 hour at 0°C, 1 hour at room temperature and 18 hours at 60°C (for product **2d**). After this, 10 mL of a saturated aqueous solution of NH_4Cl was added, the aqueous layer was extracted with ethyl acetate (3 x 50 ml) and the combined organic layers were dried with $MgSO_4$. The mixture was filtered, and the solvents were evaporated under reduced pressure. The crude products were purified by silica gel column chromatography using appropriate solvent mixtures to obtain the desired products. The following products were obtained: **2a** (69% yield, 0.483 mmol, 0.1983 g), **2b** (91% yield, 0.637 mmol, 0.2169 g), **2c** (83% yield, 0.581 mmol, 0.2246 g) and **2d** (68% yield, 0.476 mmol, 0.1597 g).

(4*RS*)-3-Benzyl-5-(4-methoxyphenyl)-4-(phenylethynyl)-3,4-dihydropyrimidine-2(1*H*)-thione (**2a**):

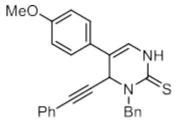

2a 69%

Pale yellow solid, m.p. 218-220°C. ^{1}H NMR (400 MHz, $CDCl_3$) δ 3.77 (s, 3H, OCH_3), 4.68 (d, *J* = 15.1 Hz, 1H, N(3)C$\underline{H}$H), 5.20 (s, 1H, CH-4), 6.28 (d, *J* = 15.1 Hz, 1H, N(3)CH$\underline{H}$), 6.49 (d, *J* = 5.2 Hz, 1H, =CH-6), 6.75 – 6.87 (m, 2H, Ar), 7.23 – 7.40 (m, 10H, ArH), 7.42 – 7.47 (m, 2H, ArH), 8.77 (d, *J* = 5.2 Hz, 1H, N(1)H). ^{13}C NMR (101 MHz, $CDCl_3$) δ 49.03 (CH-4), 54.56 [N(3)CH_2], 55.31 (OCH_3), 84.86, 85.42 (2 x ≡C), 111.92 (=C-5),

114.21 (2H, ArH), 118.57 (=CH-6), 121.89 (Ar), 125.73 (2C, ArH), 126.85 (Ar), 128.02 (3C), 128.27 (2C), 128.82, 128.85 (2C), 131.96 (2C), (ArH), 135.27 (Ar), 159.05 (Ar), 175.56 (C=S). GC-MS (EI, 70eV): Decomposition. HRMS (ESI-TOF) m/z: $[M + H]^+$ Calcd for $C_{26}H_{23}N_2OS$ 411.1531; Found 411.1529.

(4*RS*)-5-(4-Methoxyphenyl)-3-methyl-4-(thiophen-3-ylethynyl)-3,4-dihydropyrimidine-2(1*H*)-thione

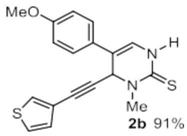
2b 91%

(**2b**): Yellow solid, m.p. 184-186°C. ^{1}H NMR (400 MHz, $CDCl_3$) δ 3.58 [s, 3H, N(3)CH_3], 3.81 (s, 3H, OCH_3), 5.27 (s, 1H, CH-4), 6.46 (d, *J* = 5.2 Hz, 1H, =CH-6), 6.80 – 6.97 (m, 2H, ArH), 7.06 [dd, *J* = 5.0, 1.2 Hz, 1H, (3-Th)], 7.23 [dd, *J* = 5.0, 3.0 Hz, 1H, (3-Th)], 7.31 – 7.40 (m, 2H, ArH), 7.44 [dd, *J* = 3.0, 1.2 Hz, 1H, (3-Th)], 8.62 (d, *J* = 5.3 Hz, 1H, NH). ^{13}C NMR (101 MHz, $CDCl_3$) δ 40.39 [N(3)CH_3], 52.82 (CH-4), 55.36 (OCH_3), 80.87, 84.28 (2 x ≡C), 111.44 (=C-5), 114.28 (2C, ArH), 118.49 (=CH-6), 120.91 (3Th), 125.43 (3ThH), 125.75 (2C, ArH), 127.02 (Ar), 129.88 (3ThH), 129.94 (3ThH), 159.08 (Ar), 174.81 (C=S). GC-MS (EI, 70eV): Decomposition. HRMS (ESI-TOF) m/z: $[M + H]^+$ Calcd for $C_{18}H_{17}N_2OS_2$ 341.0782; Found 341.0779.

(4*RS*)-3-Benzyl-5-phenyl-4-(thiophen-3-ylethynyl)-3,4-dihydropyrimidine-2(1*H*)-thione (**2c**): White

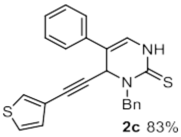
2c 83%

solid, m.p. 184-187°C. ^{1}H NMR (400 MHz, $CDCl_3$) δ 4.69 [d, *J* = 15.2 Hz, 1H, N(3)C<u>H</u>H], 5.22 (s, 1H, CH-4), 6.26 [d, *J* = 15.2 Hz, 1H, N(3)CH<u>H</u>], 6.60 (d, *J* = 5.2 Hz, 1H, =CH-6), 7.05 (dd, *J* = 4.9, 1.2 Hz, 1H, 3Th), 7.17 – 7.46 (m, 12H, 3Th, 2 x C_6H_5), 8.93 [d, *J* = 5.2 Hz, 1H, N(1)H]. ^{13}C NMR (101 MHz, $CDCl_3$) δ 48.88 (CH-4), 54.60 (NCH_2), 80.63, 84.41 (2 x ≡C), 111.92 (=C-5), 119.99 (=CH-6), 120.86 (3Th), 124.33 (2C, ArH), 125.38 (3ThH), 127.37 (ArH), 128.00 (2C, ArH), 128.04 (ArH), 128.77 (2C), 128.85 (2C), (ArH), 129.93 (3ThH), 129.95 (3ThH), 134.22 (Ar), 135.20 (Ar), 175.76 (C=S). GC-MS (EI, 70eV): Decomposition. HRMS (ESI-TOF) m/z: $[M + H]^+$ Calcd for $C_{23}H_{19}N_2S_2$ 387.0990; Found 387.0985.

(4*RS*)-5-(4-Methoxyphenyl)-3-methyl-4-(pyridin-2-ylethynyl)-3,4-dihydropyrimidine-2(1*H*)-thione*

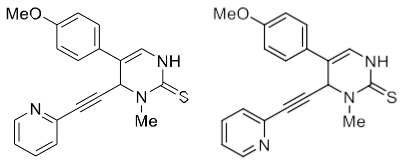
2d
68%

(**2d**): Brown solid, m.p. 151-154°C. ^{1}H NMR (400 MHz, $CDCl_3$) δ 3.60 [s, 3H, $N_{(3)}CH_3$], 3.80 (s, 3H, OCH_3), 5.30 – 5.34 (m, 1H, CH-4), 6.45 – 6.54 (m, 1H, =CH-6), 6.82 – 6.92 (m, 2H, ArH), 7.16 – 7.30 (m, 1H, Py), 7.32 – 7.46 (m, 3H, ArH, Py), 7.62 (qd, *J* = 7.5, 1.9 Hz, 1H, Py), 8.54 – 8.62 (m, 1H, Py), 8.74 [d, *J* = 5.2 Hz, 1H, N(1)H].

^{13}C NMR (101 MHz, $CDCl_3$) δ 40.50, 41.46 [N(3)CH_3], 44.97, 52.63 (CH-4), 55.35 (OCH_3), 84.00, 84.45, 84.62, 86.59 (2 x ≡C), 110.81, 113.76 (=C-5), 114.32 (2C, ArH), 118.89, 124.40 (=CH-6), 123.34, 123.45, (PyH) , 125.71, 125.97 (2C, ArH), 126.67, 126.86 (Ar), 127.65, 127.76 (PyH), 136.18, 136.24 (PyH), 142.17, 142.31 (Py), 150.02, 150.06 (PyH), 159.07, 159.34 (Ar), 175.00, 175.93 (C=S).

GC-MS (EI, 70eV): Decomposition. HRMS (ESI-TOF) m/z: [M + H]+ Calcd for $C_{19}H_{18}N_3OS$ 336.1171; Found 336.1165.

*The presence of two rotameric forms was observed in ^{1}H NMR spectrum

Procedure for the synthesis of compounds **2e-2f** (Scheme 1):
In a 100-mL Schlenk flask equipped with a magnetic stirrer bar and argon balloon, 0.15 g (0.54 mmol) of 5-(3,4,5-trimethoxyphenyl)pyrimidine-2(1H)-thione (**1d**) was dissolved in 50 mL of dry THF. The solution was cooled to 0 °C and 2-thienyllithium (1.0 M in THF/hexane) [2.7 ml, 2.7 mmol] or benzylmagnesium chloride (1.4 M in THF) [1.9 mL, 2.7 mmol] was added. The mixture was stirred at 0°C for 60 minutes. After quenching with aqueous saturated NH_4Cl (15 mL), the water layer was extracted with ethyl acetate (3 × 75 mL) and the combined organic layers were dried over $MgSO_4$. The crude products were purified by flash column chromatography (silica gel, *n*-hexane : ethyl acetate = 6 : 4) yielded 0.168 g (84%) of **2e** and 0.084 g (43%) of **2f** as products.

(4*RS*)-4-Benzyl-5-(3,4,5-trimethoxyphenyl)-3,4-dihydropyrimidine-2(1*H*)-thione (**2e**): Pale yellow solid, m.p. 93-95°C (*n*-hexane/ethyl acetate). ^{1}H NMR (400 MHz, $CDCl_3$) δ 2.86 (dd, *J* = 13.8, 7.3 Hz, 1H, 4-C<u>H</u>H), 2.97 (d, *J* = 13.8 Hz, 1H, 4-CH<u>H</u>), 3.87 (s, 3H, CH_3), 3.90 (s, 6H, 2 x OCH_3), 4.73 (br s, 1H, CH-4), 6.39 (br s, 1H, NH), 6.53 (s, 2H, ArH), 7.13 – 7.38 (m, 5H), 8.68 (br s, 0.7H, NH). ^{13}C NMR (101 MHz, $CDCl_3$) δ 42.04 (4-CH_2), 56.07 (CH-4), 56.36 (2C, 2 x OCH_3), 60.99 (OCH_3), 102.35 (2C, ArH), 119.79 (=CH-6), 127.24, 128.82, 129.77 (ArH), 130.59, 135.67, 138.04, 153.70 (2C), (Ar, =C-5), (C=S signal is not visible). GC-MS (EI, 70eV): m/z = 370 (<1) [$M^{+\cdot}$], 279 (100), 91 (15). HRMS (ESI-TOF) m/z: $[M + H]^+$ Calcd for $C_{20}H_{23}N_2O_3S$ 371.1429; Found 371.1426.

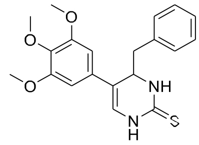

2e 84%

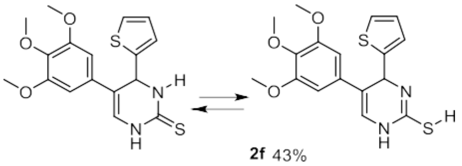

2f 43%

(4*RS*)-4-(Thiophen-2-yl)-5-(3,4,5-trimethoxyphenyl)-3,4-dihydropyrimidine-2(1*H*)-thione (**2f**): Beige solid, m.p. 105-109°C (n-hexane/ethyl acetate) ^{1}H NMR (400 MHz, $CDCl_3$) δ 3.76 (s, 6H, 2x OCH_3), 3.81 (s, 3H, OCH_3), 5.74 (s, 1H, CH-4), 6.44 (s, 2H, ArH), 6.66 (s, 1H, NH), 6.92 (dd, *J* = 5.0, 3.4 Hz, 1H, 2Th), 7.08 (d, *J* = 3.4 Hz, 1H, 2Th), 7.23 (d, *J* = 5.0 Hz, 1H, 2Th), 8.04 (br s, 0.5H,NH) 9.22 (br s, 0.5, SH). ^{13}C NMR (101 MHz, $CDCl_3$) δ 52.88 (CH-4), 56.16 (2C, 2 x OCH_3), 60.92 (OCH_3), 102.69 (2C, ArH), 119.70 (=CH-6), 125.91, 126.76, 127.37 (2ThH), 130.34 (Ar), 137.97 (Ar), 144.91 (2Th), 153.35 (3C, Ar). (Signals for C=S and N-C=N are not visible.) GC-MS (EI, 70eV): m/z = 362 (100) [$M^{+\cdot}$], 302 (16), 272 (12), 207 (23), 136 (11), 96 (10), 73 (7). HRMS (ESI-TOF) m/z: [M + H]+ Calcd for $C_{17}H_{19}N_2O_3S_2$ 363.0837; Found 363.0836.

Procedure for the synthesis of compound **1a** and **1d** (Scheme 1):

In a 100-mL flask equipped with a magnetic stirrer bar, condenser (protected with $CaCl_2$ drying tube), 0.01 mol of 2-aryl-3-(dimethylamino)allylidene(dimethyl)-ammonium perchlorate and 0.015 mol of thiourea (for **1d**) or N-Benzylthiourea (for **1a**) was dissolved in 30 mL of abs. ethanol. Subsequently, MeONa solution (20 mL, 1 M in MeOH) was added, and the reaction mixture was stirred at rt. for 0.5 h and then refluxed for 18 h. After cooling to rt. acetic acid (5 mL) was added. In preparation of **1a**, 50 mL of water was added. Precipitated product was separated by suction, washed with water, dried and crystallized from alcohol, yielding 1.24 g (40%) of **1a** and 2.03 g (73%) of **1b**.

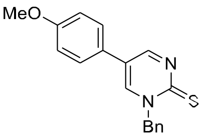

1a 40%

1-Benzyl-5-(4-methoxyphenyl)pyrimidine-2(1*H*)-thione (**1a**): Yellow solid, m.p. 188-190 °C (MeOH). ^{1}H NMR (400 MHz, DMSO) δ 3.81 (s, 3H, OCH_3), 5.76 (s, 2H, NCH_2), 6.99 – 7.11 (m, 2H, ArH), 7.25 – 7.49 (m, 5H, ArH), 7.58 – 7.86 (m, 2H, ArH), 8.95 (dd, *J* = 7.0, 2.6 Hz, 2H). ^{13}C NMR (101 MHz, DMSO) δ 55.80 (OCH_3), 59.78 (NCH_2), 115.15 (2C, ArH), 122.59, 124.74 (Ar), 127.66 (2C), 128.25, 128.38 (2C, ArH), 129.00 (2C, ArH), 135.78 (Ar), 146.90 (ArH), 158.20 (ArH), 160.08 (Ar), 180.28 (C=S). GC-MS (EI, 70eV): m/z = 308 (87) [$M^{+\cdot}$], 275 (100), 91 (31). HRMS (ESI-TOF) m/z: $[M + H]^+$ Calcd for $C_{18}H_{17}N_2OS$ 309.1062; Found 309.1059.

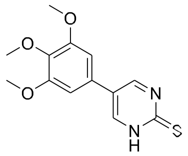

1d 73%

5-(3,4,5-Trimethoxyphenyl)pyrimidine-2(1*H*)-thione (1d): Yellow solid, m.p. 223-227°C (*i*-PrOH). ^{1}H NMR (400 MHz, DMSO) δ 3.67 (s, 3H, OCH_3), 3.85 (s, 6H, 2 x OCH_3), 7.01 (s, 2H, ArH), 8.72 (br. s, 2H. ArH), 14.23 (br. s, 1H, NH). ^{13}C NMR (101 MHz, DMSO) δ 56.62 (2C, 2 x OCH_3), 60.53 (OCH_3), 103.79 (2C, ArH), 122.24, 128.44, 138.03, 153.95 (2C), (Ar). (Some signals are not visible, probably due to the exchange process). GC-MS (EI, 70eV): m/z = decomposition. HRMS (ESI-TOF) m/z: $[M + H]^+$ Calcd for $C_{13}H_{15}N_2O_3S$ 279.0803; Found 279.0799.

Procedure for the synthesis of compounds **4a** and **4b** (Scheme 2):

To a 10-mL flask equipped with a magnetic stir bar and a condenser crowned with argon, charged with a mixture of anhydrous DMF (3 mL) and 5-iodo-2-methoxypyridine (1.0g, 4.26 mmol), degassed for 2 h using a stream of argon slowly bubbled through the solution at rt. During vigorous stirring, appropriate thienylacetylene (5.53 mmol, 1.3 equiv.), $Pd(PPh_3)_2Cl_2$ (0.043 mmol., 30.2 mg), CuI (0.043 mmol., 8 mg) were added. Subsequently, anhydrous Et_3N (2.0 mL) was added over 10 minutes, and the mixture was heated at 60^{0}C for 2 hours. After this time, the reaction mixture was cooled to rt and aqueous saturated NaCl (2 mL) was added, the mixture was extracted with ethyl acetate (3 x 70 mL) and the combined organic layers were washed with brine (20 mL), dried over $MgSO_4$ and filtered through the pad of Celite. Concentration in vacuo and purification by flash column chromatography on

silica gel using a mixture of appropriate solvents give the desired product. The following products were obtained: **4a** (86% yield, 3.66 mmol, 0.789 g) and **4b** (96% yield, 4.09 mmol, 0.880 g).

2-Methoxy-5-(thiophen-2-ylethynyl)pyridine (**4a**): Pale yellow semisolid. ^{1}H NMR (400 MHz, $CDCl_3$)

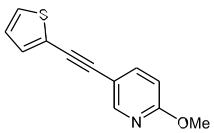

4a 86%

δ 3.96 (s, 3H, OCH_3), 6.73 (dd, *J* = 8.6, 0.8 Hz, 1H, CH-3), 7.01 (dd, *J* = 5.1, 3.7 Hz, 1H, 2Th), 7.21 – 7.34 (m, 2H, 2Th), 7.67 (dd, *J* = 8.6, 2.4 Hz, 1H, CH-4), 8.33 (dd, *J* = 2.4, 0.8 Hz, 1H, CH-6). ^{13}C NMR (101 MHz, $CDCl_3$) δ 53.71 (OCH_3), 83.92, 89.86 (-C≡C-), 110.76 (CH-3), 112.83, 123.08 (Ar, 2Th), 127.12, 127.33 (2ThH), 131.92 (2ThH), 140.98 (CH-4), 149.96 (CH-6), 163.49 (C-2). GC-MS (EI, 70eV): m/z = 215 (100) [$M^{+\cdot}$], 214 (43), 186 (47), 185 (14), 145 (30). HRMS (ESI-TOF) m/z: $[M + H]^+$ Calcd for $C_{12}H_{10}NOS$ 216.0483; Found 216.0478.

2-Methoxy-5-(thiophen-3-ylethynyl)pyridine (**4b**): White semisolid. ^{1}H NMR (400 MHz, $CDCl_3$) δ

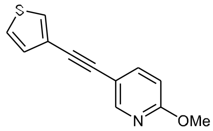

4b 96%

3.96 (s, 3H, OCH_3), 6.73 (dd, *J* = 8.6, 0.8 Hz, 1H, CH-3), 7.19 (dd, *J* = 5.0, 1.2 Hz, 1H, 3Th), 7.31 (dd, *J* = 5.0, 3.0 Hz, 1H, 3Th), 7.52 (dd, *J* = 3.0, 1.2 Hz, 1H, 3Th), 7.67 (dd, *J* = 8.6, 2.3 Hz, 1H, CH-4), 8.33 (dd, *J* = 2.3, 0.8 Hz, 1H, CH-6). ^{13}C NMR (101 MHz, $CDCl_3$) δ 53.68 (OCH_3), 85.69, 85.81 (2 x ≡C), 110.70 (CH-3), 113.07 (C-5), 122.05 (3Th), 125.46, 128.63, 129.77 (3ThH), 141.08 (CH-4), 149.95 (CH-6), 163.34 (C-2). GC-MS (EI, 70eV): m/z = 215 (100) [$M^{+\cdot}$], 214 (54), 186 (46), 185 (17), 145 (28). HRMS (ESI-TOF) m/z: $[M + H]^+$ Calcd for $C_{12}H_{10}NOS$ 216.0483; Found 216.0479.

Procedure for the synthesis of compounds **5a** and **5b** (Scheme 2):

A mixture of **4a** (0.37 g, 1.71 mmol, *reaction a*) or **4b** (0.82 g, 3.81 mmol, *reaction b*) and pyridine hydrochloride (1.98 g, 17.1 mmol for substrate **4a**; 4.4 g, 38.1 mmol for substrate **4b**) was heated in a 100-mL flask at 160°C (oil bath) on continuous stirring for 20 minutes. After cooling to rt brine (10 ml for substrate **4a**; 18 mL for substrate **4b**), water (10 or 20 mL, respectively) was added. Solid was filtered off, dissolved in ethyl acetate, passed through a pad of SiO_2 and washed with ethyl acetate untill complete product was obtained (TLC-control). The product was used in the second stage without further purification. The entire product of the first stage was dissolved in dry methanol (in 25 and 50 ml, respectively), and KOH was added (4.3 mmol; 0.240 g, 2.5 equiv. - continuation of the *reaction a* and 9.5 mmol; 0.534 g, 2.5 equiv. - continuation of the *reaction b*). The reaction mixture was heated to 70°C and stirred for 18h. After this time, the mixture was cooled, extracted with ethyl acetate (3x80 ml), and the combined organic layers were dried over $MgSO_4$. Concentration in vacuo and purification by flash column chromatography on silica gel using a mixture of appropriate solvents give the desired product. The following products were obtained: **5a** (63% yield, 1.077 mmol, 0.217 g) and **5b** (73% yield, 2.78 mmol, 0.560 g).

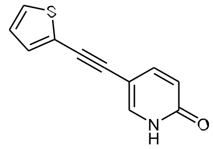
5a 63%

5-(Thiophen-2-ylethynyl)pyridin-2(1*H*)-one (**5a**): Brown solid, m.p. 164-166°C. ^{1}H NMR (400 MHz, DMSO) δ 6.37 (dd, *J* = 9.5, 0.8 Hz, 1H, CH-3), 7.11 (dd, *J* = 5.2, 3.6 Hz, 1H, 2Th), 7.35 (dd, *J* = 3.6, 1.2 Hz, 1H, 2Th), 7.52 (dd, *J* = 9.5, 2.7 Hz, 1H, CH-4), 7.63 (dd, *J* = 5.2, 1.2 Hz, 1H, 2Th), 7.78 (dd, *J* = 2.7, 0.8 Hz, 1H, CH-9), 12.00 (s, 1H, NH). ^{13}C NMR (101 MHz, DMSO) δ 82.60, 89.94 (2 x ≡C), 100.37 (C-5), 120.66 (CH-3), 122.57 (2Th), 128.18, 129.02, 132.70 (2ThH), 140.48 (CH-9), 142.86 (CH-4), 161.63 (C=O). GC-MS (EI, 70eV): m/z = 201 (100) [$M^{+\cdot}$], 172 (14), 145 (21). HRMS (ESI-TOF) m/z: $[M + H]^+$ Calcd for $C_{11}H_8NOS$ 202.0327; Found 216.0322.

5-(Thiophen-3-ylethynyl)pyridin-2(1H)-one (**5b**): Brown solid, m.p. 200-202°C. ^{1}H NMR (400 MHz, DMSO) δ 6.37 (dd, *J* = 9.5, 0.7 Hz, 1H, CH-3), 7.21 (dd, *J* = 5.0, 1.2 Hz, 1H, 3Th), 7.51 (dd, *J* = 9.5, 2.6 Hz, 1H, CH-4), 7.63 (dd, *J* = 5.0, 2.9 Hz, 1H, 3Th), 7.73 (dd, *J* = 2.6, 0.7 Hz, 1H, CH-6), 7.81 (dd, *J* = 2.9, 1.2 Hz, 1H, 3Th), 11.96 (s, 1H, NH). ^{13}C NMR (101 MHz, DMSO) δ 84.32, 85.03 (2 x ≡C), 100.24 (C-5), 120.09 (CH-3), 121.25 (3Th), 126.81, 129.26, 129.46 (3ThH), 139.55 (CH-6), 142.47 (CH-4), 161.07 (C=O). GC-MS (EI, 70eV): m/z = 201 (100) [$M^{+\cdot}$], 200 (12), 173 (15), 172 (15), 146 (12), 145 (23). HRMS (ESI-TOF) m/z: $[M + H]^+$ Calcd for $C_{11}H_8NOS$ 202.0327; Found 216.0323.

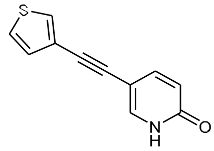
5b 73%

Procedure for the synthesis of compounds **6a** and **6b** (Scheme 2):

Stage 1

To a solution of 5-(thiophen-3-ylethynyl)pyridin-2(1*H*)-one (**5a**, 0.3573g; 1.78 mmole) or 5-(thiophen-2-ylethynyl)pyridin-2(1H)-one (**5b**, 0.5204g; 2.59 mmole) in anhydrous toluene (20 or 30 ml respectively) Lawesson reagent (0.98 mmole; 0.3950g, for substrate **5a** or 1.42 mmole; 0.5753g, for substrate **5b**) was added, and the mixture was stirred for 5 h at 120°C (oil bath temperature). After this time, the mixture was cooled to rt and left standing without stirring overnight. The crude products were purified by silica gel column chromatography using appropriate solvent mixtures to obtain the desired products. Due to the fact that the products of this stage (**5a** and **5b**) turned out to be unstable, they were used directly in the benzyl group addition stage (Stage 2).

Stage 2

A stirred solution of BnMgCl (1.4 M in THF; 0.6 mmol, 0.43 mL for reaction with *N*-lithium 5-(thiophen-3-ylethynyl)pyridin-2(1*H*)-thione (*reaction a*) or 1.2 mmol, 0.86 mL for reaction with *N*-lithium 5-(thiophen-2-ylethynyl)pyridin-2(1*H*)-thione (*reaction b*)) in dry THF (2 or 4 mL respectively) in a Schlenk flask was cooled to 0°C under argon, and MeLi (3.1 M in diethoxymethane; 1.2 mmol, 0.39 mL (*reaction a*) or 2.4 mmol, 0.78 mL (*reaction b*)) was added by syringe over 5 min. The resulting solution was stirred for 5 min at 0°C. In the second Schlenk flask, the solution of *N*-lithium 2-thiopyridone was prepared by adding by syringe a solution of MeLi (3.1 M in

diethoxymethane; 0.42 mmol, 0.14 mL – deprotonation of 5-(thiophen-3-ylethynyl)pyridin-2(1*H*)-thione; 0.84 mmol, 0.28 mL – deprotonation of 5-(thiophen-2-ylethynyl)pyridin-2(1*H*)-thione) to the solution of corresponding *NH* 2-(thio)pyridone (0.4 mmol or 0.8 mmol respectively) in THF (6 or 12 mL) at 0°C. The solution containing lithium benzyldimethyl-magnesiate and LiCl (prepared in the first Schlenk flask) was then transferred by syringe to a cooled (0°C) solution of NLi compound (prepared in the second Schlenk flask). The resulting solution was stirred for 30 minutes at 0°C and 120 minutes at room temperature. After this time, the mixture was carefully quenched with saturated aqueous NH_4Cl (5 mL) using the ice-water bath. The aqueous layer was extracted with ethyl acetate (3 x 50 mL), and the combined organic layers were dried with $MgSO_4$. The mixture was filtered, and the solvents were evaporated under reduced pressure. The crude product was purified by column chromatography on silica gel using a mixture of appropriate solvents to give the desired product. The following products were obtained: **6a** (50% yield, 0.89 mmol, 0.275 g) and **6b** (47% yield, 1.22 mmol, 0.377 g).

(*4RS)*-4-Benzyl-5-(thiophen-2-ylethynyl)-3,4-dihydropyridine-2(1H)-thione (**6a**): Brown solid, m.p. 108-110°C. ^{1}H NMR (400 MHz, $CDCl_3$) δ 2.60 (dd, *J* = 13.2, 9.8 Hz, 1H, 4-CHH), 2.71 (dddd, *J* = 9.8, 6.6, 5.3, 4.6 Hz, 1H, CH-4), 2.85 (dd, *J* = 17.1, 6.6 Hz, 1H, CHH-3), 2.95 (dd, *J* = 17.1, 5.3 Hz, 1H, CHH-3), 3.05 (dd, *J* = 13.2, 4.6 Hz, 1H, 4-CHH), 6.48 (d, *J* = 4.8 Hz, 1H, =CH-6), 6.99 (dd, *J* = 5.2, 3.6 Hz, 1H, 2Th), 7.17 (dd, *J* = 3.7, 1.2 Hz, 1H, 2Th), 7.20 – 7.36 (m, 6H, C_6H_5, 2Th), 9.70 (d, *J* = 4.6 Hz, 1H, NH). ^{13}C NMR (101 MHz, $CDCl_3$) δ 36.78 (4-CH), 38.21 (4-CH_2), 42.24 (CH_2-3), 87.43, 90.10 (2 x ≡C), 109.44 (C-5), 122.99 (2Th), 126.64, 127.19, 127.51 (2ThH), 128.28 (=CH-6), 128.49 (2C), 129.51 (2C), 131.86 (ArH), 138.06 (Ar), 198.42 (C=S). GC-MS (EI, 70eV): m/z = 309 (63) [$M^{+\cdot}$], 218 (100), 217 (17), 185 (46), 173 (13), 147 (10), 91 (27). HRMS (ESI-TOF) m/z: $[M + H]^+$ Calcd for $C_{18}H_{16}NS_2$ 310.0724; Found 310.0723.

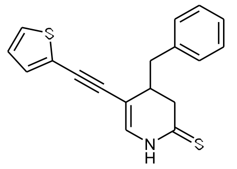

6a 50%

(4*RS*)-4-Benzyl-5-(thiophen-3-ylethynyl)-3,4-dihydropyridine-2(1H)-thione (**6b**): Yellow solid, m.p. 153-156°C. ^{1}H NMR (400 MHz, $CDCl_3$) δ 2.61 (dd, *J* = 13.1, 9.8 Hz, 1H, 4-CHH), 2.70 (dq, *J* = 9.8, 6.6, 5.3, 4.5 Hz, 1H, CH-4), 2.85 (dd, *J* = 17.1, 6.6 Hz, 1H, CHH-3), 2.95 (dd, *J* = 17.1, 5.3 Hz, 1H, CHH-3), 3.06 (dd, *J* = 13.1, 4.5 Hz, 1H, 4-CHH), 6.48 (d, *J* = 4.7 Hz, 1H, =CH-6), 7.08 (dd, *J* = 5.0, 1.2 Hz, 1H, 3Th), 7.19 – 7.34 (m, 6H, C_6H_5, 3Th), 7.41 (dd, *J* = 3.0, 1.2 Hz, 1H, 3Th), 9.55 (d, *J* = 4.4 Hz, 1H, NH). ^{13}C NMR (101 MHz, $CDCl_3$) δ 36.96 (CH-4), 38.26 (4-CH_2), 42.30 (CH_2-3), 85.92, 89.47 (2 x ≡C), 109.79 (C-5), 122.04 (3Th), 125.58, 126.65 (3ThH), 128.12 (=CH-6), 128.53 (2C, ArH), 128.60 (3ThH), 129.58 (2C, ArH), 129.69 (ArH), 138.21 (Ar), 198.44 (C=S). GC-MS (EI, 70eV): m/z = 309 (50) [$M^{+\cdot}$], 218 (100), 217 (14), 186 (15), 185 (28), 173 (10), 91 (24). HRMS (ESI-TOF) m/z: $[M + H]^+$ Calcd for $C_{18}H_{16}NS_2$ 310.0724; Found 310.0723.

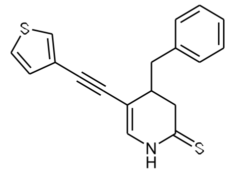

6b 47%

Procedure for the synthesis of compounds **8a-8c** (Scheme 3):

(6-Methoxypyridin-3-yl)boronic acid (**7a**) (1.0 g, 6.55 mmol) and sodium azide (0.8501 g, 13.1 mmol) were stirred in water : MeCN (1:1 v/v, 100 mL in total) for 10 min at room temperature. $CuSO_4$▪$5H_2O$ (0.654mmole, 0.1634g, 10 mol %) was then added, and the reaction mixture was stirred for another 30 min. The in situ formation of aryl azide was monitored by TLC. The alkyne (7.2 mmol) was added, and the reaction mixture was stirred at room temperature for 18h. (The completion of the reaction was monitored by GC-MS.) The reaction mixture was filtered through the Celite, and the residue was washed with ethyl acetate (100 mL). The filtrate was extracted with EtOAc (3x70 mL) and dried over anhydrous sodium sulfate. The combined organic layer was concentrated *in vacuo*, and the crude reaction mixture was purified by flash column chromatography using EtOAc : hexane as an eluting solvent to give the desired product. The following products were obtained: **8a** (23% yield, 1.507 mmol, 0.3891 g), **8b** (27% yield, 1.77 mmol, 0.4462 g) and **8c** (45% yield, 2.95 mmol, 0.7465 g).

2-Methoxy-5-(4-(thiophen-3-yl)-1*H*-1,2,3-triazol-1-yl)pyridine (**8a**): White solid, m.p. 165-167°C. ^1H NMR (400 MHz, DMSO) δ 3.95 (s, 3H, OCH_3), 7.10 (d, *J* = 8.9 Hz, 1H, CH-3), 7.58 (dd, *J* = 5.0, 1.3 Hz, 1H, 3Th), 7.72 (dd, *J* = 5.1, 3.0 Hz, 1H, 3Th), 7.95 (dd, *J* = 2.9, 1.2 Hz, 1H, 3Th), 8.25 (dd, *J* = 8.9, 2.8 Hz, 1H, CH-4), 8.73 (d, *J* = 2.8 Hz, 1H), 9.10 (s, 1H, CH-6). ^{13}C NMR (101 MHz, DMSO) δ 53.75 (OCH_3), 111.36 (CH-3), 119.73 (CH-5'), 121.41, 125.70, 127.38 (3ThH), 128.23 (C-5), 131.34 (3Th), 132.11 (CH-4), 138.79 (CH-6), 143.73 (C-4'), 163.25 (C-2). GC-MS (EI, 70eV): m/z = 258 (2) [$M^{+\cdot}$], 231 (15), 230 (75), 229 (100), 215 (24), 202 (13), 201 (41), 200 (18), 187 (21), 160 (14), 122 (47), 96 (38), 93 (14), 80 (14). HRMS (ESI-TOF) m/z: $[M + H]^+$ Calcd for $C_{12}H_{11}N_4OS$ 259.0654; Found 259.0650.

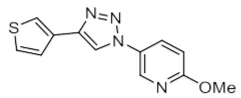

8a 23%

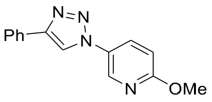

8b 27%

2-Methoxy-5-(4-phenyl-1*H*-1,2,3-triazol-1-yl)pyridine (**8b**): White solid, m.p. 151-153°C. ^1H NMR (400 MHz, DMSO) δ 3.95 (s, 3H, OCH_3), 7.10 (d, *J* = 8.8 Hz, 1H, CH-3), 7.33 – 7.44 (m, 1H, C_6H_5), 7.51 (dd, *J* = 8.3, 7.0 Hz, 2H, C_6H_5), 7.86 – 7.98 (m, 2H, C_6H_5), 8.27 (dd, *J* = 8.8, 2.7 Hz, 1H, CH-4), 8.76 (d, *J* = 2.7 Hz, 1H, CH-9), 9.25 (s, 1H, CH-5').^{13}C NMR (101 MHz, DMSO) δ 53.76 (OCH_3), 111.36 (CH-3), 120.01 (CH-5'), 125.28 (2C), 128.21 (ArH), 128.26 (C-5), 128.95 (2C), (ArH), 130.12 (Ar), 132.10 (CH-4), 138.82 (CH-6), 147.22 (C-4'), 163.28 (C-2). GC-MS (EI, 70eV): m/z = 252 (1) [$M^{+\cdot}$], 224 (100), 223 (87), 209 (16), 195 (46), 193 (20), 181 (21), 154 (29), 127 (15), 116 (39), 102 (11), 93 (12), 89 (29), 80 (13). HRMS (ESI-TOF) m/z: $[M + H]^+$ Calcd for $C_{14}H_{13}N_4O$ 253.1089; Found 253.1086.

2-Methoxy-5-(4-(pyridin-2-yl)-1H-1,2,3-triazol-1-yl)pyridine (**8c**): White solid, m.p. 123-124°C. ^1H NMR (400 MHz, DMSO) δ 3.95 (s, 3H, OCH_3), 7.08 (d, *J* = 8.9 Hz, 1H, CH-3),

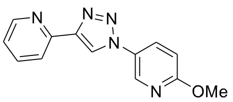

8c 45%

7.42 (ddd, *J* = 7.8, 4.8, 1.2 Hz, 1H, CH-5”), 7.96 (td, *J* = 7.8, 1.8 Hz, 1H, CH-4”), 8.13 (dt, *J* = 7.8, 1.2 Hz, 1H, CH-3”), 8.34 (dd, *J* = 8.9, 2.8 Hz, 1H, CH-4), 8.67 (ddd, *J* = 4.8, 1.8, 1.2 Hz, 1H, CH-6”), 8.82 (d, *J* = 2.8 Hz, 1H, CH-6), 9.31 (s, 1H, CH-5’). ^{13}C NMR (101 MHz, DMSO) δ 54.26 (OCH_3), 111.80 (CH-3), 120.21 (CH-3”), 122.13 (CH-5’), 123.80 (CH-5”), 128.70 (C-5), 132.67 (CH-4), 137.79 (CH-4”), 139.47 (CH-6), 148.62, 149.90, C-4’. C-2”) 150.12 (CH-6”), 163.81 (C-2). GC-MS (EI, 70eV): m/z = 253 (5) [$M^{+\cdot}$], 225 (69), 224 (100), 210 (15), 196 (36), 195 (21), 182 (34), 181 (14), 155 (32), 129 (10), 118 (13), 93 (12), 78 (18). HRMS (ESI-TOF) m/z: $[M + H]^+$ Calcd for $C_{13}H_{12}N_5O$ 254.1042; Found 254.1038.

Procedure for the synthesis of compounds **9a-c** (Scheme 3):
A mixture of 2-methoxypyridine **8a** (0.27g, 1.045 mmol) or **8b** (0.18g, 0.71 mmol) or **8c** (0.6648, 2.63 mmol) and pyridine hydrochloride (1.21 g, 10.45 mmol for substrate **8a** *(reaction a)*; 0.8245 g, 7.135 mmol for substrate **8b** *(reaction b)*; 3.034 g, 26.25 mmol for substrate **8c** *(reaction c)*) was heated in a 100-mL flask at 160°C (oil bath) on continuous stirring for 30 minutes. After cooling to room temperature, brine (10 mL – *reaction a* and *b*; 20 mL - *reaction c*) was added, followed by water (5 mL – *reaction a* and *b*; 10 mL - *reaction c*). The solid was filtered off and purified by flash column chromatography using EtOAc : hexane as an eluting solvent to produce the desired products. According to the procedure, 0.148 g (58% yield) of **9a**, 0.108 g (64% yield) of **9b** and 0.554 g (88% yield) of **9c** were obtained.

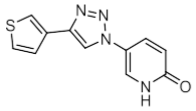

9a 58%

5-(4-(Thiophen-3-yl)-1H-1,2,3-triazol-1-yl)pyridin-2(1*H*)-one (**9a**): White solid, m.p. 290-291°C (decomposition). ^{1}H NMR (400 MHz, DMSO) δ 6.58 (d, *J* = 9.6 Hz, 1H, CH-3), 7.54 (dd, *J* = 5.0, 1.3 Hz, 1H, 3Th), 7.70 (dd, *J* = 5.0, 2.9 Hz, 1H, 3Th), 7.90 (dd, *J* = 3.0, 1.3 Hz, 1H, 3Th), 7.98 (dd, *J* = 9.7, 3.0 Hz, 1H, CH-4), 8.09 (d, *J* = 3.1 Hz, 1H, CH-6), 8.94 (s, 1H, CH-5’), 12.08 (s, 1H, NH). ^{13}C NMR (101 MHz, DMSO) δ 119.11 (C-5), 119.71 (CH-3, br), 119.87 (CH-5’), 121.21, 125.62, 127.34 (3ThH), 129.79 (CH-6 br), 131.41 (3Th), 135.01 (CH-4), 143.38 (C-4’), 161.64 (C=O). GC-MS (EI, 70eV): decomposition. HRMS (ESI-TOF) m/z: $[M + H]^+$ Calcd for $C_{11}H_9N_4OS$ 245.0497; Found 245.0490.

5-(4-Phenyl-1H-1,2,3-triazol-1-yl)pyridin-2(1*H*)-one (**9b**): White solid, m.p. 300-305°C

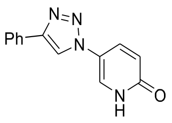

9b 64%

(decomposition). ^{1}H NMR (400 MHz, DMSO) δ 6.59 (d, *J* = 9.6 Hz, 1H, CH-3), 7.38 (t, *J* = 7.4 Hz, 1H, C_6H_5), 7.50 (t, *J* = 7.7 Hz, 2H, C_6H_5), 7.78 – 7.95 (m, 2H, C_6H_5), 8.00 (dd, *J* = 9.7, 3.0 Hz, 1H, CH-4), 8.12 (d, *J* = 3.0 Hz, 1H, CH-6), 9.09 (s, 1H, CH-5’), 12.11 (s, 1H, NH). ^{13}C NMR (101 MHz, DMSO) δ 119.16 (br ,C-5) 119.71 (br, CH-3), 120.13 (CH-4’), 125.17 (2C), 128.12, 128.93 (2C), ArH, 129.84 (br, CH-6), 130.17 (Ar), 135.01 (CH-4), 146.85 (C-4’), 161.68 (C=O). GC-MS (EI, 70eV): decomposition. HRMS (ESI-TOF) m/z: $[M + H]^+$ Calcd for $C_{13}H_{11}N_4O$ 239.0933; Found 239.0928.

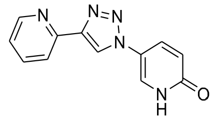
9c 88%

5-(4-(Pyridin-2-yl)-1H-1,2,3-triazol-1-yl)pyridin-2(1*H*)-one (**9c**): Gray solid, m.p. 235-237°C. ^{1}H NMR (400 MHz, DMSO) δ 6.58 (d, *J* = 9.7 Hz, 1H, CH-3), 7.40 (ddd, *J* = 7.5, 4.9, 1.2 Hz, 1H, CH-5''), 7.95 (td, *J* = 7.8, 1.8 Hz, 1H, CH-4''), 8.06 (dd, *J* = 9.7, 3.0 Hz, 1H, CH-4), 8.10 (dt, *J* = 7.8, 1.2 Hz, 1H, CH-3''), 8.19 (d, *J* = 3.0 Hz, 1H, CH-6), 8.59 – 8.73 (m, 1H, CH-6''), 9.15 (s, 1H, CH-5'), 12.10 (s, 1H, NH). ^{13}C NMR (101 MHz, DMSO) δ 119.61 (C-5), 120.17 (CH-3''), 120.23 (br, CH-3), 122.31 (CH-5'), 123.76 (CH-5''), 130.55 (CH-6), 135.63 (CH-4), 137.80 (CH-4''), 148.32, 150.01 (C-2'', C-4'), 150.15 (CH-6''), 162.23 (C=O). GC-MS (EI, 70eV): decomposition. HRMS (ESI-TOF) m/z: $[M + H]^+$ Calcd for $C_{12}H_{10}N_5O$ 240.0885; Found 240.0880.

Procedure for the synthesis of compounds **10a** and **10b** (Scheme 3):

A stirred solution of BnMgCl (1.4 M in THF; 2.1 mmol, 1.5 mL - reaction with **9a** (*reaction a*) or 1.6 mmol, 1.12 mL for reaction with **9b** (*reaction b*)) in dry THF (10 mL) in a Schlenk flask was cooled to 0°C under argon, and MeLi (3.1 M in diethoxymethane; 4.2 mmol, 1.36 mL (for **9a**) or 3.15 mmol, 1.02 mL (for **9b**) was added by syringe over 5 min. The resulting solution was stirred for 5 min at 0°C. In the second Schlenk flask, the solution of *N*-lithium 2-pyridone was prepared by adding by syringe a solution of MeLi (3.1 M in diethoxymethane; 1.54 mmol, 0.5 mL – deprotonation of **9a**; 1.15 mmol, 0.37 mL – deprotonation of **9b**) to the solution of corresponding *NH* 2-pyridone (1.4 mmol or 1.05 mmol respectively) in THF (10 mL) at 0°C. The solution containing lithium benzyldimethyl-magnesiate and LiCl (prepared in the first Schlenk flask) was then transferred by syringe to a cooled (0°C) solution of NLi compound (prepared in the second Schlenk flask). The resulting solution was stirred for 1.5h at 0°C and 2.5h at room temperature. After this time, the mixture was carefully quenched with saturated aqueous NH_4Cl (10 mL) using the ice-water bath. The aqueous layer was extracted with ethyl acetate (3 x 50 mL), and the combined organic layers were dried with $MgSO_4$. The mixture was filtered, and the solvents were evaporated under reduced pressure. The crude product was purified by column chromatography on silica gel using a mixture of appropriate solvents to give the desired products. The following products were obtained: **10a** (18% yield, 0.252 mmol, 0.085 g) and **10b** (41% yield, 0.43 mmol, 0.142 g).

(4*RS*)-4-Benzyl-5-(4-(thiophen-3-yl)-1*H*-1,2,3-triazol-1-yl)-3,4-dihydropyridin-2(1*H*)-one (**10a**):

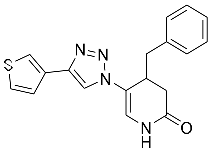
10a 18%

White solid, m.p. 195-197°C. ^{1}H NMR (400 MHz, $CDCl_3$) δ 2.65 (dt, *J* = 16.7, 1.5 Hz, 1H, C<u>H</u>H-3), 2.76 (dd, *J* = 13.5, 8.8 Hz, 1H, 4-CH<u>H</u>), 2.88 (dd, *J* = 16.7, 7.5 Hz, 1H, CH<u>H</u>-3), 3.01 (dd, *J* = 13.5, 5.6 Hz, 1H, 4-CH<u>H</u>), 3.51 (dddd, *J* = 8.8, 7.5, 5.6, 1.5 Hz, 1H, CH-4), 6.79 (d, *J* = 5.2 Hz, 1H, =CH-6), 7.16 – 7.24 (m, 3H, C_6H_5), 7.25 – 7.31 (m, 2H, C_6H_5), 7.38 – 7.44 (m, 2H, 3Th), 7.49 (s, 1H, CH-5'), 7.68 (dd, *J* = 2.7, 1.6

Hz, 1H, 3Th), 7.91 (d, *J* = 5.1 Hz, 1H, NH). ^{13}C NMR (101 MHz, $CDCl_3$) δ 34.68 (CH_2-3), 36.51 (CH-4), 37.95 (4-CH_2), 117.35 (CH-5’), 118.27 (=CH-6), 121.13 (C-5), 121.54, 125.71, 126.57 (3ThH), 126.98 (ArH), 128.67 (2C), 129.42 (2C), 131.25 (3Th), 137.30 (Ar), 144.05 (C-4’), 169.35 (C=O). GC-MS (EI, 70eV): m/z = 336 (1) [$M^{+\cdot}$], 308 (27), 281 (23), 253 (14), 217 (100), 207 (72), 189 (22), 164 (12), 133 (18), 122 (13), 108 (11), 97 (13), 91 (45), 65 (18). HRMS (ESI-TOF) m/z: $[M + H]^+$ Calcd for $C_{18}H_{17}N_4OS$ 337.1123; Found 337.1122.

(4*RS*)-4-Benzyl-5-(4-phenyl-1H-1,2,3-triazol-1-yl)-3,4-dihydropyridin-2(1*H*)-one (**10b**): White solid, m.p. 184-185°C. ^1H NMR (400 MHz, $CDCl_3$) δ 2.64 (dt, *J* = 16.7, 1.5 Hz, 1H, CHH-3), 2.77 (dd, *J* = 13.5, 8.8 Hz, 1H, 4-CHH), 2.88 (dd, *J* = 16.7, 7.5 Hz, 1H, CHH-3), 3.02 (dd, *J* = 13.5, 5.6 Hz, 1H, 4-CHH), 3.53 (dddd, *J* = 8.8, 7.5, 5.6, 1.5 Hz, 1H, CH-4), 6.81 (d, *J* = 5.2 Hz, 1H, =CH-6), 7.13 – 7.22 (m, 3H, C_6H_5), 7.28 (td, *J* = 6.9, 1.9 Hz, 2H, C_6H_5), 7.32 – 7.40 (m, 1H, C_6H_5), 7.45 (dd, *J* = 8.3, 6.7 Hz, 2H, C_6H_5), 7.61 (s, 1H, CH-5’), 7.70 – 7.86 (m, 2H, C_6H_5), 8.20 (d, *J* = 5.2 Hz, 1H, NH). ^{13}C NMR (101 MHz, $CDCl_3$) δ 34.68 (CH_2-3), 36.45 (CH-4), 37.94 (4-CH_2), 117.58 (CH-5’), 118.30 (=CH-6), 121.19 (C-5), 125.77 (2C), 126.96, 128.49, 128.65 (2C), 128.95 (2C), 129.41 (2C), ArH, 130.04 (Ar), 137.31 (Ar), 147.85 (C-4’), 169.65 (C=O). GC-MS (EI, 70eV): m/z = 330 (1) [$M^{+\cdot}$], 302 (35), 301 (11), 211 (100), 207 (14), 184 (19), 183 (52), 182 (18), 156 (19), 128 (13), 116 (21), 102 (18), 91 (69), 77 (13), 65 (12). HRMS (ESI-TOF) m/z: $[M + H]^+$ Calcd for $C_{20}H_{19}N_4O$ 331.1559; Found 331.1554.

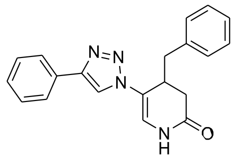

10b 41%

General procedure for the synthesis of 5-hetroaryl substituted 2-methoxypyridines **11a** and **11b** (Schemat 4):

To a 100-mL Schlenk flask, equipped with a magnetic stir bar and a condenser crowned with argon balloon, charged with 40 mL of degassed DMF, heterocyclic halides (4.7 mmol), 1.086 g (7.1 mmol) of (6-methoxypyridin-3-yl)boronic acid (**7a**), and 0.271 g (5 mol%) tetrakis(triphenylpho-sphino)palladium(0) was added. The mixture was stirred at rt for 60 min. Next, 21.1 mL of degassed 1.0 M aqueous Na_2CO_3 solution was added, and the reaction mixture was heated under argon at 80 °C for 12 h in the case of 3BTh-Br and 4BTh-Br. After this time, the reaction mixture was cooled to rt., and the solvent was removed under reduced pressure. The residue was extracted by ethyl acetate, and the organic layer was washed with brine and dried over $MgSO_4$. The mixture was filtered, and the solvents were evaporated under reduced pressure. The crude product was purified by column chromatography on silica gel using a mixture of *n*-hexane and ethyl acetate (11:1 v/v) as the eluent yielded 1.028 g (91%) of **11a** and 0.926 g (82%) of **11b** as products.

5-(Benzo[*b*]thiophen-3-yl)-2-methoxypyridine (**11a**): Pale yellow solid, m.p. 43-47°C (*n*-hexane/ethyl acetate). ^1H NMR (400 MHz, $CDCl_3$) δ 4.01 (s, 3H, OCH_3), 6.87 (dd, *J* = 8.4, 0.7

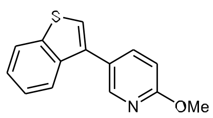

11b 91%

Hz, 1H, CH-3), 7.36 (s, 1H, 3BTh), 7.38 – 7.49 (m, 2H, 3BTh), 7.78 (dd, *J* = 8.4, 2.4 Hz, 1H, CH-4), 7.80 – 7.89 (m, 1H, 3BTh), 7.88 – 7.98 (m, 1H, 3BTh), 8.38 (dd, *J* = 2.4, 0.8 Hz, 1H, CH-6). ^{13}C NMR (101 MHz, $CDCl_3$) δ 53.58 (OMe), 110.86 (CH-3), 122.48, 123.01, 123.45, 124.50, 124.57 (3BThH), 125.05, 134.35, 137.85 (Ar), 138.90 (CH-4), 140.62 (Ar), 146.30 (CH-6), 163.61 (C-2). GC-MS (EI, 70eV): m/z = 241 (100) [$M^{+\cdot}$], 240 (63), 212 (42), 210 (23), 171 (24), 127 (10). HRMS (ESI-TOF) m/z: $[M + H]^+$ Calcd for $C_{14}H_{12}NOS$ 242.0640; Found 242.0635.

5-(Benzo[b]thiophen-4-yl)-2-methoxypyridine (**11b**): Pale yellow solid, m.p. 50-51°C (*n*-hexane/ethyl

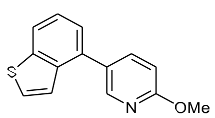

11b 82%

acetate). ^{1}H NMR (400 MHz, $CDCl_3$) δ 4.01 (s, 3H, OCH_3), 6.87 (dd, *J* = 8.5, 0.8 Hz, 1H, CH-3), 7.30 (dd, *J* = 7.3, 1.1 Hz, 1H, 4-BTh), 7.37 – 7.50 (m, 3H, 4-BTh), 7.78 (dd, *J* = 8.5, 2.5 Hz, 1H, CH-4), 7.88 (dt, *J* = 8.1, 1.0 Hz, 1H, 4-BTh), 8.37 (dd, *J* = 2.5, 0.8 Hz, 1H, CH-6). ^{13}C NMR (101 MHz, $CDCl_3$) δ 53.58 (OCH_3), 110.64 (CH-3), 121.85, 122.74, 124.42, 124.60, 126.70 (4BThH), 129.85, 134.14, 137.96 (Ar), 139.27 (CH-4), 140.56 (Ar), 146.52 (CH-6), 163.55 (C-2). GC-MS (EI, 70eV): m/z = 241 (100) [M+], 240 (75), 212 (39), 210 (28), 171 (21). HRMS (ESI-TOF) m/z: $[M + H]^+$ Calcd for $C_{14}H_{12}NOS$ 242.0640; Found 242.0633.

Procedure for the synthesis of 5-hetroaryl substituted pyridine-2(1*H*)-ones **12a** and **12b** (Scheme 4):
A mixture of 2-methoxy-5-(benzo[*b*]thiophen-3(4)yl)pyridine (1.8 mmol) and pyridine hydrochloride (2.08 g, 18 mmol) was heated in a 100 mL flask at 160°C (oil bath) on continuous stirring for 20 min. After cooling to rt brine (40 mL), water (20 mL) was added. The water layer was extracted with ethyl acetate (3 × 100 mL), and the combined organic layers were dried over $MgSO_4$. The mixture was filtered, and the solvents were evaporated under reduced pressure. The crude product was purified by crystallization from isopropyl alcohol and yielded 0.282 g (69%) of **12a** and 0.237 g (58%) of **12b** as products.

5-(Benzo[*b*]thiophen-3-yl)pyridin-2(1*H*)-one (**12a**): White solid, m.p. 201-205°C (*n*-hexane/ethyl

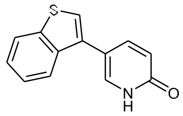

12a 69%

acetate). ^{1}H NMR (400 MHz, DMSO) δ 6.51 (d, *J* = 9.4 Hz, 1H, CH-3), 7.38 – 7.50 (m, 2H, C_6H_4), 7.64 (d, *J* = 2.7 Hz, 1H, CH-6), 7.73 (dd, *J* = 9.4, 2.7 Hz, 1H, CH-4), 7.76 (s, 1H, CH-2'), 7.79 – 7.84 (m, 1H, C_6H_4), 8.01 – 8.10 (m, 1H, C_6H_4), 11.90 (s, 1H, NH). ^{13}C NMR (101 MHz, DMSO) δ 113.09 (C-5), 119.98 (CH-3), 122.13 (CH-2'), 123.18, 123.62, 124.50, 124.58 (ArH), 132.53 (Ar), 134.16 (CH-6), 137.05, 139.85 (Ar), 141.57 (CH-4), 161.68 (C=O). GC-MS (EI, 70eV): m/z = decomposition. HRMS (ESI-TOF) m/z: $[M + H]^+$ Calcd for $C_{13}H_{10}NOS$ 228.0483; Found 228.0476.

5-(Benzo[*b*]thiophen-4-yl)pyridin-2(1*H*)-one (**12b**): White solid, m.p. 228-231°C (*n*-hexane/ethyl

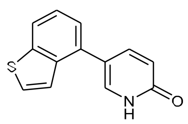

12b 58%

acetate). ^{1}H NMR (400 MHz, DMSO) δ 6.50 (d, *J* = 9.4 Hz, 1H, CH-3), 7.33 (dd, *J*

= 7.3, 1.1 Hz, 1H, ArH), 7.41 (d, *J* = 7.8 Hz, 1H, ArH), 7.42 – 7.45 (m, 1H, ArH), 7.59 (d, *J* = 2.7 Hz, 1H, CH-9), 7.71 (dd, *J* = 9.4, 2.7 Hz, 1H, CH-4), 7.82 (d, *J* = 5.6 Hz, 1H, ArH), 7.99 (dd, *J* = 8.0, 1.0 Hz, 1H, ArH), 11.91 (s, 1H, NH). ^{13}C NMR (101 MHz, DMSO) δ 117.78 (C-5), 119.76, 121.61, 122.32, 123.90, 124.47, 127.78 (ArH), 132.67 (Ar), 134.44 (CH-3), 137.05 (Ar), 139.95 (Ar), 141.91 (CH-4), 161.68 (C=O). GC-MS (EI, 70eV): m/z = decomposition. HRMS (ESI-TOF) m/z: $[M + H]^+$ Calcd for $C_{13}H_{10}NOS$ 228.0483; Found 228.0479.

Procedure for the synthesis of compounds **13a** and **13b** (Scheme 4):
Lawesson's reagent (0.336 g, 0.83 mmol) was added to the solution of 2-pyridone **12a** and **12b** (1.5 mmol) in dry toluene (30 mL). The resulting mixture was stirred for 6 h at 80 °C, concentrated to 1/3 volume under reduced pressure and in this form was applied to column chromatography on silica gel using a mixture of *n*-hexane and ethyl acetate in a ratio of 10:1. Subsequently, it was additionally purified by column chromatography on silica gel using chloroform to afford 0.321 g (88%) of **13a** and 0.277 g (76%) of **13b**.

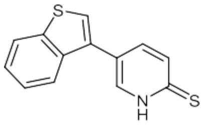

13a 88%

5-(Benzo[*b*]thiophen-3-yl)pyridine-2(1*H*)-thione (**13a**): Yellow solid, m.p. 185-201°C (hexane/ethyl acetate) [from 176 °C, partial sublimation]. ^{1}H NMR (400 MHz, $CDCl_3$) δ 7.40 – 7.50 (m, 3H, C_6H_5, CH-2'), 7.65 (dd, *J* = 8.9, 2.1 Hz, 1H, CH-4), 7.69 (dd, *J* = 8.9, 0.8 Hz, 1H, CH-3), 7.76 (ddt, *J* = 7.4, 3.4, 1.7 Hz, 1H, C_6H_5), 7.84 (dd, *J* = 2.1, 1.0 Hz, 1H, C_{H-6}), 7.90 – 7.97 (m, 1H, C_6H_5), 14.06 (s, 1H, NH). ^{13}C NMR (101 MHz, $CDCl_3$) δ 121.86 (ArH), 123.20 (C-5), 123.27 (ArH), 124.78 (CH-2'), 125.01 (ArH), 125.08 (ArH), 131.47 (Ar), 133.91 (CH-3), 135.49 (CH-6), 136.85 (Ar), 138.41 (CH-4), 140.68 (Ar), 175.75 (C=O). GC-MS (EI, 70eV): m/z = 243 (100) $[M^{+\cdot}]$, 242 (21), 210 (11), 199 (18), 186 (19), 171 (16). HRMS (ESI-TOF) m/z: $[M + H]^+$ Calcd for $C_{13}H_{10}NS_2$ 244.0255; Found 244.0252.

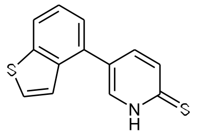

13b 76%

5-(Benzo[*b*]thiophen-4-yl)pyridine-2(1*H*)-thione (**13b**): Yellow solid, m.p. 203-217°C (*n*-hexane/ethyl acetate) [from 174 °C, partial sublimation]. ^{1}H NMR (400 MHz, DMSO) δ 7.36 – 7.50 (m, 4H, 4BTh, CH-3), 7.67 (dd, *J* = 8.9, 2.3 Hz, 1H, CH-4), 7.82 (dd, 2.3, 0.8 Hz, 1H, CH-6), 7.87 (d, *J* = 5.5 Hz, 1H, 4BTh), 8.06 (dt, *J* = 8.0, 1.1 Hz, 1H, 4BTh), 13.79 (s, 1H, NH). ^{13}C NMR (101 MHz, DMSO) δ 121.99, 122.42, 124.14, 124.48 (ArH), 125.03 (Ar), 128.38 (ArH), 131.42(Ar), 132.98 (ArH), 136.53 (CH-3), 136.73 (Ar), 137.72 (CH-4), 140.07 (Ar), 176.66 (C=S). GC-MS (EI, 70eV): m/z = 243 (100) $[M^{+\cdot}]$, 242 (25), 210 (13), 199 (26), 171 (18). HRMS (ESI-TOF) m/z: $[M + H]^+$ Calcd for $C_{13}H_{10}NS_2$ 244.0255; Found 244.0250.

Procedure for the synthesis of compounds **15a** and **15b** (Schemat 5):

To a stirred solution of **14a** (0.7 g, 3.6 mmol) in anhydrous acetonitrile (20 mL), 5.5 mmol of appropriate bromide and 5.5 mmol of NaI (0.823g) was added. The reaction mixture was heated to the boiling point of acetonitrile and refluxing for 17 hours (GC-MS control). After this time, the reaction mixture was cooled and washed with brine. The resulting aqueous layer was extracted 3 times with AcOEt (70 mL) and dried over $MgSO_4$. The mixture was filtered, and the solvents were evaporated under reduced pressure. The crude product was purified by column chromatography on silica gel, using a mixture of appropriate solvents, yielding 0.57 g (50%) of **15a** and 0.6 g (46%) of **15b** as products.

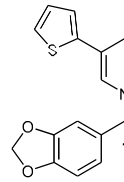
15a 50%

1-(Benzo[d][1,3]dioxol-5-ylmethyl)-5-(thiophen-2-yl)pyridin-2(1*H*)-one (**15a**): Blue oil. ^{1}H NMR (400 MHz, $CDCl_3$) δ 5.08 (s, 2H, NCH_2), 5.95 (s, 2H, CH_2), 6.67 (d, *J* = 9.4 Hz, 1H, CH-3), 6.76 – 6.88 (m, 3H, ArH), 6.99 – 7.04 (m, 2H, ArH), 7.17 – 7.23 (m, 1H, ArH), 7.49 (d, *J* = 2.6 Hz, 1H, CH-6), 7.57 (dd, *J* = 9.4, 2.6 Hz, 1H, CH-4). ^{13}C NMR (101 MHz, $CDCl_3$) δ 52.03 (NCH_2), 101.24 (OCH_2O), 108.48, 108.78 (ArH), 114.75 (C-5), 121.33 (CH-3), 121.84, 122.67, 124.15, 128.03 (ArH), 129.88 (Ar), 133.30 (CH-6), 138.71 (CH-4), 139.37, 147.61, 148.20 (Ar), 161.70 (C=O). GC-MS (EI 70Ev) m/z= 311 (23) [$M^{+\cdot}$], 135 (100), 77 (17). HRMS (ESI-TOF) m/z: $[M + H]^+$ Calcd for $C_{17}H_{14}NO_3S$ 312.0694; Found 312.0691.

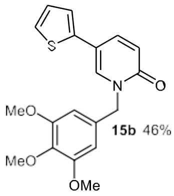
15b 46%

5-(Thiophen-2-yl)-1-(3,4,5-trimethoxybenzyl)pyridin-2(1*H*)-one (**15b**): Oil. ^{1}H NMR (400 MHz, $CDCl_3$) δ 3.83 (s, 3H, OCH_3), 3.85 (s, 6H, 2 x OCH_3), 5.11 (s, 2H, CH_2), 6.58 (s, 2H, ArH), 6.68 (d, *J* = 9.4 Hz, 1H, CH-3), 6.98 – 7.08 (m, 2H, ArH), 7.21 (dd, *J* = 4.5, 1.9 Hz, 1H, ArH), 7.53 (d, *J* = 2.5 Hz, 1H, CH-6), 7.60 (dd, *J* = 9.4, 2.5 Hz, 1H, CH-4). ^{13}C NMR (101 MHz, $CDCl_3$) δ 52.37 (CH_2), 56.25 (2C, 2 x OCH_3), 60.85 (OCH_3), 105.41 (2C, ArH), 114.77 (Ar), 121.35 (CH-3), 122.73, 124.22 (ArH), 128.10 (CH-4), 131.73 (Ar), 133.34 (CH-6), 137.98 (Ar), 138.80 (CH-4), 139.35 (Ar), 153.65 (2C, Ar), 161.76 (C=O). GC-MS (EI 70Ev) m/z = 357 (11) [$M^{+\cdot}$], 281 (16), 207 (39), 181 (100), 73 (13). HRMS (ESI-TOF) m/z: $[M + H]^+$ Calcd for $C_{19}H_{20}NO_3S_2$ 374.0885; Found 374.0883.

Procedure for the synthesis of compounds **15c** and **15d** (Scheme 5):

The mixture of **14a** or **14b** (4 mmol), NaI (12 mmol) and cyclopropylmethyl bromide (12 mmol) was heated in CH_3CN (30 mL) at 80 °C for 4 days. Subsequently, the solvent was evaporated, and brine was added. The aqueous solution was extracted with ethyl acetate (3 x 50 mL), and the combined organic layers were dried over $MgSO_4$. The mixture was filtered, and the solvents were evaporated under reduced pressure. The crude product was purified by column chromatography on silica gel using a mixture of *n*-hexane and ethyl acetate (5:1 → 1:1 v/v) as the eluent yielded 0.407 g (44%) of **15c** and 0.319 g (37%) of **15d** as products.

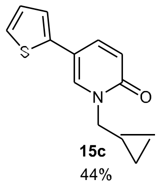

15c 44%

1-(Cyclopropylmethyl)-5-(thiophen-2-yl)pyridin-2(1*H*)-one (**15c**): Pink solid, m.p. 89-90°C (*n*-hexane/ethyl acetate). ^{1}H NMR (400 MHz, $CDCl_3$) δ 0.39 – 0.46 (m, 2H, CH_2), 0.59 – 0.71 (m, 2H, CH_2), 1.22 – 1.37 (m, 1H, CH), 3.86 (d, *J* = 7.2 Hz, 2H, NCH_2), 6.68 (d, *J* = 9.3 Hz, 1H, CH-3), 7.01 – 7.11 (m, 2H, 2ThH), 7.23 (dd, *J* = 5.0, 1.3 Hz, 1H, 2ThH), 7.61 (dd, *J* = 9.3, 2.6 Hz, 1H, CH-4), 7.64 (d, *J* = 2.6 Hz, 1H, CH-6). ^{13}C NMR (101 MHz, $CDCl_3$) δ 4.01 (2C, 2 x CH_2), 10.63 (CH), 53.91 (NCH_2), 114.58 (C-5), 120.96 (CH-3), 122.57, 124.08, 128.09 (2ThH), 133.51 (CH-6), 138.57 (CH-4), 139.59 (2Th), 161.83 (C=O). GC-MS (EI, 70eV): m/z = 231 (63) [M+], 230 (11), 214 (18), 203 (24), 202 (61), 177 (100), 162 (11), 160 (9), 149 (38), 148 (14), 122 (11), 121 (32), 51 (17). HRMS (ESI-TOF) m/z: [M + H]+ Calcd for $C_{13}H_{14}NOS$ 232.0796; Found 232.0792.

1-(Cyclopropylmethyl)-5-(furan-2-yl)pyridin-2(1*H*)-one (**15d**): Beige solid, m.p. 72-73°C (*n*-hexane/ethyl acetate). ^{1}H NMR (400 MHz, $CDCl_3$) δ 0.32 – 0.49 (m, 2H, CH_2), 0.55 – 0.76 (m, 2H, CH_2), 1.11 – 1.45 (m, 1H, CH), 3.87 (d, *J* = 7.2 Hz, 2H, NCH_2), 6.41 (dd, *J* = 3.4, 0.8 Hz, 1H, 2FuH), 6.45 (dd, *J* = 3.4, 1.8 Hz, 1H, 2FuH), 6.67 (d, *J* = 9.4 Hz, 1H, CH-3), 7.40 (dd, *J* = 1.8, 0.8 Hz, 1H, 2FuH), 7.60 (dd, *J* = 9.4, 2.5 Hz, 1H, CH-4), 7.77 (d, *J* = 2.5 Hz, 1H, CH-6). ^{13}C NMR (101 MHz, $CDCl_3$) δ 4.00 (2C, CH_2), 10.62 (CH), 54.02 (NCH_2), 103.80 (2FuH), 111.46 (=C-5), 111.65 (2FuH), 120.99 (CH-3), 132.24 (CH-6), 136.30 (CH-4), 141.57 (2FuH), 150.29 (2Fu), 161.81 (C=O). GC-MS (EI, 70eV): m/z = 215 (58) [$M^{+\cdot}$], 198 (18), 187 (21), 186 (50), 161 (100), 133 (21), 105 (26), 104 (21), 77 (12), 55 (13), 51 (12). HRMS (ESI-TOF) m/z: $[M + H]^+$ Calcd for $C_{13}H_{14}NO_2$ 216.1025; Found 216.1021.

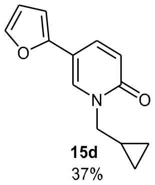

15d 37%

Procedure for the synthesis of compounds **16a** – **16c** (Schemat 5):

Lawesson's reagent (0.336 g, 0.83 mmol, 0.55 eq.) was added to the solution of 2-pyridone **15** (1.5 mmol) in anhydrous toluene (30 mL). The resulting mixture was stirred for 1.5-2 h at 120 °C (TLC control), then cooled, concentrated to 1/3 volume under reduced pressure, and in this form, was applied to column chromatography on silica gel using a mixture of *n*-hexane and ethyl acetate in a ratio of 10:2-10:3. Subsequently, it was additionally purified by column chromatography on silica gel using chloroform to afford 0.252 g (54%) of **16a**, 0.295 g (55%) of **16b**, 0.308 g (83%) of **16c** and 0.226 g (65%) of **16d**.

1-(Benzo[d][1,3]dioxol-5-ylmethyl)-5-(thiophen-2-yl)pyridine-2(1*H*)-thione (**16a**): Yellow solid. ^{1}H NMR (400 MHz, $CDCl_3$) δ 5.75 (s, 2H, NCH_2), 5.97 (s, 2H, OCH_2O), 6.81 (d, *J* = 7.9 Hz, 1H, ArH), 6.86 (dd, *J* = 7.9, 1.7 Hz, 1H, ArH), 6.91 (d, *J* = 1.7 Hz, 1H, ArH), 7.06 (dd, *J* = 5.1, 3.6 Hz, 1H, ArH), 7.13 (dd, *J* = 3.7, 1.2 Hz, 1H, ArH), 7.29 (dd, *J* = 5.0, 1.1 Hz, 1H, ArH), 7.38 (dd, *J* = 9.0, 2.2 Hz, 1H, CH-3), 7.75 (dd, *J* = 9.2, 0.7 Hz, 1H, CH-4), 7.77 (dd, *J* = 2.2, 0.7 Hz, 1H, CH-6). ^{13}C NMR (101 MHz, $CDCl_3$) δ 58.75 (NCH_2),

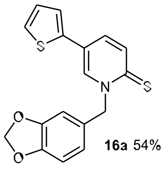

16a 54%

101.37 (CH_2OCH_2), 108.67, 108.93 (ArH), 121.79 (Ar), 122.28, 123.81, 125.48, 128.36, 128.66 (ArH), 131.90 (CH-3), 135.62 (CH-6), 136.41 (CH-4), 138.07, 147.91, 148.36 (Ar), 179.39 (C=S). GC-MS (EI 70Ev) m/z= 327 (24) [$M^{+\cdot}$], 294 (37), 135 (100), 77 (25). HRMS (ESI-TOF) m/z: $[M + H]^+$ Calcd for $C_{17}H_{14}NO_2S_2$ 328.0466; Found 328.0459.

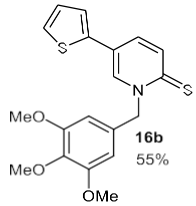
16b 55%

5-(Thiophen-2-yl)-1-(3,4,5-trimethoxybenzyl)pyridine-2(1*H*)-thione (**16b**): Solid, m.p. 98-99°C. ^1H NMR (400 MHz, $CDCl_3$) δ 3.85 (s, 3H, OCH_3), 3.85 (s, 6H, 2 x OCH_3), 5.77 (s, 2H, NCH_2), 6.65 (s, 2H, ArH), 7.06 (dd, *J* = 5.1, 3.6 Hz, 1H, 2ThH), 7.14 (dd, *J* = 3.6, 1.2 Hz, 1H, 2ThH), 7.29 (dd, *J* = 5.1, 1.2 Hz, 1H, 2ThH), 7.39 (dd, *J* = 9.0, 2.2 Hz, 1H, CH-4), 7.77 (d, *J* = 9.0 Hz, 1H, CH-3), 7.79 (d, *J* = 2.2 Hz, 1H, CH-6). ^{13}C NMR (101 MHz, $CDCl_3$) δ 56.30 (2C, 2 x OCH_3), 59.10 (NCH_2), 60.88 (OCH_3), 105.76 (2C, ArH), 121.80 (Ar), 123.85, 125.52, 128.41 (ArH), 130.54 (Ar), 131.93 (CH-4), 135.59 (CH-6), 136.34 (CH-3), 138.00, 138.25 (Ar), 153.77 (2C, Ar), 179.41 (C=S). GC-MS (EI= 70eV) = 373 (13) [$M^{+\cdot}$], 314 (20), 182 (11), 181 (100), 148 (10). HRMS (ESI-TOF) m/z: $[M + H]^+$ Calcd for $C_{19}H_{20}NO_3S_2$ 374.0885; Found 374.0883.

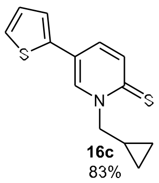
16c 83%

1-(Cyclopropylmethyl)-5-(thiophen-2-yl)pyridine-2(1*H*)-thione (**16c**): Yellow semisolid. ^1H NMR (400 MHz, $CDCl_3$) δ 0.44 – 0.59 (m, 2H, CH_2), 0.64 – 0.79 (m, 2H, CH_2), 1.47 – 1.60 (m, 1H, CH), 4.42 (d, *J* = 7.2 Hz, 2H, NCH_2), 7.10 (dd, *J* = 5.1, 3.6 Hz, 1H, 2ThH), 7.22 (dd, *J* = 3.6, 1.2 Hz, 1H, 2ThH), 7.32 (dd, *J* = 5.1, 1.2 Hz, 1H, 2ThH), 7.42 (dd, *J* = 8.9, 2.2 Hz, 1H, CH-4), 7.73 (d, *J* = 8.9 Hz, 1H, CH-3), 8.04 (d, *J* = 2.2 Hz, 1H, CH-6). ^{13}C NMR (101 MHz, $CDCl_3$) δ 4.24 (2C, 2 x CH_2), 9.97 (CH), 61.15 (NCH_2), 121.72 (C-5), 123.79, 125.42, 128.42 (2ThH), 131.90 (CH-4), 135.77 (CH-6), 136.27 (CH-3), 138.28 (2Th), 178.32 (C=S). GC-MS (EI, 70eV): m/z = 247 (26) [M+], 219 (18), 218 (100), 214 (29), 200 (19), 121 (14). HRMS (ESI-TOF) m/z: $[M + H]^+$ Calcd for $C_{13}H_{14}NS_2$ 248.0568; Found 248.0563.

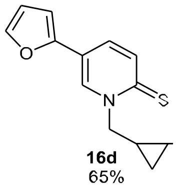
16d 65%

1-(Cyclopropylmethyl)-5-(furan-2-yl)pyridine-2(1*H*)-thione (**16d**): Yellow semisolid. ^1H NMR (400 MHz, $CDCl_3$) δ 0.47 – 0.57 (m, 2H, CH_2), 0.66 – 0.77 (m, 2H, CH_2), 1.49 – 1.61 (m, 1H, CH), 4.44 (d, *J* = 7.2 Hz, 2H, NCH_2), 6.50 (dd, *J* = 3.4, 1.8 Hz, 1H, 2FuH), 6.60 (d, *J* = 3.4 Hz, 1H, 2FuH), 7.42 (d, *J* = 9.0 Hz, 1H, CH-3), 7.46 (d, *J* = 1.8 Hz, 1H, 2FuH), 7.75 (d, *J* = 9.0 Hz, 1H, CH-4), 8.16 (s, 1H, CH-6). ^{13}C NMR (101 MHz, $CDCl_3$) δ 4.23 (2C, 2 x CH_2), 9.96 (CH), 61.30 (NCH_2), 106.00 (2FuH), 111.99 (2FuH), 118.24 (=C-5), 129.45 (=CH-3), 134.37 (CH-9), 136.26 (CH-4), 142.53 (2FuH), 149.18 (2Fu), 178.37 (C=S). GC-MS (EI, 70eV): m/z = 231 (31) [$M^{+\cdot}$], 203 (16), 202 (100), 198 (27), 184 (19). HRMS (ESI-TOF) m/z: $[M + H]^+$ Calcd for $C_{13}H_{14}NOS$ 232.0796; Found 232.0790.

Procedure for the synthesis of compounds **17c** and **17d** (Schemat 5):

To a 50 mL Schlenk flask, the solution of BnMgCl l (1.4 M in THF; 1.6 mmol, 1.15 mL or) in dry THF (5 mL) was added under argon and cooled to 0 °C, and MeLi (3.1 M in cyclohexane; 3.2 mmol, 1.04 mL) was added by syringe over 5 minutes. The resulting solution was stirred for 5 minutes and then cooled to -80°C. The solution containing lithium benzyldimethylmagnesiate and LiCl was then transferred with a syringe to a precooled (-80°C) solution of N-substituted 2-thiopyridone (1.1 mmol) in THF (20 mL) prepared in another Schlenk flask. The resulting solution was stirred for 1.5 h at -80 °C (TLC control). Subsequently, the mixture was carefully quenched with saturated aqueous NH_4Cl (10 mL), allowed to warm up to rt and diluted with water (ca. 10 mL). The aqueous layer was extracted with ethyl acetate (3 x 50 mL), and the combined organic layers were dried with $MgSO_4$. The mixture was filtered, and the solvents were evaporated under reduced pressure. The crude product was purified by column chromatography on silica gel using a mixture of n-hexane and ethyl acetate (10 : 1 v/v) as the eluent to afford 0.213 g (58% yield) of **17c** and 0.165 g (51% yield) of **17d** as products.

(6*RS*)-6-Benzyl-1-(cyclopropylmethyl)-5-(thiophen-2-yl)-3,6-dihydropyridine-2(1*H*)-thione (**17c**):

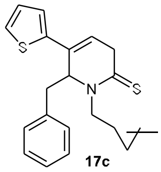

17c
58%

Yellow semisolid. ^{1}H NMR (400 MHz, $CDCl_3$) δ 0.23 – 0.48 (m, 2H, CH_2), 0.50 – 0.75 (m, 2H, CH_2), 1.17 – 1.44 (m, 1H, CH), 2.21 (dt, *J* = 22.4, 2.5 Hz, 1H, C<u>H</u>H-3), 2.83 – 3.04 (m, 2H, 6-C<u>H</u>H, NC<u>H</u>H), 3.21 (dd, *J* = 13.7, 4.6 Hz, 1H, 6-CH<u>H</u>), 3.53 (ddd, *J* = 22.4, 6.0, 1.1 Hz, 1H, CH<u>H</u>-3), 5.02 – 5.06 (m, 1H, CH-6), 5.09 (dd, *J* = 13.8, 5.7 Hz, 1H, NCH<u>H</u>), 6.01 (dd, *J* = 6.0, 2.5 Hz, 1H, =CH-4), 6.93 – 7.09 (m, 4H, ArH), 7.18 – 7.35 (m, 4H, ArH). ^{13}C NMR (101 MHz, $CDCl_3$) δ 2.65 (CH_2), 4.82 (CH_2), 8.72 (CH), 38.08 (6-CH_2), 42.93 (CH_2-3), 56.82 (NCH_2), 63.50 (CH-6), 120.37 (=CH-4), 122.68, 124.74, 127.40 (2ThH), 127.79, 128.32 (2C), 129.67 (=C-5), 130.31 (2C), (PhH), 134.84 (Ph), 141.01 (2Th), 197.59 (C=S). GC-MS (EI, 70eV): m/z = 339 (5) [$M^{+\cdot}$], 311 (18), 310 (65), 249 (16), 248 (100), 218 (27), 214 (14), 194 (17), 161 (20), 134 (10), 91 (41), 65 (11), 55 (26). HRMS (ESI-TOF) m/z: $[M + H]^+$ Calcd for $C_{20}H_{22}NS_2$ 340.1194; Found 340.1191.

(6-*RS*)-6-Benzyl-1-(cyclopropylmethyl)-5-(furan-2-yl)-3,6-dihydropyridine-2(1*H*)-thione (**17d**):

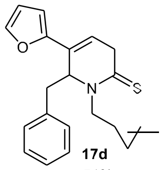

17d
51%

Yellow semisolid. ^{1}H NMR (400 MHz, $CDCl_3$) δ 0.31 – 0.46 (m, 2H, CH_2), 0.52 – 0.68 (m, 2H, CH_2), 1.27 – 1.37 (m, 1H, CH), 2.25 (dt, *J* = 22.5, 2.7 Hz, 1H, C<u>H</u>H-3), 2.94 (dd, *J* = 13.7, 4.6 Hz, 1H, 6-C<u>H</u>H), 3.02 (ddd, *J* = 13.7, 7.9, 0.9 Hz, 1H, NC<u>H</u>H), 3.20 (dd, *J* = 13.7, 4.9 Hz, 1H, 6-CH<u>H</u>), 3.56 (ddd, *J* = 22.5, 6.0, 1.1 Hz, 1H, CH<u>H</u>-3), 4.96 – 5.01 (m, 1H, CH-6), 5.04 (dd, *J* = 13.8, 5.8 Hz, 1H, NCH<u>H</u>), 6.12 (dd, *J* = 6.1, 2.4 Hz, 1H, =CH-4), 6.30 (d, *J* = 3.4 Hz, 1H, 2FuH), 6.44 (dd, *J* = 3.4, 1.8 Hz, 1H, 2FuH), 6.93 – 7.00 (m, 2H, C_6H_5), 7.19 – 7.28 (m, 3H, C_6H_5), 7.42 (d, *J* = 1.8 Hz, 1H, 2FuH). ^{13}C NMR (101 MHz, $CDCl_3$) δ 2.75 (CH_2), 4.69 (CH_2), 8.72 (CH), 38.58 (6-CH_2), 42.71 CH2-3), 56.88 (NCH), 61.46 (CH-6), 105.33, 111.36 (2FuH), 119.01 (=CH-4), 126.29 (=C-5), 127.36, 128.31 (2C), 130.32 (2C), (PhH), 134.88 (Ph), 142.31

(2FuH), 151.01 (2Fu), 197.78 (C=S). GC-MS (EI, 70eV): m/z = 323 (3) $[M^{+\cdot}]$, 295 (22), 294 (82), 233 (16), 232 (100), 204 (16), 202 (27), 198 (14), 178 (18), 145 (22), 91 (40), 65 (12), 55 (23). HRMS (ESI-TOF) m/z: $[M + H]^+$ Calcd for $C_{20}H_{22}NOS$ 324.1422; Found 324.1419.

Procedure for the synthesis of compounds **18e** from **14b** (Scheme 6):

5-(Furan-2-yl)-2-methoxypyridine [36] (**14b**): was obtained from 2-iodofuran, according to the procedure described for the synthesis of compounds **11a** and **11b**. Yield 0,708 g (86%).

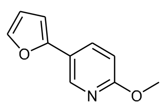

14b
86%

5-(Furan-2-yl)-2-methoxypyridine (**14b**). Pale yellow oil: ^{1}H NMR (400 MHz, $CDCl_3$) δ 3.97 (s, 3H, OCH_3), 6.46 (dd, *J* = 3.5, 1.8 Hz, 1H, 2FuH), 6.56 (dd, *J* = 3.5, 0.8 Hz, 1H, 2FuH), 6.77 (dd, *J* = 8.6, 0.8 Hz, 1H, CH-3), 7.46 (dd, *J* = 1.8, 0.8 Hz, 1H, 2FuH), 7.84 (dd, *J* = 8.7, 2.4 Hz, 1H, CH-4), 8.49 (dd, *J* = 2.4, 0.7 Hz, 1H, CH-6). ^{13}C NMR (101 MHz, $CDCl_3$) δ 53.64 (OCH_3), 104.36, 110.89, 111.60 (2FuH), 120.90 (C-5), 134.54 (CH-3), 142.07, 142.45 (CH-4, CH-6), 151.49, 163.28 (Ar).GC-MS (EI, 70eV): m/z = 175 (100) $[M^{+\cdot}]$, 174 (53), 146 (51), 145 (20), 132 (11), 118 (16), 105 (11), 77 (10).

A solution of 5-(furan-2-yl)-2-methoxypyridine **14b** (0.6655 g, 3.8 mmol), LiCl (0.805 g, 19 mmol) and PTSA (3.6123 g, 19 mmol) in DMF (20 mL) was stirred at 120 °C for 0.5 h. After cooling, the reaction mixture was quenched by adding water (10 mL), extracted with EtOAc (3 x 75 mL), and washed with 15 mL of brine. The combined organic phase was dried over $MgSO_4$. The mixture was filtered, and the solvents were evaporated under reduced pressure. The crude product was purified by crystallization from a mixture of n-hexane and ethyl acetate, yielding 0.480 g (78%) of **15e** as a product.

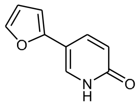

15e
78%

5-(Furan-2-yl)pyridin-2(1H)-one [37] (**15e**): White solid, m.p. 157-158 °C (*n*-hexane/ethyl acetate). ^{1}H NMR (400 MHz, $CDCl_3$) δ 6.43 (dq, *J* = 5.1, 3.3, 2.5 Hz, 2H, ArH), 6.69 (d, *J* = 9.3 Hz, 1H, CH-3), 7.40 (d, *J* = 1.6 Hz, 1H, CH-6), 7.71 – 7.92 (m, 2H, ArH), 120 (br. S, 1H NH). ^{13}C NMR (101 MHz, $CDCl_3$) δ 104.08, 111.59, 112.95, 120.23, 129.94, 138.66, 141.89, 149.91, 164.39. GC-MS (EI, 70eV): m/z = 161 (100) [M+], 132 (16), 104 (33), 78 (14), 51 (14).

Compound **16e** was obtained in 84% yield (0.231 g) according to the procedure for the synthesis of compounds **13a** and **13b**.

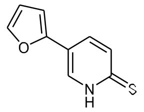

16e 84%

5-(Furan-2-yl)pyridine-2(1*H*)-thione (**16e**): Yellow semisolid. ^{1}H NMR (400 MHz, DMSO) δ 6.48 – 6.59 (m, 1H), 6.92 (d, *J* = 3.4 Hz, 1H), 7.32 (d, *J* = 9.0 Hz, 1H), 7.64 – 7.80 (m, 2H), 7.89 (s, 1H), 13.71 (s, 1H). ^{13}C NMR (101 MHz, DMSO) δ 106.65, 112.65, 117.13, 132.62, 132.88, 133.80, 143.46, 149.35, 176.88. GC-MS (EI, 70eV):

m/z = 177 (100) [$M^{+\cdot}$], 148 (16), 133 (28), 121 (16), 104 (12). HRMS (ESI-TOF) m/z: $[M + H]^+$ Calcd for C_9H_8NOS 178.0327; Found 178.0322.

Procedure for the synthesis of compound **18e**:

In a 25 mL Schlenk flask, the solution of BnMgCl (0.98 mL, 1.4 M in THF; 1.37 mmol) and dry THF (5 mL) was added under argon and cooled to 0°C, followed by the addition of MeLi (0.89 mL, 3.1 M in diethoxymethane; 2.75 mmol) with a syringe over 5 min. The resulting solution was stirred for 5 min at 0°C. In a second Schlenk flask (50 mL), N-lithium 2-thiopyridone solution was prepared by adding with a syringe a solution of MeLi (3.1 M in diethoxymethane; 0.96 mmol, 0.31 mL) to 5-(furan-2-yl)pyridine-2(1*H*)-thione (**16e)** solution (0.92 mmol) in THF (15 mL) at 0 °C. The solution prepared in the first Schlenk flask was then transferred to a cooled (0°C) solution prepared in the second Schlenk flask. The resulting solution was stirred for 1 h at 0°C and 2 h at rt. After this time, the mixture was carefully quenched with saturated aqueous NH_4Cl (10 mL). The aqueous layer was extracted with ethyl acetate (3 x 50 mL), and the combined organic layer was dried with $MgSO_4$. The mixture was filtered, and the solvents were evaporated under reduced pressure. The crude product was purified by column chromatography on silica gel using a mixture of n-hexane and ethyl acetate (1 : 2 v/v) as the eluent yielded 0.107 g (43%) of **18e** as a yellow solid.

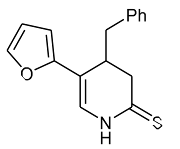

18e 43%

4-Benzyl-5-(furan-2-yl)-3,4-dihydropyridine-2(1*H*)-thione (**18e**): Yellow semisolid. ^{1}H NMR (400 MHz, $CDCl_3$) δ 2.59 (dd, *J* = 12.8, 9.6 Hz, 1H, CHH), 2.69 – 2.90 (m, 3H, CHH, CHH, CH-4), 3.17 (d, *J* = 15.7 Hz, 1H, CHH), 6.30 (d, *J* = 3.4 Hz, 1H, 2Fu), 6.42 (dd, *J* = 3.4, 1.8 Hz, 1H, 2Fu), 6.67 (d, *J* = 4.6 Hz, 1H, =CH-6), 7.15 – 7.35 (m, 5H, C_6H_5), 7.38 (d, *J* = 1.8 Hz, 1H, 2Fu), 9.56 – 10.43 (m, 1H, NH). ^{13}C NMR (101 MHz, $CDCl_3$) δ 34.85 (CH-4), 37.82 (CH_2), 41.40 (CH_2), 106.29 (2FuH), 111.64 (2FuH), 117.93 (=C-5), 118.25 (=CH-6), 126.62, 128.55 (2C), 129.54 (2C), (PhH), 138.46 (Ph), 142.12 (2FuH), 151.03 (2Fu), 197.32 (C=S). GC-MS (EI, 70eV): m/z = 269 (48) [$M^{+\cdot}$], 178 (100), 177 (15), 150 (16), 145 (26), 123 (17), 117 (10), 91 (34), 65 (14). HRMS (ESI-TOF) m/z: $[M + H]^+$ Calcd for $C_{16}H_{16}NOS$ 270.0953; Found 270.0947.

Procedure for the synthesis of compound **20** (Scheme 7):

To a solution of 6-chloro-3-pyridinecarboxylic acid (**19**) (0.7 g, 4.443 mmol) in anhydrous toluene, (7 mL) $SOCl_2$ (0.49 mL, 1.5 eq.) and DMF (0.007 mL 0.02 eq.) were added. The resulting solution was heated at 80°C and stirred for 2.5 h, after which the solvent was evaporated. The precipitate was dissolved in anhydrous CH_2Cl_2, cooled to 0°C and then morpholine (1.16 mL, 13.32 mmol, 3 eq.) was added dropwise over 20 minutes. The reaction mixture was warmed up to rt., stirred for 75 minutes, quenched with a saturated solution of $NaHCO_3$ (aq.) and extracted with ethyl acetate (3 × 70 mL). The combined organic layers were dried over $MgSO_4$. The mixture was filtered, and the solvents were

evaporated under reduced pressure. The crude product was crystallized from *n*-hexane and ethyl acetate to afford 0.676 g (67% yield) of **20** as a yellow solid (Mp. 62-64°C).

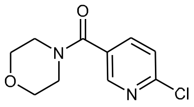

20 67%

(6-Chloropyridin-3-yl)(morpholino)methanone [38] (**20**): ^{1}H NMR (400 MHz, $CDCl_3$) δ 3.01 – 4.07 (m, 8H, 4 x CH_2), 7.42 (dd, *J* = 8.2, 0.8 Hz, 1H, CH-3), 7.75 (dd, *J* = 8.2, 2.4 Hz, 1H, CH-4), 8.46 (dd, *J* = 2.4, 0.8 Hz, 1H, CH-6). ^{13}C NMR (101 MHz, $CDCl_3$) δ 44.72 (br, CH_2N), 48.27 (br, CH_2N), 66.75 (2C, 2 x CH_2O), 124.47 (CH-3), 129.91 (C-5), 138.00 (CH-4), 148.16 (CH-6), 152.89 (C-2), 166.75 (C=O).

Procedure for the synthesis of compound **21** was developed on the basis of the similar synthesis described earlier [21].

To a solution of **20** (0.2 g, 0.882 mmol) in anhydrous THF (2 mL) cooled to 0°C a solution of BnMgCl (2.5 mL, 1.4 M in THF, 4 eq.) was added dropwise over 20 minutes with stirring. The resulting solution was stirred for an additional 1 hour at room temperature. After this time, the solution was cooled to 0°C, and an aqueous solution of NH_4Cl was carefully added over 20 minutes. The mixture was stirred for an additional 18 h at room temperature. After this time, the mixture was extracted 3 times with ethyl acetate (40 mL). Combined organic layers were washed with brine and concentrated on a rotary evaporator, and the residue was digested in ethyl acetate (3 mL) for 1h at room temperature. The crude product was purified by column chromatography (SiO_2, hexane : AcOEt 4:1 → AcOEt → metanol) to afford 0.05 g (20%) of **21** as white semisolid.

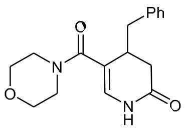

21 20%

(4*RS*)-4-Benzyl-5-(morpholine-4-carbonyl)-3,4-dihydropyridin-2(1*H*)-one (**21**): White semisolid. ^{1}H NMR (400 MHz, $CDCl_3$) δ 2.46 (dd, *J* = 16.6, 3.4 Hz, 1H, CHH-3), 2.58 – 2.77 (m, 2H, CHH-3, 4-CHH), 2.95 (dd, *J* = 13.7, 7.0 Hz, 1H, 4-CHH), 3.22 (dtd, *J* = 8.7, 7.0, 3.4 Hz, 1H, CH-4), 3.37 – 3.45 (m, 2H, CH_2), 3.46 – 3.63 (m, 6H, 3 x CH_2), 6.35 (d, *J* = 4.9 Hz, 1H, =CH-6), 7.15 – 7.25 (m, 3H, C_6H_5), 7.26 – 7.32 (m, 2H, C_6H_5), 8.22 (br s, 1H, NH). ^{13}C NMR (101 MHz, $CDCl_3$) δ 34.47 (CH-4), 35.03 (CH_2-3), 38.82 (4-CH_2), 45.51 (2C, br, 2 x CH_2N), 66.71 (2C, 2 x CH_2O), 115.19 (=C-5), 126.70 (PhH), 127.38 (=CH-6), 128.54 (2C), 129.24 (2C, PhH), 138.09 (Ph), 168.67 (C=O), 170.92 (C=O). GC-MS (EI 70eV)= 300 (18) [$M^{+\cdot}$], 210 (13), 209 (100), 208 (11), 172 (11), 145 (11), 124 (51), 115 (11), 86 (38). HRMS (ESI-TOF) m/z: $[M + H]^+$ Calcd for $C_{17}H_{21}N_2O_3$ 301.1552; Found 301.1548.

Procedure for the synthesis of compounds **23a**,**b** and **24a**,**b** (Scheme 8):

To a cooled to 0°C solution of 1,2-dimethylindole (1.602 mmol) in anhydrous THF (6.6 mL), placed in Schlenk flask, 1.05 eq. of *n*-BuLi (0.7 mL, 2.5M) was added. The reaction mixture was stirred at 0°C for 45 minutes under an argon atmosphere. Next, the solution of lithiated 1,2-dimethylindole was slowly added with a syringe to a cooled to -80°C solution of 1.168 mmol of *N*-phenyl-2-pyridone

(**22a**) or *N*-phenyl-2-pyridinthione (**22b**) (1.068mmol) in anhydrous THF (7 mL) placed in Schlenk flask under argon. The reaction mixture was stirred for 2.5 hours at -80°C, then quenched with saturated NH_4Cl (aq.), warmed to rt, extracted 3 times with AcOEt (3 x 30 mL) and dried over $MgSO_4$. Solvents were evaporated under reduced pressure, and crude reaction products were separated by column chromatography to afford 0.169 g (45%) of **23a** and 0.09 g (24%) of **24a** as well as 0.102 g (29%) of **23b** and 0.105 g (30%) of **24b** as products.

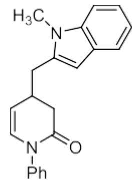

23a 45%

(4*RS*)-4-((1-Methyl-1*H*-indol-2-yl)methyl)-1-phenyl-3,4-dihydropyridin-2(1*H*)-one (**23a**): Beige solid, m.p. 141-146°C. ^{1}H NMR (400 MHz, $CDCl_3$) δ 2.65 (dd, *J* = 15.8, 9.0 Hz, 1H, CHH-3), 2.85 (ddd, *J* = 15.8, 6.3, 0.8 Hz, 1H, CHH-3), 2.91 (d, *J* = 7.4 Hz, 2H, 6-CH_2), 2.98 – 3.07 (m, 1H, CH-4), 3.69 (s, 3H, NCH_3), 5.30 (dd, *J* = 7.8, 3.8 Hz, 1H, =CH-5), 6.29 (dd, *J* = 7.8, 1.5 Hz, 1H, =CH-6), 6.34 (d, *J* = 0.9 Hz, 1H, ArH), 7.09 (ddd, *J* = 8.0, 7.0, 1.1 Hz, 1H, ArH), 7.15 – 7.26 (m, 3H, ArH), 7.27 – 7.31 (m, 2H, ArH), 7.36 – 7.42 (m, 2H, ArH), 7.55 (dt, *J* = 7.8, 1.0 Hz, 1H, ArH). ^{13}C NMR (101 MHz, $CDCl_3$) δ 29.71 (CH_3), 31.60 (CH_2-3), 31.98 (CH-4), 38.37 (4-CH_2), 100.71, 108.98 (ArH), 110.45 (=CH-5), 119.53, 119.98, 121.02, 125.95 (2C), 127.10, 127.73, 129.11 (2C), 130.62 (=CH-6), 137.31, 137.47, 140.29 (Ar), 168.42 (C=O). GC-MS (EI 70Ev) m/z= 316 (10) [$M^{+\cdot}$], 172 (65), 145 (36), 144 (100), 143 (13), 115 (11), 77 (11). HRMS (ESI-TOF) m/z: $[M + H]^+$ Calcd for $C_{21}H_{20}N_2O$ 317.1654; Found 317.1651.

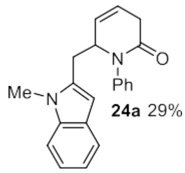

24a 29%

(6*RS*)-6-((1-Methyl-1*H*-indol-2-yl)methyl)-1-phenyl-3,6-dihydropyridin-2(1*H*)-one (**24a**): Brown solid. Mp. 148-150°C. ^{1}H NMR (400 MHz, $CDCl_3$) δ 2.89 (dd, *J* = 14.5, 9.7 Hz, 1H, 6-CHH), 3.03 – 3.10 (m, 2H, CH_2-3), 3.14 (ddt, *J* = 14.5, 3.5, 1.0 Hz, 1H, 6-CHH), 3.41 (t, *J* = 1.0 Hz, 3H, NCH_3), 4.62 (dt, *J* = 9.7, 3.5 Hz, 1H, CH-6), 5.80 – 5.94 (m, 2H, =CH-4, =CH-5), 6.32 (d, *J* = 0.9 Hz, 1H, ArH), 7.08 (ddt, *J* = 7.9, 6.9, 1.1 Hz, 1H, ArH), 7.17 (ddt, *J* = 8.2, 6.9, 1.1 Hz, 1H, ArH), 7.23 (d, *J* = 8.0 Hz, 1H, ArH), 7.32 – 7.40 (m, 3H, ArH), 7.46 – 7.54 (m, 3H, ArH). ^{13}C NMR (101 MHz, $CDCl_3$) δ 29.45 (NCH_3), 32.19 (6-CH_2), 32.81 (CH_2-3), 61.60 (=CH-6), 101.35, 109.01, 119.60, 120.05, 121.17, 122.99, 125.06 (ArH), 127.67 (Ar), 127.73 (ArH), 128.08 (2C), 129.57 (2C), (ArH), 135.19, 137.31, 140.71 (Ar), 167.82 (C=O). GC-MS (EI 70Ev) m/z= 316 (6) [$M^{+\cdot}$], 172 (76), 145 (42), 144 (100), 143 (14), 115 (12), 77 (13). HRMS (ESI-TOF) m/z: $[M + H]^+$ Calcd for $C_{21}H_{20}N_2O$ 317.1654; Found 317.1653.

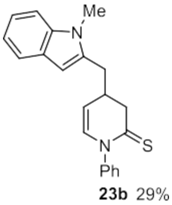

23b 29%

(4*RS*)-4-((1-Methyl-1*H*-indol-2-yl)methyl)-1-phenyl-3,4-dihydropyridine-2(1*H*)-thione (**23b**): Yellow solid, m.p. 156-158°C. ^{1}H NMR (400 MHz, $CDCl_3$) δ 2.85 – 3.00 (m, 3H, 4-CH_2, CH-4), 3.19 (dd, *J* = 16.7, 8.7 Hz, 1H, CHH-3), 3.34 – 3.46 (m, 1H, CHH-3), 3.71 (s, 3H, NCH_3), 5.61 (dd, *J* = 7.2, 2.3 Hz, 1H, =CH-5), 6.31 (dd, *J* = 7.7, 1.2 Hz, 1H, =CH-6), 6.36 (d, *J* = 0.8 Hz, 1H, ArH), 7.10 (ddd, *J* = 7.9, 7.0, 1.1 Hz, 1H, ArH), 7.16 – 7.33 (m, 4H, ArH), 7.33 – 7.43 (m, 1H, ArH), 7.43 – 7.50 (m, 2H, ArH), 7.56

(dt, *J* = 7.7, 1.0 Hz, 1H, ArH). ^{13}C NMR (101 MHz, $CDCl_3$) δ 29.78 (NCH_3), 30.86 (4-CH_2), 30.93 (CH-4), 47.55 (CH_2-3), 100.93, 109.02, 114.80, 119.56, 120.01, 121.08, 126.87 (2C), (ArH), 127.71 (Ar), 128.38, 129.60 (2C), 130.67 (ArH), 136.98, 137.49, 144.33 (Ar), 199.17 (C=O). GC-MS (EI 70Ev) m/z= 332 (15) [$M^{+\cdot}$], 188 (59), 186 (15), 145 (31), 144(100), 77(11). HRMS (ESI-TOF) m/z: [M + H]+ Calcd for $C_{21}H_{21}N_2S$ 333.1425; Found 333.1420.

(6*RS*)-6-((1-Methyl-1*H*-indol-2-yl)methyl)-1-phenyl-3,6-dihydropyridine-2(1*H*)-thione (**24b**): White solid. Mp. 172-176°C. ^{1}H NMR (400 MHz, $CDCl_3$) δ 3.02 (dd, *J* = 14.4, 9.7 Hz, 1H, 6-CHH), 3.31 (dd, *J* = 14.4, 3.7 Hz, 1H, 6-CHH), 3.39 (s, 3H, NCH_3), 3.51 – 3.63 (m, 1H, CHH-3), 3.88 (ddd, *J* = 22.4, 4.6, 2.7 Hz, 1H, CHH-3), 4.51 – 4.63 (m, 1H, CH-6), 5.90 (ddd, *J* = 10.2, 4.6, 2.0 Hz, 1H, =CH-4), 5.95 (ddd, *J* = 10.1, 4.0, 2.7 Hz, 1H, =CH-5), 6.32 (s, 1H, ArH), 7.08 (ddd, *J* = 7.9, 6.7, 1.3 Hz, 1H, ArH), 7.18 (ddd, *J* = 8.3, 6.8, 1.2 Hz, 1H, ArH), 7.23 (dd, *J* = 8.5, 1.1 Hz, 1H, ArH), 7.30 – 7.37 (m, 2H, ArH), 7.42 – 7.48 (m, 1H, ArH), 7.50 – 7.58 (m, 3H, ArH). ^{13}C NMR (101 MHz, $CDCl_3$) δ 29.42 (NCH_3), 31.66 (6-CH_2), 42.59 (CH_2-3), 63.98 (CH-6), 101.62, 109.06, 119.73, 120.17, 121.40 (ArH), 123.45 (=CH-4), 124.41 (=CH-5), 127.62 (Ar), 127.78 br (ArH), 128.47, 129.91 (2C), (ArH), 134.42, 137.37, 144.86 (Ar), 199.68 (C=S). GC-MS (EI 70Ev) m/z= 332 (63) [$M^{+\cdot}$], 331 (50), 207 (10), 183 (21), 182 (100), 181 (42), 167 (58), 77 (14). HRMS (ESI-TOF) m/z: $[M + H]^+$ Calcd for $C_{21}H_{21}N_2S$ 333.1425; Found 333.1421.

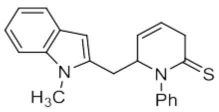

24b 30%

Procedure for the synthesis of compounds **25a-c** and **26a-c** (Scheme 9):

Compounds **25a-c** and **26a** were obtained following a meticulously performed procedure for compounds **23**/**24**. The preparation of lithiated heterocyclic reagents [(benzo[d]oxazol-2-ylmethyl)lithium and (benzo[d]thiazol-2-ylmethyl)lithium] involved a series of precise steps: To a cooled to -80°C solution of 2-methylbenzoxazole or 2-methylbenzothiazole (2.631 mmol) in anhydrous THF (10.9 mL) placed in Schlenk flask *n*-BuLi (1.1 mL, 2.5M, 1.05 eq.) was added. The reaction mixture was stirred at -80°C for 20 minutes under an argon atmosphere.

Note1. In the case of the synthesis of compounds **25a** and **26a** 0.3 g (1.754 mmol) of substrate **22a** was used, and despite stirring of benzo[d]oxazol-2-ylmethyl)lithium for 2.5 h at -80°C and 1 h at -50°C full conversion of the substrate was not achieved.

Note 2. In the case of the synthesis of compound **25b**, 0.15 g (0.801 mmol) of substrate 22b was used and the reaction between benzo[d]oxazol-2-ylmethyl)lithium and **22b** was stirred at -80°C for 3 hours.

(4*RS*)-4-(Benzo[d]oxazol-2-ylmethyl)-1-phenyl-3,4-dihydropyridin-2(1*H*)-one (**25a**): Crude product was purified by column chromatography (SiO_2, hexane : AcOEt 4 : 1) to afford 0.196 g (37% yield) of solid, m.p. 90-91°C. ^{1}H NMR (400 MHz, $CDCl_3$) δ 2.73 (dd, *J* = 16.0, 9.2 Hz, 1H, CHH-3), 2.93 (ddd, *J* = 16.0, 6.5, 0.8 Hz, 1H, CHH-3), 3.04 – 3.18 (m, 2H, 4-CH_2), 3.30 – 3.41 (m, 1H, CH-4), 5.32 (dd, *J* = 7.8, 3.9 Hz, 1H, =CH-4), 6.32 (dd, *J* = 7.8, 1.7 Hz, 1H, =CH-6), 7.18 – 7.24 (m, 2H, ArH), 7.25 – 7.36 (m, 3H, ArH), 7.36 –

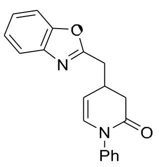

25a 37%

7.43 (m, 2H, ArH), 7.45 – 7.55 (m, 1H, ArH), 7.63 – 7.74 (m, 1H, ArH). ^{13}C NMR (101 MHz, $CDCl_3$) δ 30.72 (CH-4), 33.49 (4-CH_2), 38.10 (CH_2-3), 109.26 (=CH-4), 110.49, 119.79, 124.35, 124.88, 126.02 (2C), 127.19, 129.12 (2C), (ArH), 131.19 (=CH-6), 140.21, 141.22, 150.85, 164.33 (Ar), 167.93 (C=O). GC-MS (EI 70Ev) m/z= 304 (2) [M+], 173 (13), 172(100), 144 (10), 77 (14). HRMS (ESI-TOF) m/z: [M + H]+ Calcd for $C_{19}H_{17}N_2O_2$ 305.1290; Found 305.1289.

(6*RS*)-6-(Benzo[d]oxazol-2-ylmethyl)-1-phenyl-3,6-dihydropyridin-2(1*H*)-one (**26a**): Crude product

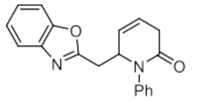

26a 22%

was purified by column chromatography (SiO_2, *n*-hexane : AcOEt 2 : 1) to afford 0.115 g (22% yield) of yellow oil. ^{1}H NMR (400 MHz, $CDCl_3$) δ 2.94 (ddt, *J* = 21.9, 3.7, 2.5 Hz, 1H, CHH-3), 3.05 (dt, *J* = 21.9, 3.6 Hz, 1H, CHH-3), 3.13 (dd, *J* = 15.1, 6.7 Hz, 1H, 6-CHH), 3.19 (dd, *J* = 15.1, 4.5 Hz, 1H, 6-CHH), 4.87 – 4.98 (m, 1H, CH-6), 5.83 – 5.90 (m, 1H, =CH-4), 5.91 – 5.98 (m, 1H, =CH-5), 7.28 – 7.53 (m, 8H, ArH), 7.63 – 7.70 (m, 1H, ArH). ^{13}C NMR (101 MHz, $CDCl_3$) δ 32.62 (CH_2-3), 33.52 (6-CH_2), 59.94 (CH-6), 110.43, 119.88, 124.11, 124.27, 124.44, 125.00, 127.82 (ArH), 128.21 (2C), 129.59 (2C, ArH) 140.35, 141.10, 150.72, 162.63 (Ar), 167.77 (C=O). GC-MS (EI 70Ev) m/z= 304 (<1) [$M^{+\cdot}$], 173 (37), 172(100), 144 (10), 143 (10), 133 (10), 104 (14), 77 (18). HRMS (ESI-TOF) m/z: [M + H]+ Calcd for $C_{19}H_{17}N_2O_2$ 305.1290; Found 305.1286.

(4*RS*)-4-(Benzo[d]oxazol-2-ylmethyl)-1-phenyl-3,4-dihydropyridine-2(1*H*)-thione (**25b**): Crude

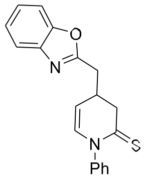

25b 70%

product was purified by column chromatography (SiO_2, hexane : AcOEt 4 : 1) to afford 0.18 g (70% yield) of brown oil. ^{1}H NMR (400 MHz, $CDCl_3$) δ 2.96 – 3.15 (m, 2H, 4-CH_2), 3.16 – 3.31 (m, 2H, CHH-3, CH-4), 3.38 – 3.51 (m, 1H, CHH-3), 5.64 (dd, *J* = 7.7, 3.5 Hz, 1H, =CH-5), 6.33 (dd, *J* = 7.6, 1.4 Hz, 1H, =CH-6), 7.20 – 7.25 (m, 2H, ArH), 7.29 – 7.40 (m, 3H, ArH), 7.42 – 7.48 (m, 2H, ArH), 7.47 – 7.55 (m, 1H, ArH), 7.66 – 7.73 (m, 1H, ArH). ^{13}C NMR (101 MHz, $CDCl_3$) δ 29.54 (CH-4), 32.76 (4-CH_2), 47.28 (CH_2-3), 110.51 (ArH), 113.68 (=CH-5), 119.77, 124.34, 124.88 (ArH), 126.86 (2C), 128.39, 129.57 (2C), (PhH), 131.08 (=CH-6), 141.16, 144.21, 150.82, 164.12 (Ar), 198.62 (C=S). GC-MS (EI 70Ev): m/z= 320 (<1) [$M^{+\cdot}$], 188 (100), 186 (41), 133 (23), 77 (14). HRMS (ESI-TOF) m/z: $[M + H]^+$ Calcd for $C_{19}H_{17}N_2OS$ 321.1062; Found 321.1057.

(4*RS*)-4-(Benzo[*d*]thiazol-2-ylmethyl)-1-phenyl-3,4-dihydropyridin-2(1*H*)-one (**25c**): Crude product

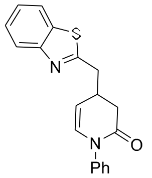

25c 28%

was purified by column chromatography (SiO_2, hexane : AcOEt 4 : 1→ 1:1) to afford 0.154 g (28% yield) of solid, m.p. 108-120°C. ^{1}H NMR (400 MHz, $CDCl_3$) δ 2.71 (dd, *J* = 16.1, 8.5 Hz, 1H, CHH-3), 2.90 (dd, *J* = 16.1, 6.1 Hz, 1H, CHH-3), 3.17 – 3.38 (m, 3H, CH-4, 4-CH_2), 5.32 (dd, *J* = 7.9, 3.3 Hz, 1H, =CH_2-5), 6.32 (dd, *J* = 7.8, 1.4 Hz, 1H, =CH-6), 7.21 – 7.31 (m, 3H, ArH), 7.35 – 7.43 (m, 3H, ArH) 7.49 (ddd, *J* = 8.3, 7.2, 1.3 Hz, 1H, ArH), 7.87 (dt, *J* = 8.0, 0.9 Hz, 1H, ArH), 8.01 (dt, *J* = 8.1, 0.9 Hz, 1H, ArH). ^{13}C NMR (101 MHz, $CDCl_3$) δ 32.65 (4-CH), 38.05 (CH_2-3), 38.78 (4-CH_2), 109.52, 121.53, 122.81, 125.04, 125.98 (2C),

126.12, 127.14, 129.10 (2C), 131.04 (ArH), 135.19, 140.22, 153.28, 168.01 (Ar), 168.07 (C=O). GC-MS (EI 70Ev): m/z= 320 (>1) [M+], 173 (14), 172 (100), 149 (21), 77(15). HRMS (ESI-TOF) m/z: [M + H]+ Calcd for $C_{19}H_{17}N_2OS$ 321.1062; Found 321.1059.

Procedure for the synthesis of compound **29** (Scheme 10):

To a stirred solution of *N*-methyl-3-oxo-3-phenylpropanethioamide (**28**) (0.134 g, 0.7 mmol) in toluene (0.8 mL) in a round bottom open flask, (*E*)-2,3-diphenylacrylaldehyde (**27**) (0.21 g, 1.0 mmol) and catalyst **30** (0.022 g, 0.07 mmol) were added. The solution was stirred for 1 hour at room temperature, and then benzoic acid (0.84g, 0.69 mmol) was added. The reaction was completed after one day (NMR analyses), and a then saturated, aqueous solution of $NaHCO_3$ (1 mL) was added and the aqueous layer was extracted with chloroform (3 x 10 mL). The combined organic layers were dried with Na_2SO_4 and filtered, and the solvents were evaporated under reduced pressure. The crude product was used in the next stage. The solution of TFA (0.157 g, 1.38 mmol) in DCM (5 mL) prepared in a Schlenk flask under argon was transferred in portions with a syringe to a stirred solution of crude product dissolved in DMF (1.5 mL) in another Schlenk flask. The resulting solution was stirred for 18 hours at room temperature, then an aqueous solution of $NaHCO_3$ (1 mL) was added, and the aqueous layer was extracted with chloroform (3 x 50 mL). The combined organic layers were washed with saturated NaCl (aq.) and dried with Na_2SO_4. The mixture was filtered, and the solvents were evaporated under reduced pressure. The crude product was purified by column chromatography on (SiO_2, *n*-hexane : ethyl acetate, 15 : 1) to afford 0.11 g (43% yield) of the yellow solid.

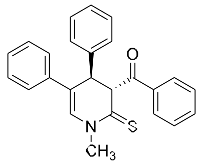

29 43%

((3*R*,4*R*)-1-Methyl-4,5-diphenyl-2-thioxo-1,2,3,4-tetrahydropyridin-3-yl)(phenyl)methanone (**29**): Yellow solid, m.p. 119-120°C. ^{1}H NMR (400 MHz, $CDCl_3$) δ 3.77 (s, 3H, NCH_3), 4.13 (d, *J* = 1.5 Hz, 1H, 3-CH), 5.40 (d, *J* = 1.5 Hz, 1H, CH-4), 6.87 (s, 1H, =CH-6), 7.08 – 7.21 (m, 5H, ArH), 7.22 – 7.31 (m, 3H, ArH), 7.31 – 7.40 (m, 2H, ArH), 7.51 (dd, *J* = 8.3, 6.9 Hz, 2H, ArH), 7.55 – 7.67 (m, 1H, ArH), 8.01 – 8.08 (m, 2H, ArH). ^{13}C NMR (101 MHz, $CDCl_3$) δ 43.00 (NCH_3), 44.32 (3-CH), 65.31 (CH-4), 123.03 (=C-5), 125.26 (2C), 126.95 (2C), 127.61, 127.84 (ArH), 127.94 (=CH-6), 128.68 (2C), 128.98 (4C), 129.49 (2C), 133.64 (ArH), 134.74, 136.89, 139.87 (Ar), 190.82 (C=O), 194.65 (C=S). GC-MS (EI, 70eV): *m/z* 383 (3) [M^+], 280 (7), 279 (21), 278 (100), 276 (2), 246 (12), 228 (20), 202 (5), 147 (2), 115 (5), 105 (41), 106 (3), 77 (31). HRMS (ESI-TOF) m/z: [M + H]+ Calcd for $C_{25}H_{22}NOS$ 384.1422; Found 384.1416. $[\alpha]^{20}_D$ = -52.58 (MeOH). The absolute configuration was estimated on the basis of earlier experiments.[17] Enantiomeric excess (*ee*) was estimated for 20%.

^{1}H and ^{13}C NMR spectra

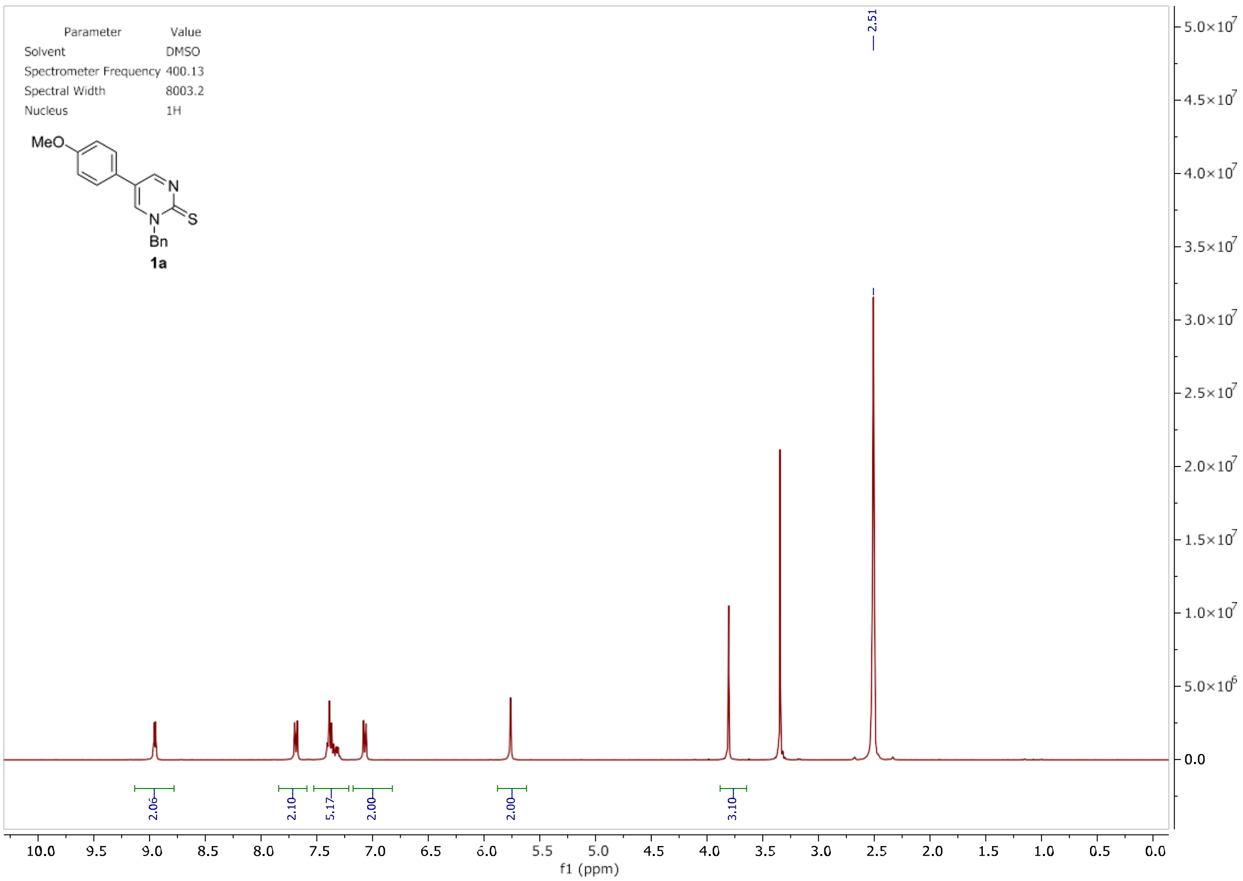

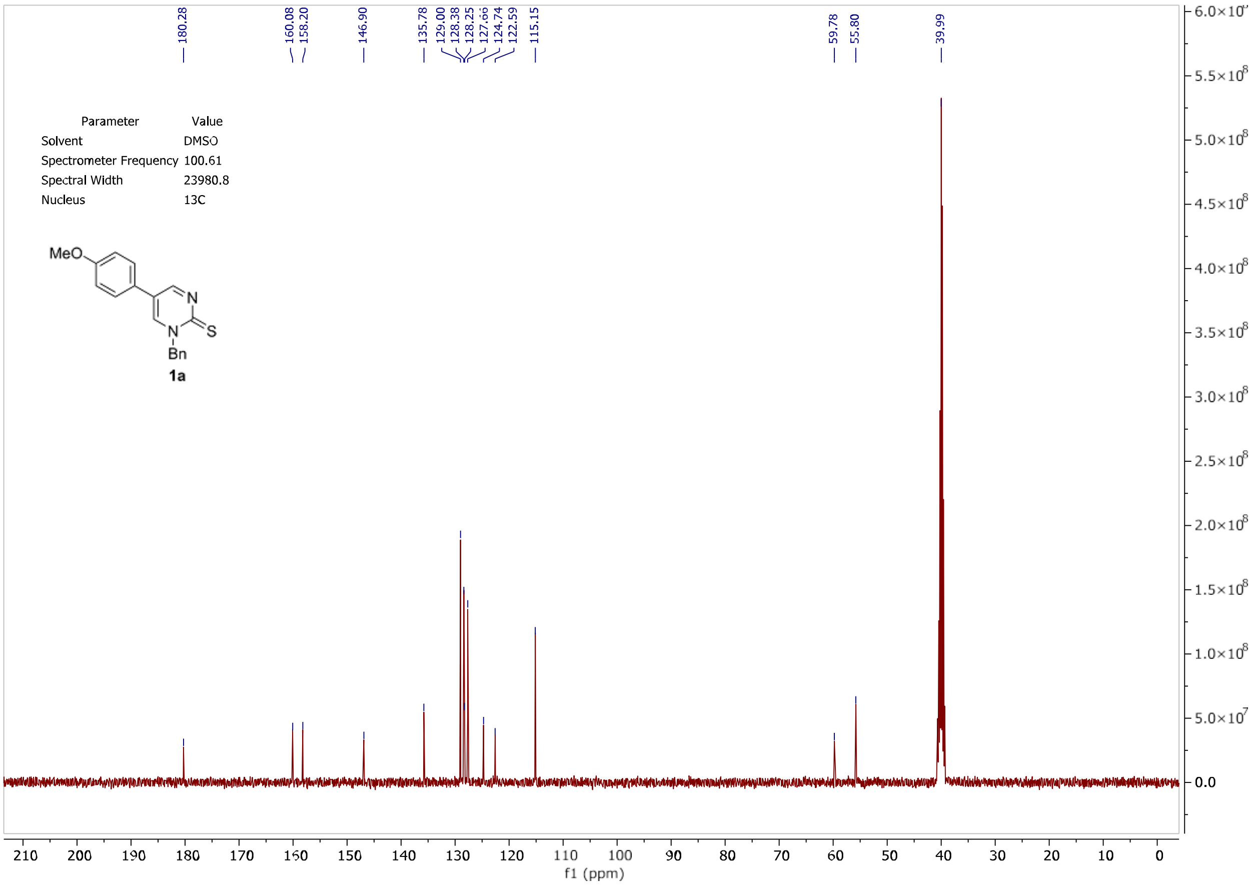

Figure S2.1. ^{1}H NMR (top) and ^{13}C NMR (bottom) spectra of compound **1a**

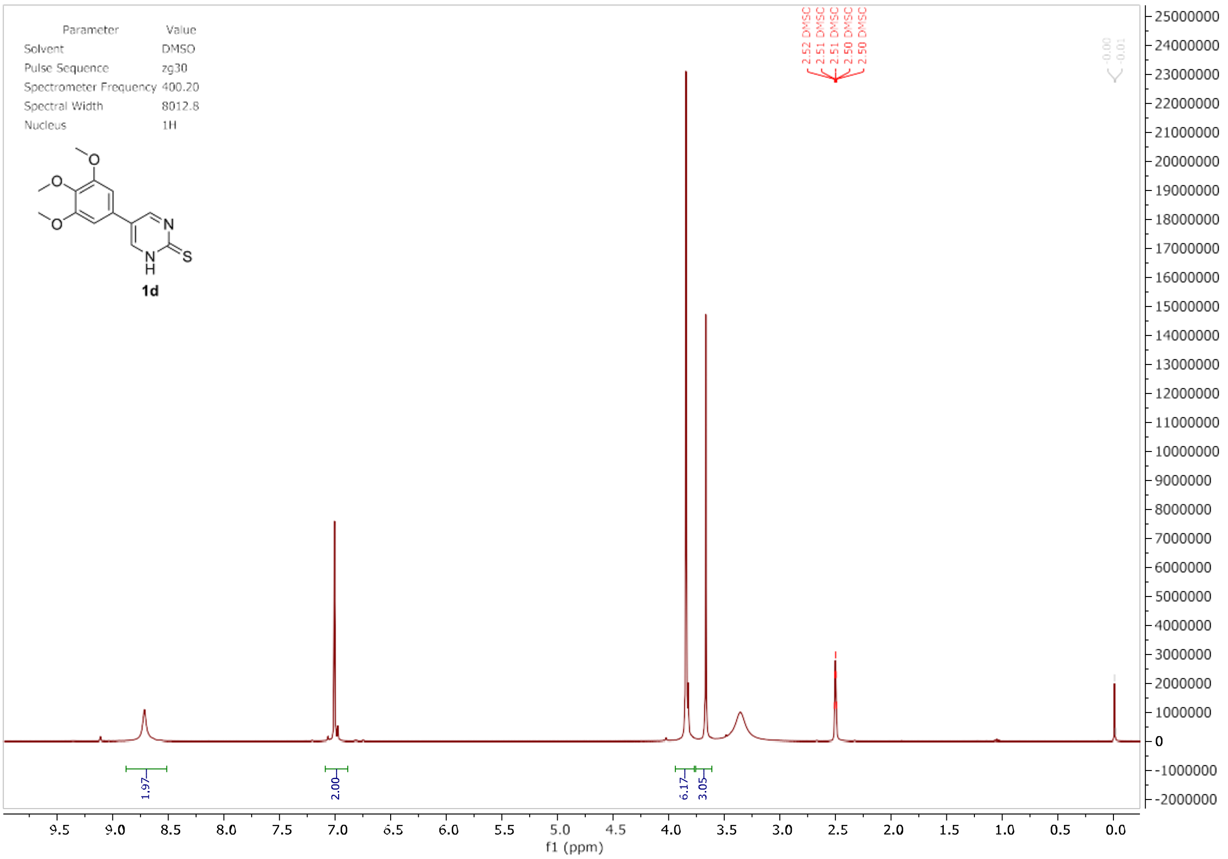

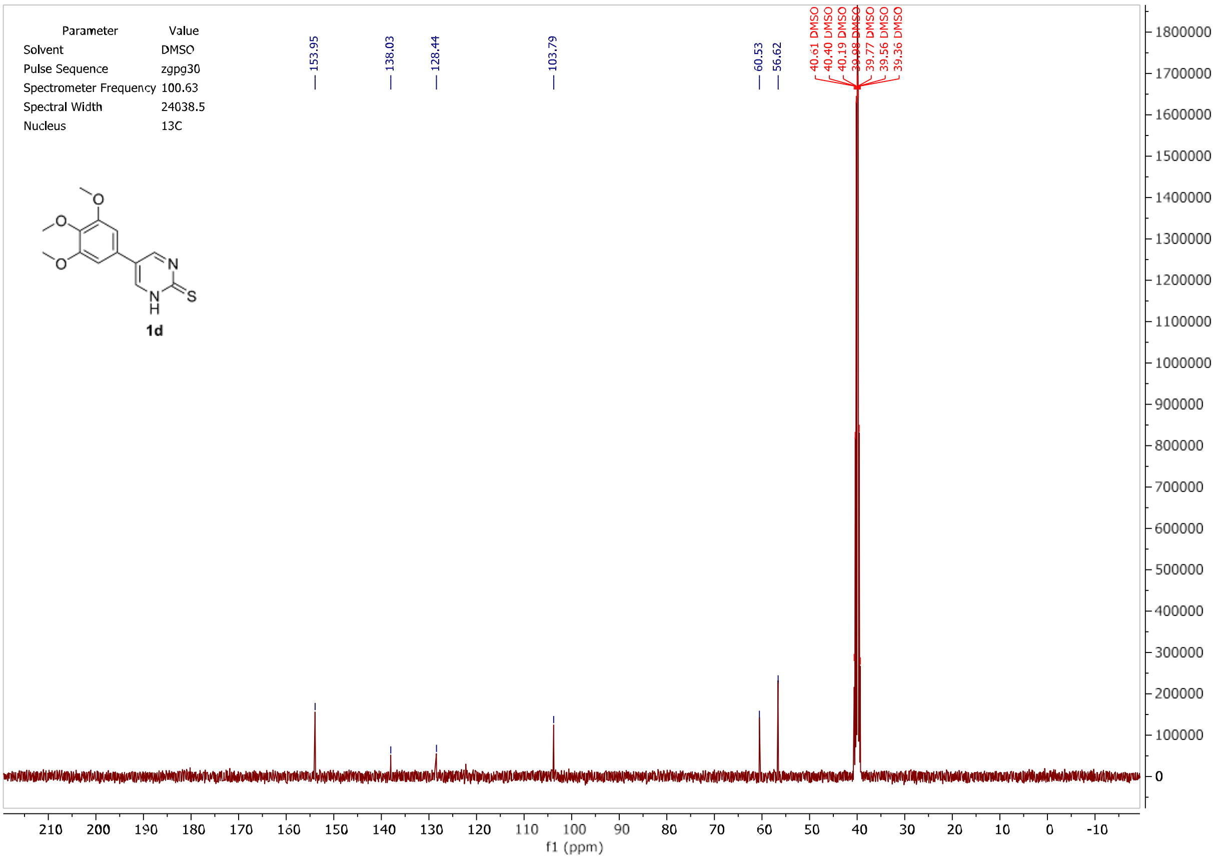

Figure S2.2. ^{1}H NMR (top) and ^{13}C NMR (bottom) spectra of compound **1d**

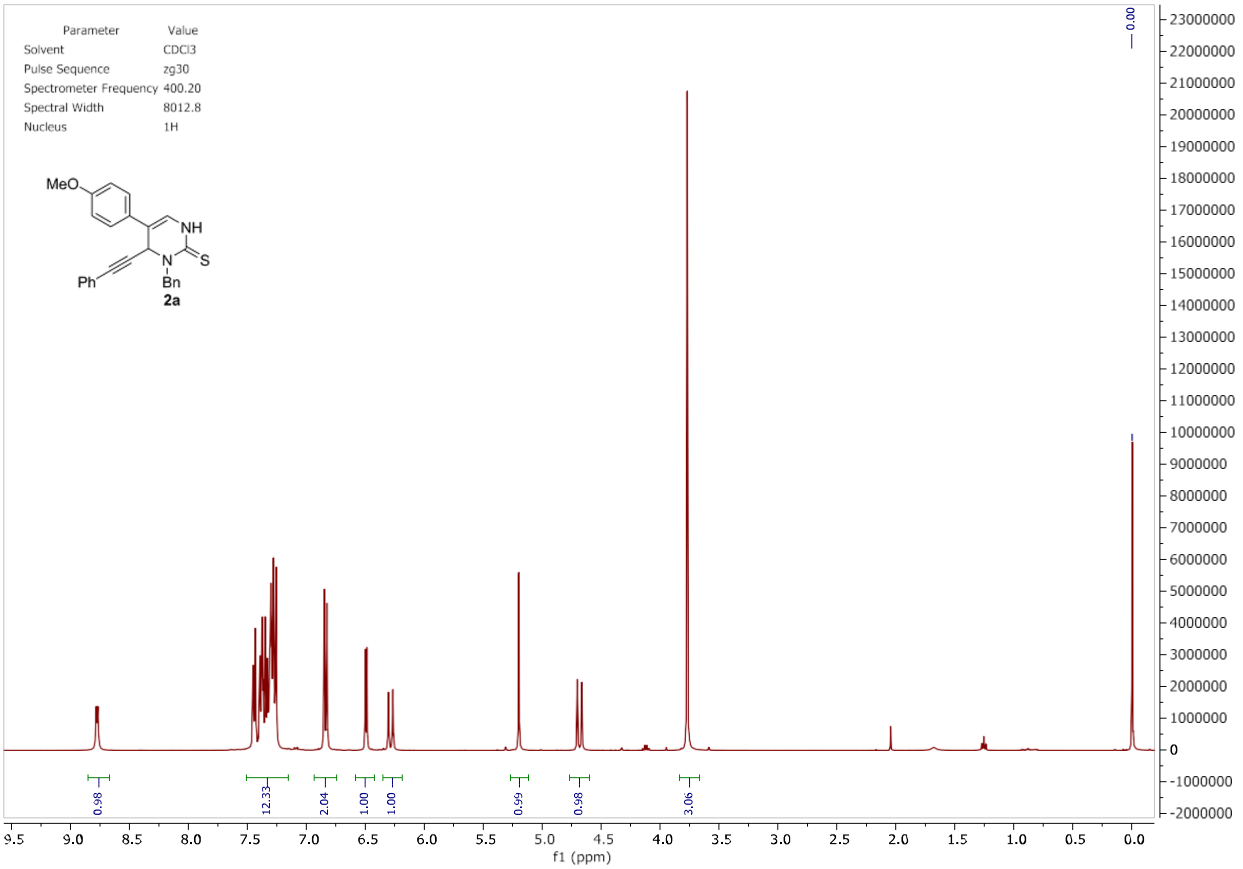

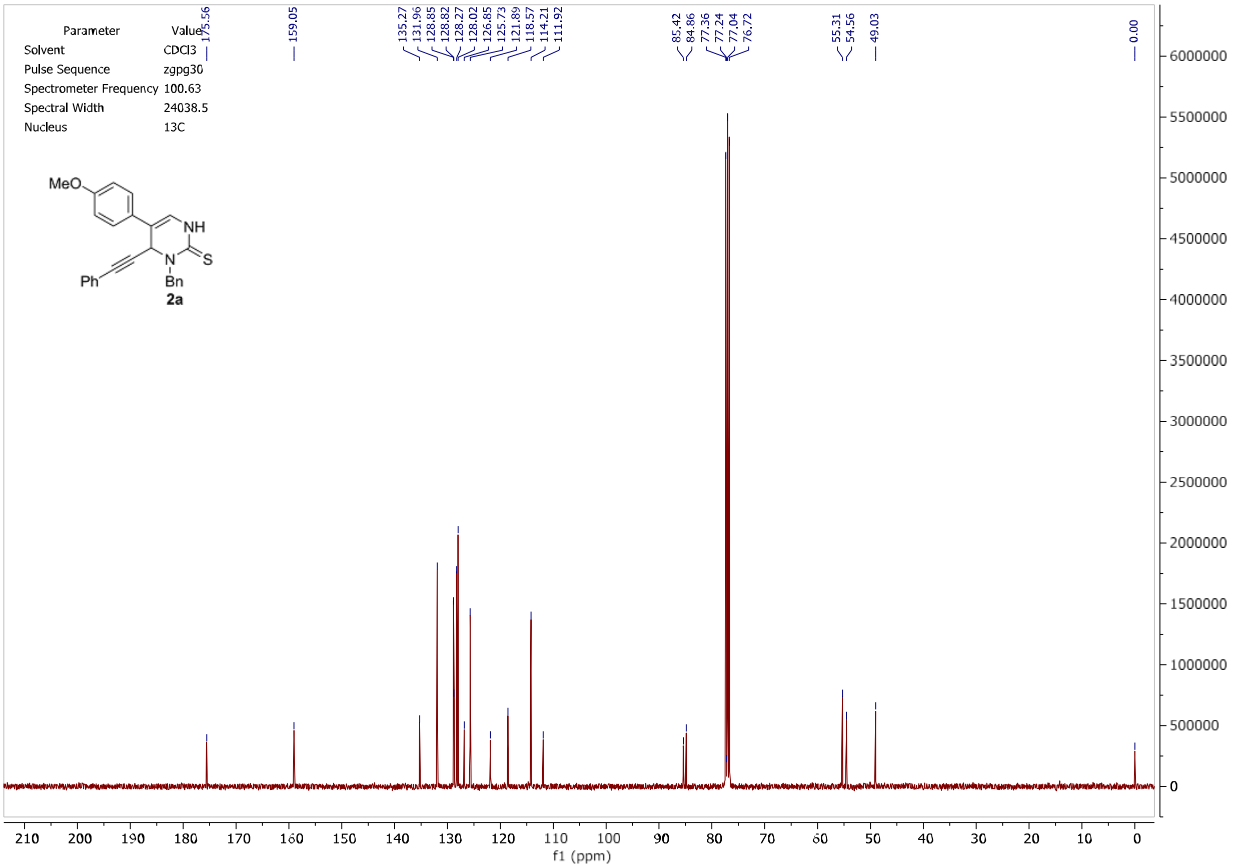

Figure S2.3. ^{1}H NMR (top) and ^{13}C NMR (bottom) spectra of compound **2a**

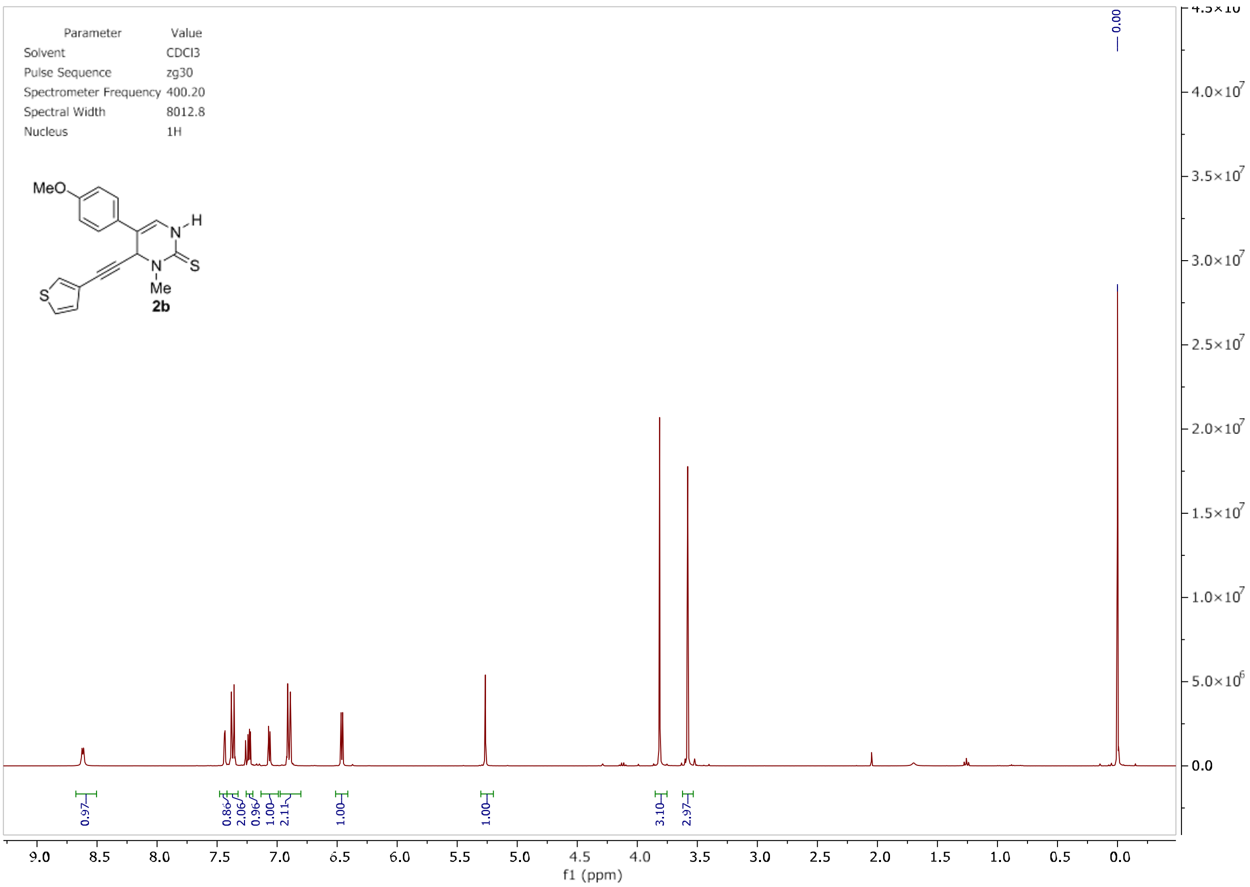

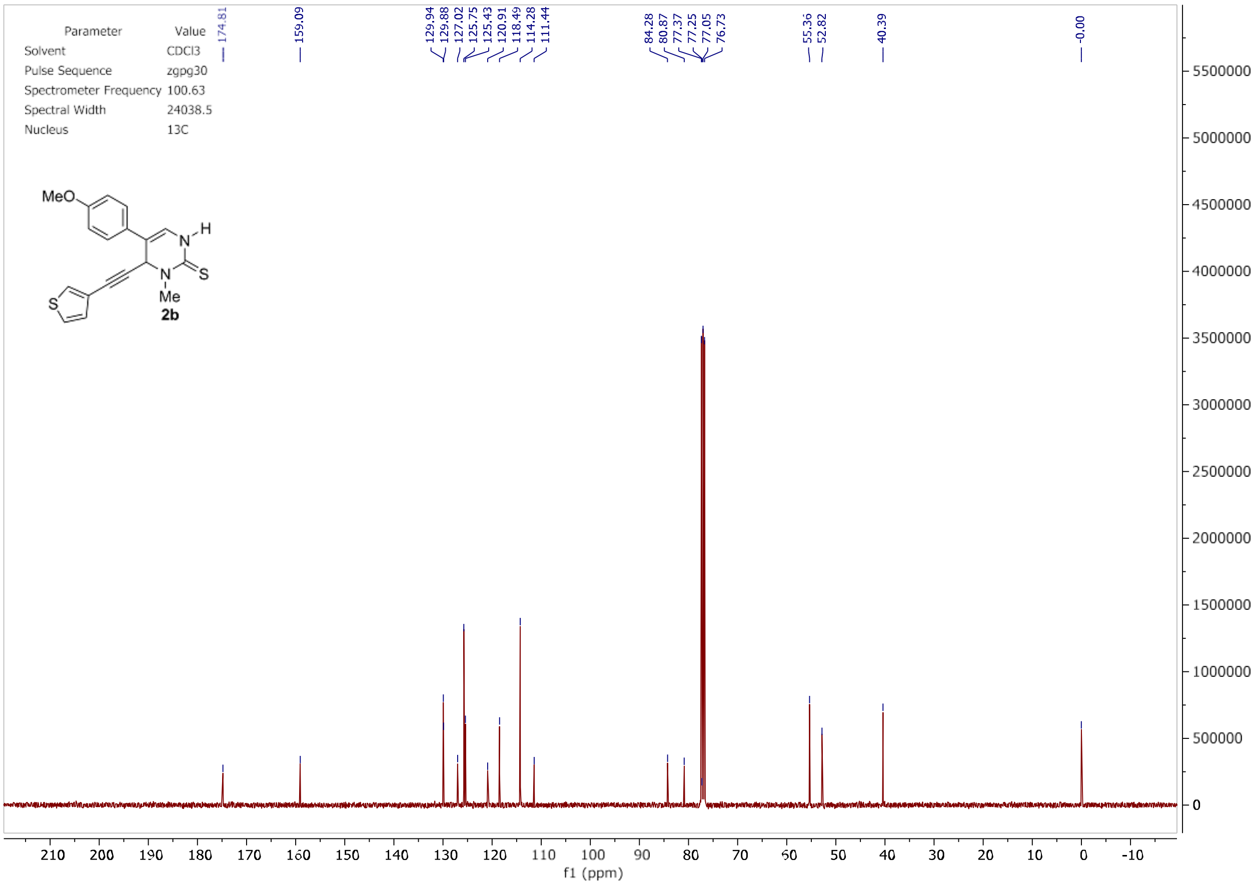

Figure S2.4. ^{1}H NMR (top) and ^{13}C NMR (bottom) spectra of compound **2b**

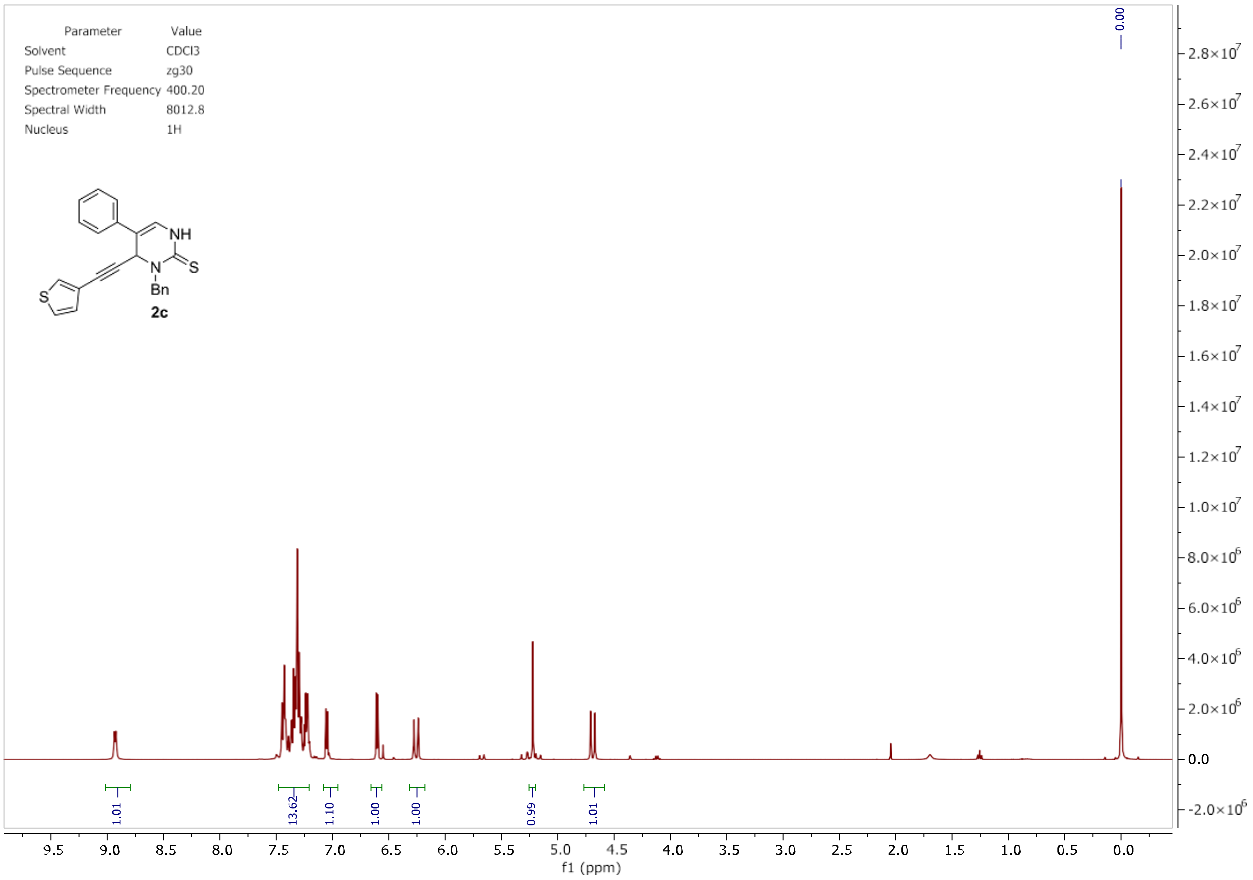

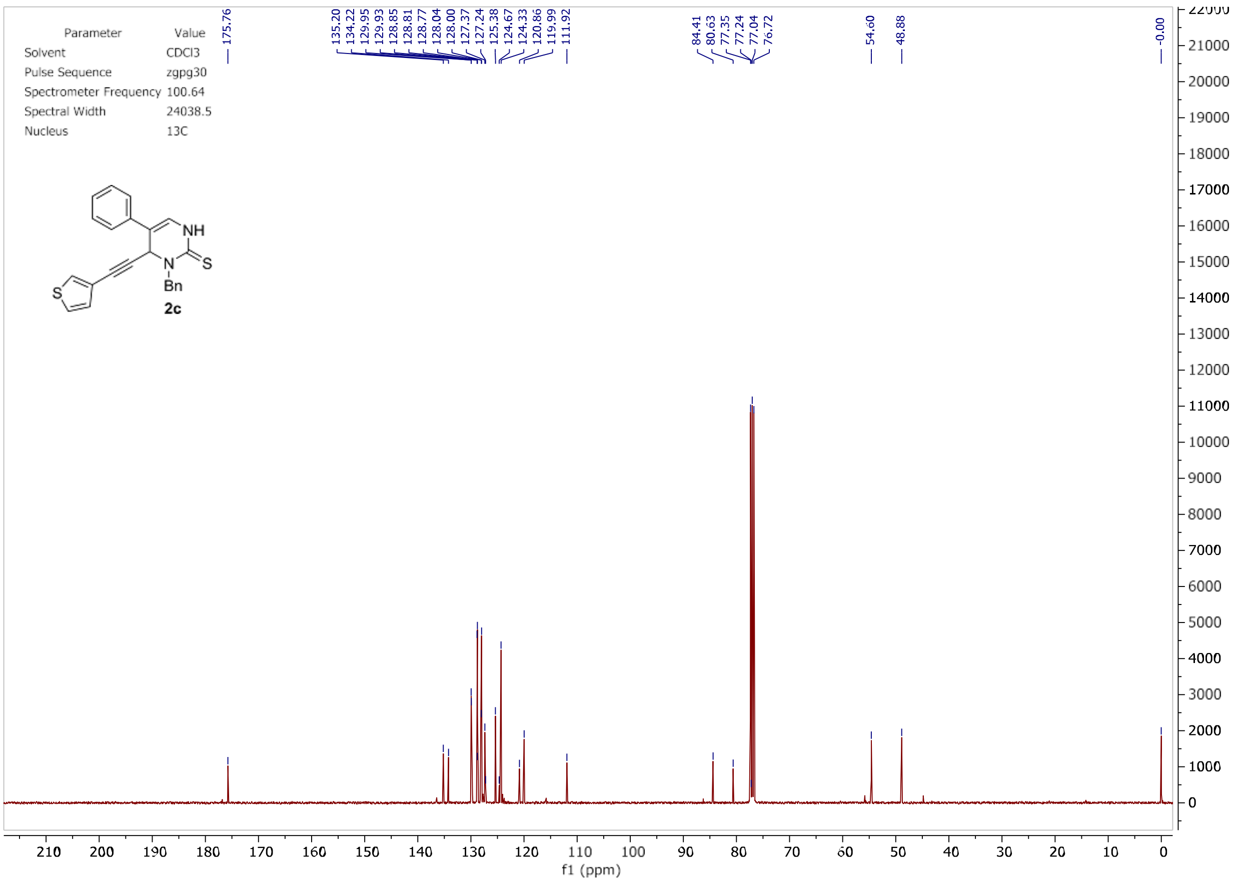

Figure S2.5. ^{1}H NMR (top) and ^{13}C NMR (bottom) spectra of compound **2c**

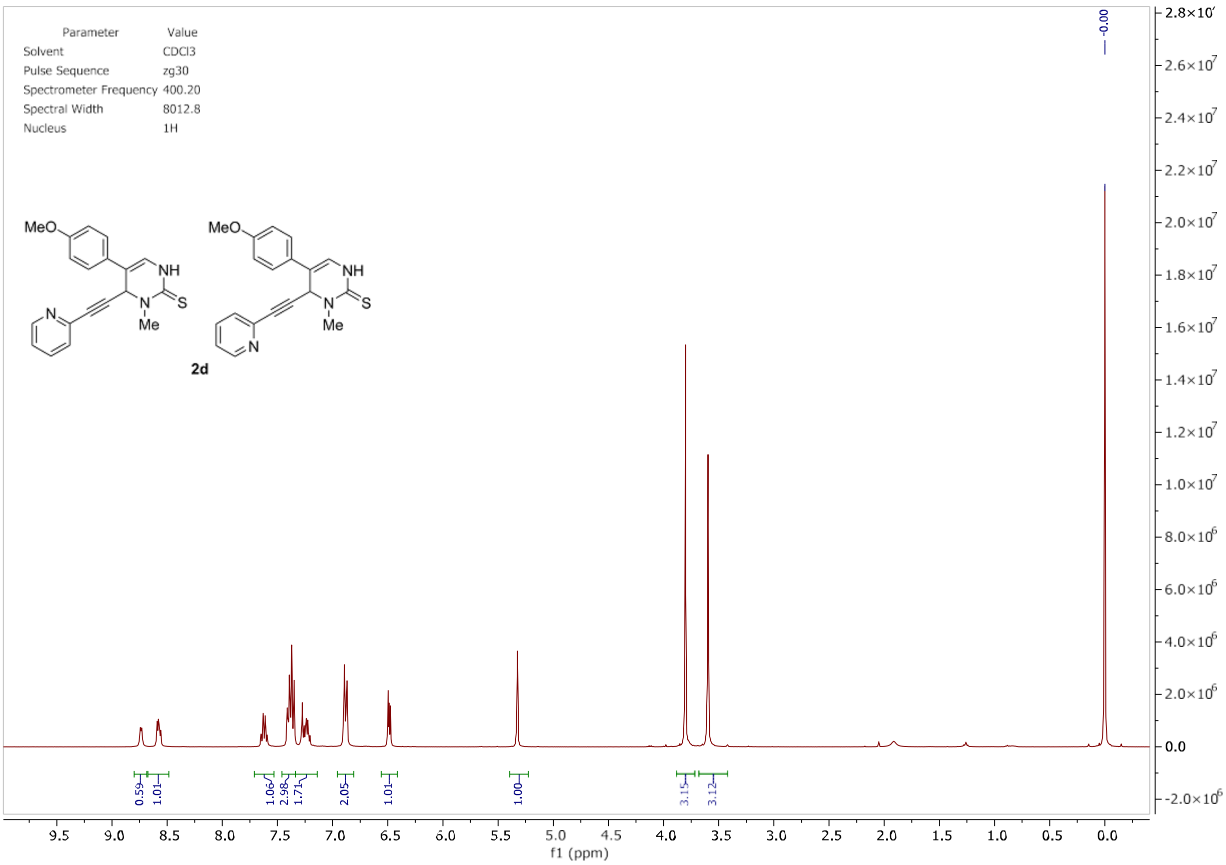

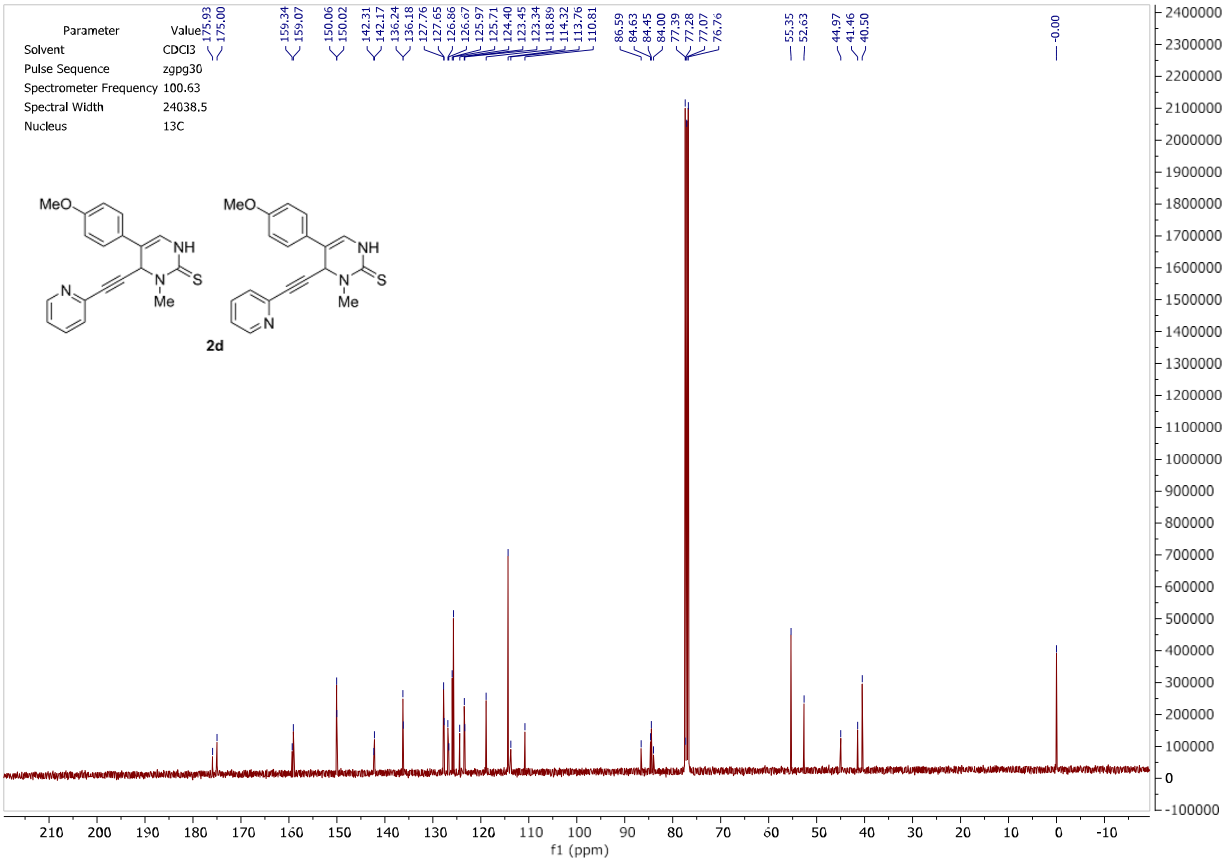

Figure S2.6. ^{1}H NMR (top) and ^{13}C NMR (bottom) spectra of compound **2d**

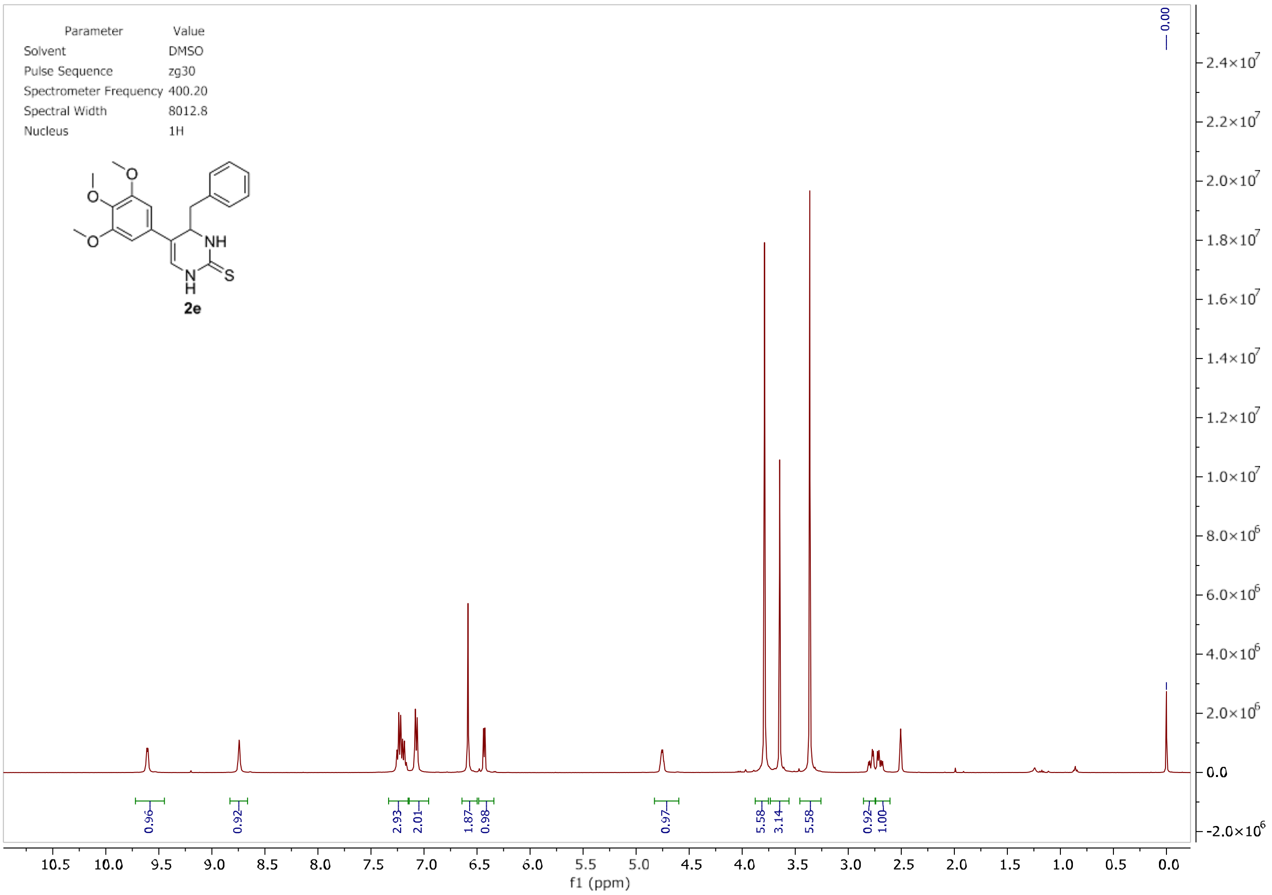

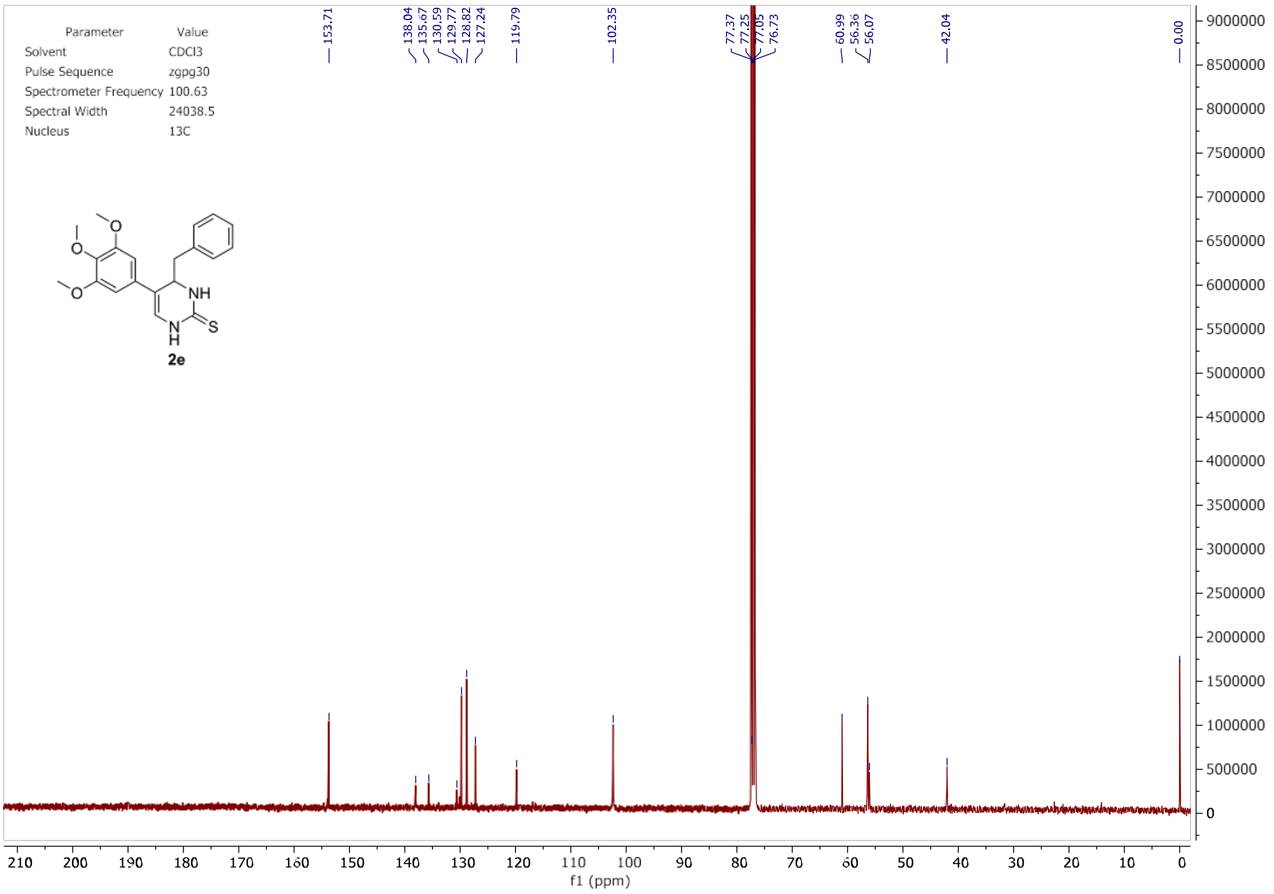

Figure S2.7. ^{1}H NMR (top) and ^{13}C NMR (bottom) spectra of compound **2e**

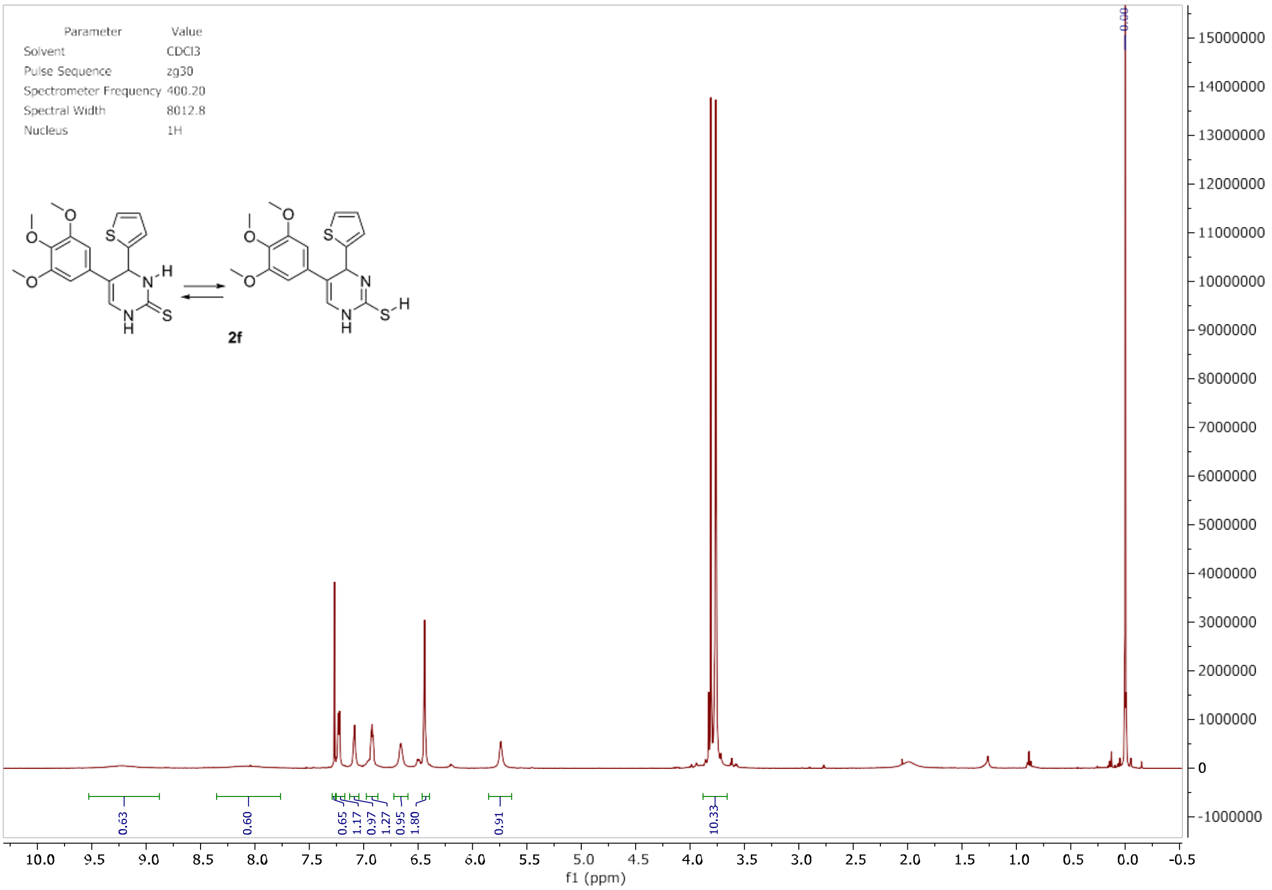

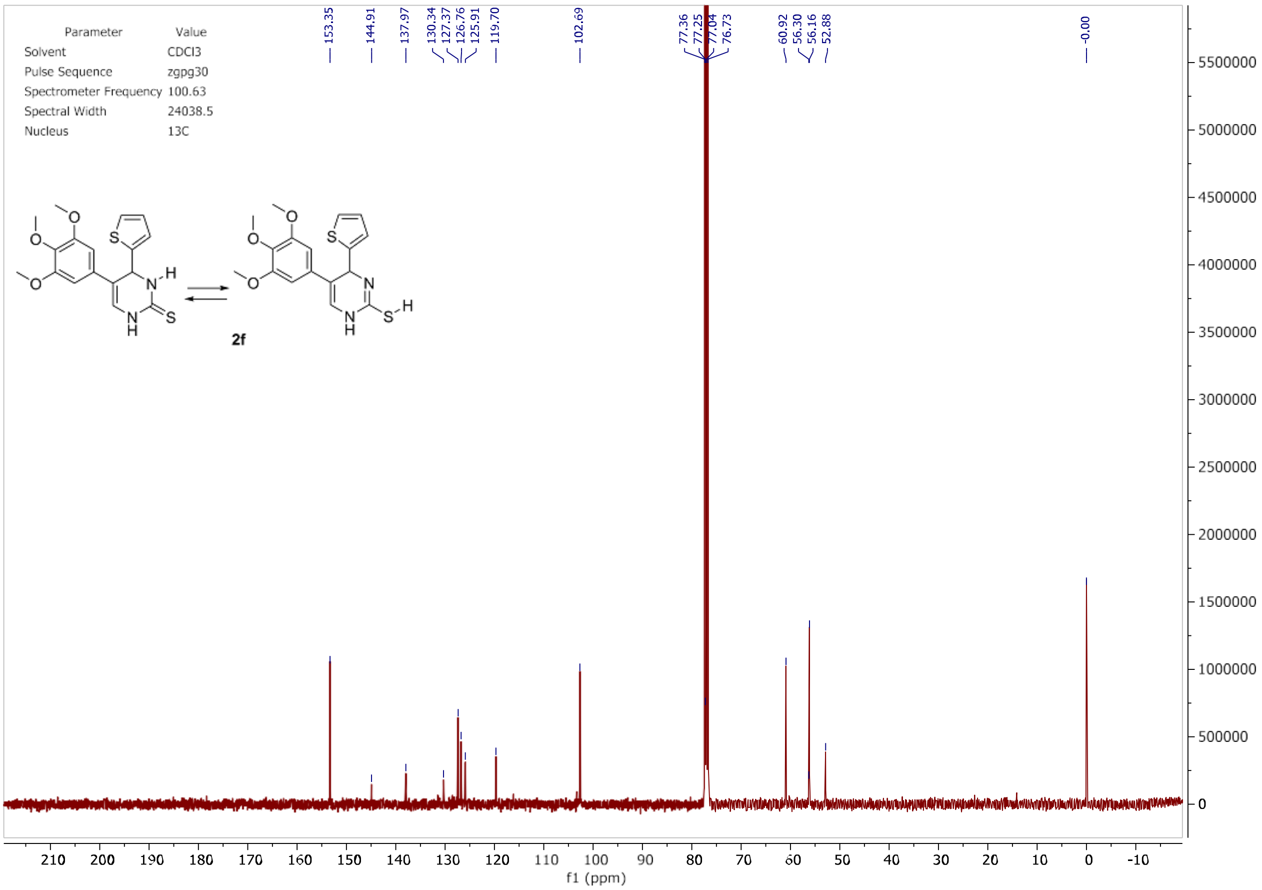

Figure S2.8. ^{1}H NMR (top) and ^{13}C NMR (bottom) spectra of compound **2f**

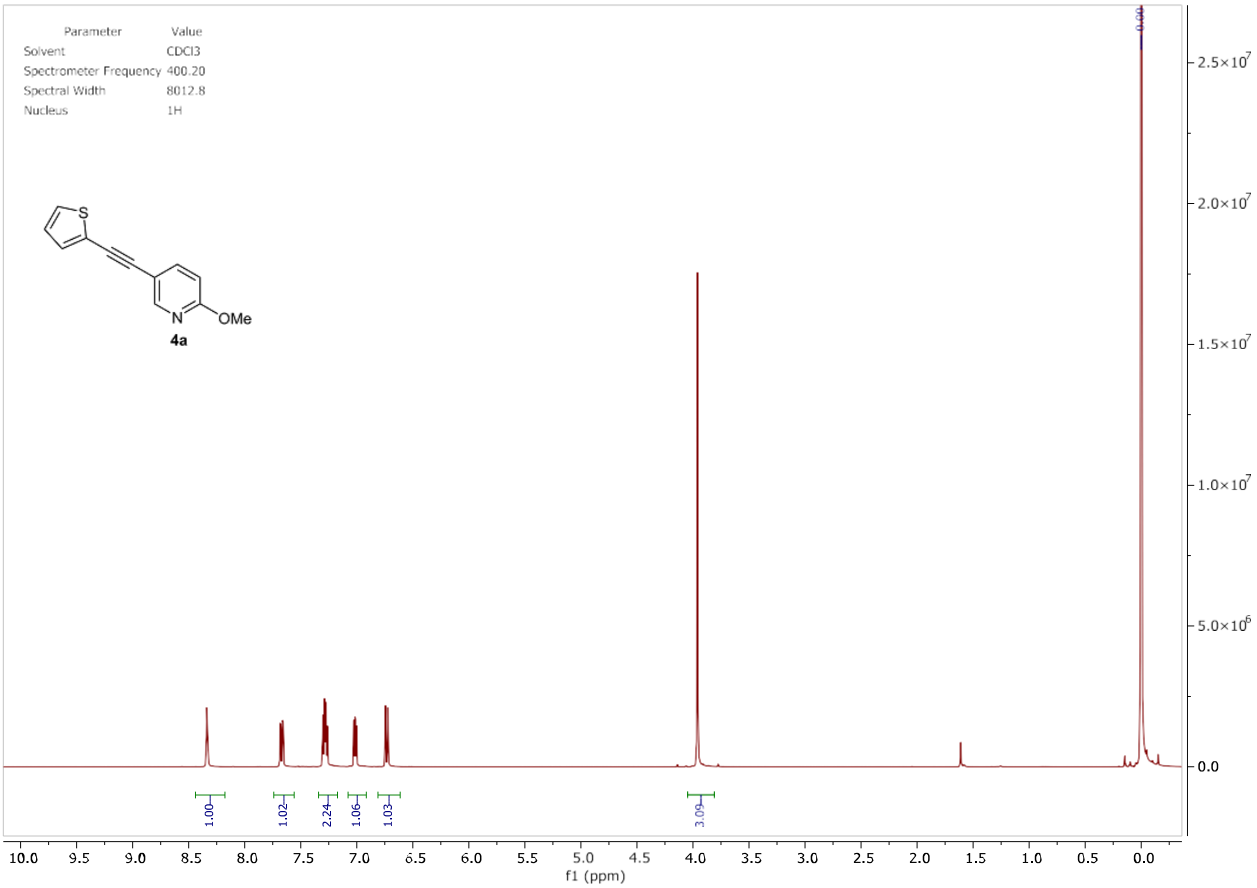

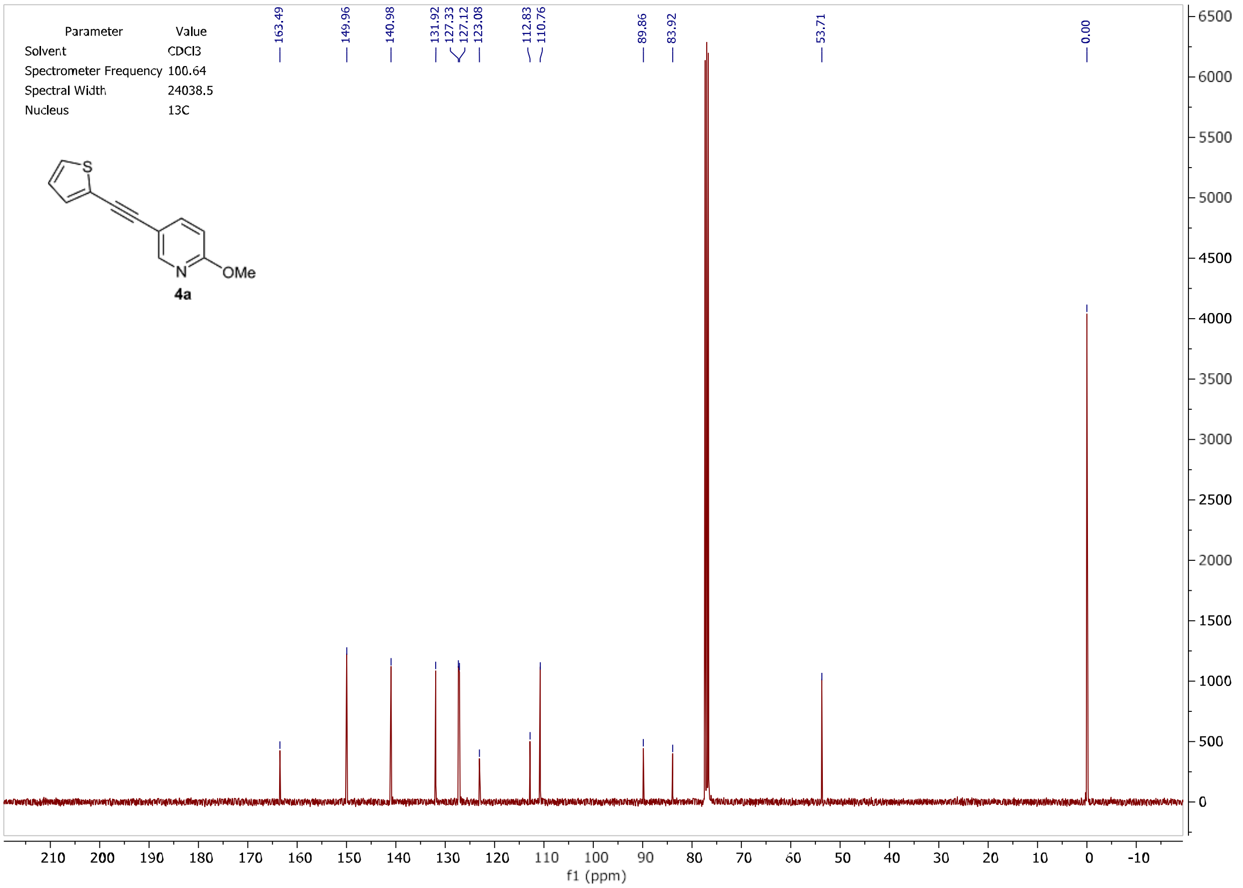

Figure S2.9. ^{1}H NMR (top) and ^{13}C NMR (bottom) spectra of compound **4a**

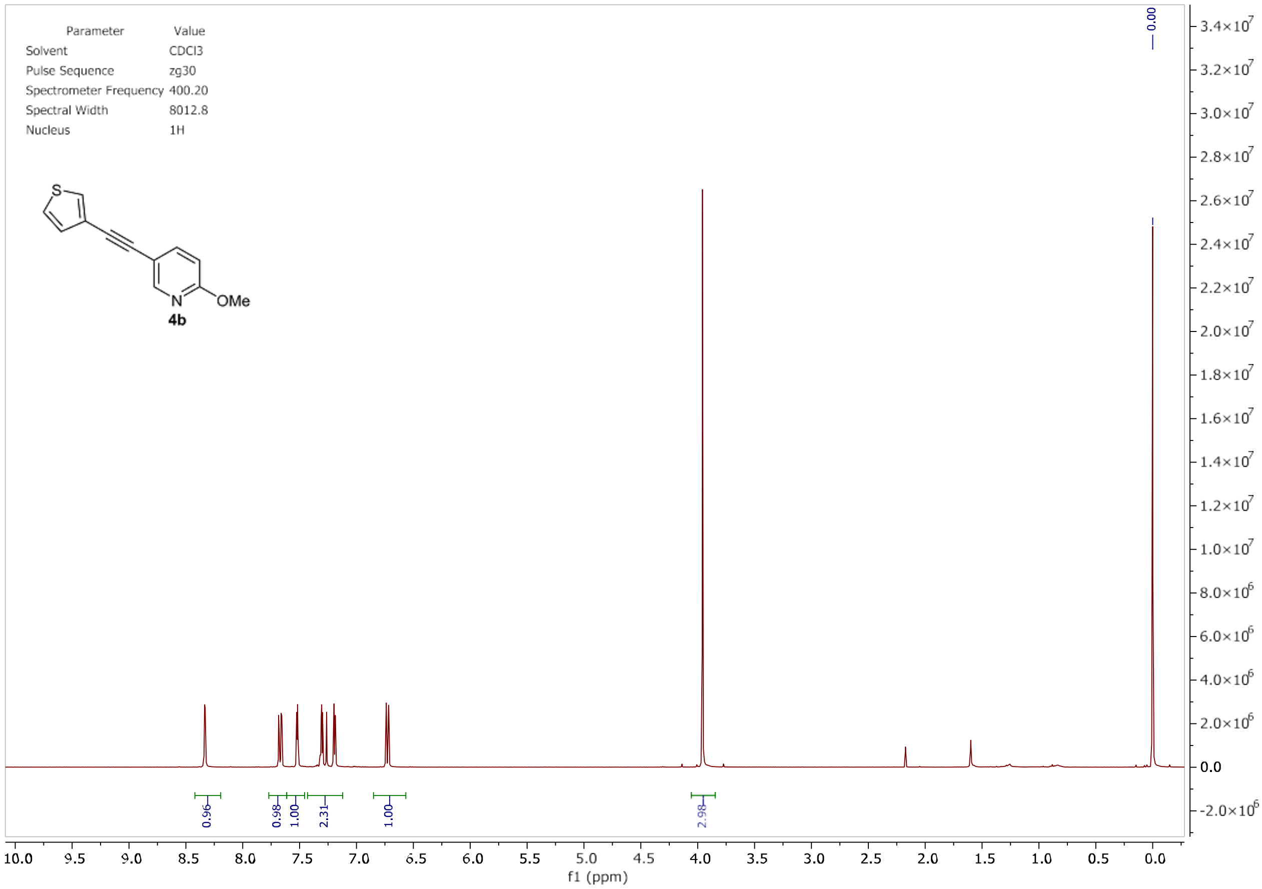

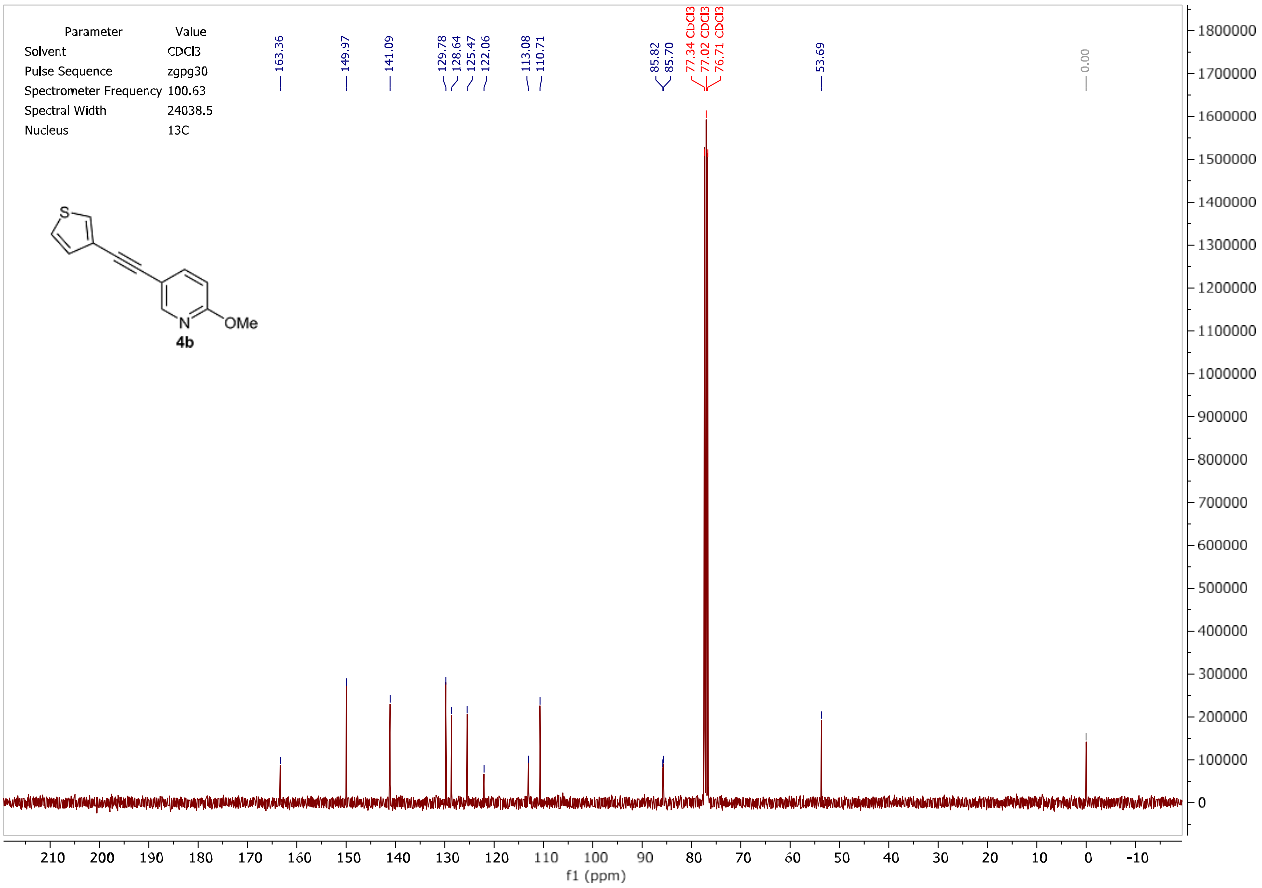

Figure S2.10. ^{1}H NMR (top) and ^{13}C NMR (bottom) spectra of compound **4b**

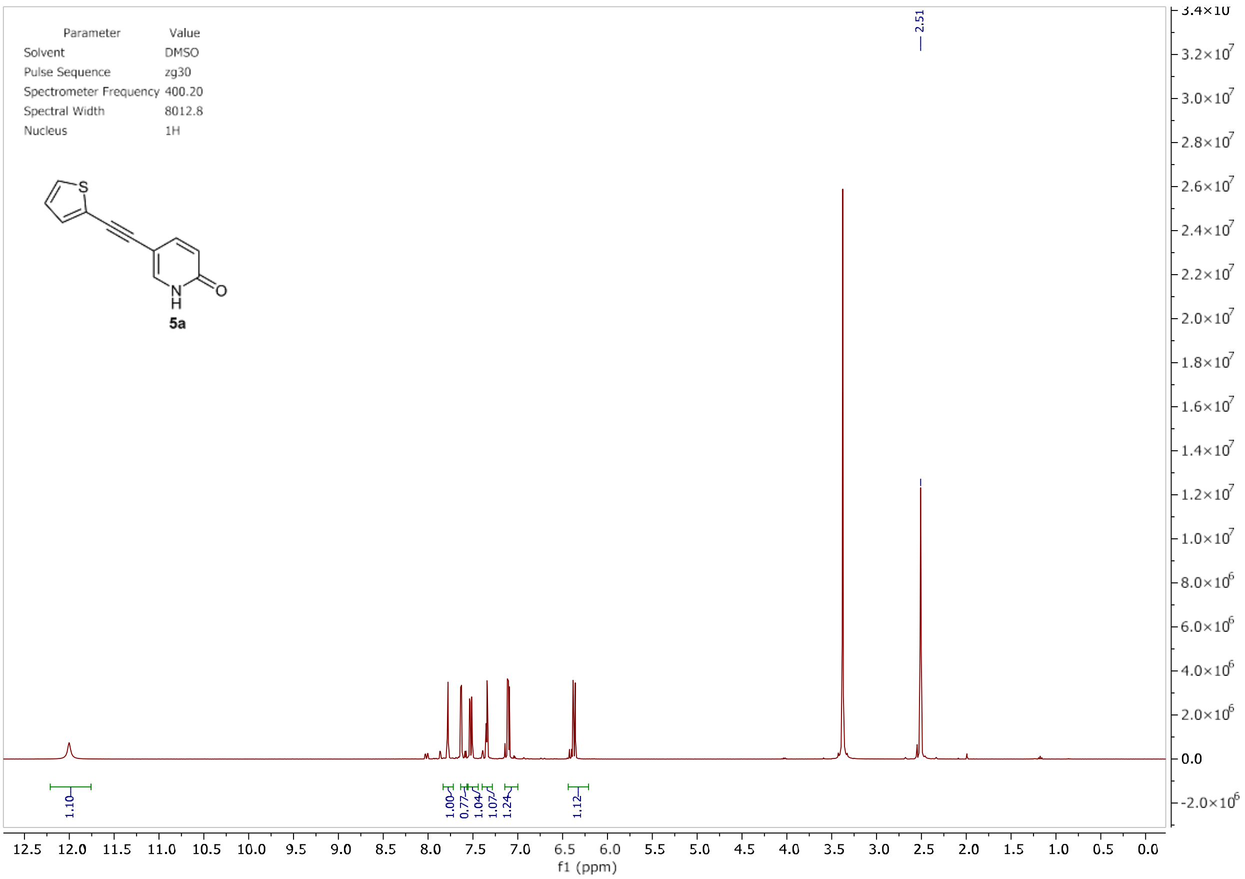

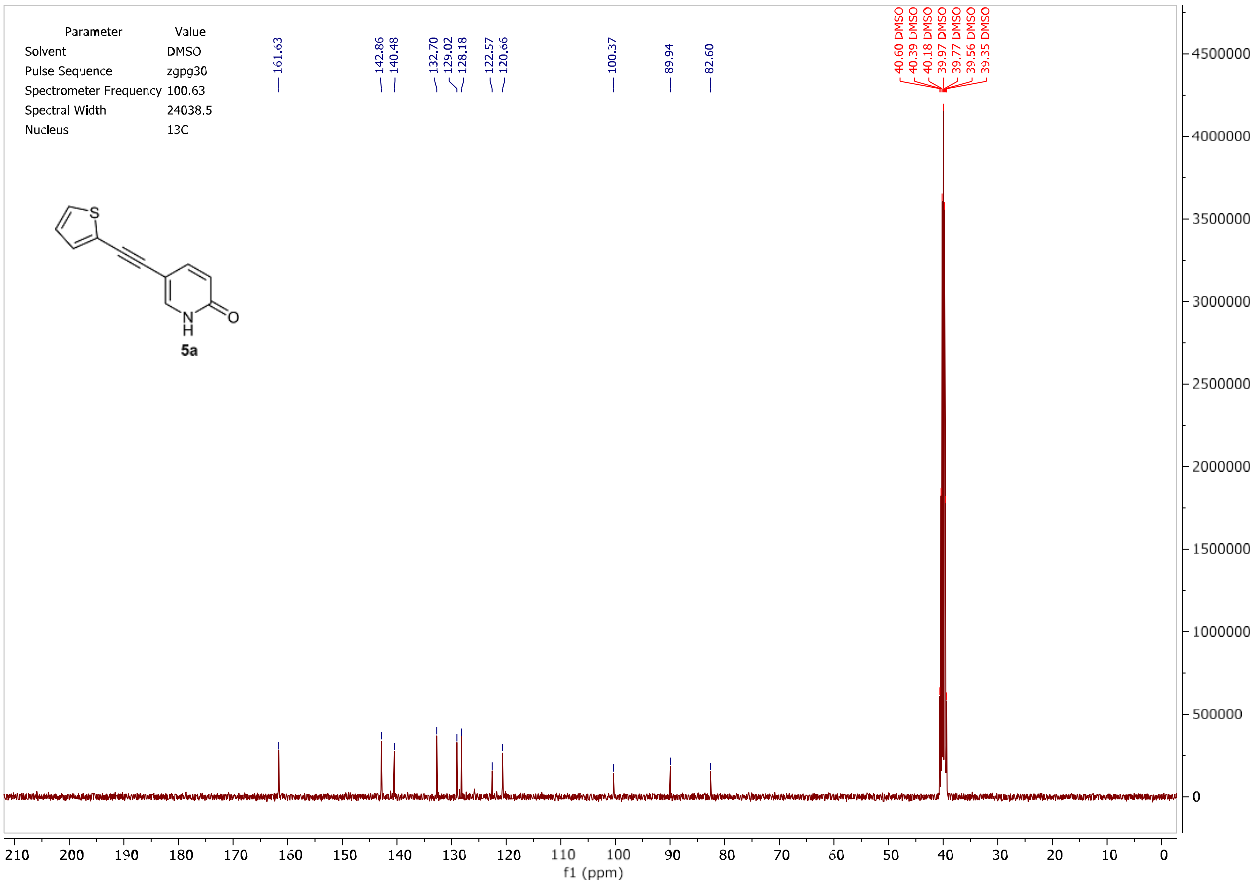

Figure S2.11. ^{1}H NMR (top) and ^{13}C NMR (bottom) spectra of compound **5a**

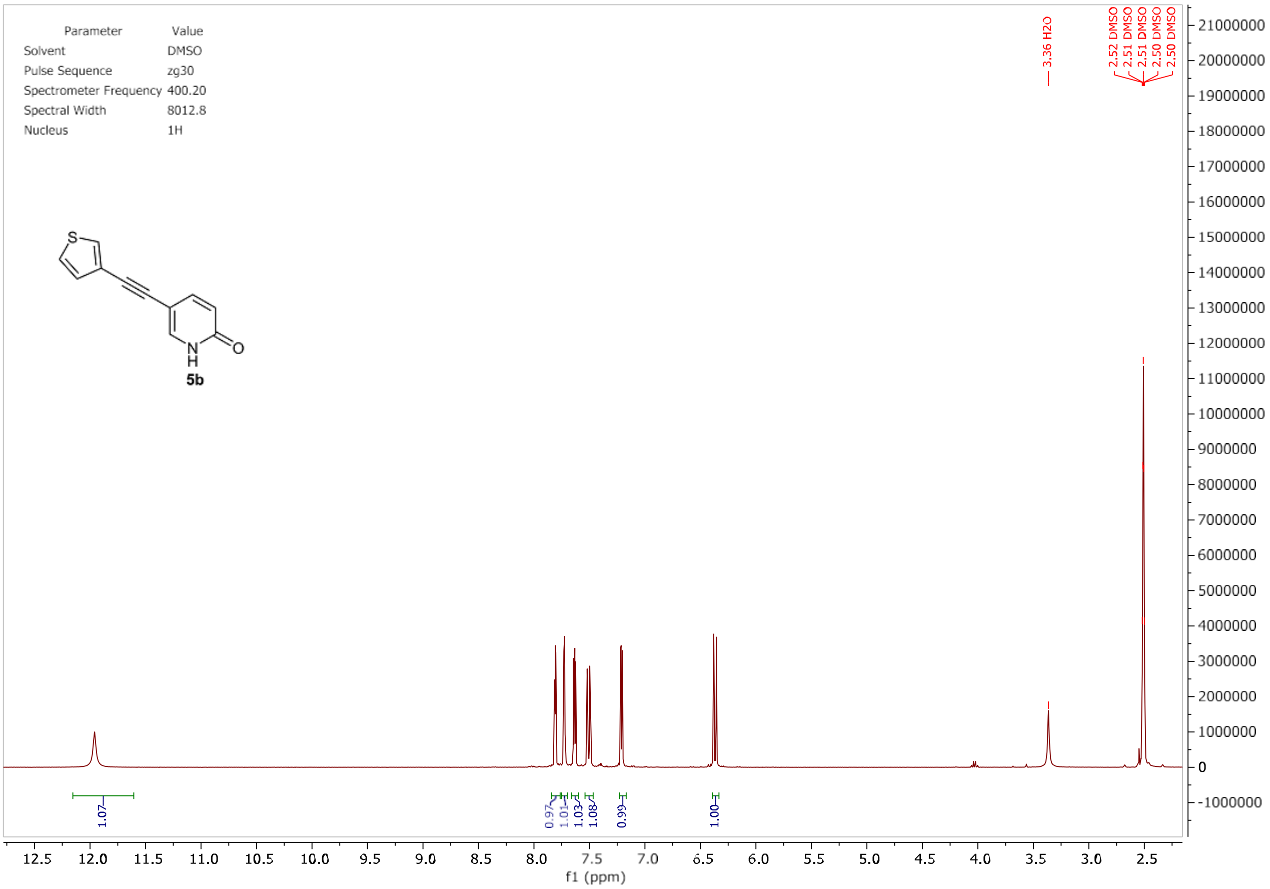

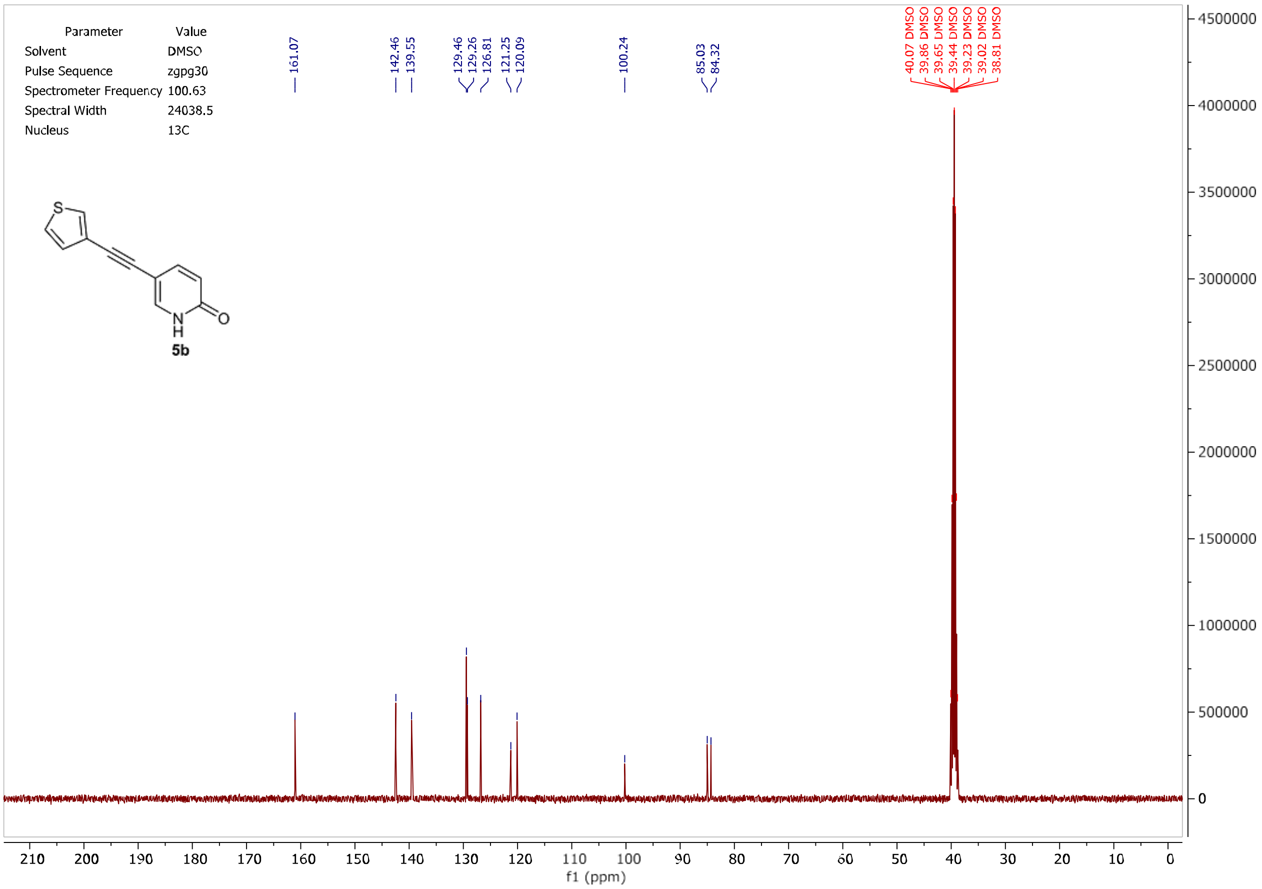

Figure S2.12. ^{1}H NMR (top) and ^{13}C NMR (bottom) spectra of compound **5b**

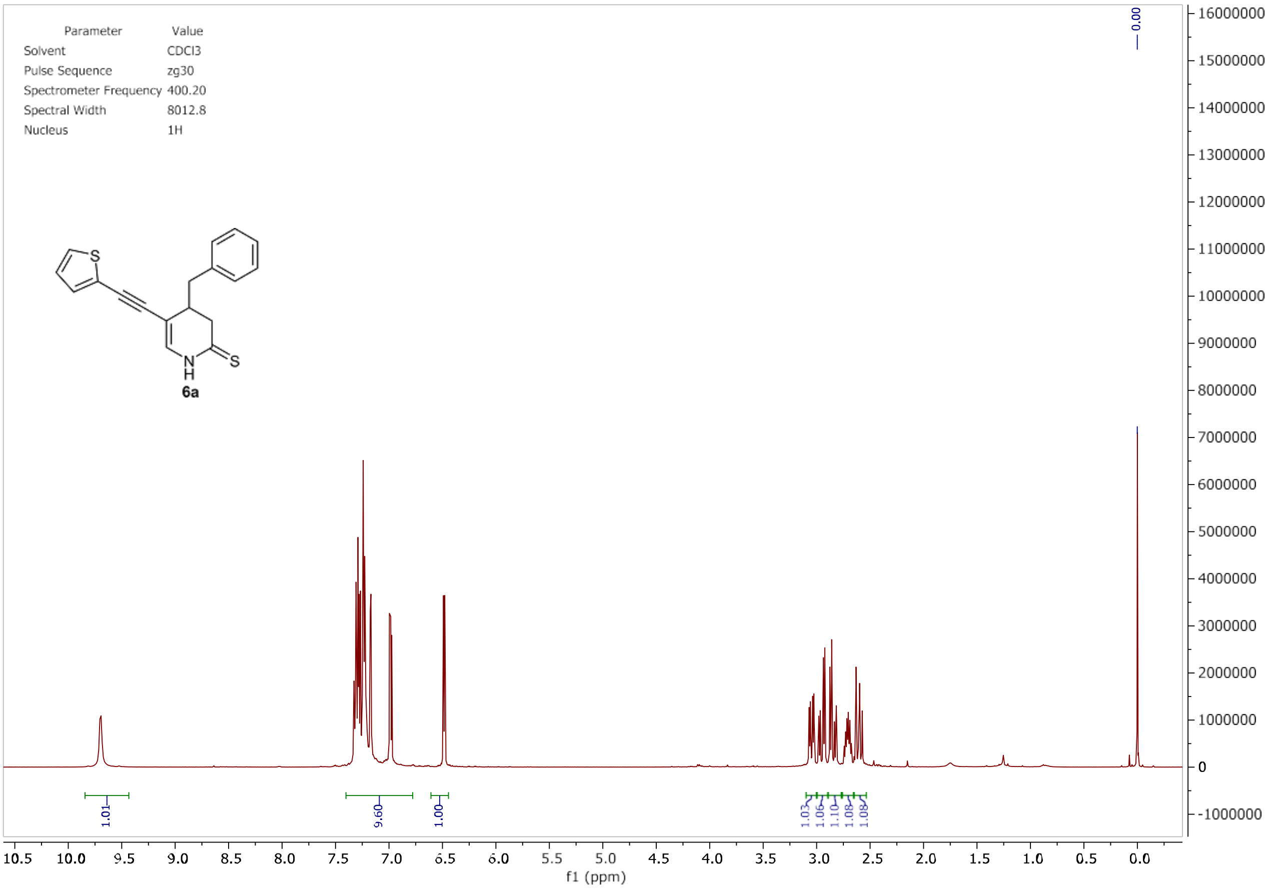

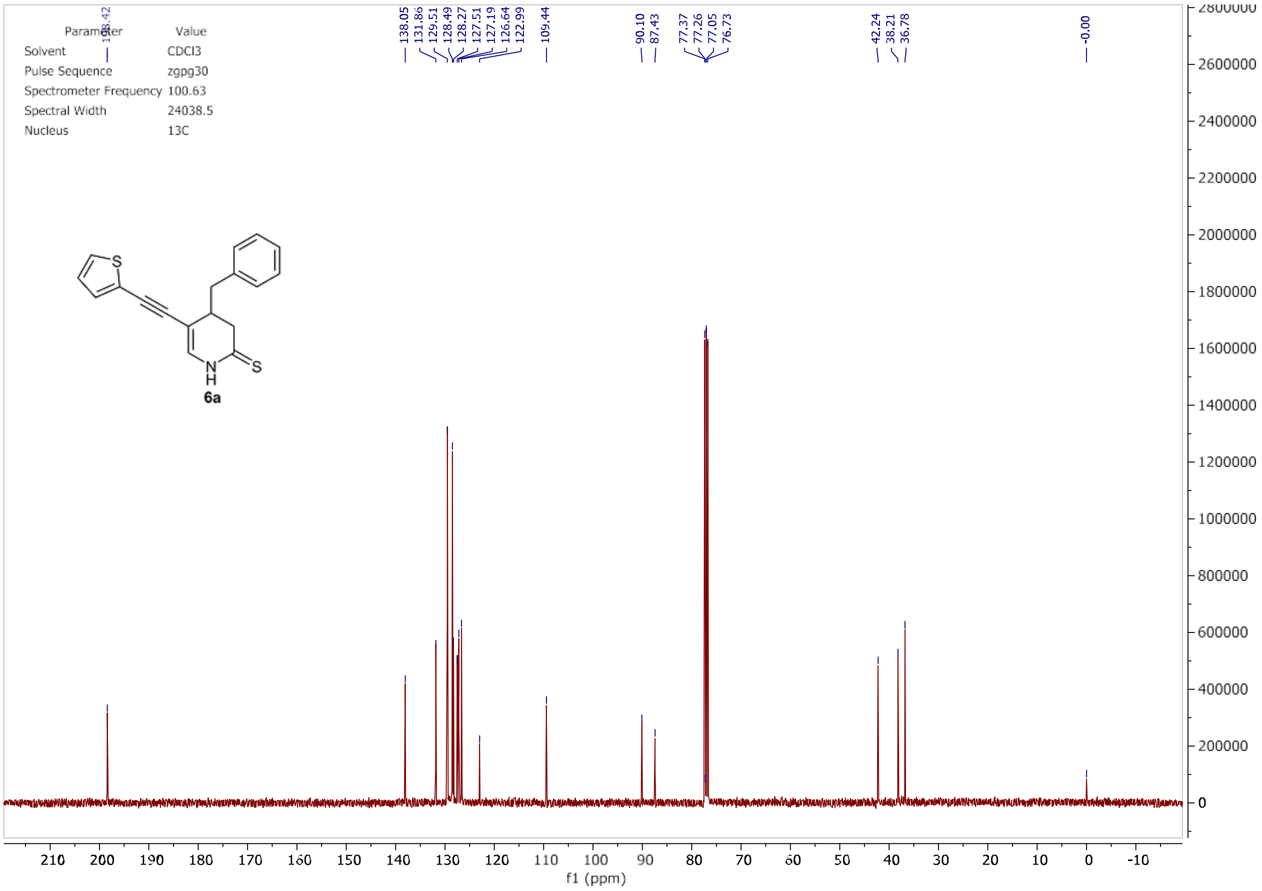

Figure S2.13. ^{1}H NMR (top) and ^{13}C NMR (bottom) spectra of compound **6a**

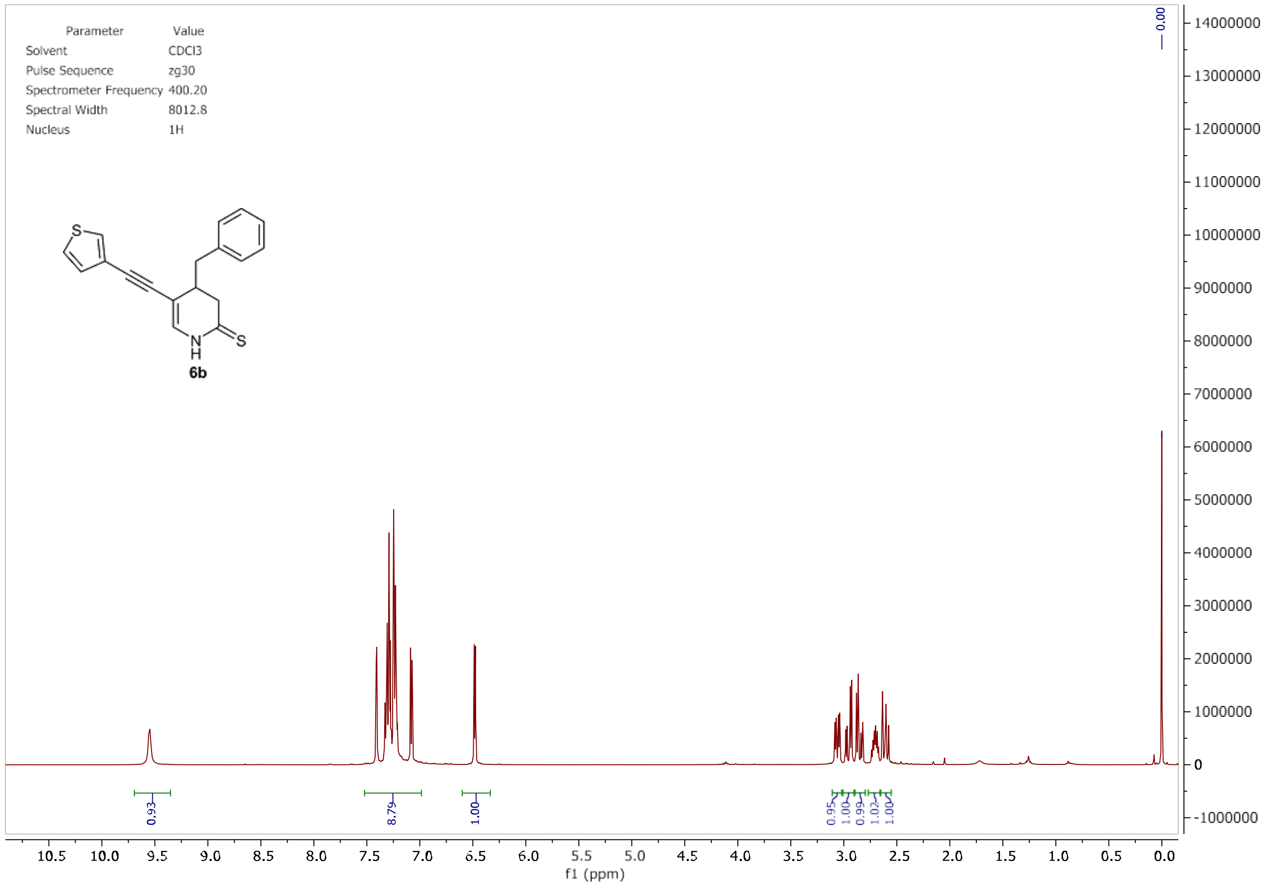

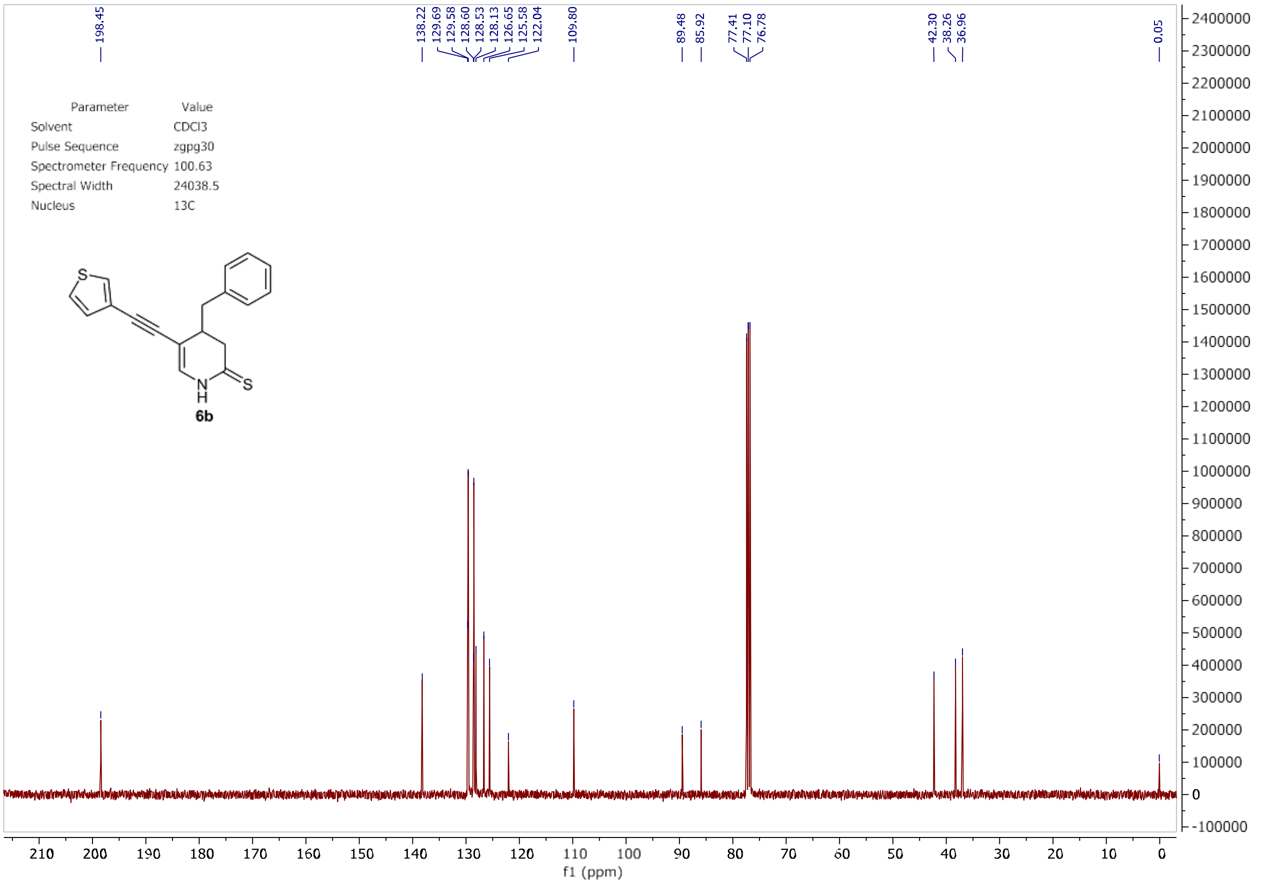

Figure S2.14. ^{1}H NMR (top) and ^{13}C NMR (bottom) spectra of compound **6b**

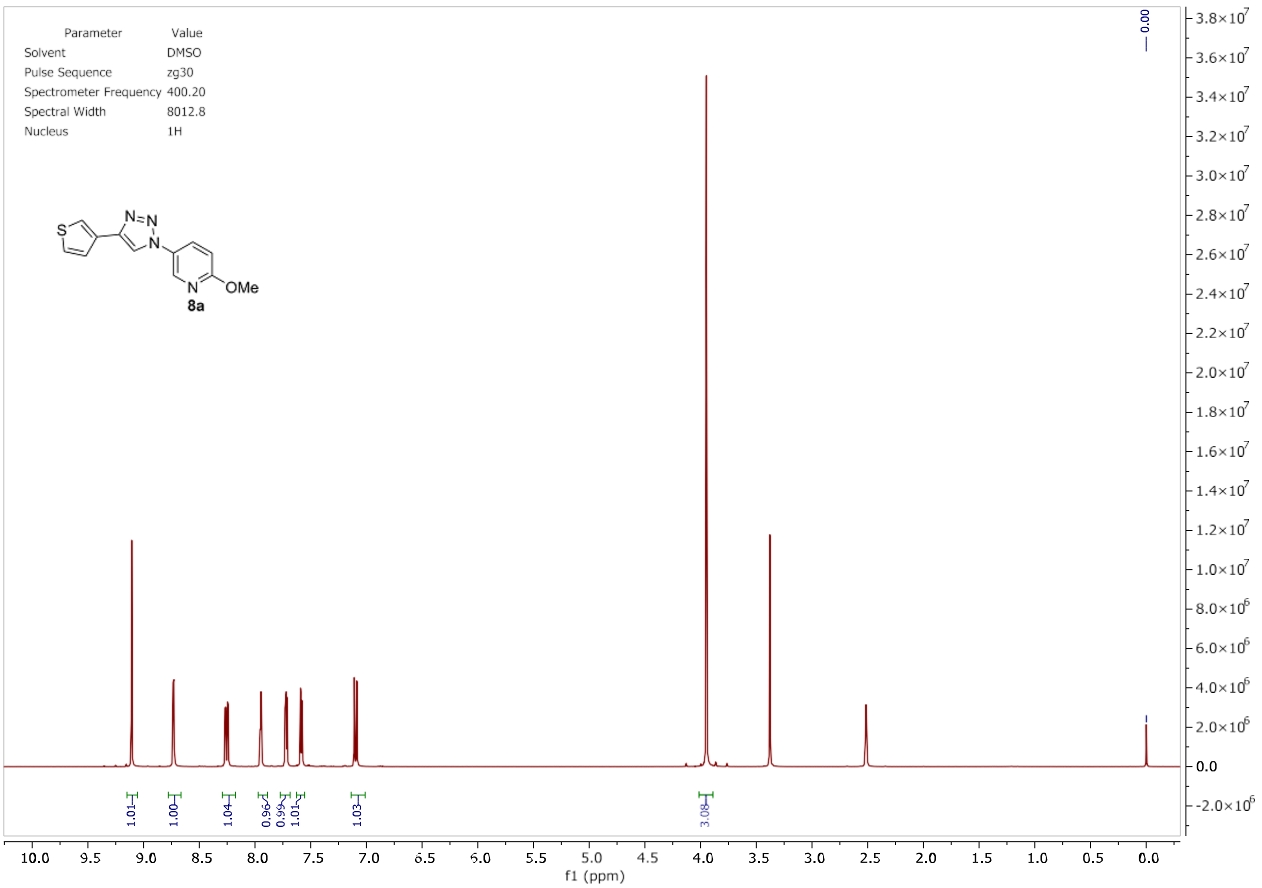

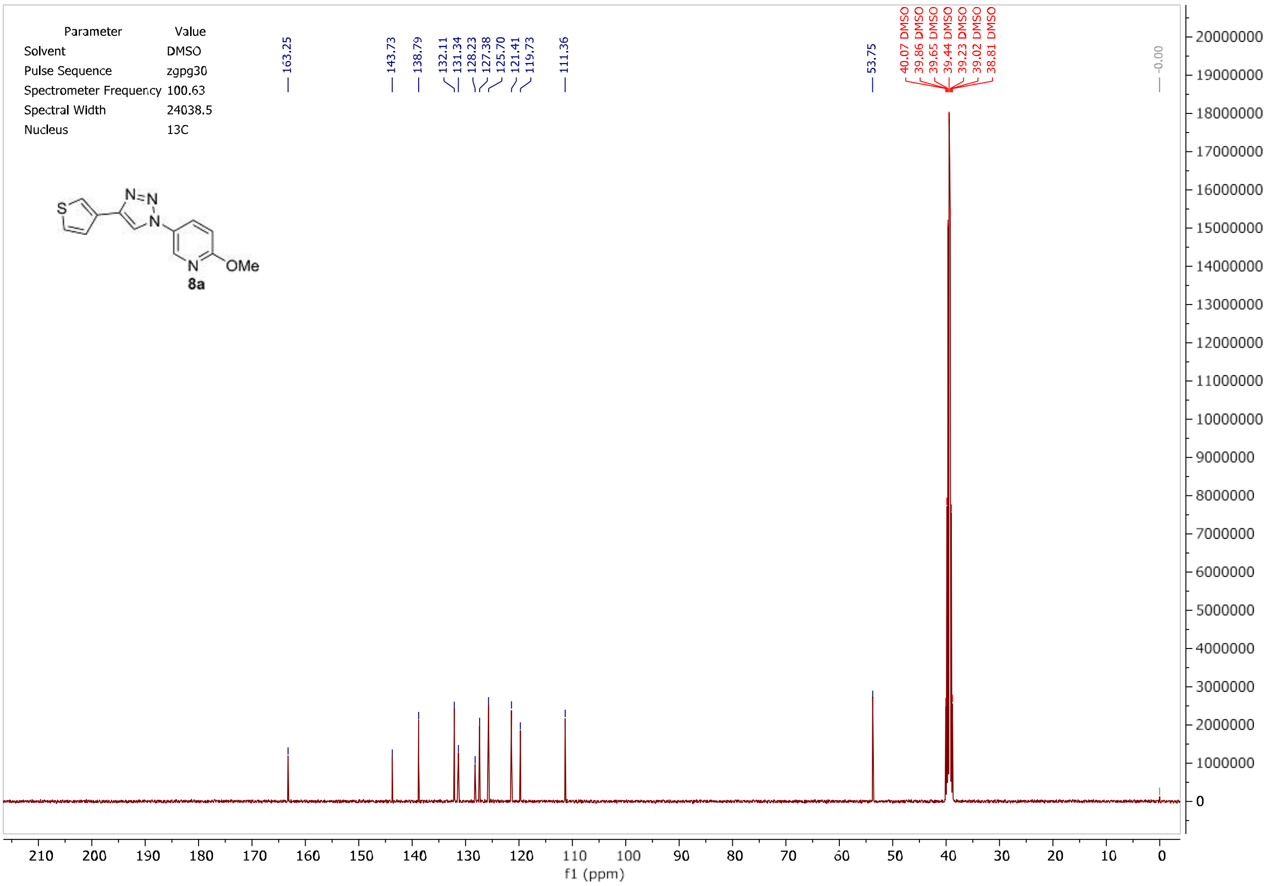

Figure S2.15. ^{1}H NMR (top) and ^{13}C NMR (bottom) spectra of compound **8a**

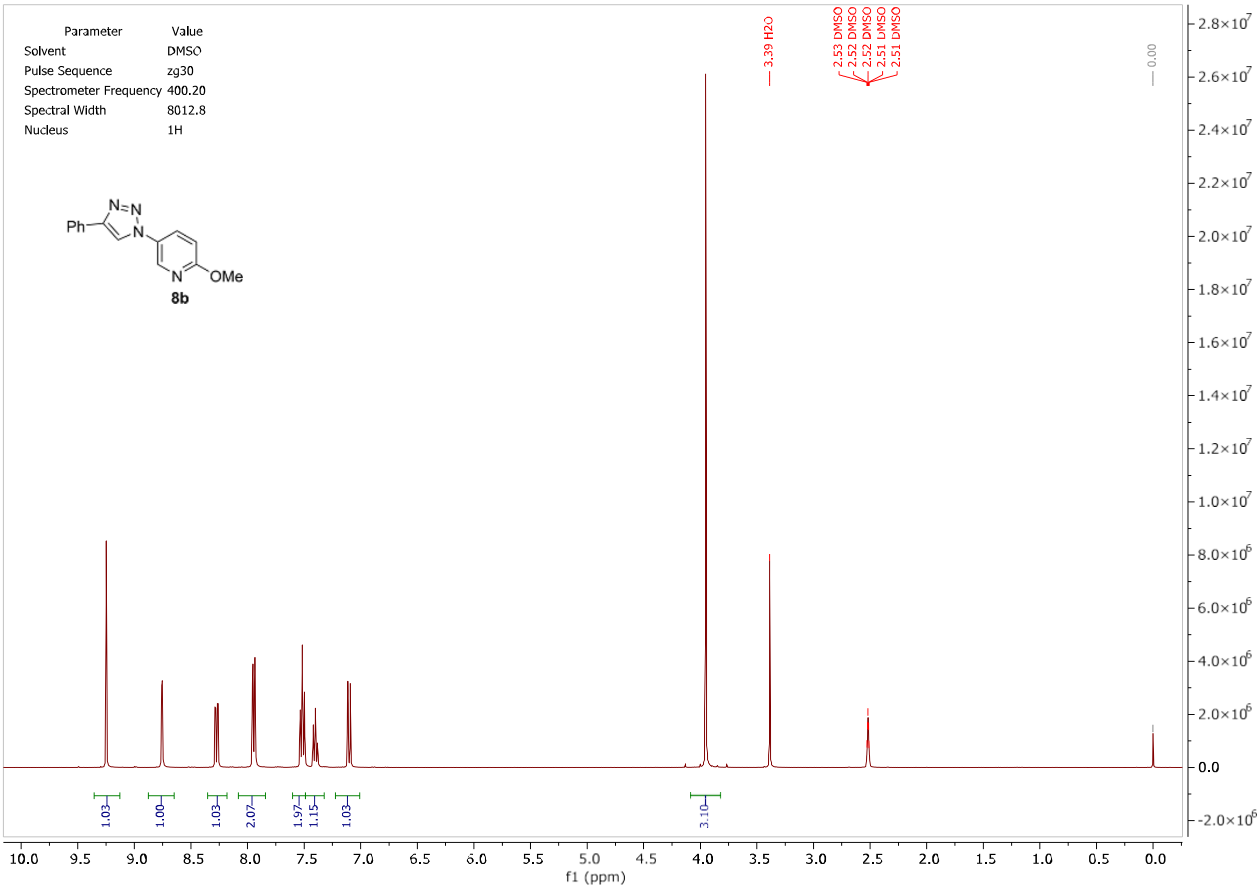

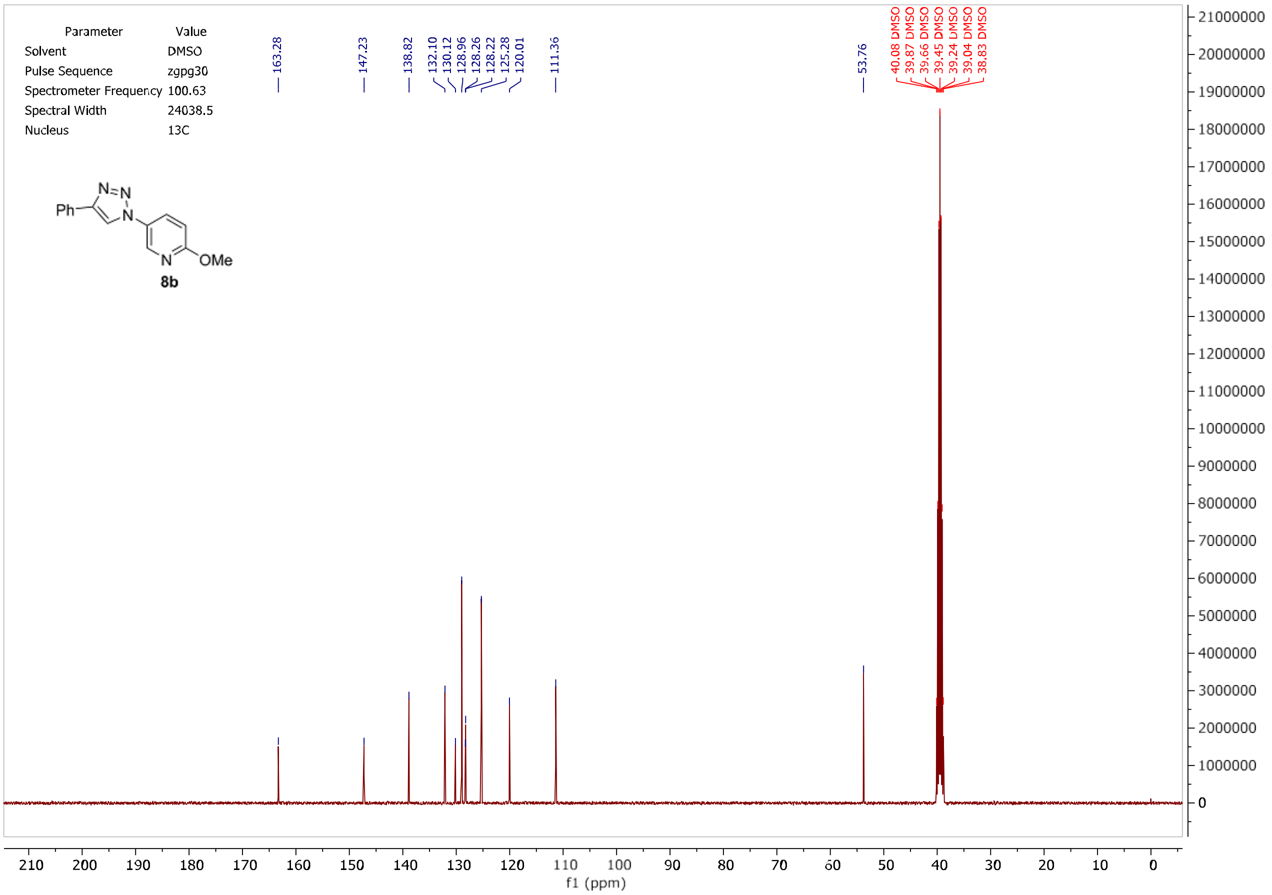

Figure S2.16. ^{1}H NMR (top) and ^{13}C NMR (bottom) spectra of compound **8b**

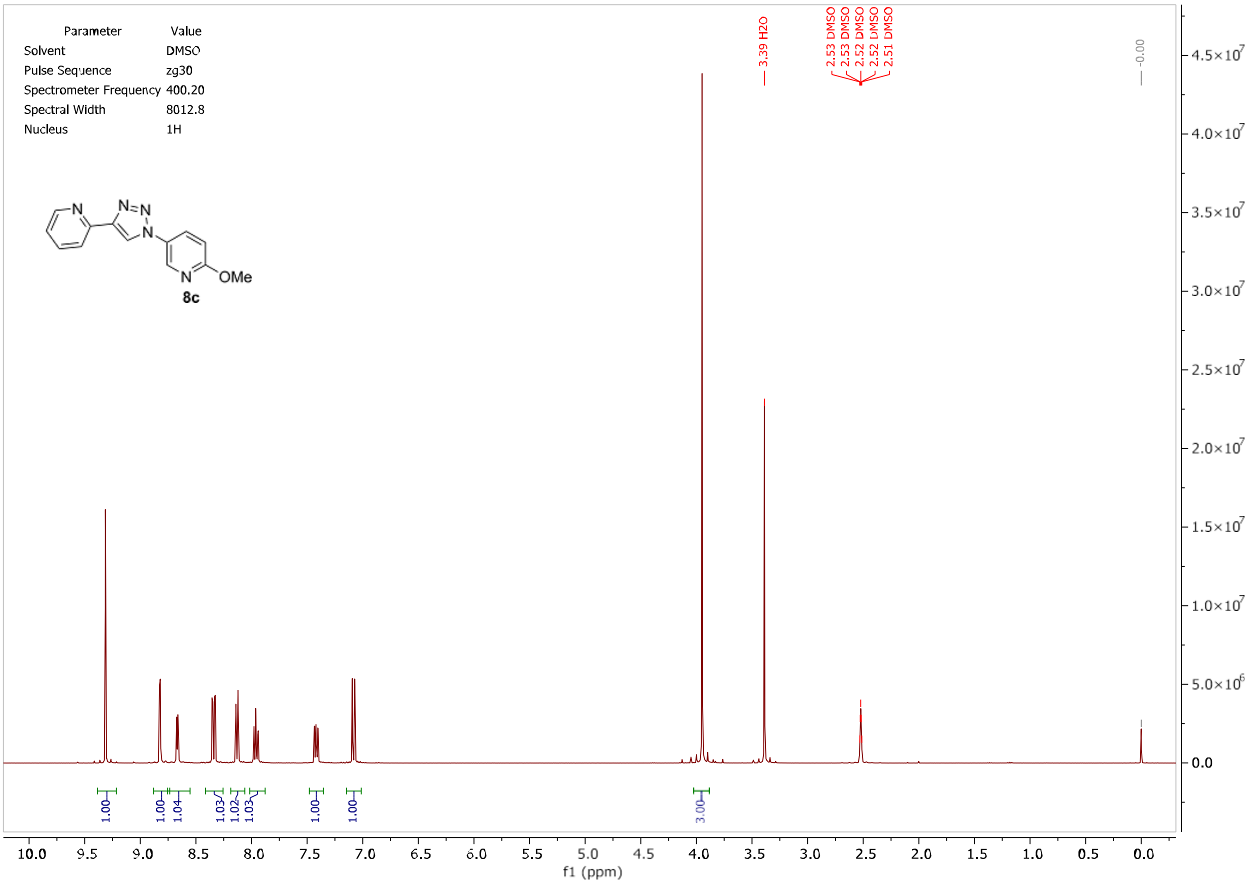

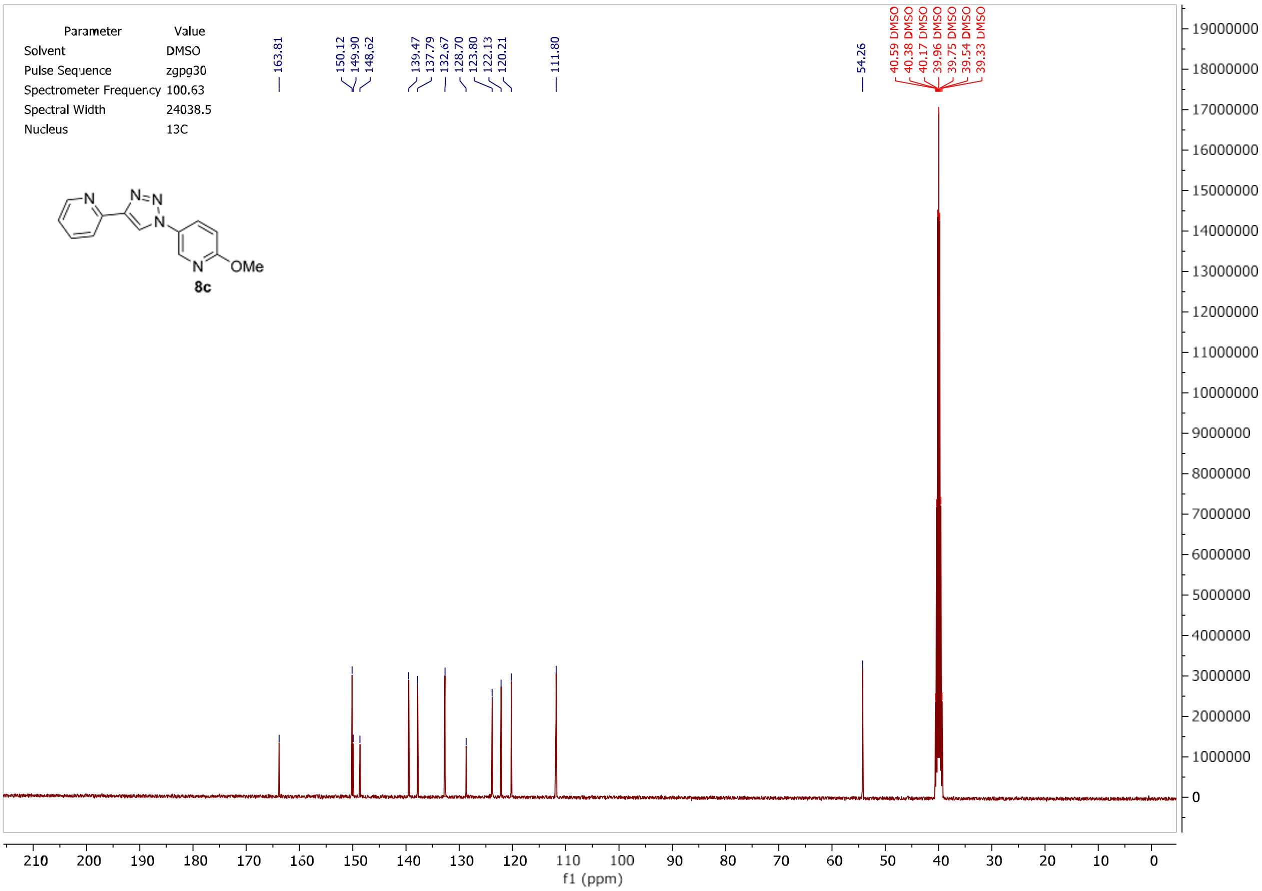

Figure S2.17. ^{1}H NMR (top) and ^{13}C NMR (bottom) spectra of compound **8c**

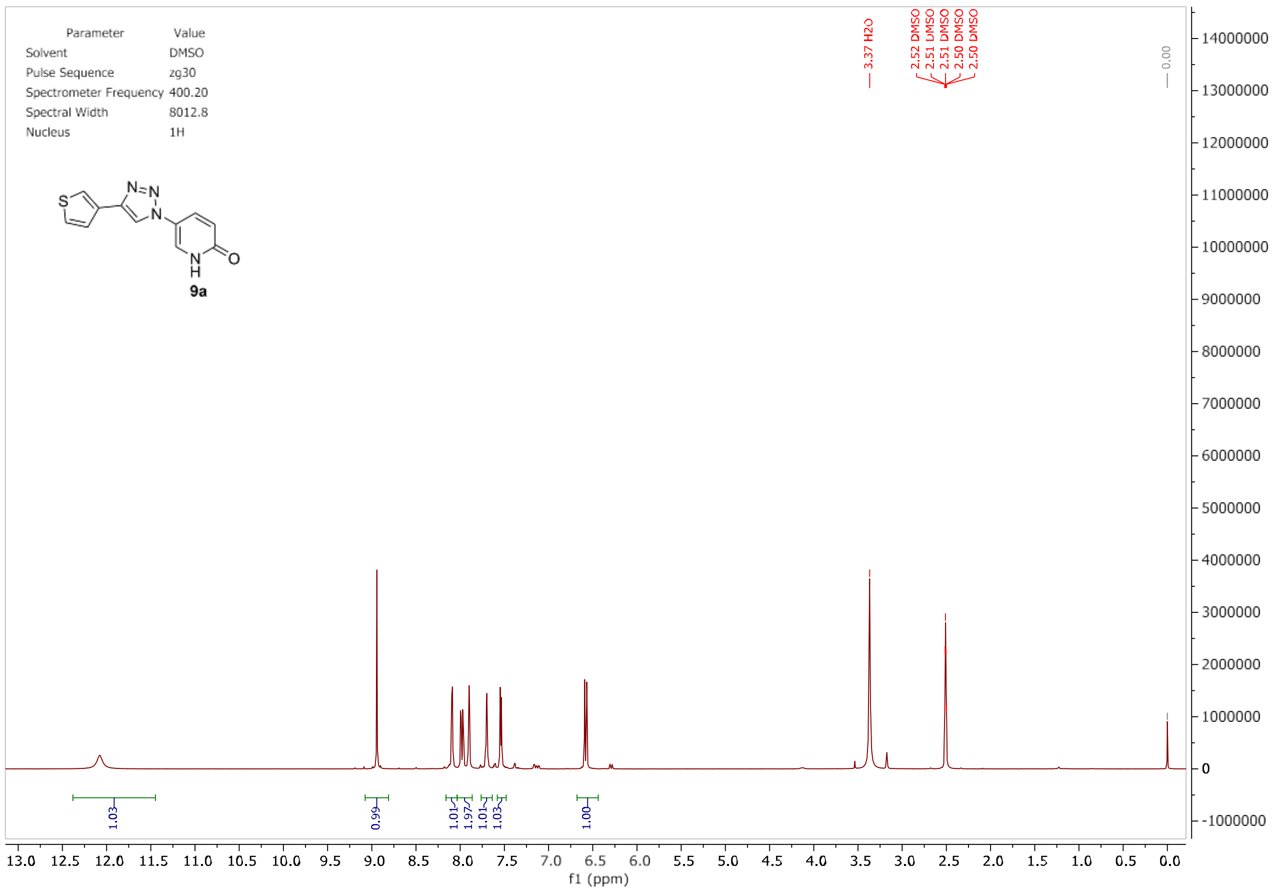

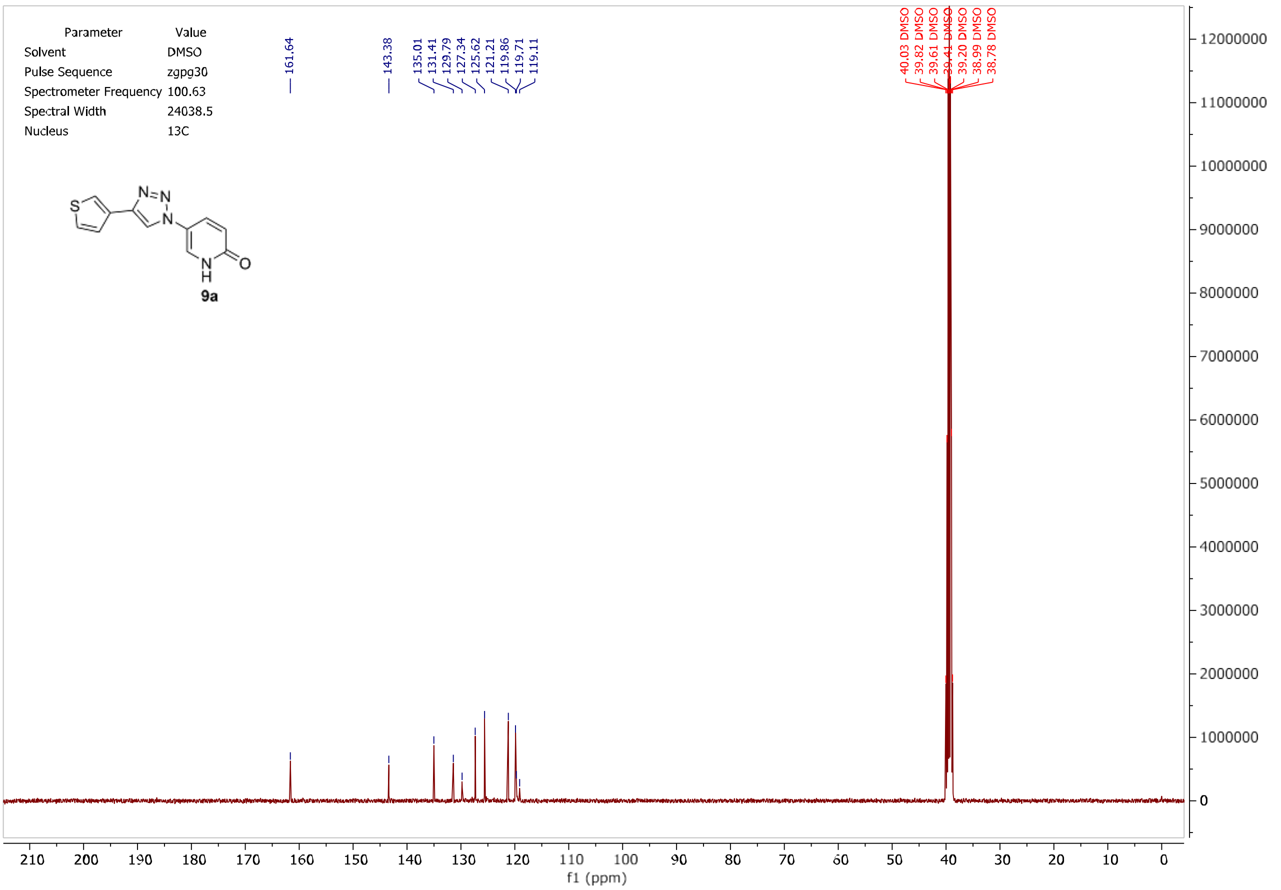

Figure S2.18. ^{1}H NMR (top) and ^{13}C NMR (bottom) spectra of compound **9a**

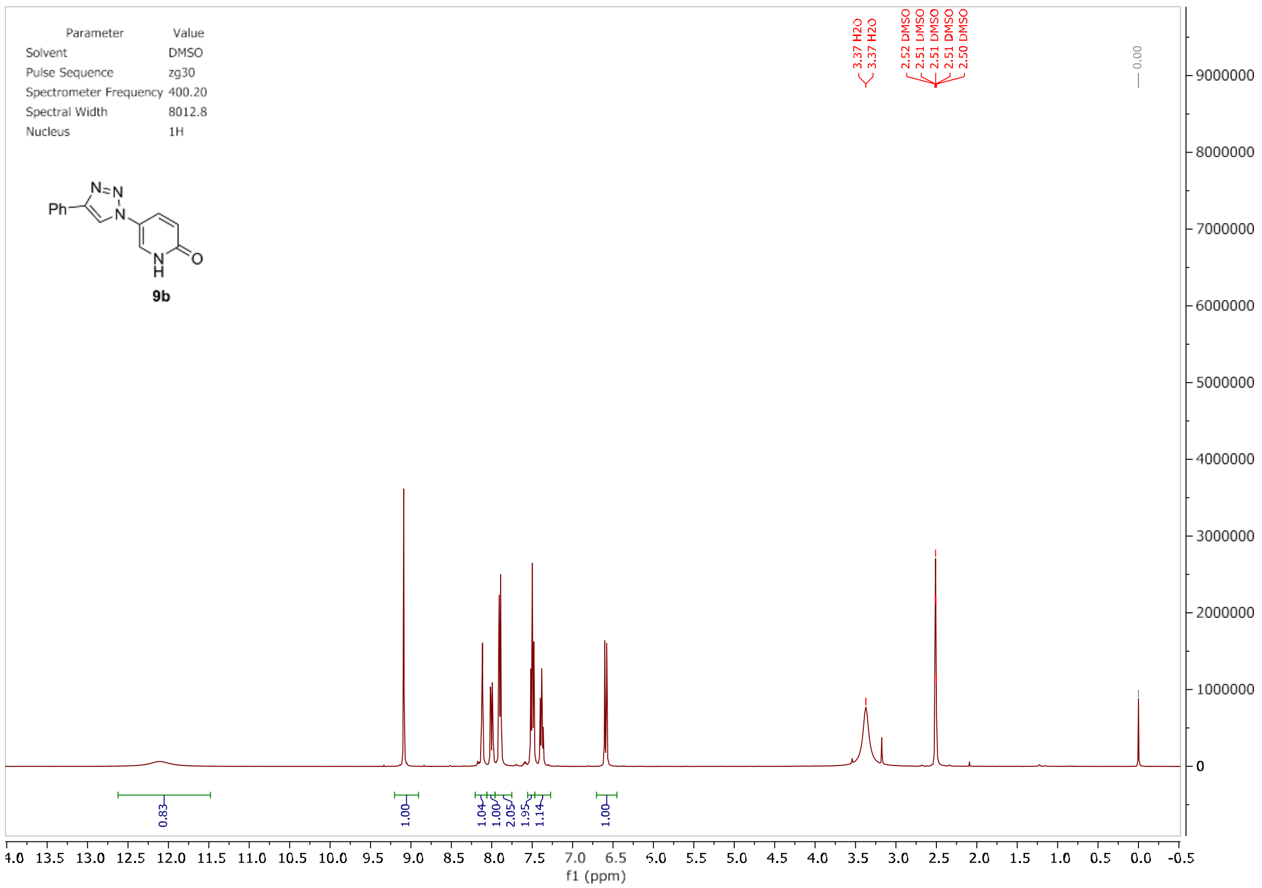

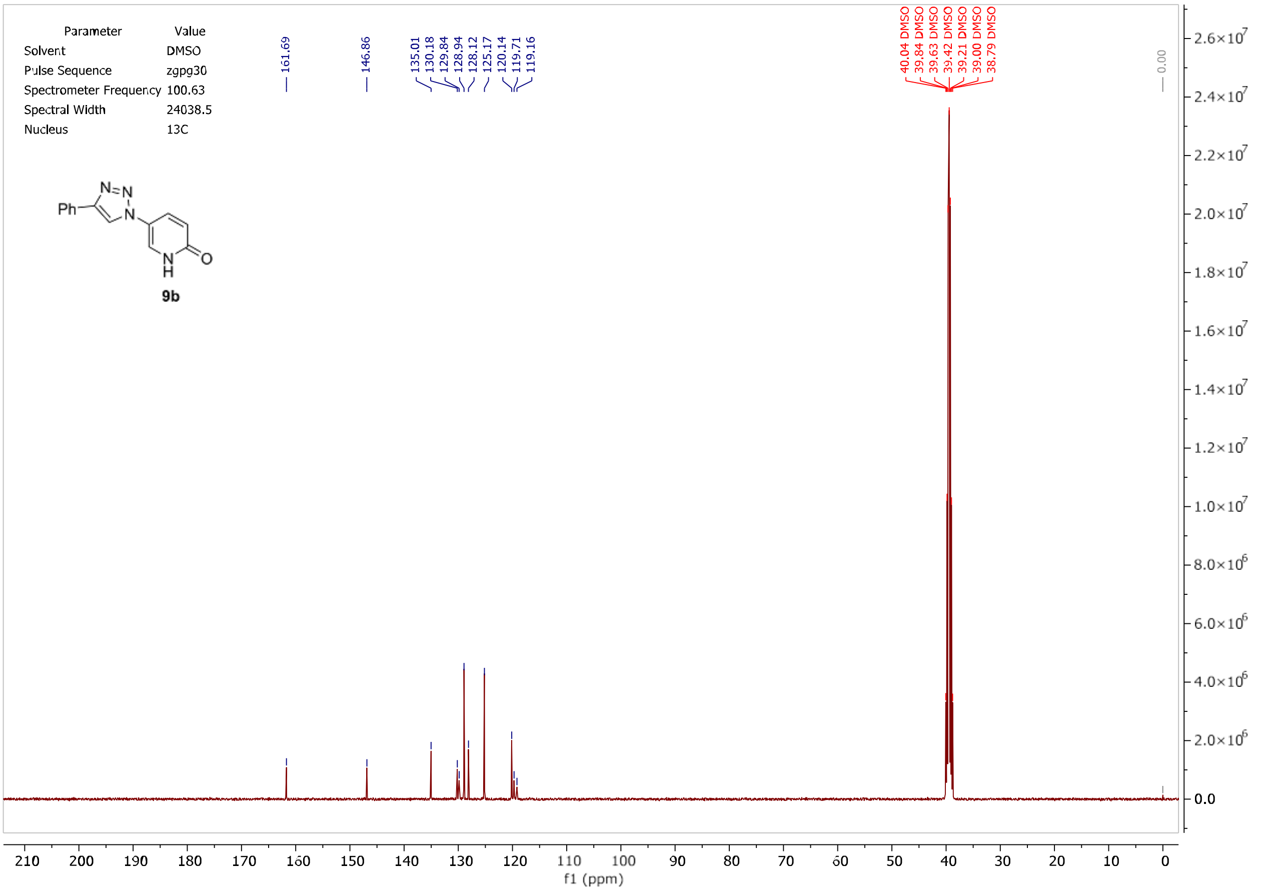

Figure S2.19. ^{1}H NMR (top) and ^{13}C NMR (bottom) spectra of compound **9b**

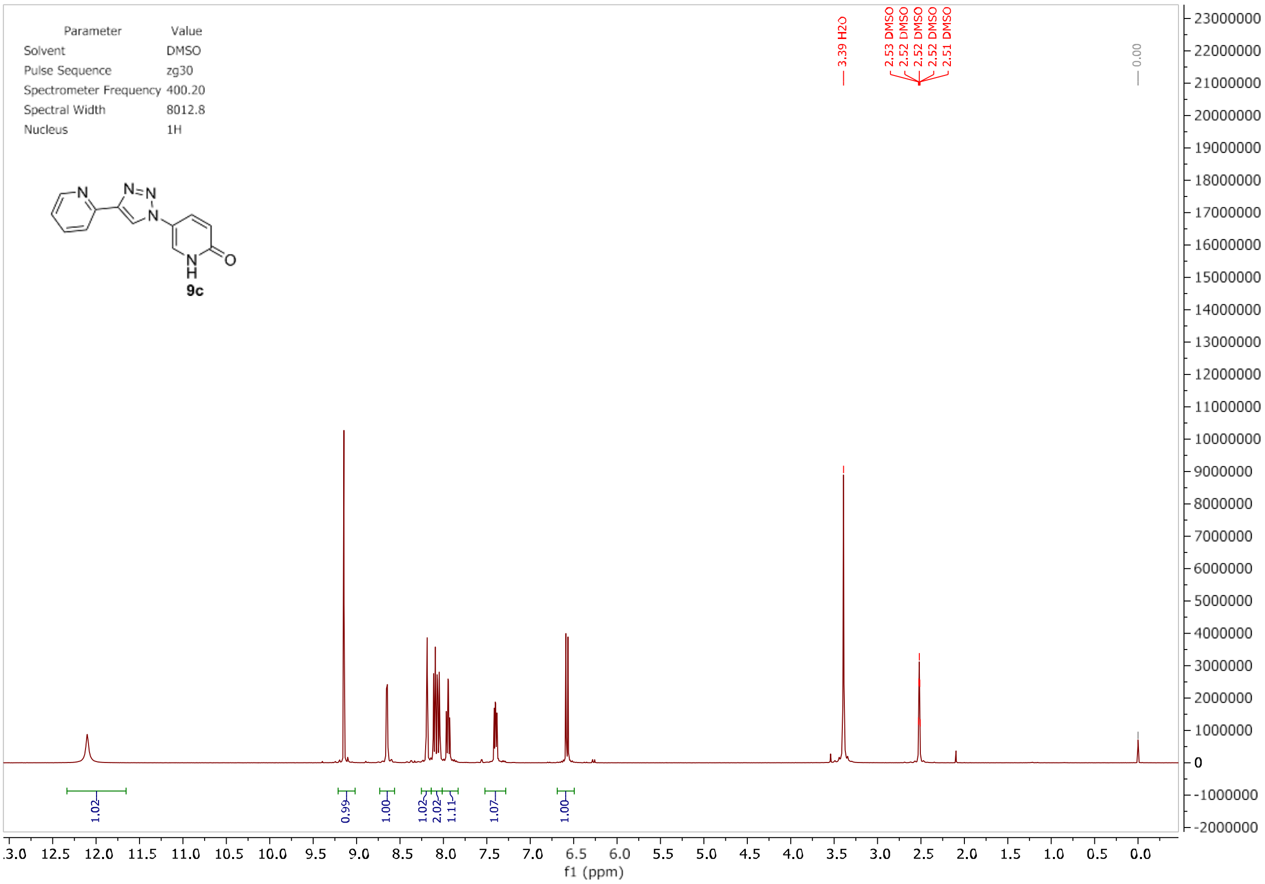

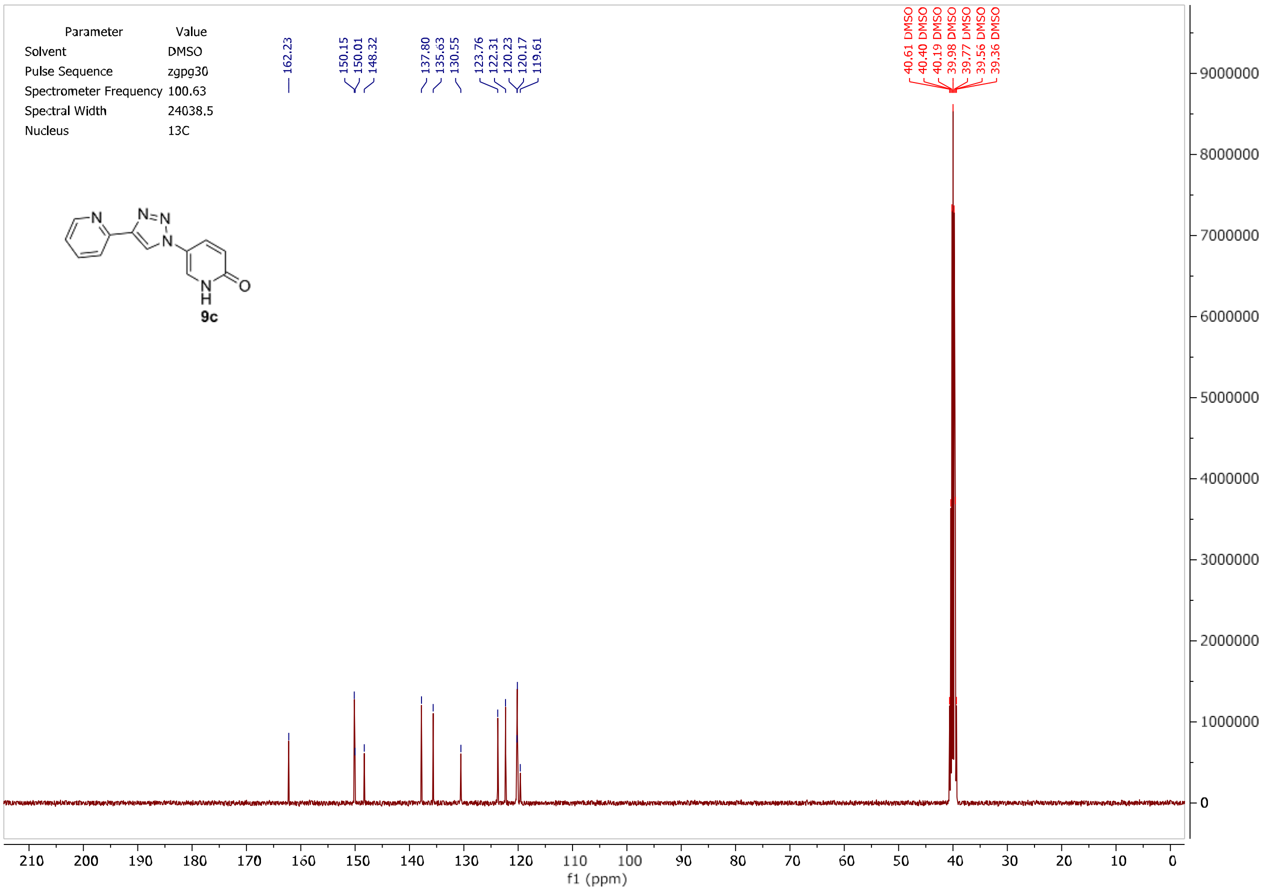

Figure S2.20. ^{1}H NMR (top) and ^{13}C NMR (bottom) spectra of compound **9c**

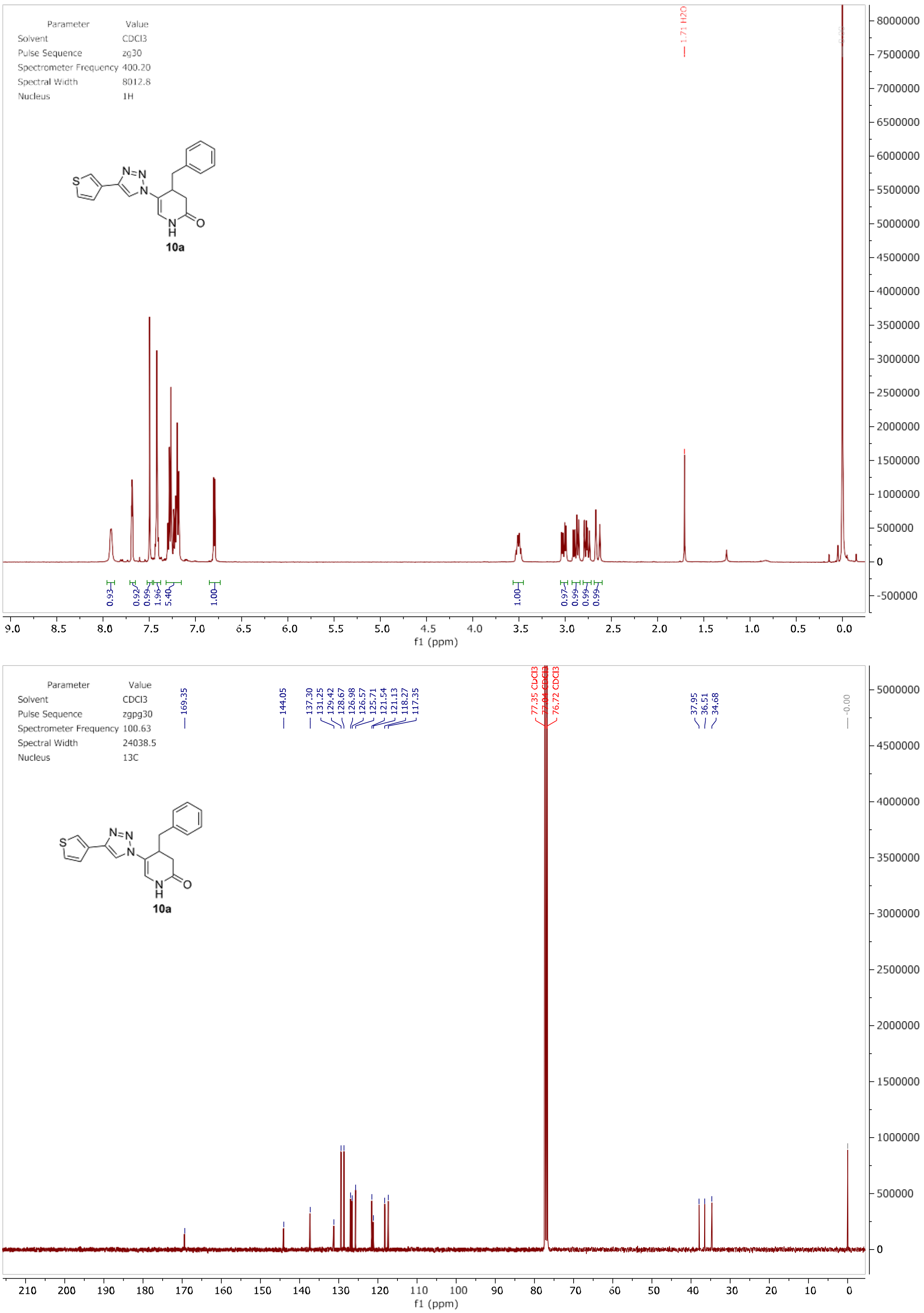

Figure S2.21. ^{1}H NMR (top) and ^{13}C NMR (bottom) spectra of compound **10a**

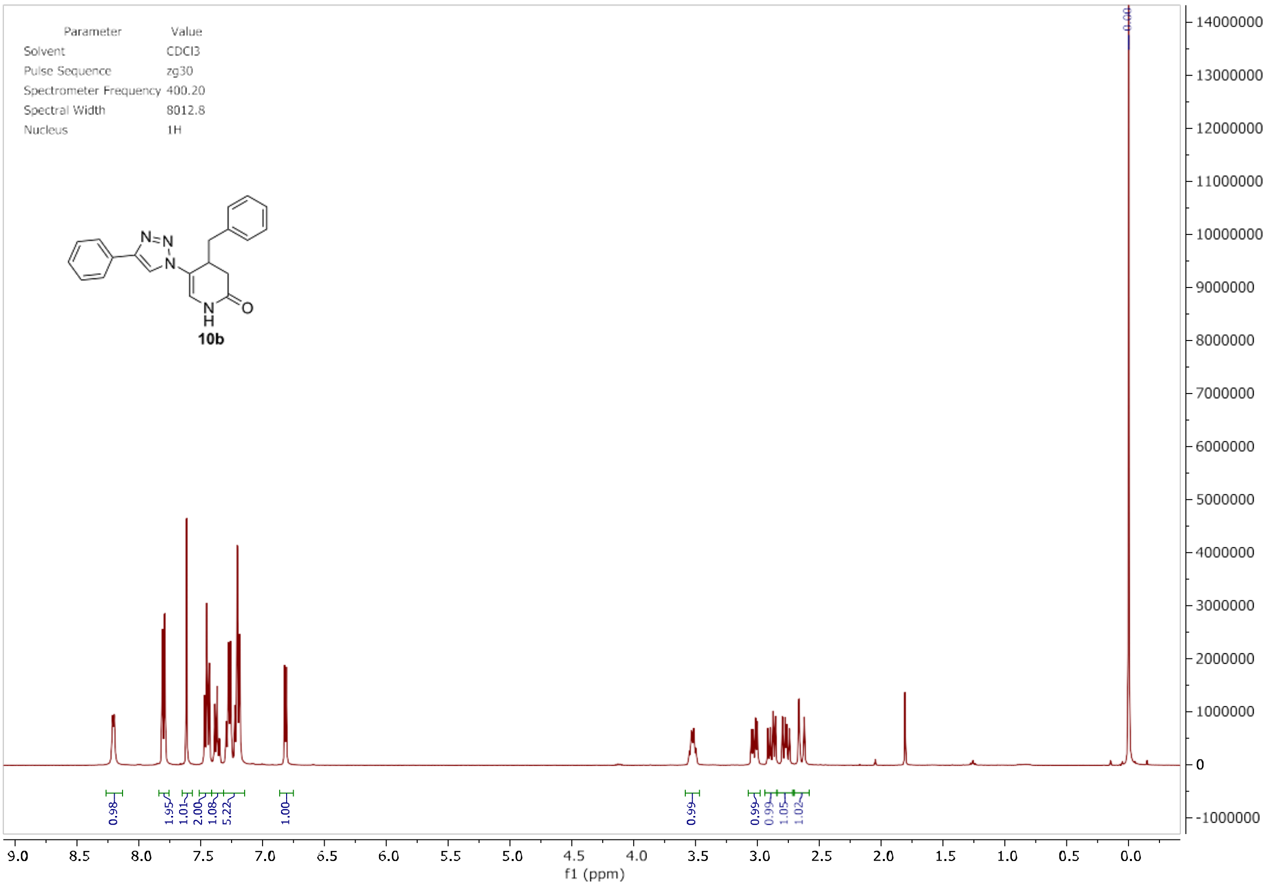

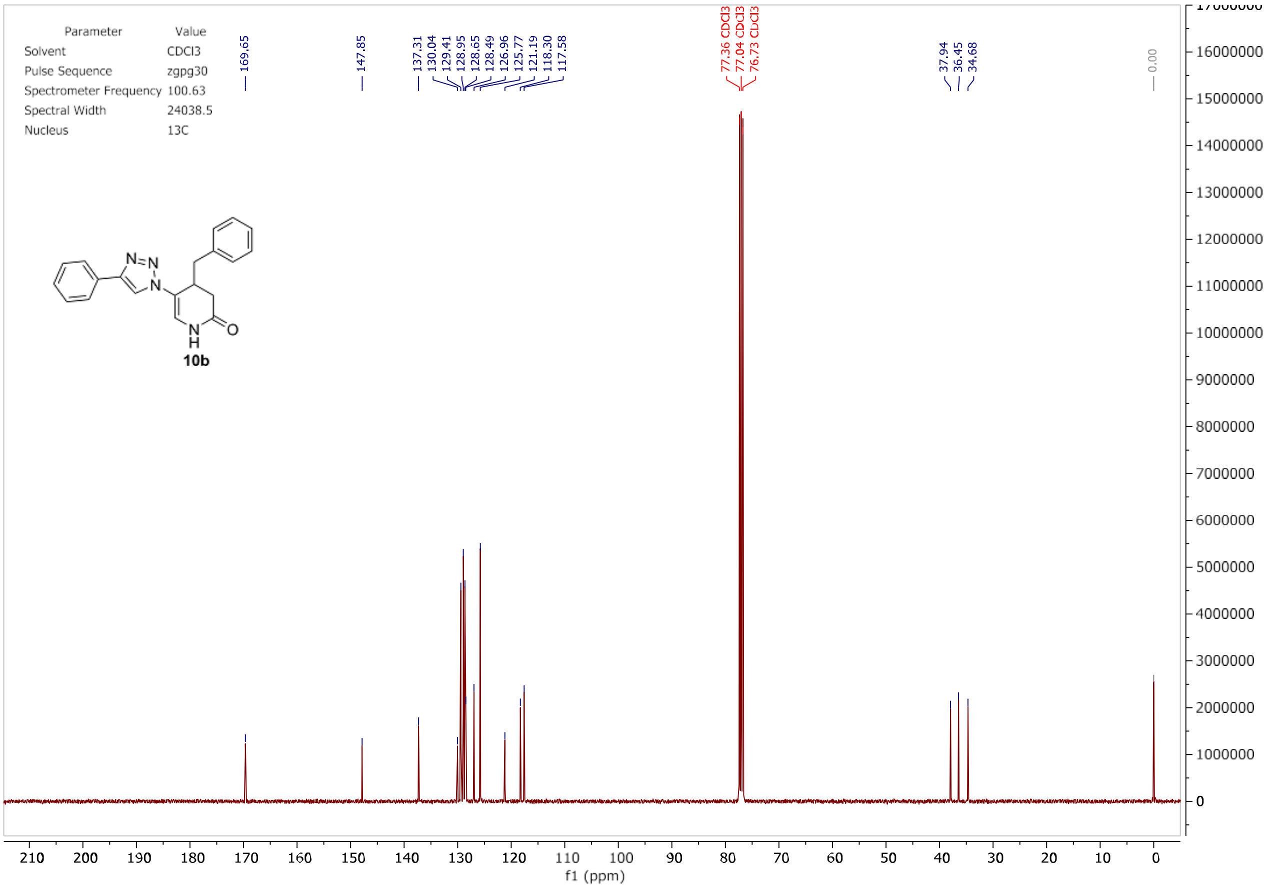

Figure S2.22. ^{1}H NMR (top) and ^{13}C NMR (bottom) spectra of compound **10b**

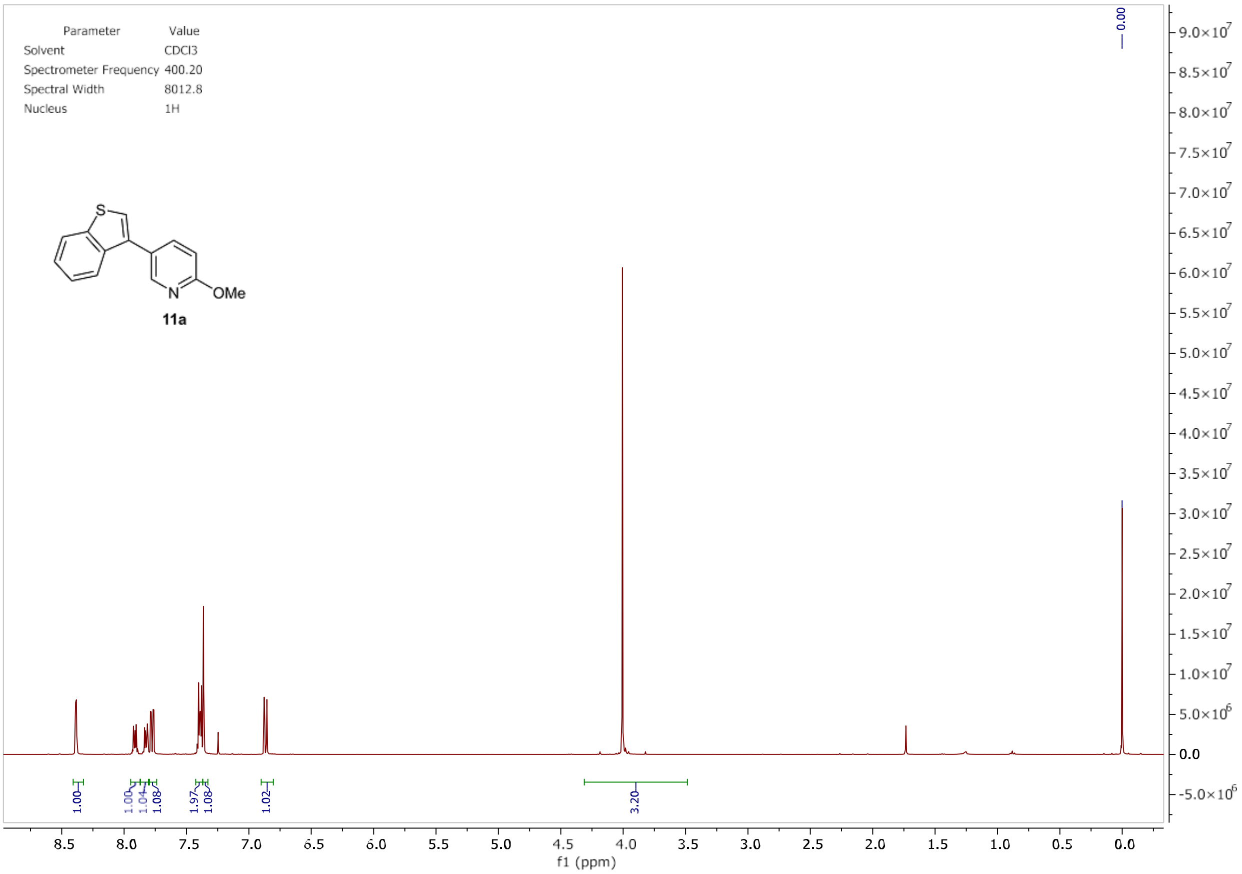

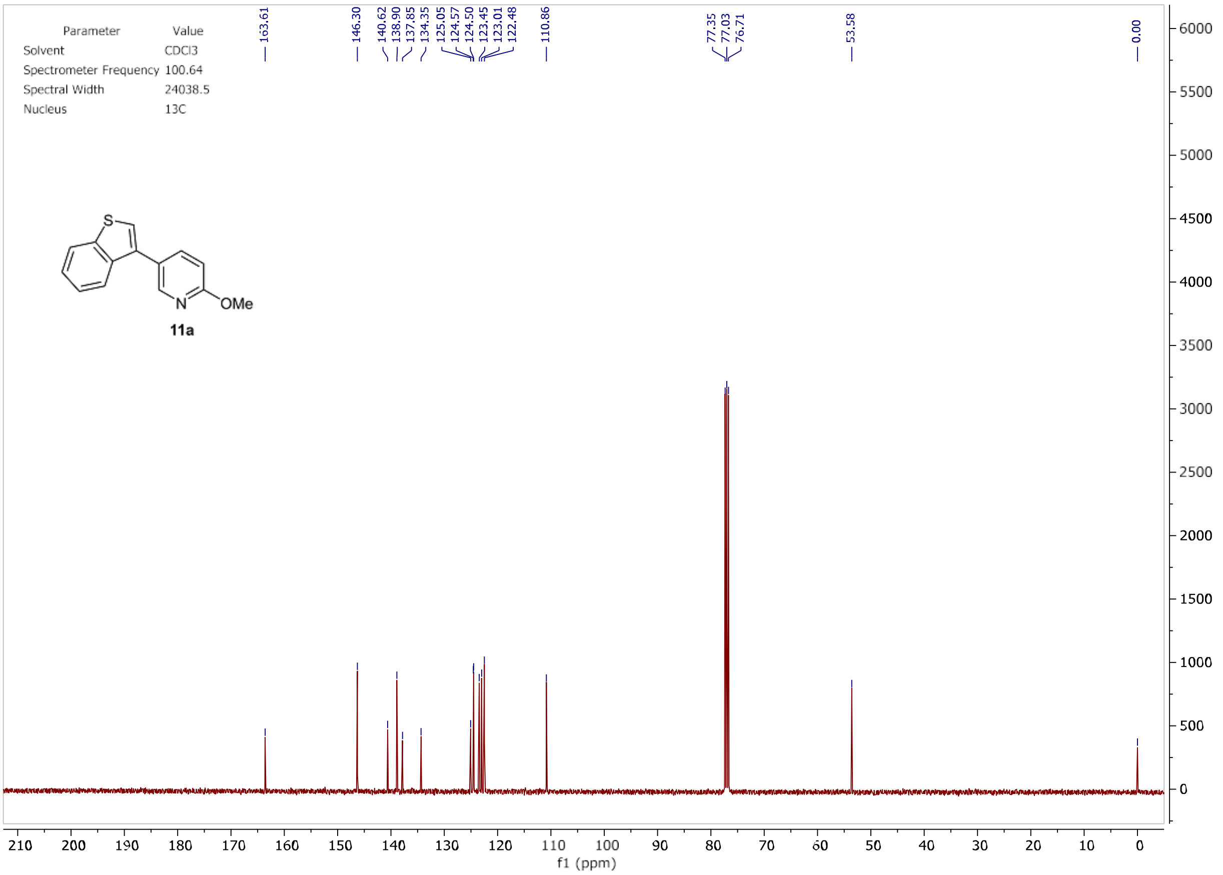

Figure S2.23. ^{1}H NMR (top) and ^{13}C NMR (bottom) spectra of compound **11a**

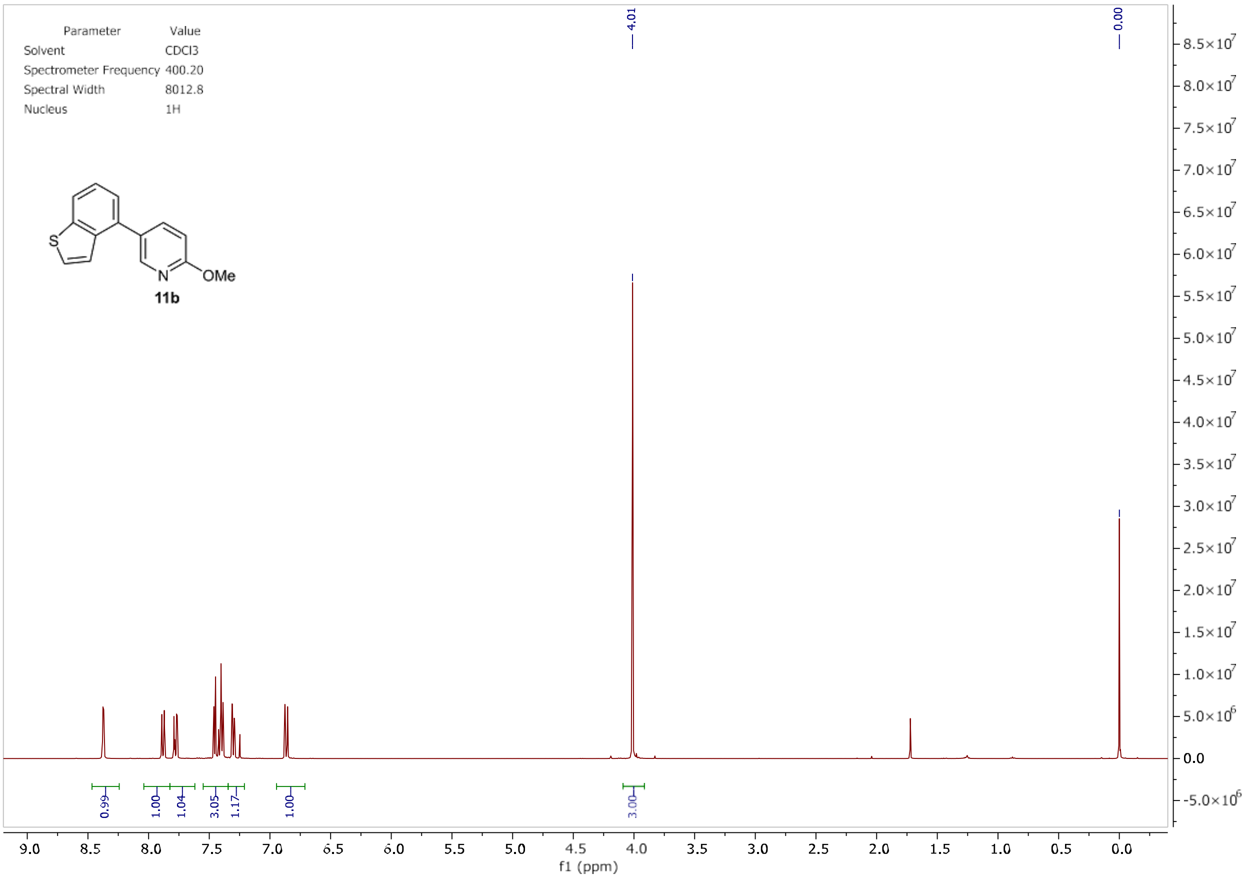

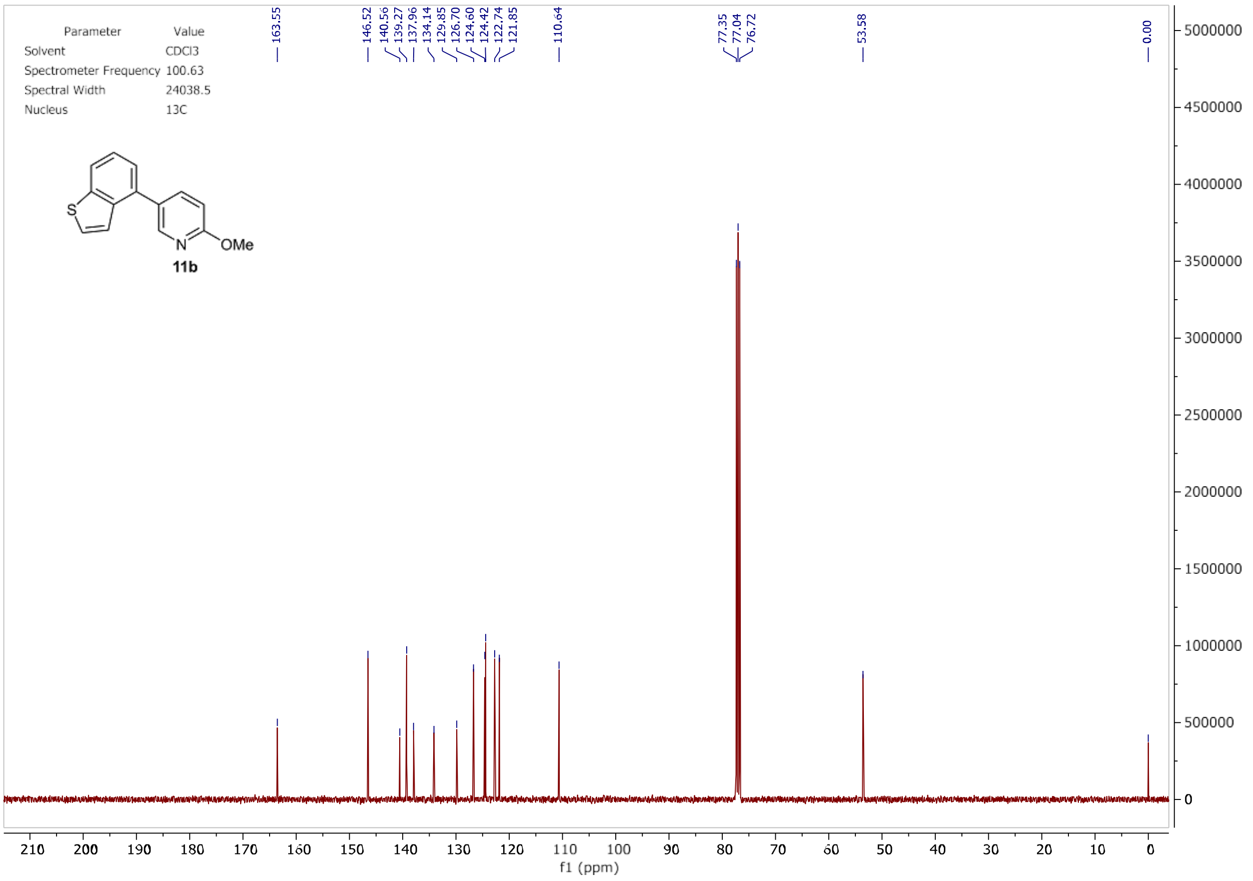

Figure S2.24. ^{1}H NMR (top) and ^{13}C NMR (bottom) spectra of compound **11b**

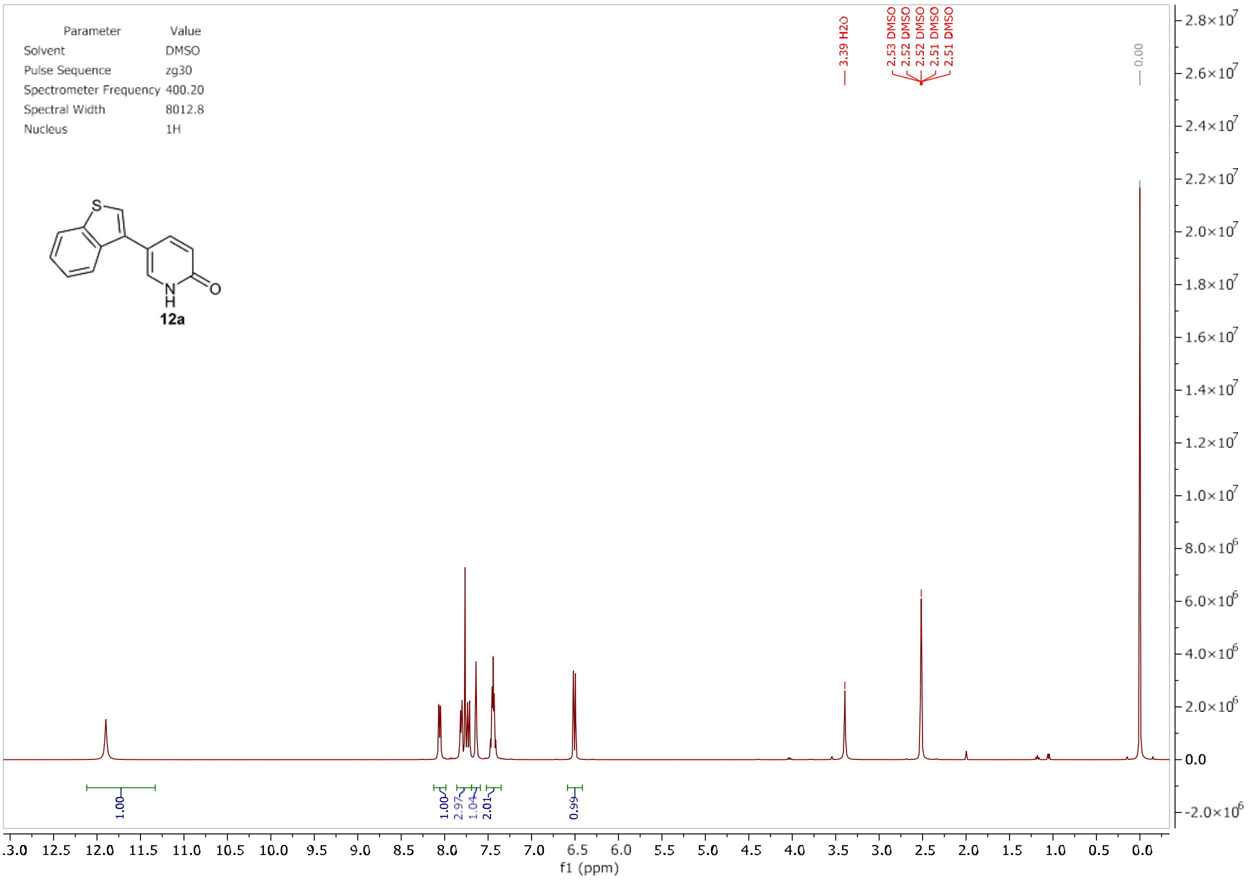

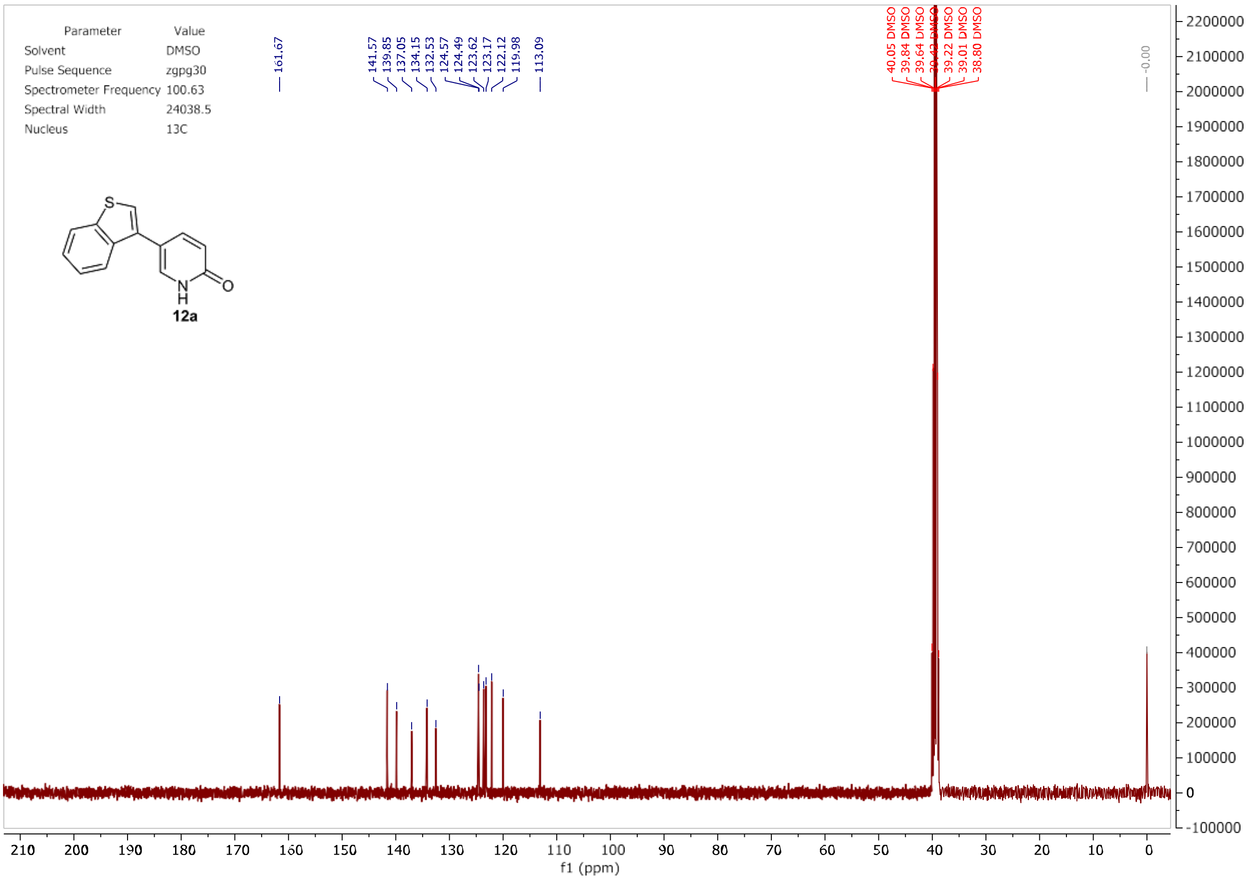

Figure S2.25. ^{1}H NMR (top) and ^{13}C NMR (bottom) spectra of compound **12a**

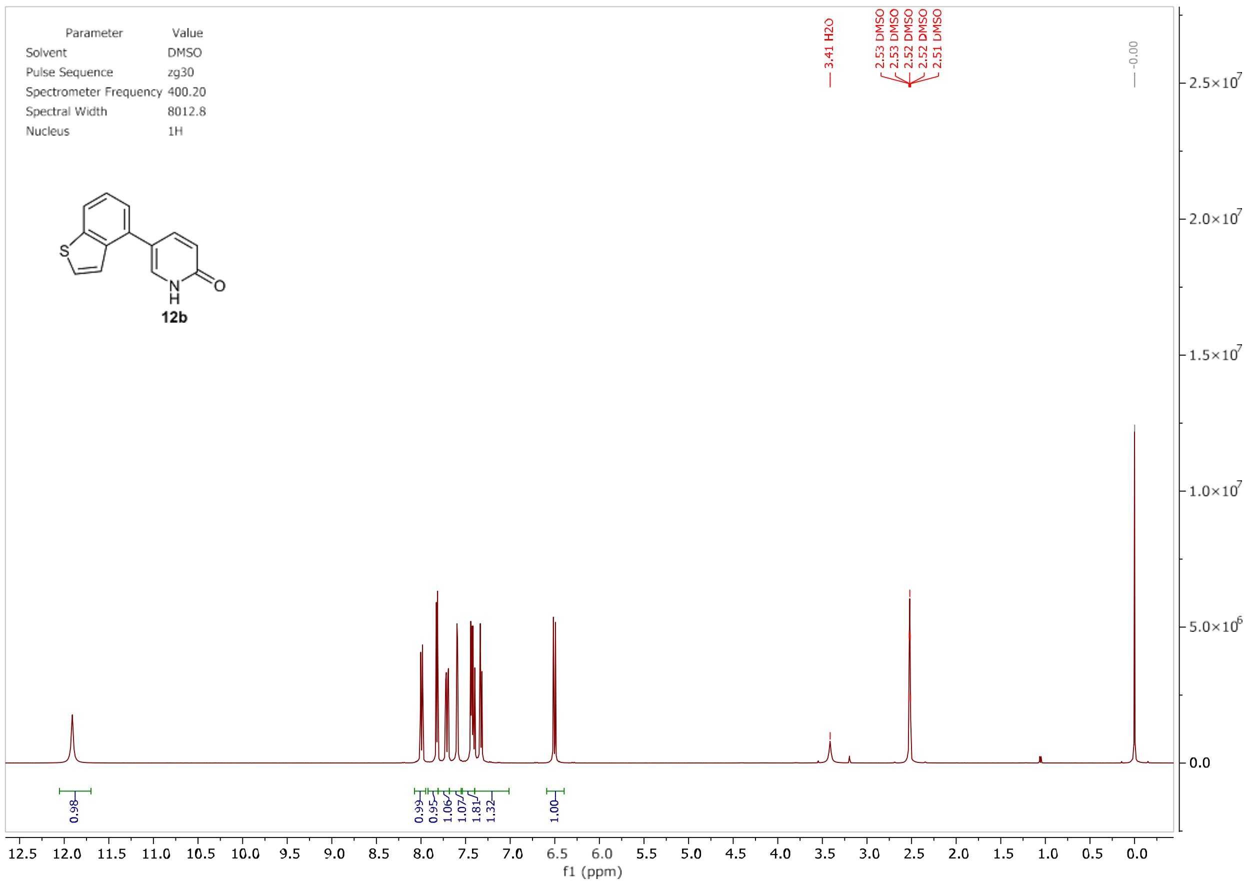

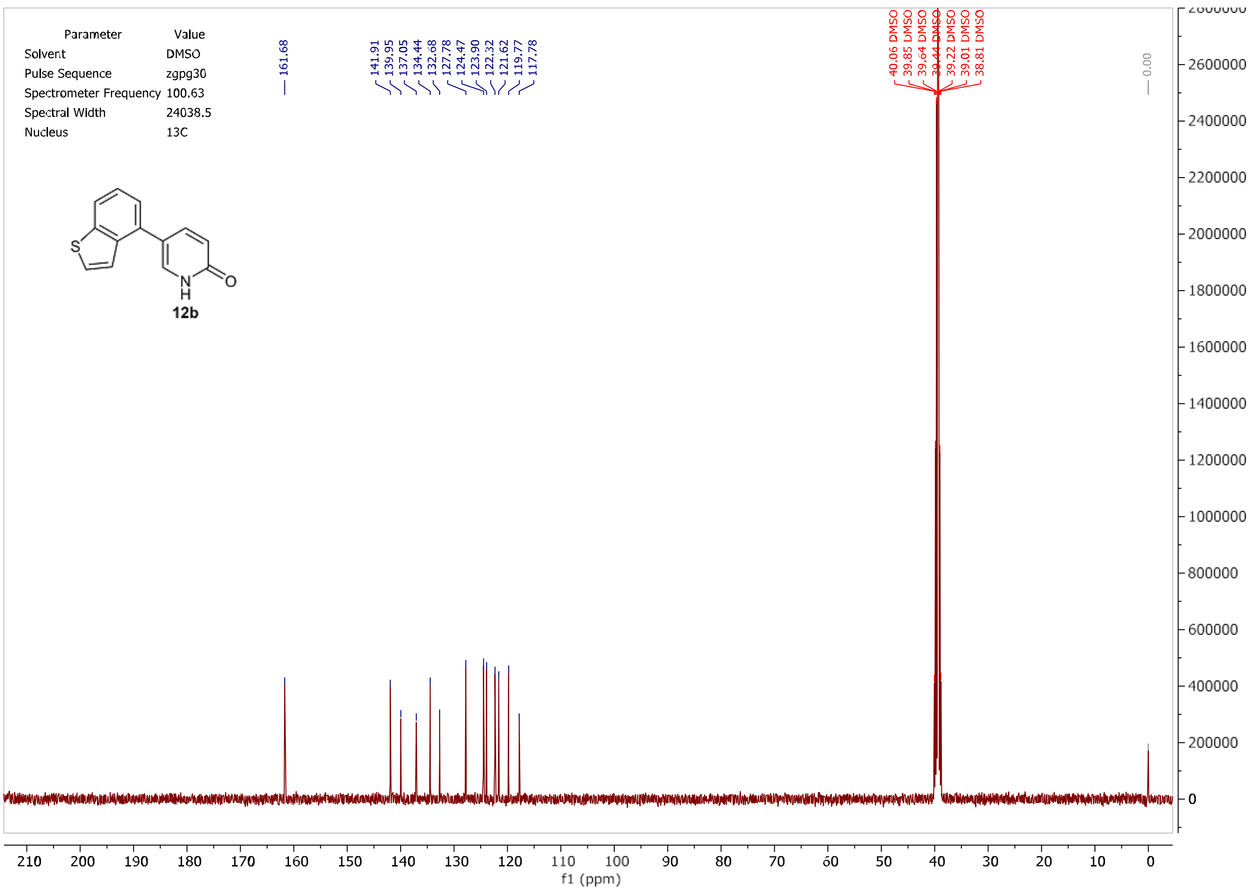

Figure S2.26. ^{1}H NMR (top) and ^{13}C NMR (bottom) spectra of compound **12b**

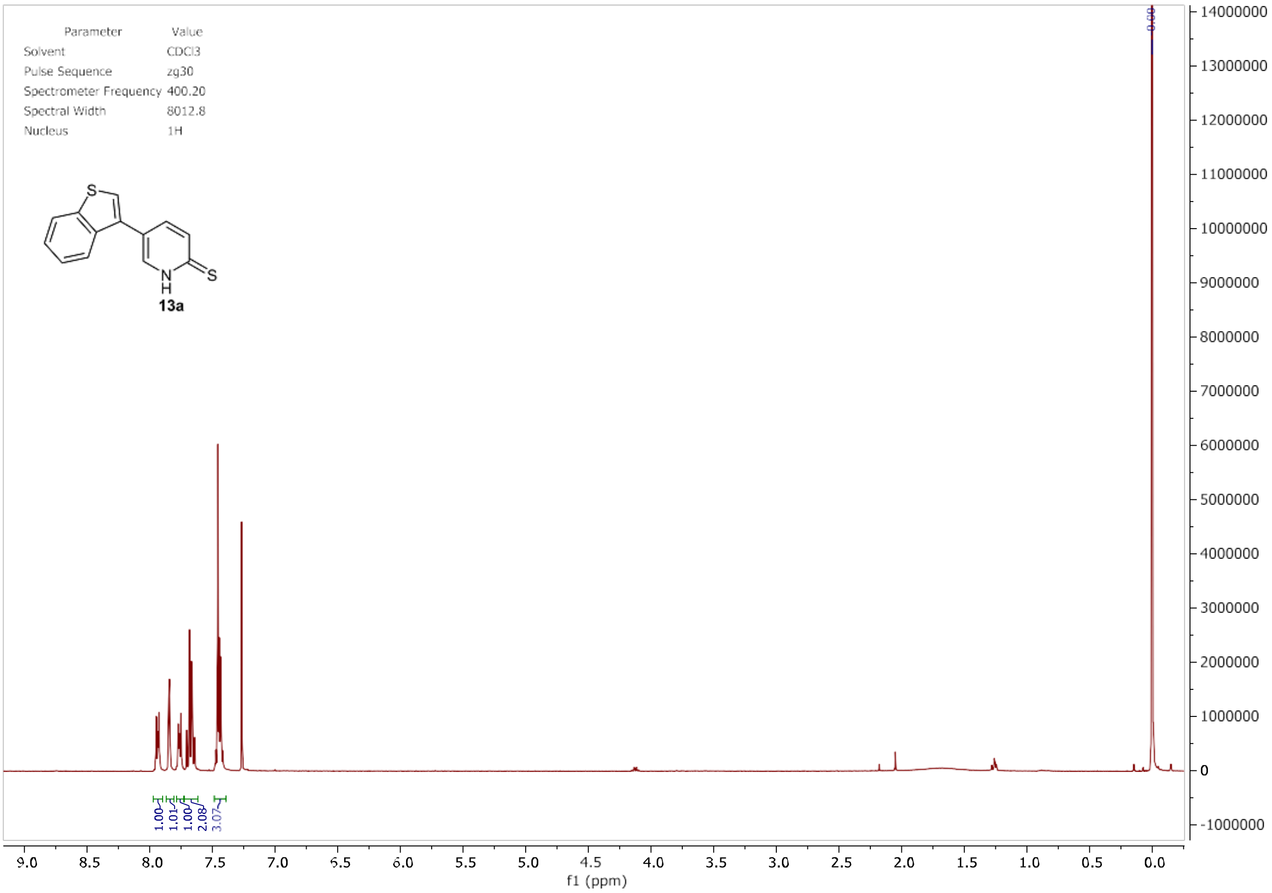

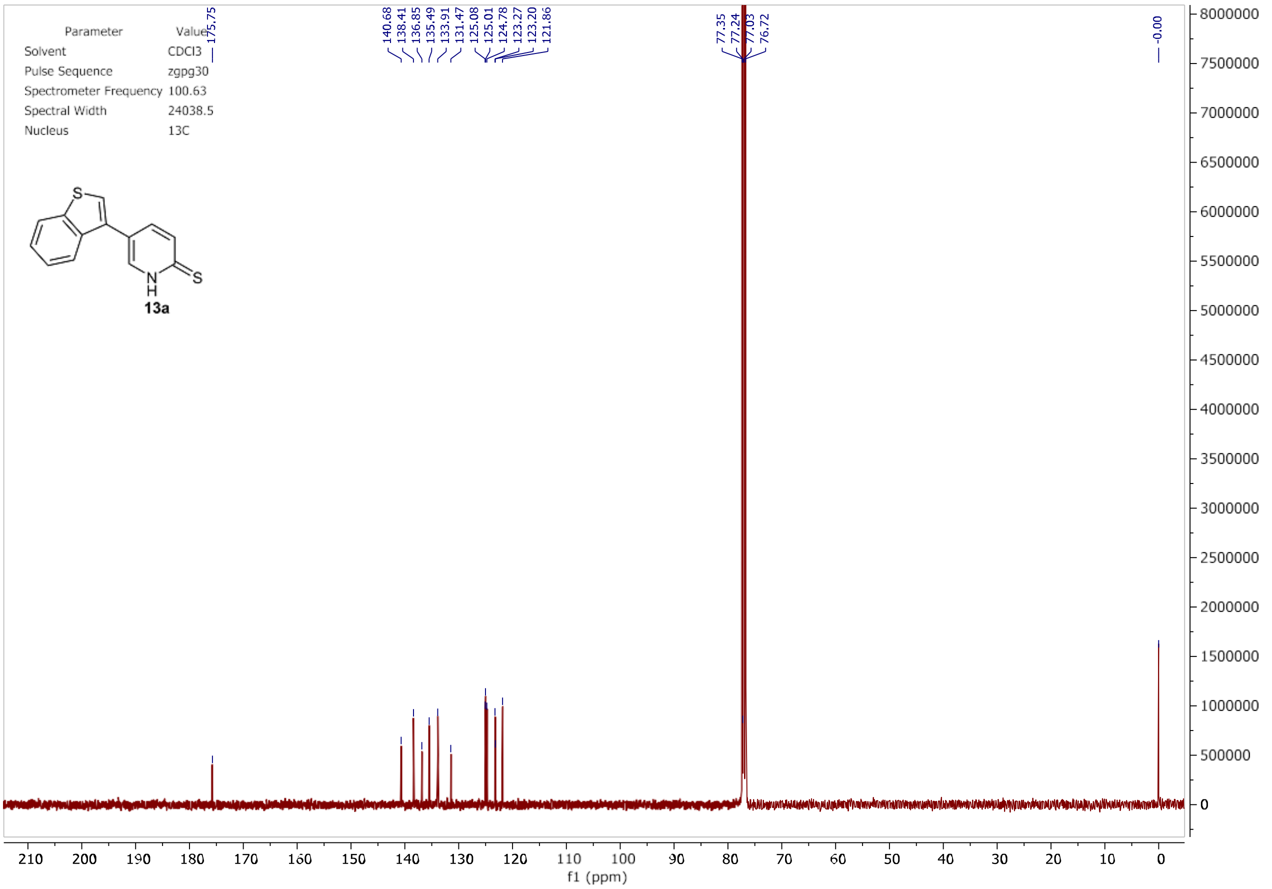

Figure S2.27. ^{1}H NMR (top) and ^{13}C NMR (bottom) spectra of compound **13a**

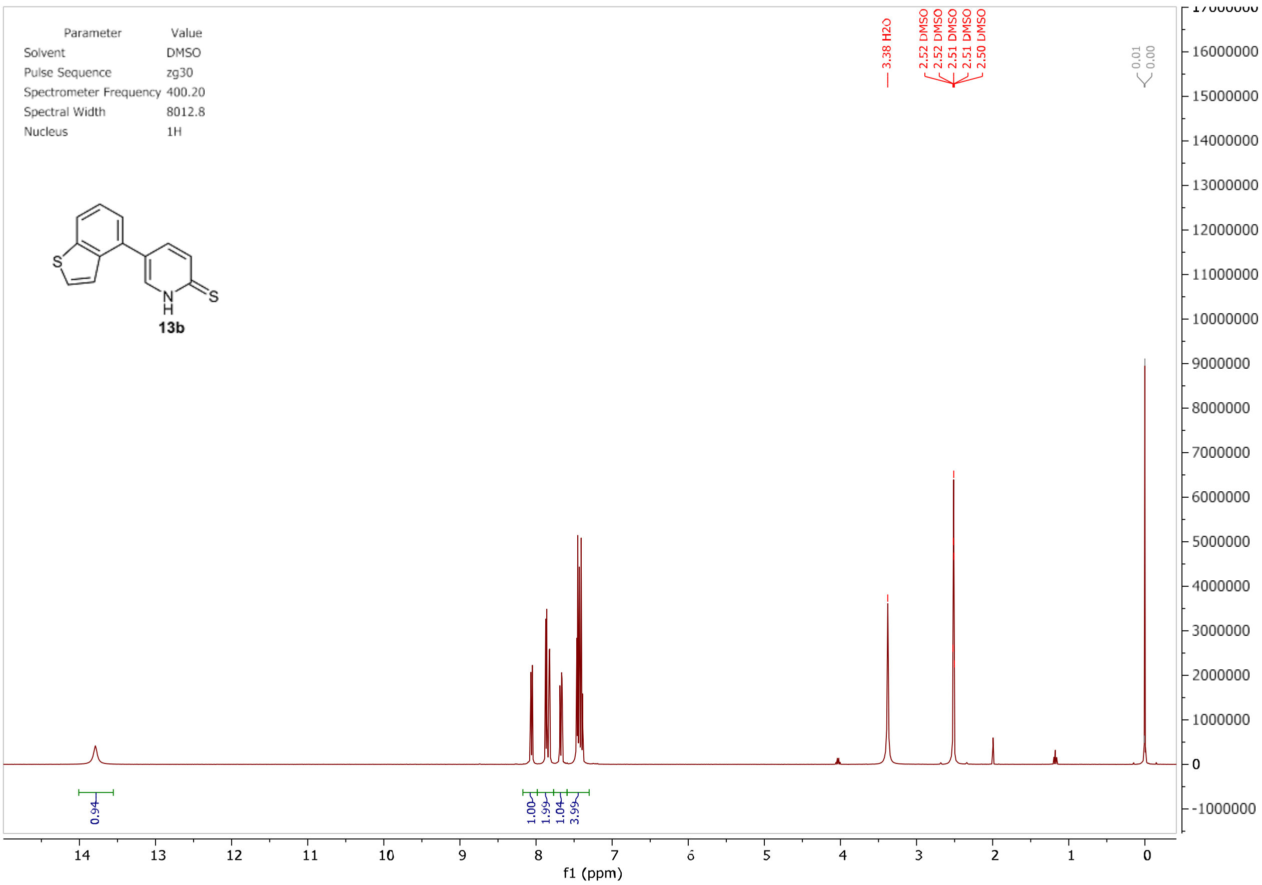

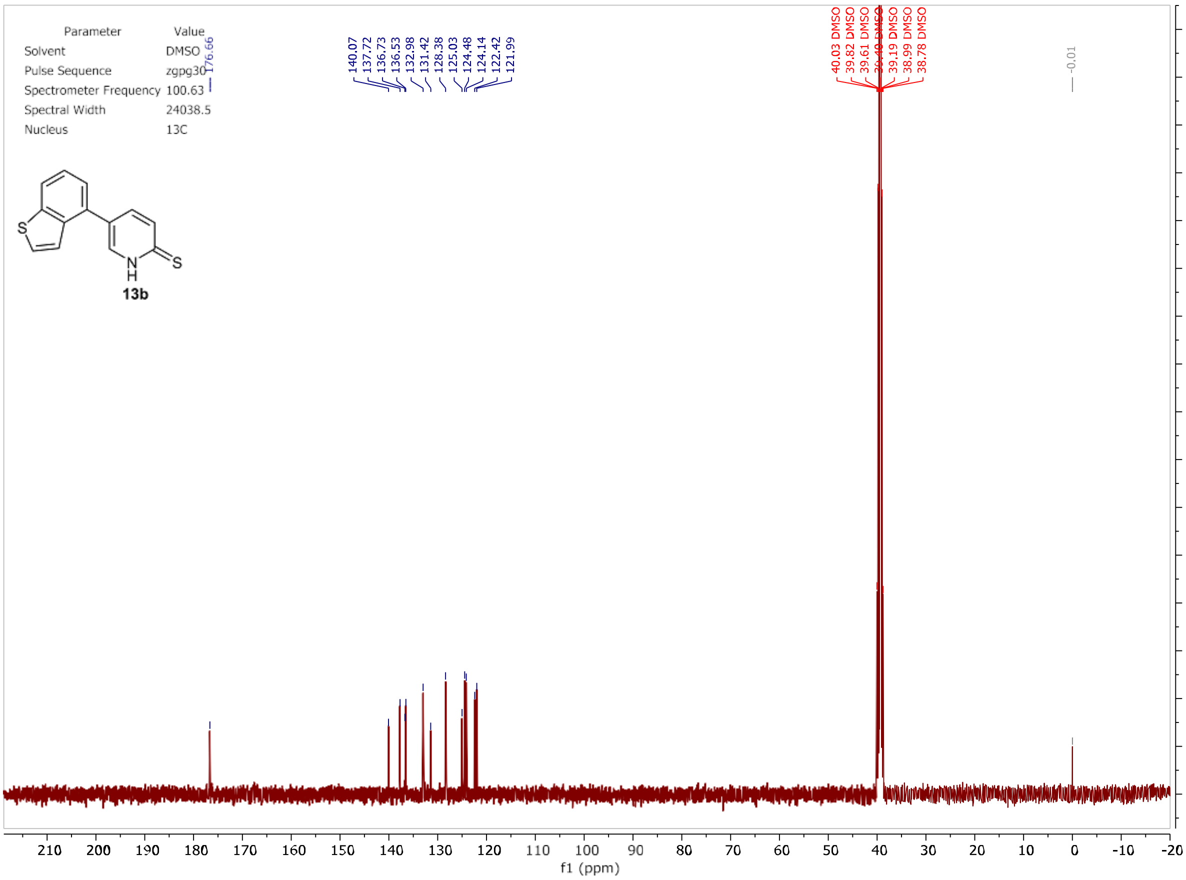

Figure S2.28. ^{1}H NMR (top) and ^{13}C NMR (bottom) spectra of compound **13b**

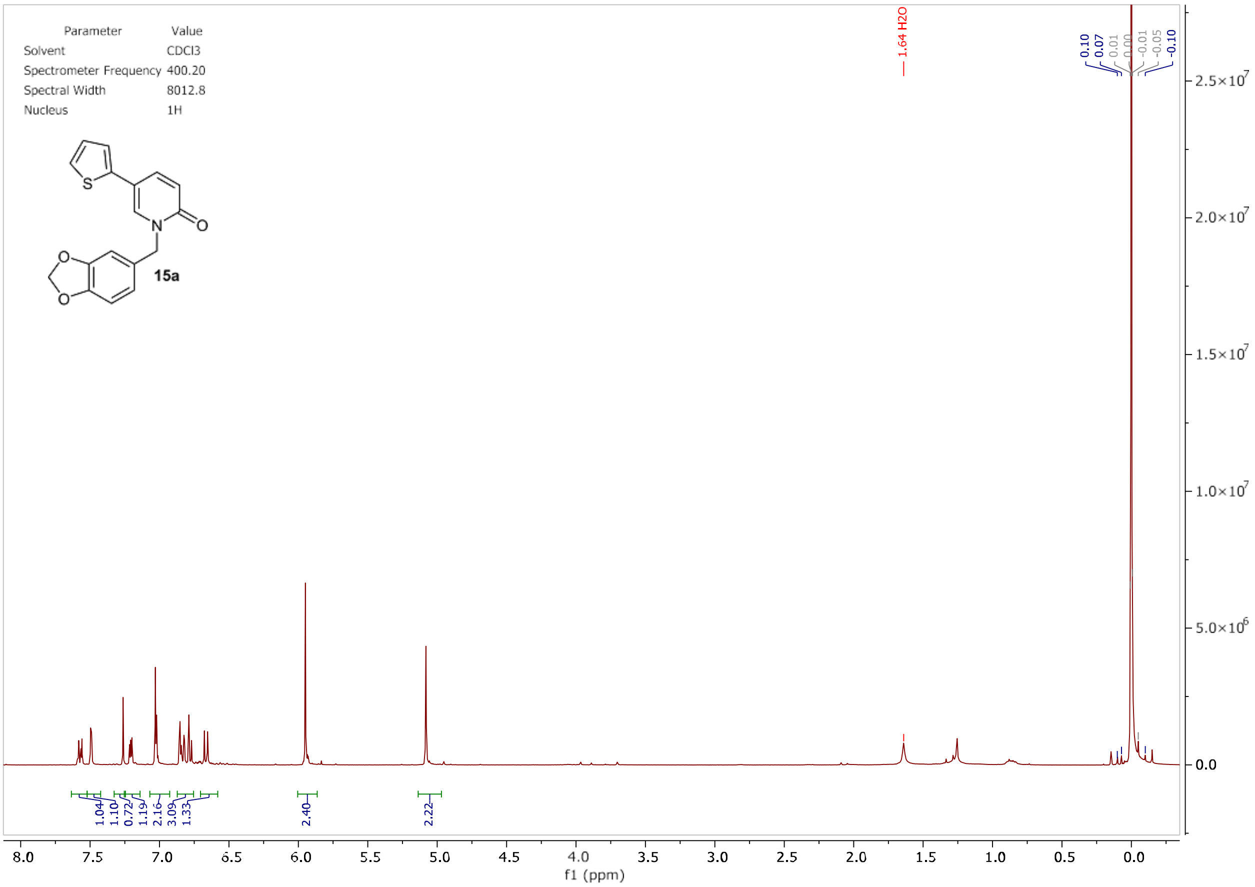

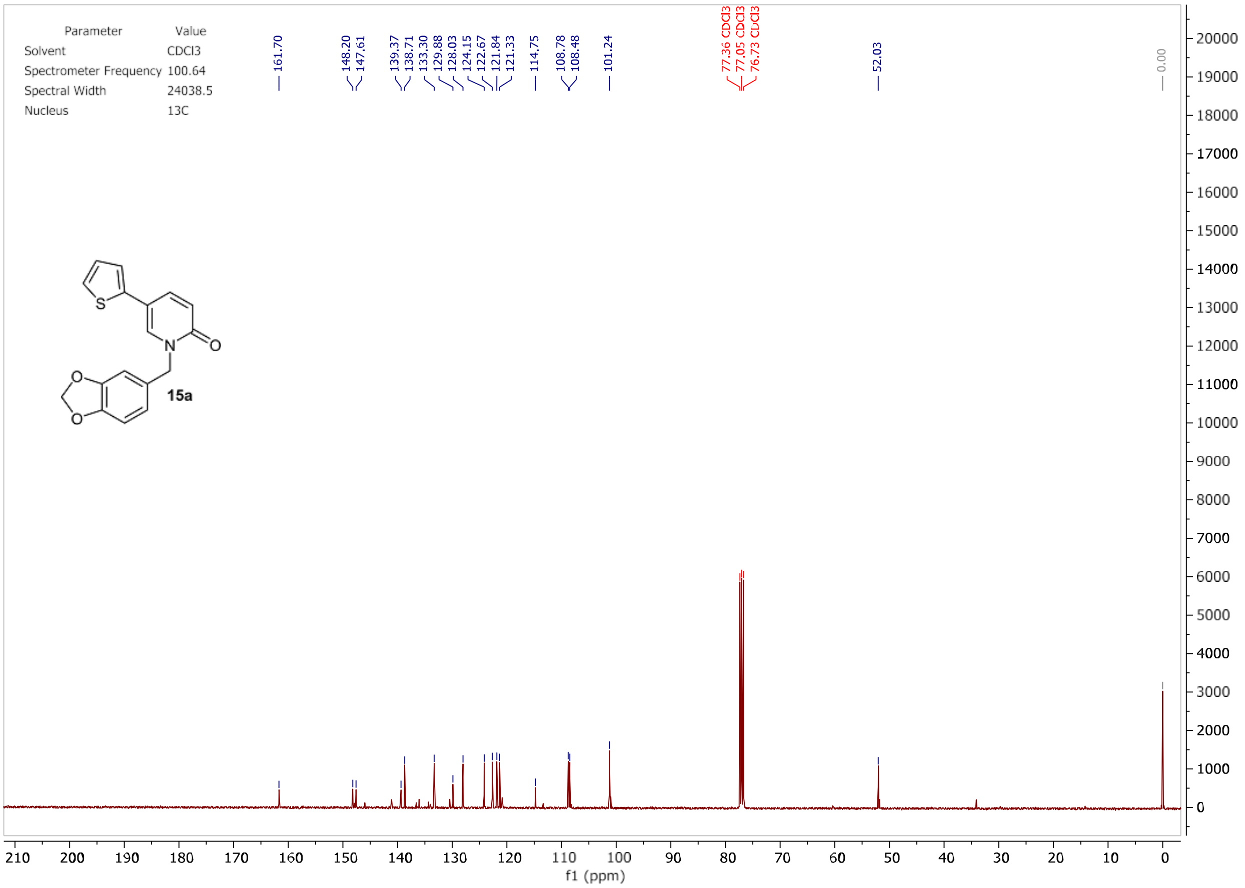

Figure S2.29. ^{1}H NMR (top) and ^{13}C NMR (bottom) spectra of compound **15a**

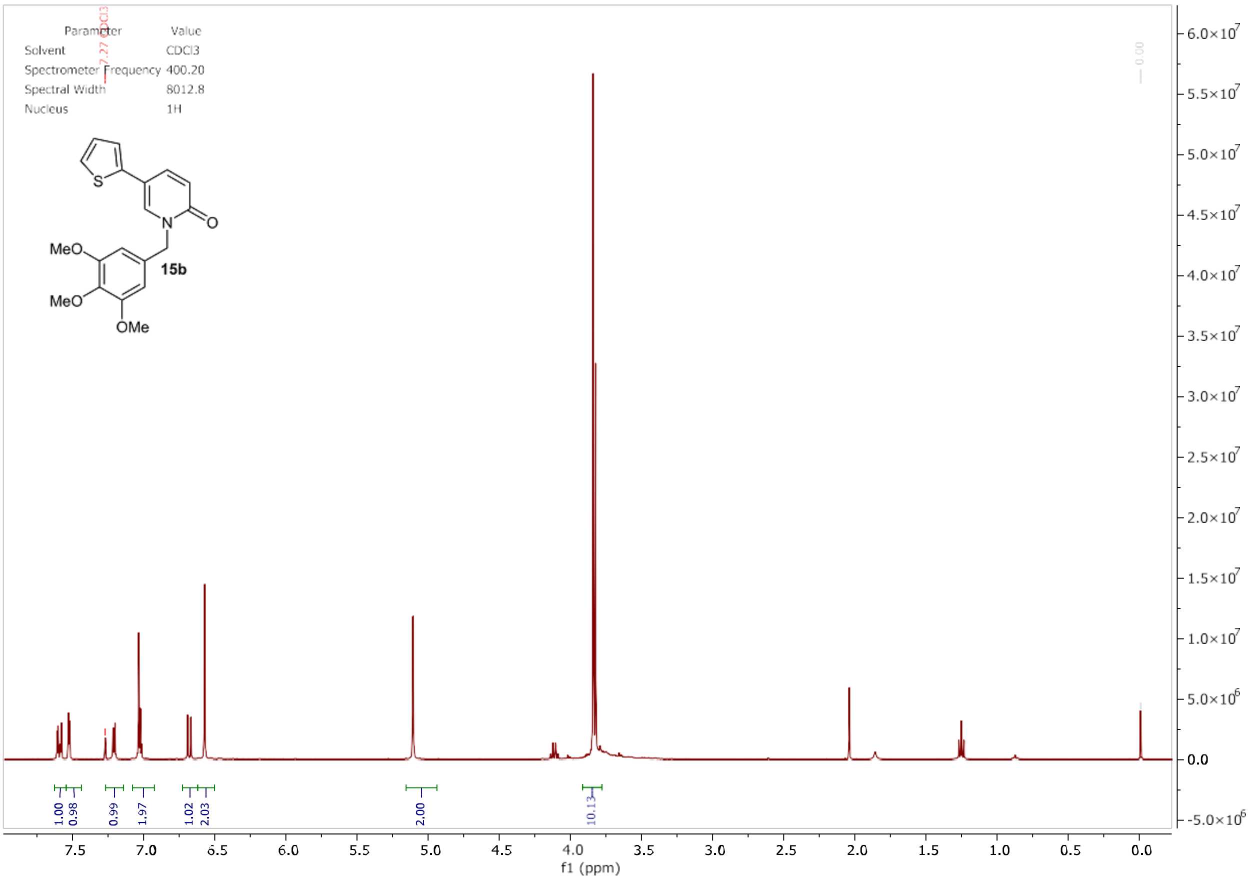

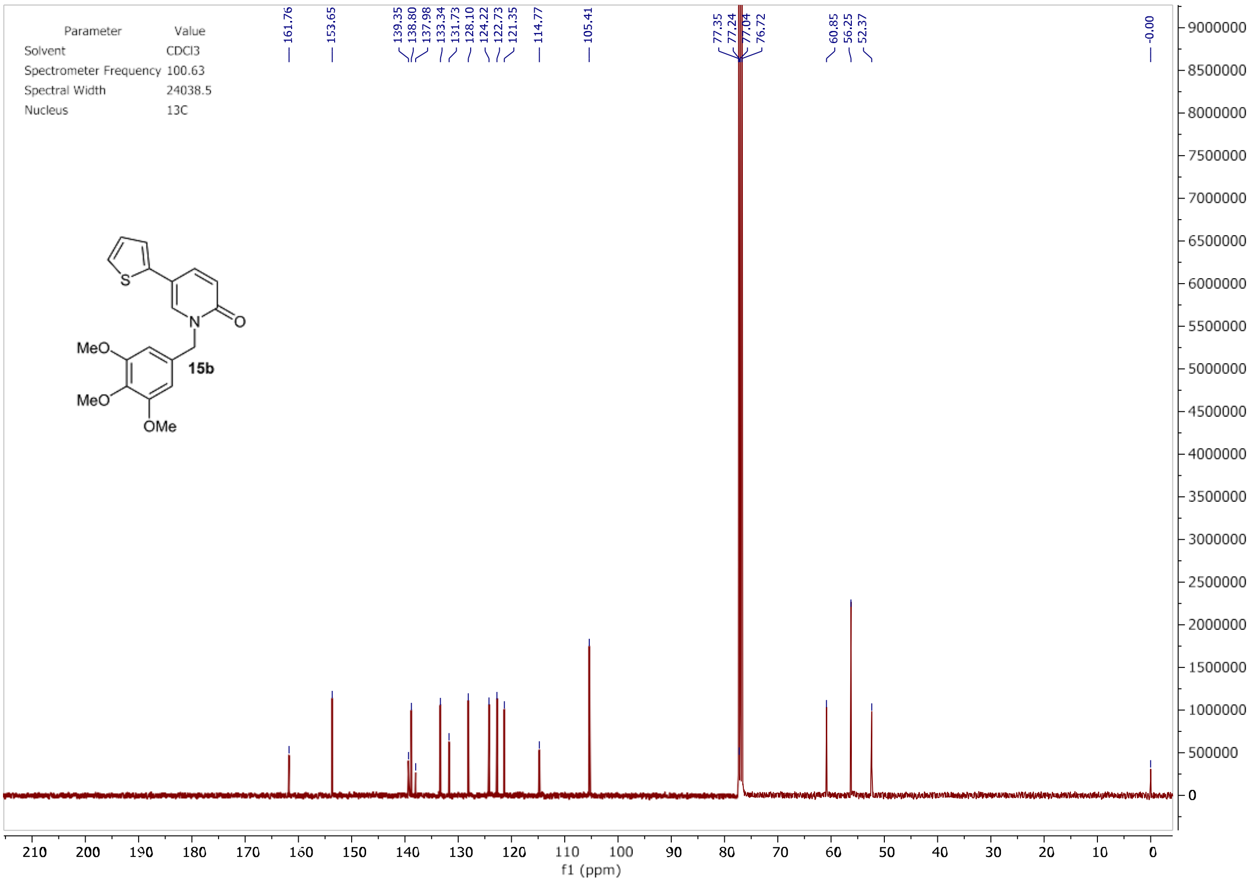

Figure S2.30. ^{1}H NMR (top) and ^{13}C NMR (bottom) spectra of compound **15b**

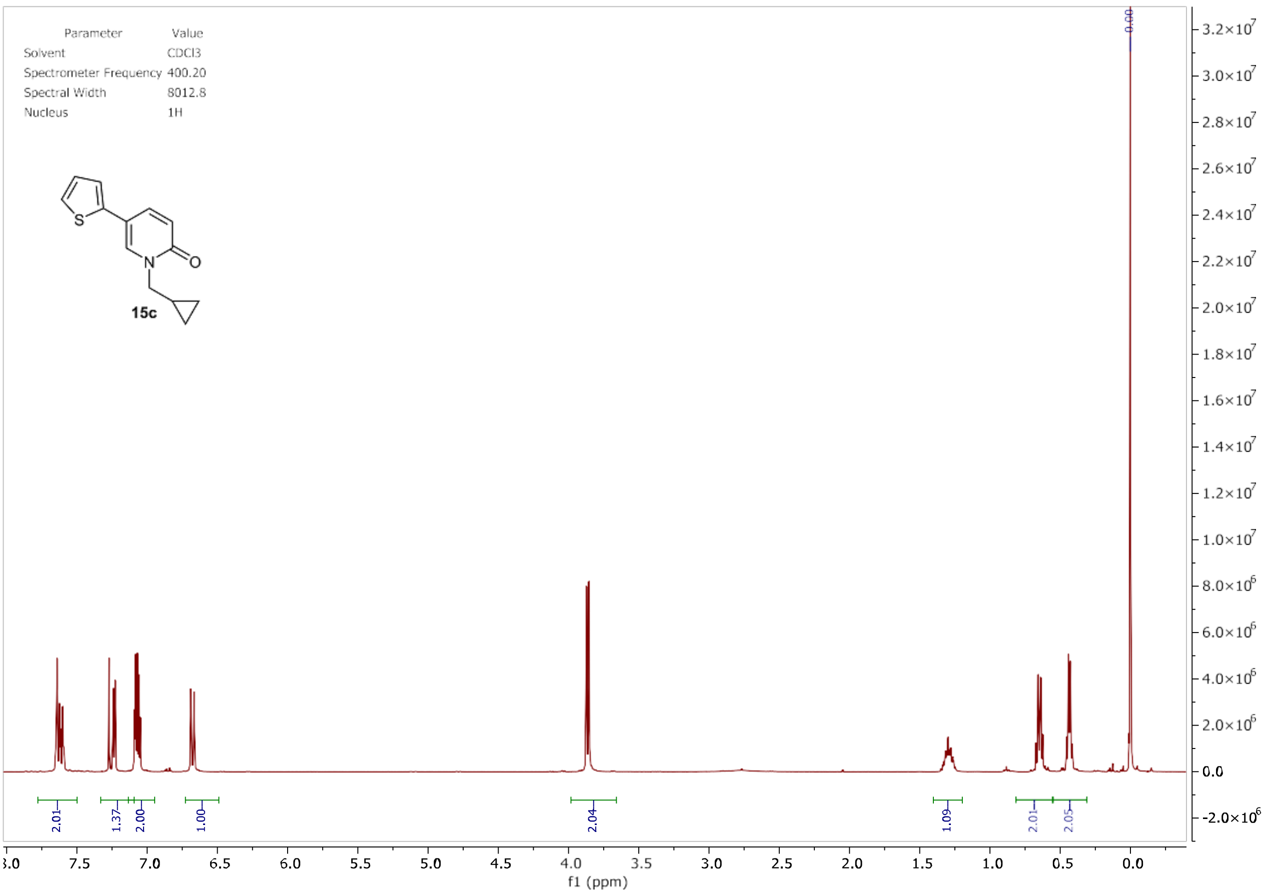

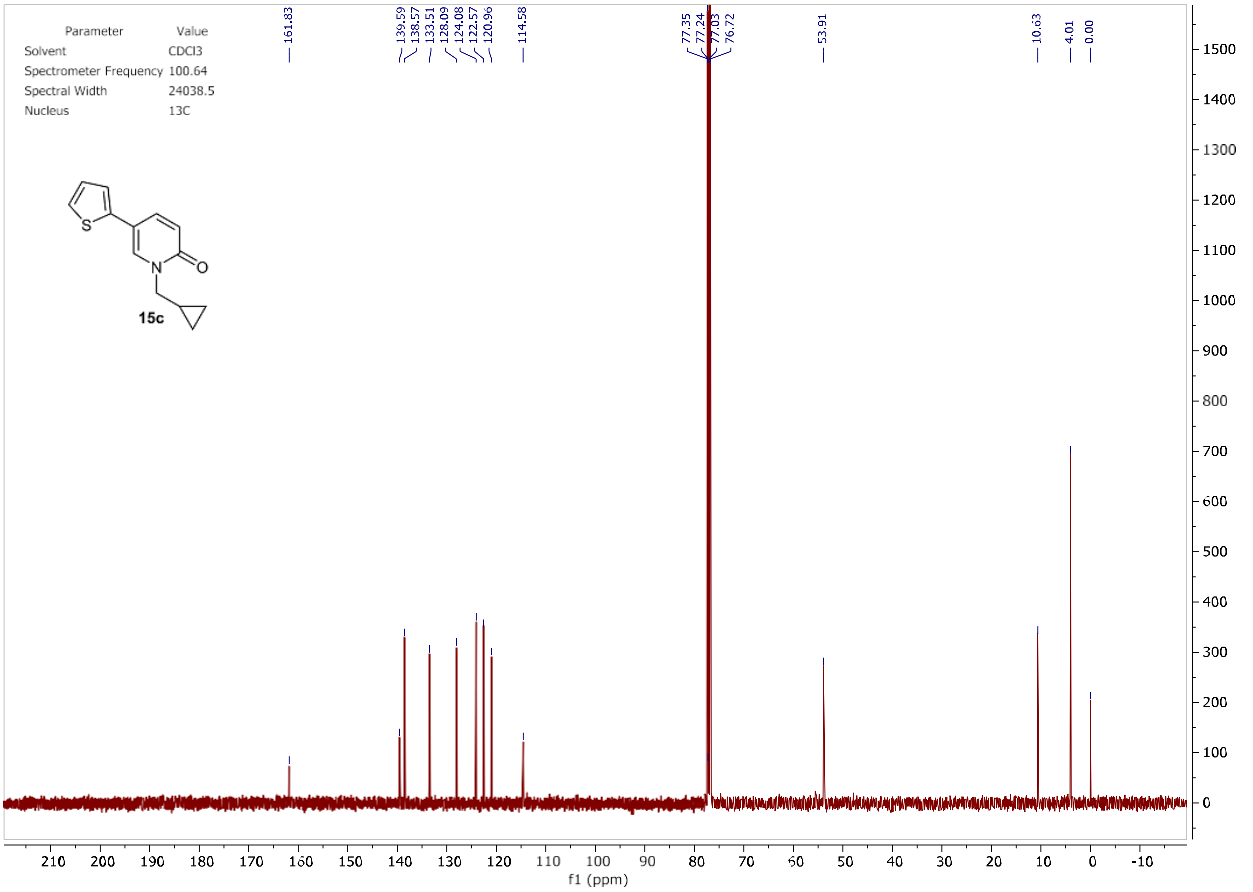

Figure S2.31. ^{1}H NMR (top) and ^{13}C NMR (bottom) spectra of compound **15c**

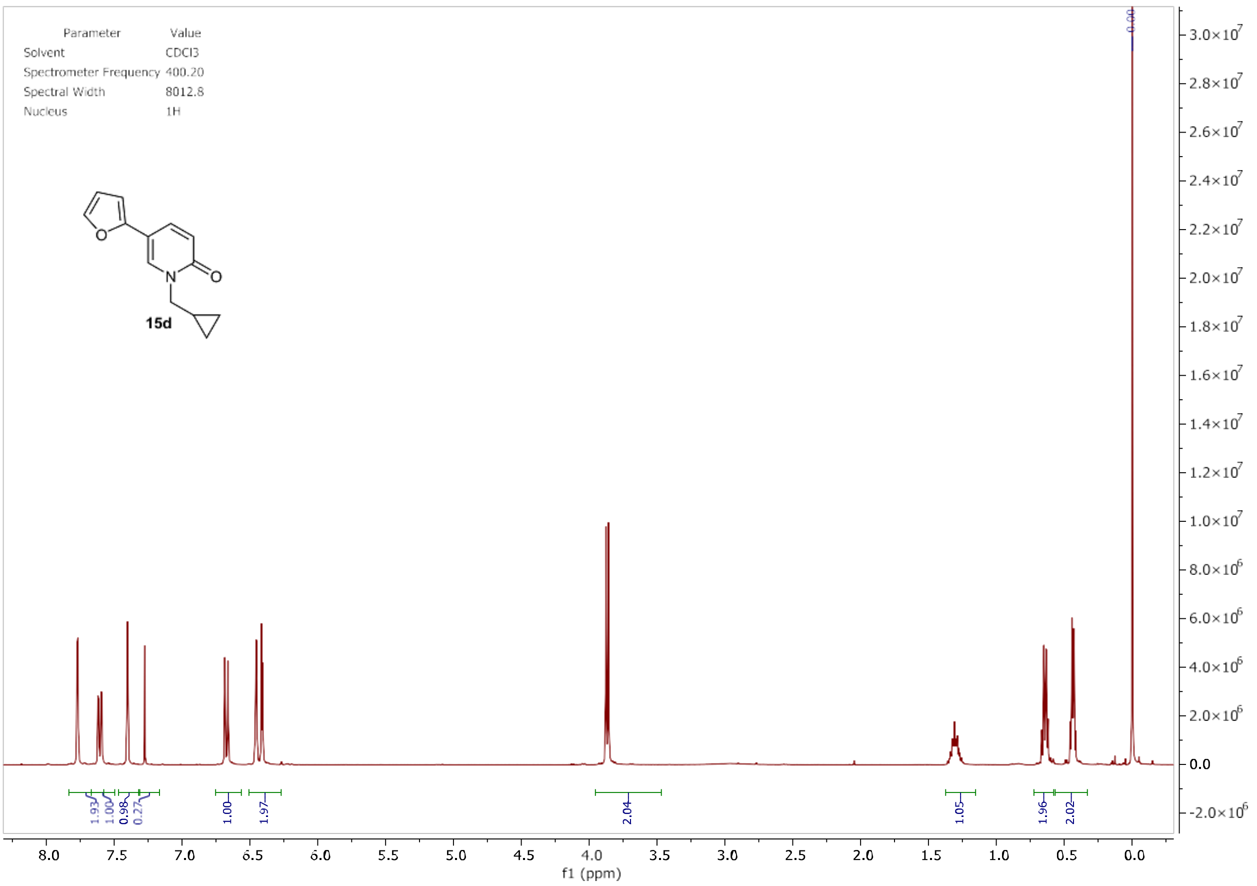

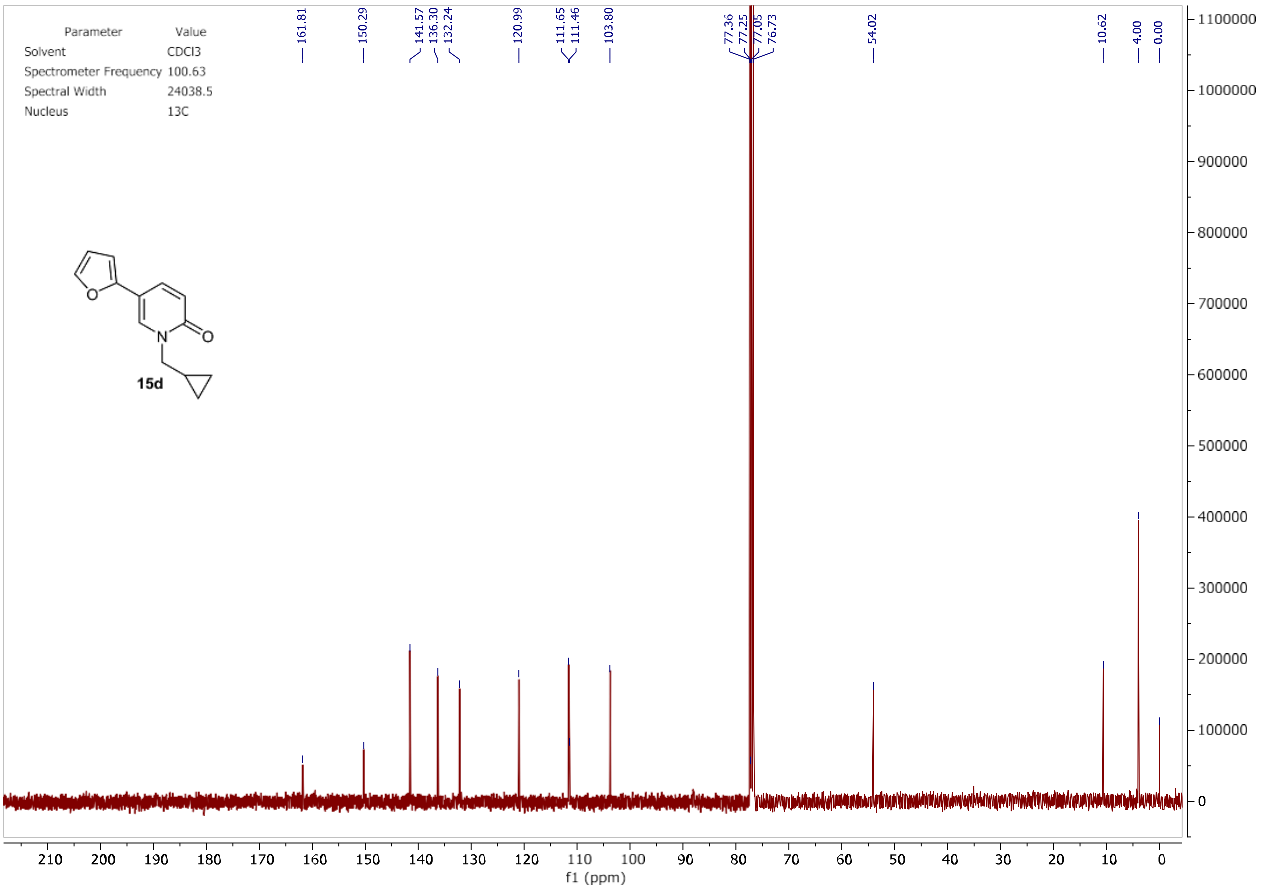

Figure S2.32. ^{1}H NMR (top) and ^{13}C NMR (bottom) spectra of compound **15d**

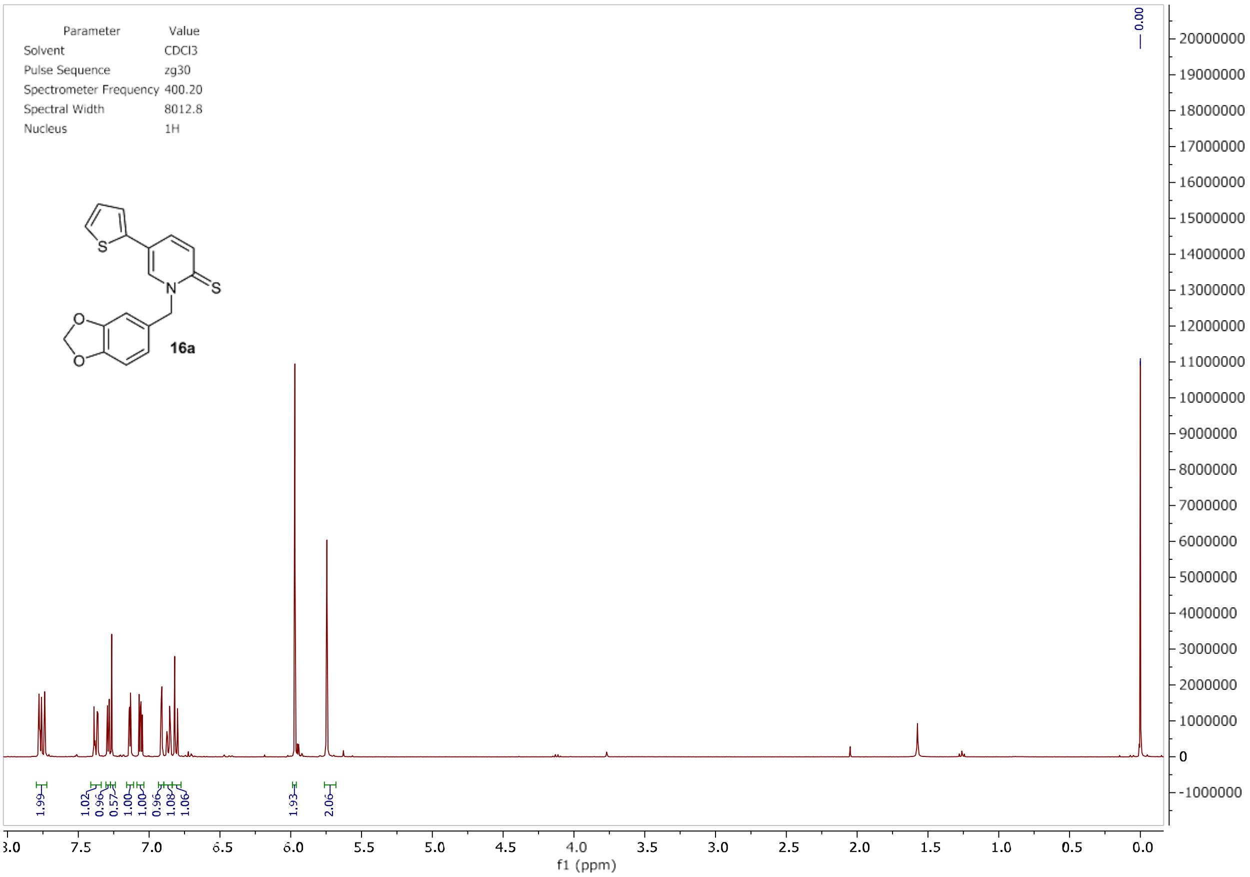

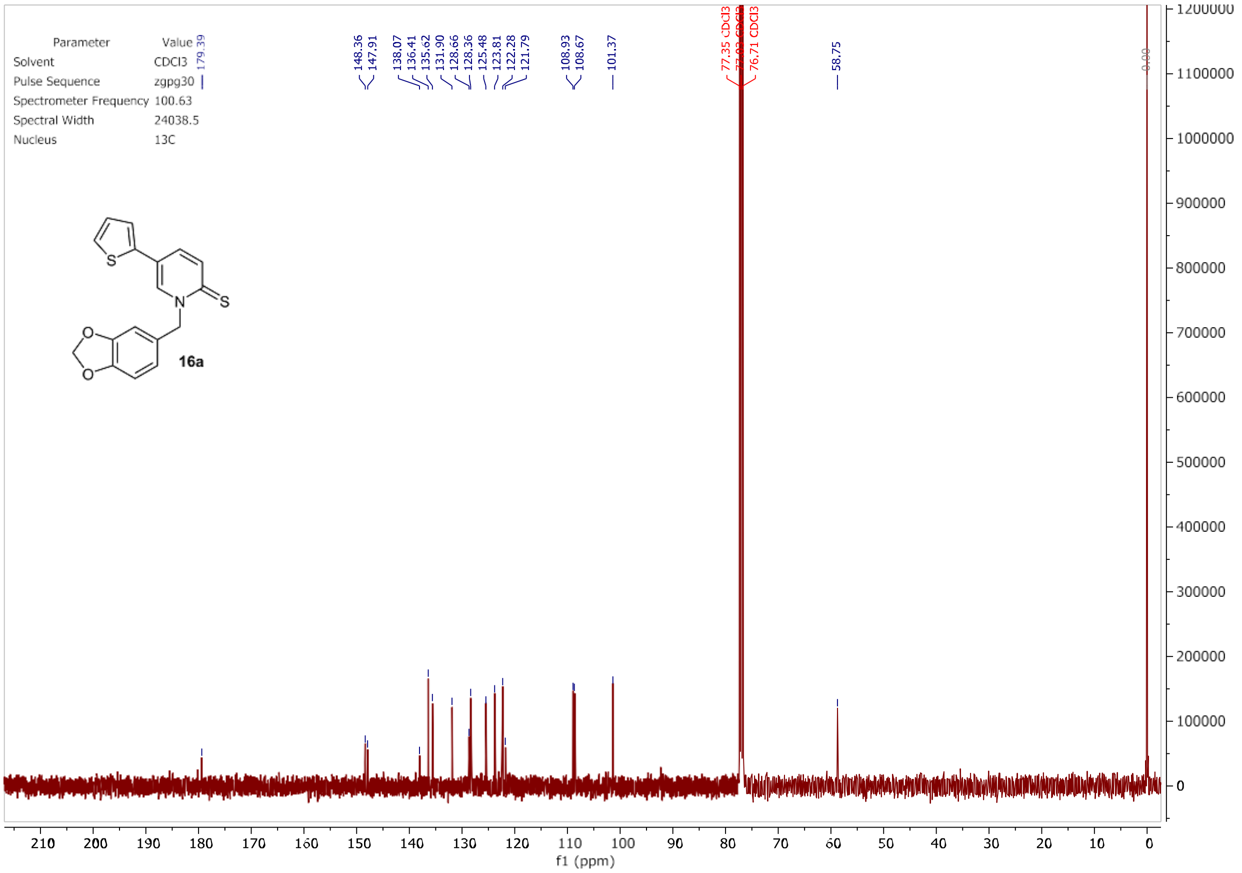

Figure S2.33. ^{1}H NMR (top) and ^{13}C NMR (bottom) spectra of compound **16a**

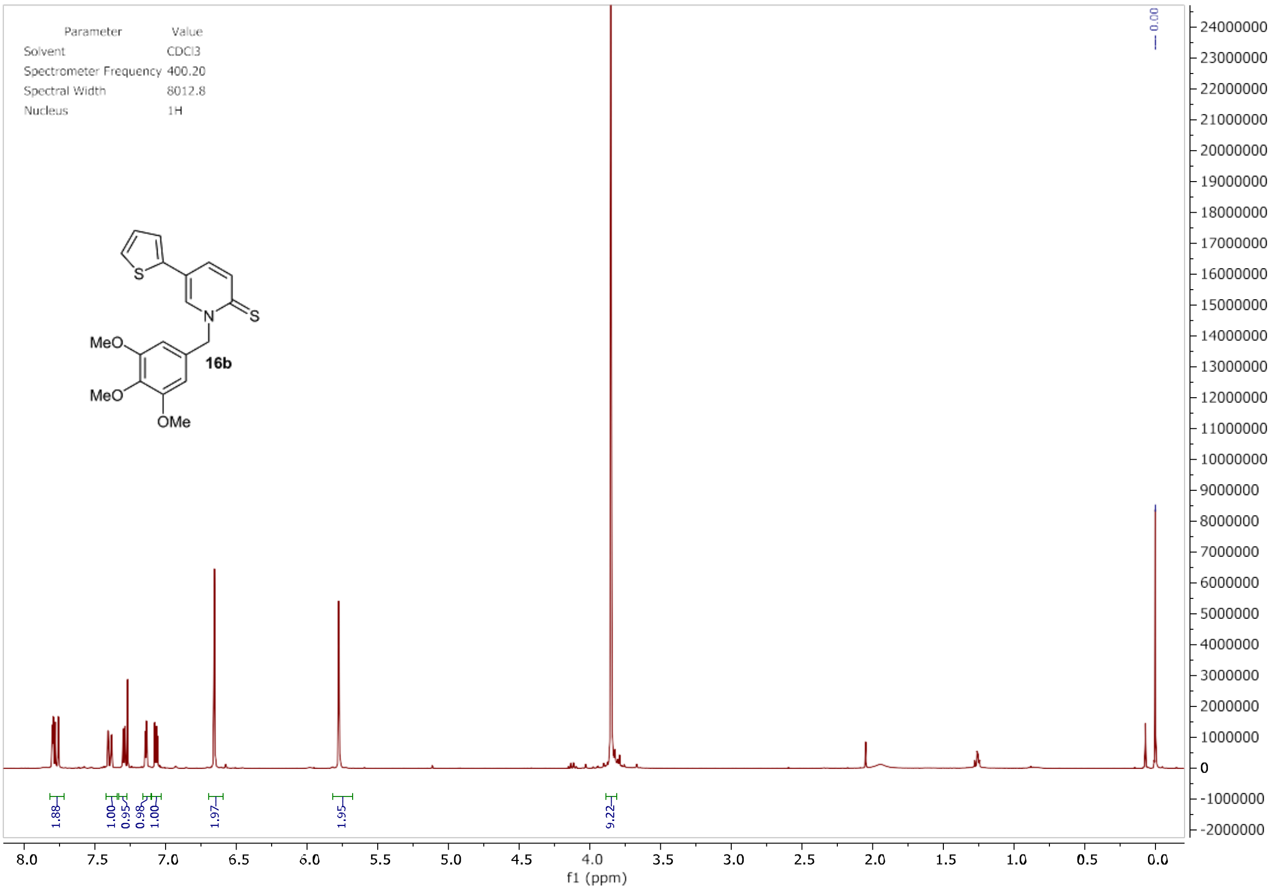

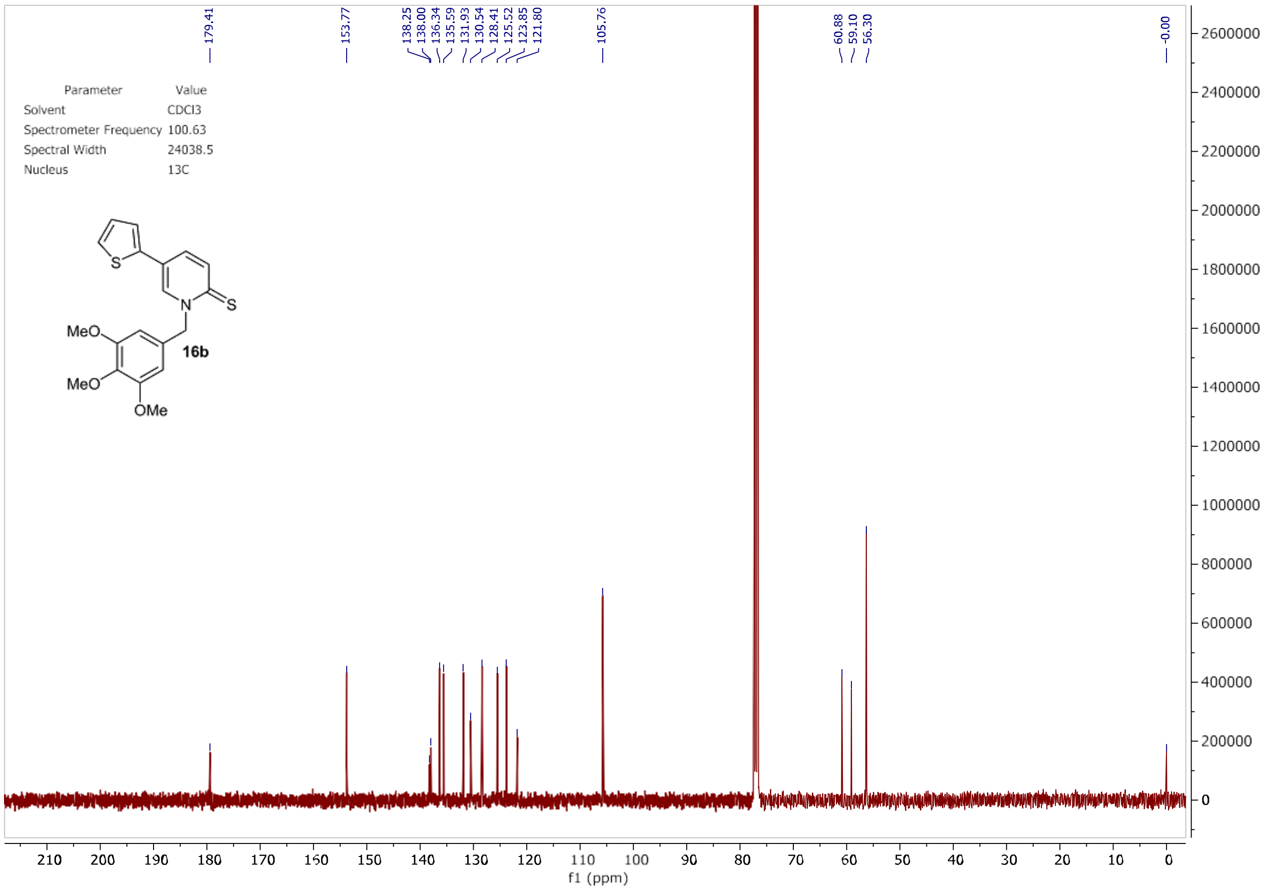

Figure S2.34. ^{1}H NMR (top) and ^{13}C NMR (bottom) spectra of compound **16b**

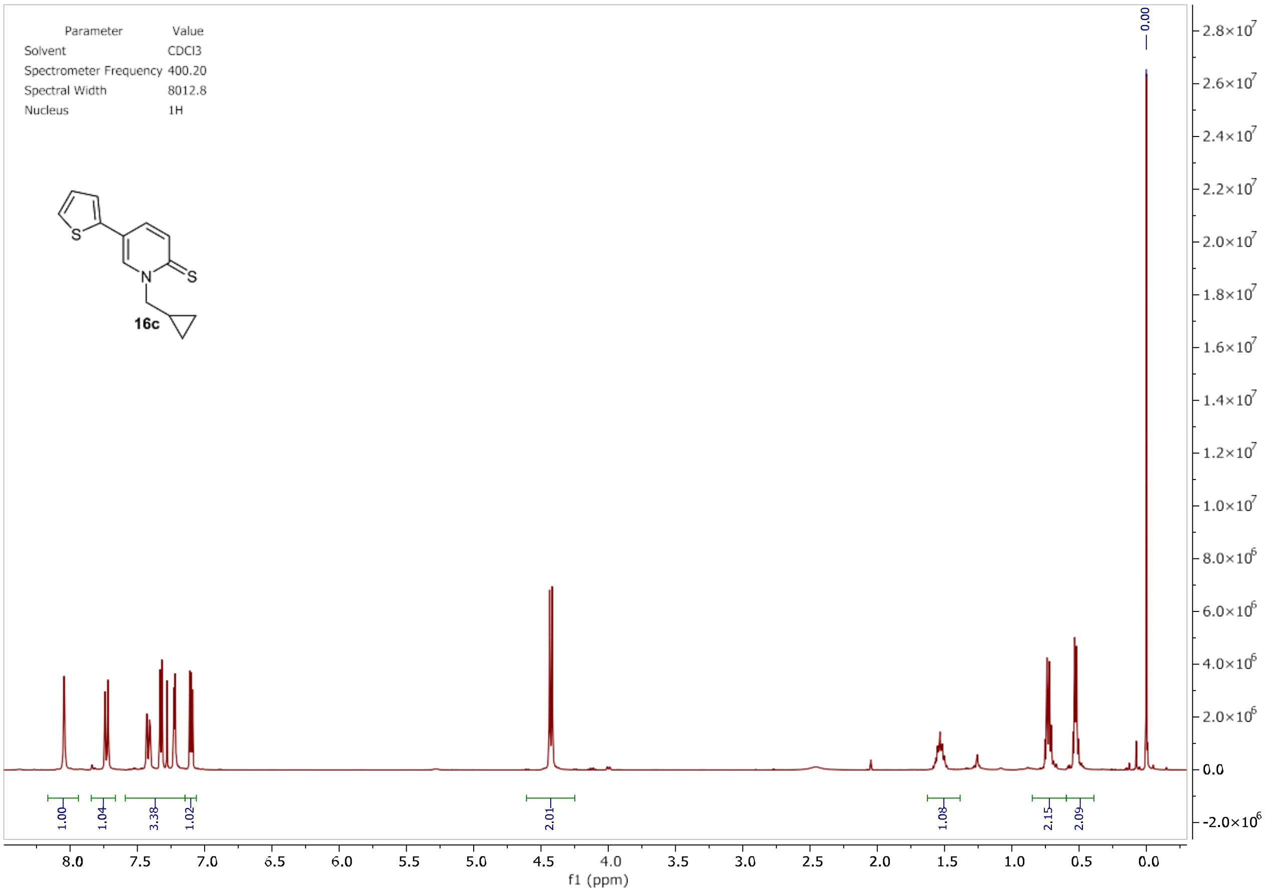

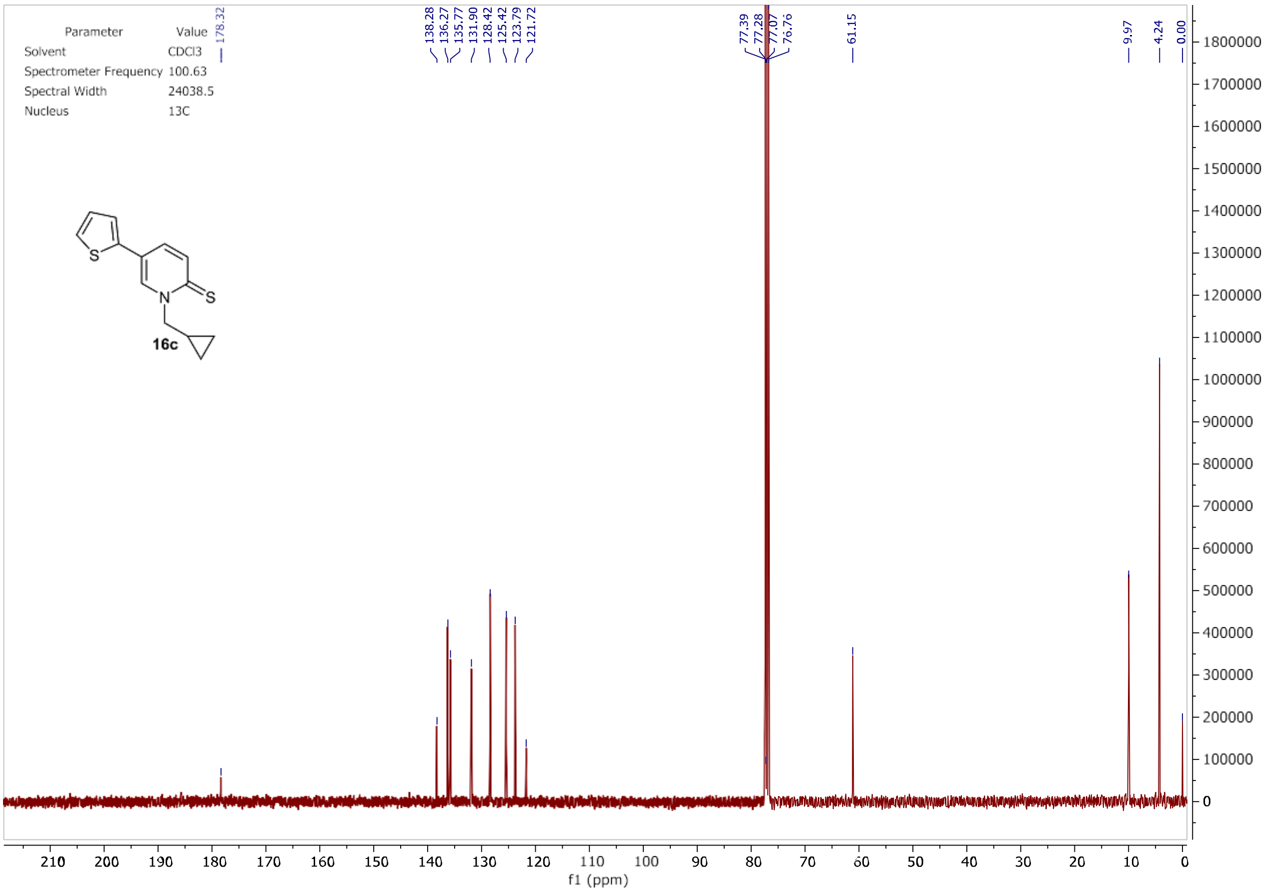

Figure S2.35. ^{1}H NMR (top) and ^{13}C NMR (bottom) spectra of compound **16c**

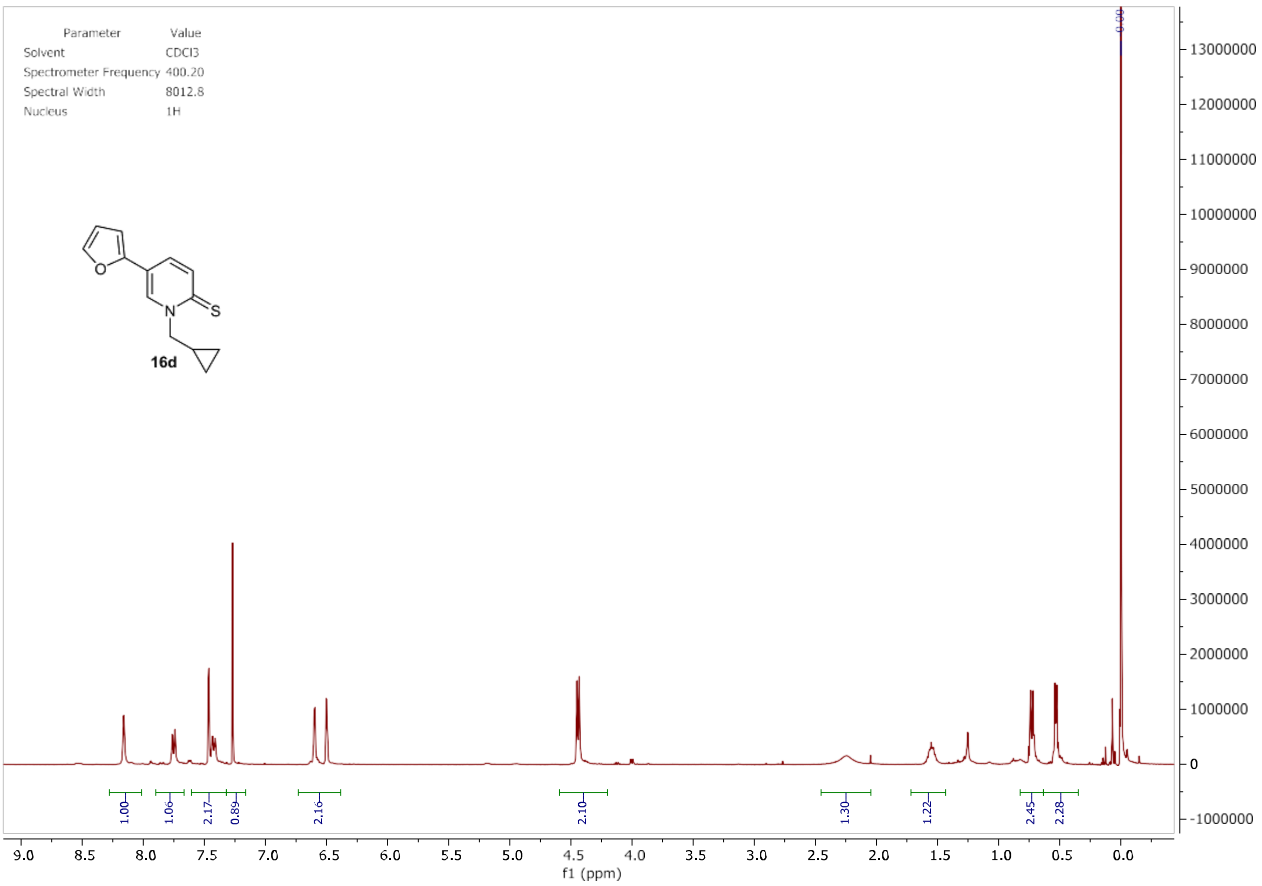

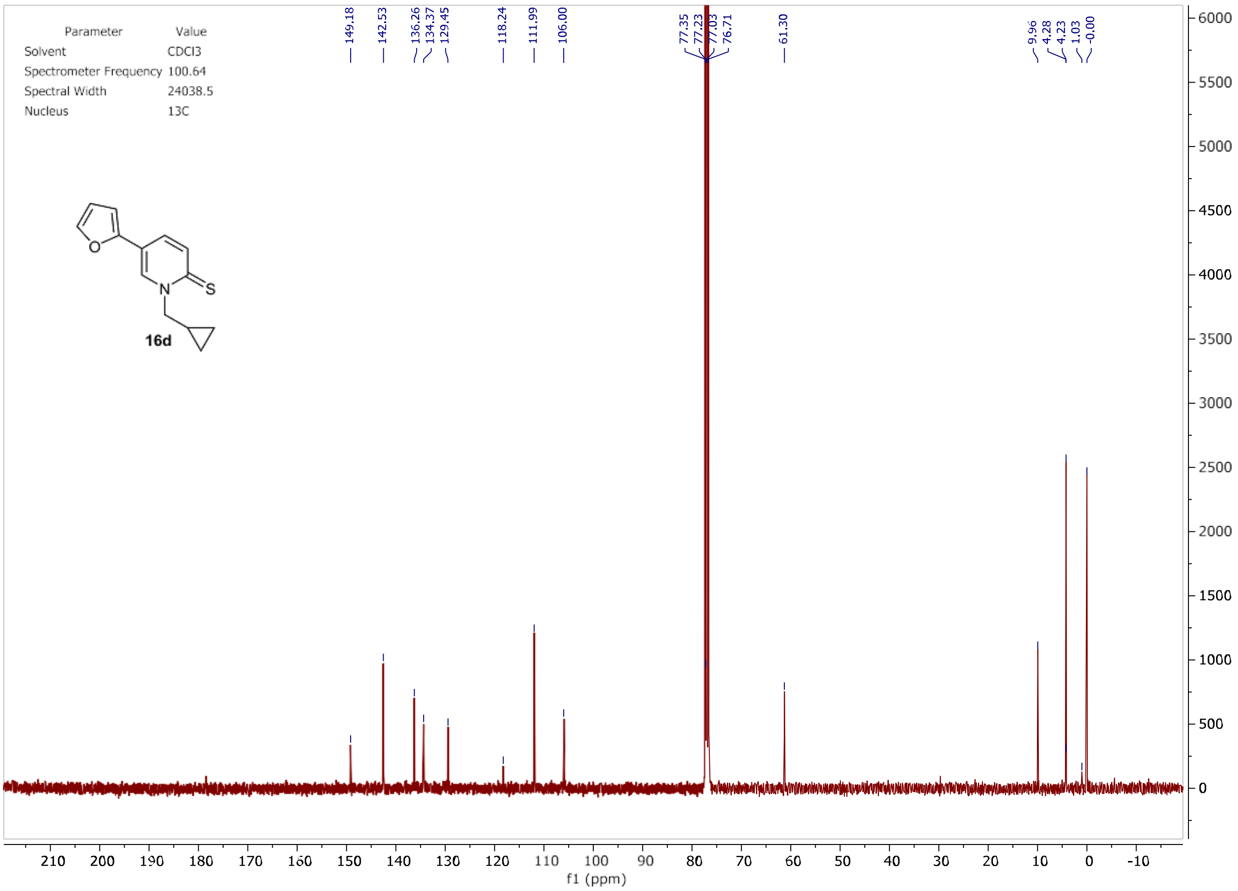

Figure S2.36. ^{1}H NMR (top) and ^{13}C NMR (bottom) spectra of compound **16d**

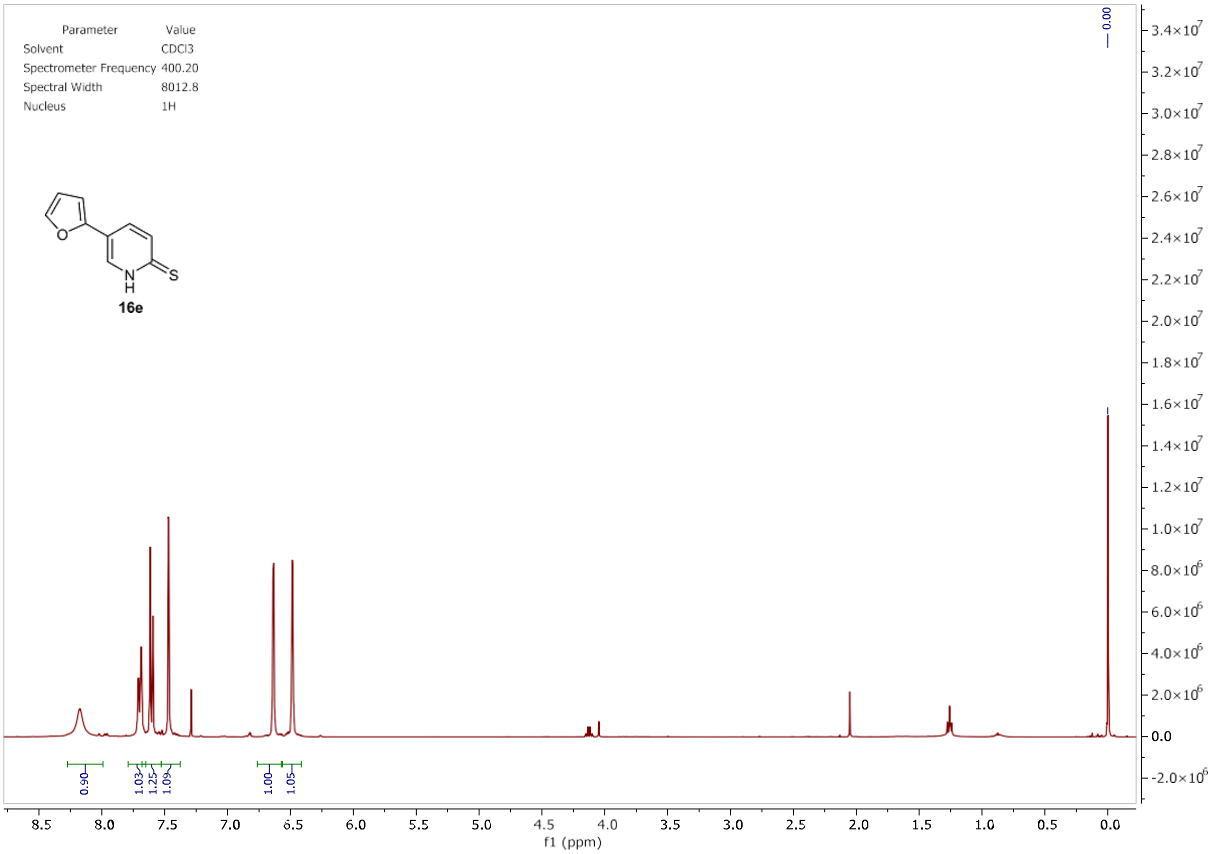

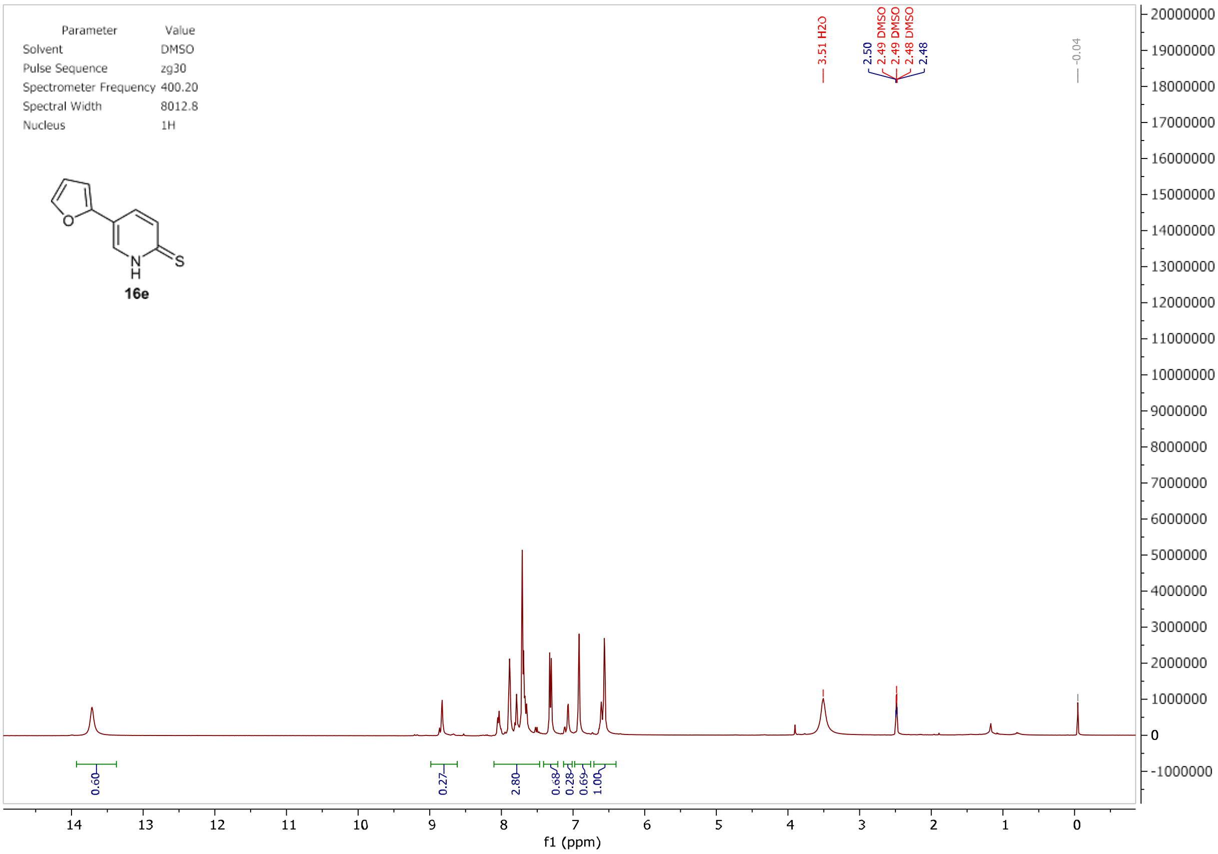

Figure S2.37. ^{1}H NMR (in $CDCl_3$, top) and ^{1}H NMR (in DMSO-d_6, bottom) spectra of compound **16e**

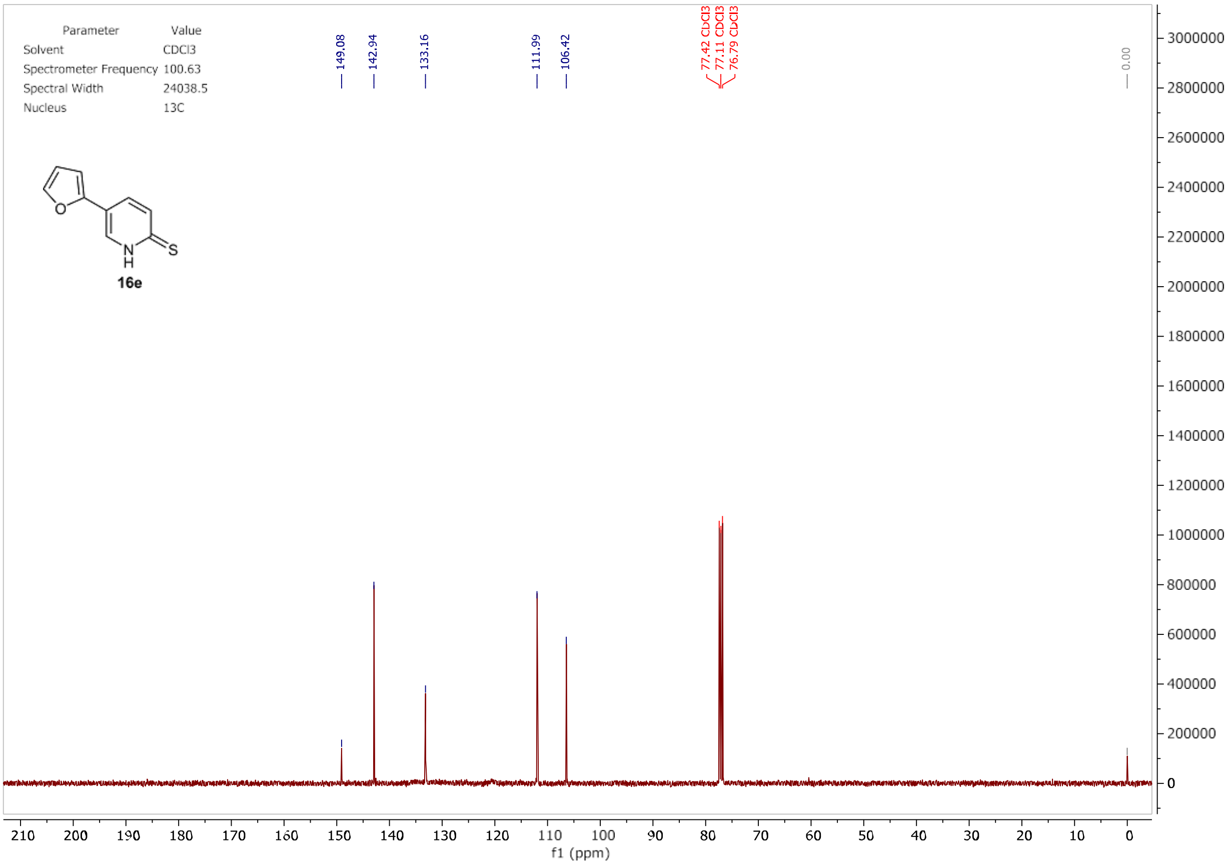

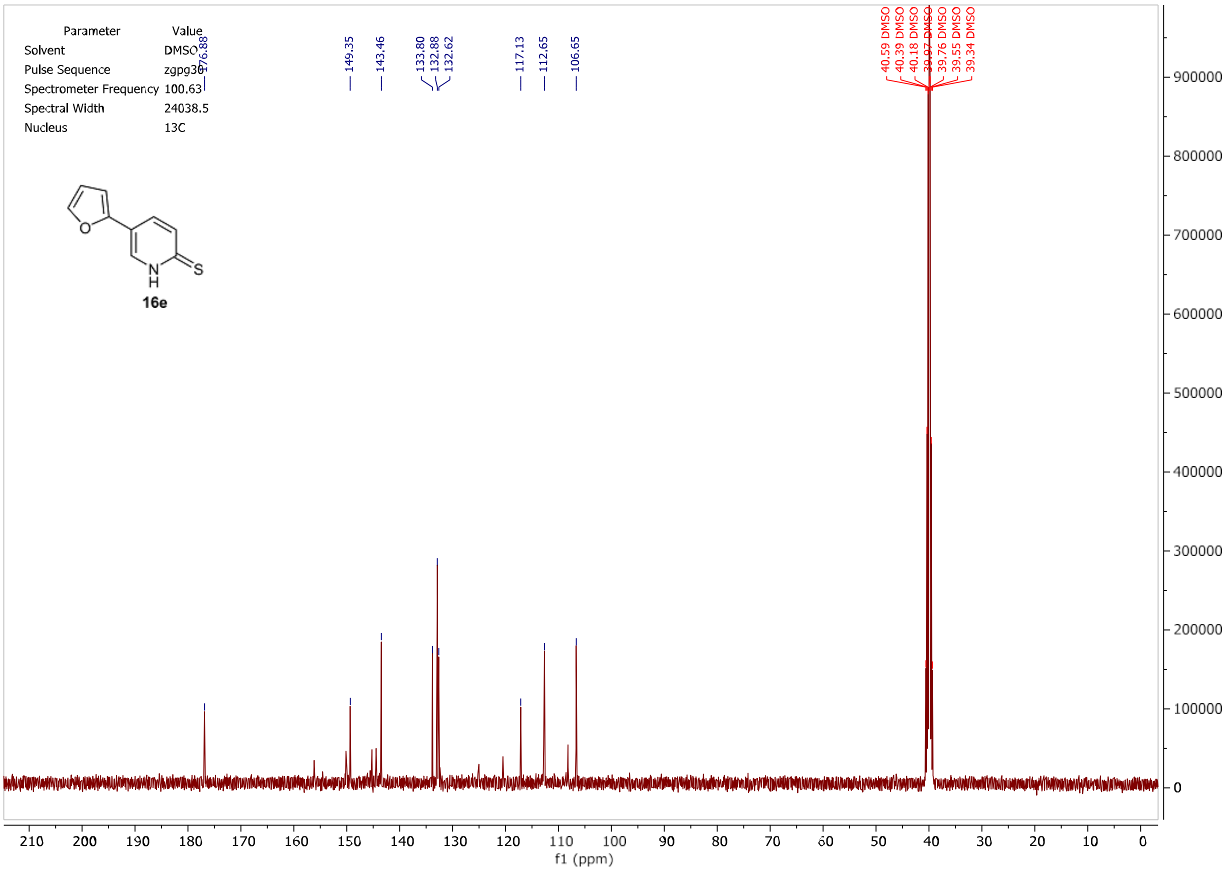

Figure S2.38. ^{13}C NMR (in $CDCl_3$, top) and ^{13}C NMR (in DMSO-d_6, bottom) spectra of compound **16e**

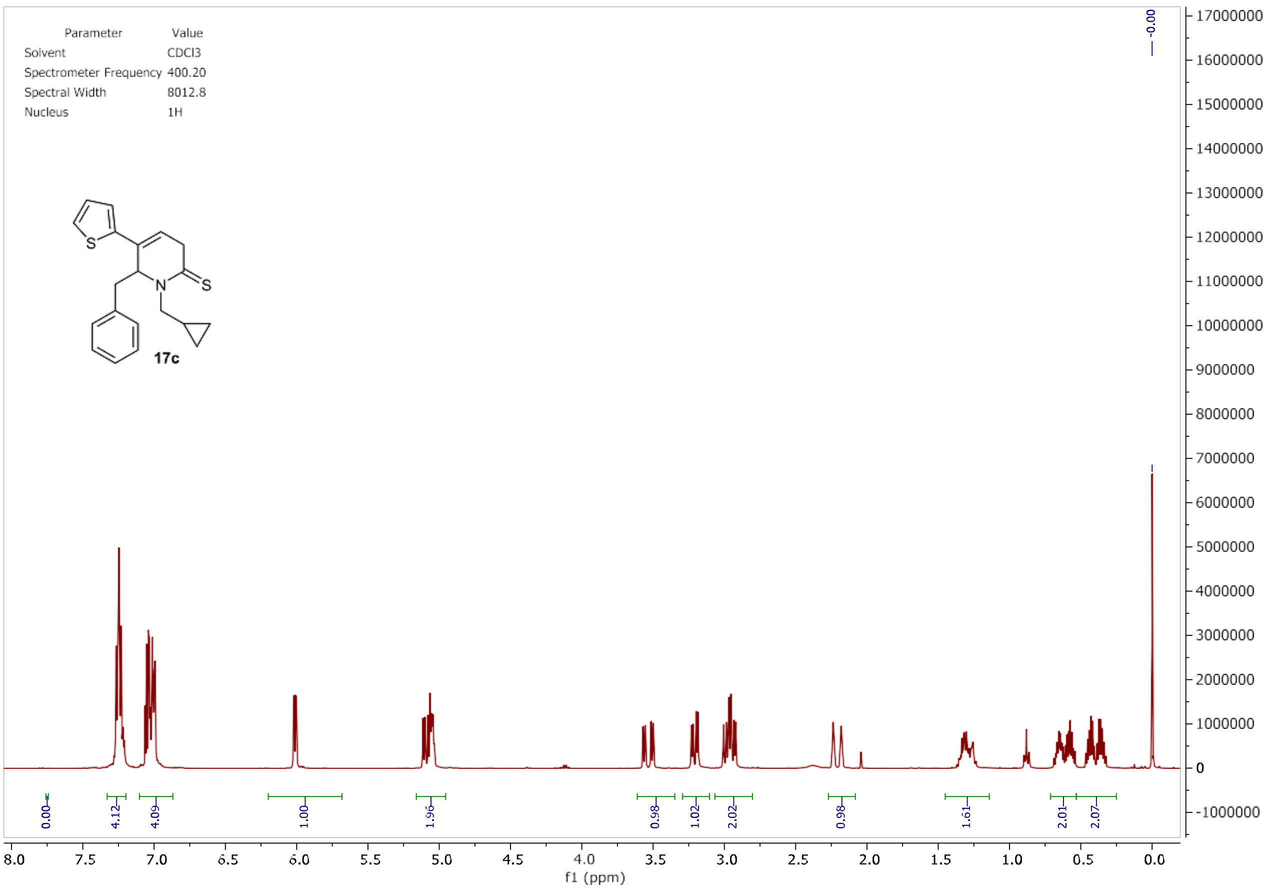

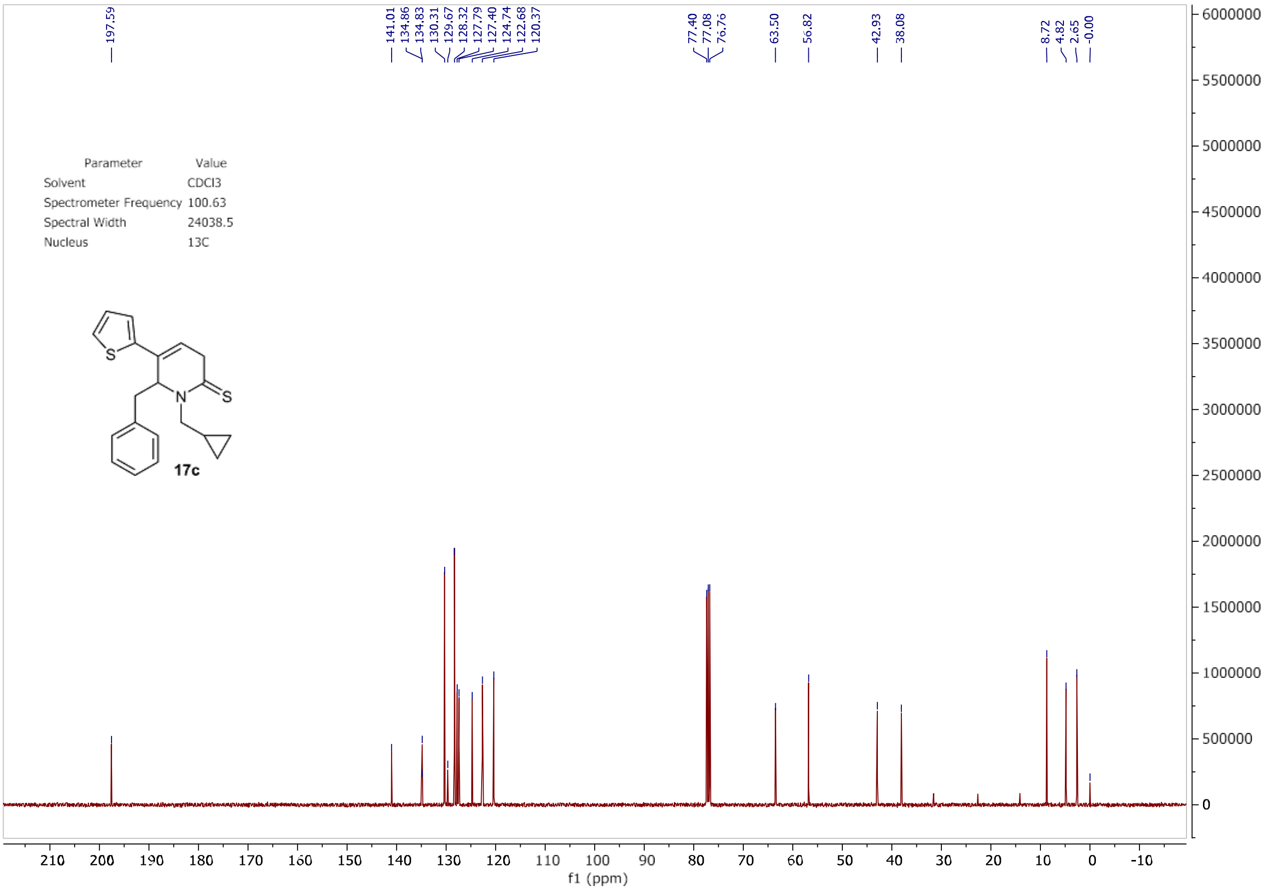

Figure S2.39. ^{1}H NMR (top) and ^{13}C NMR (bottom) spectra of compound **17c**

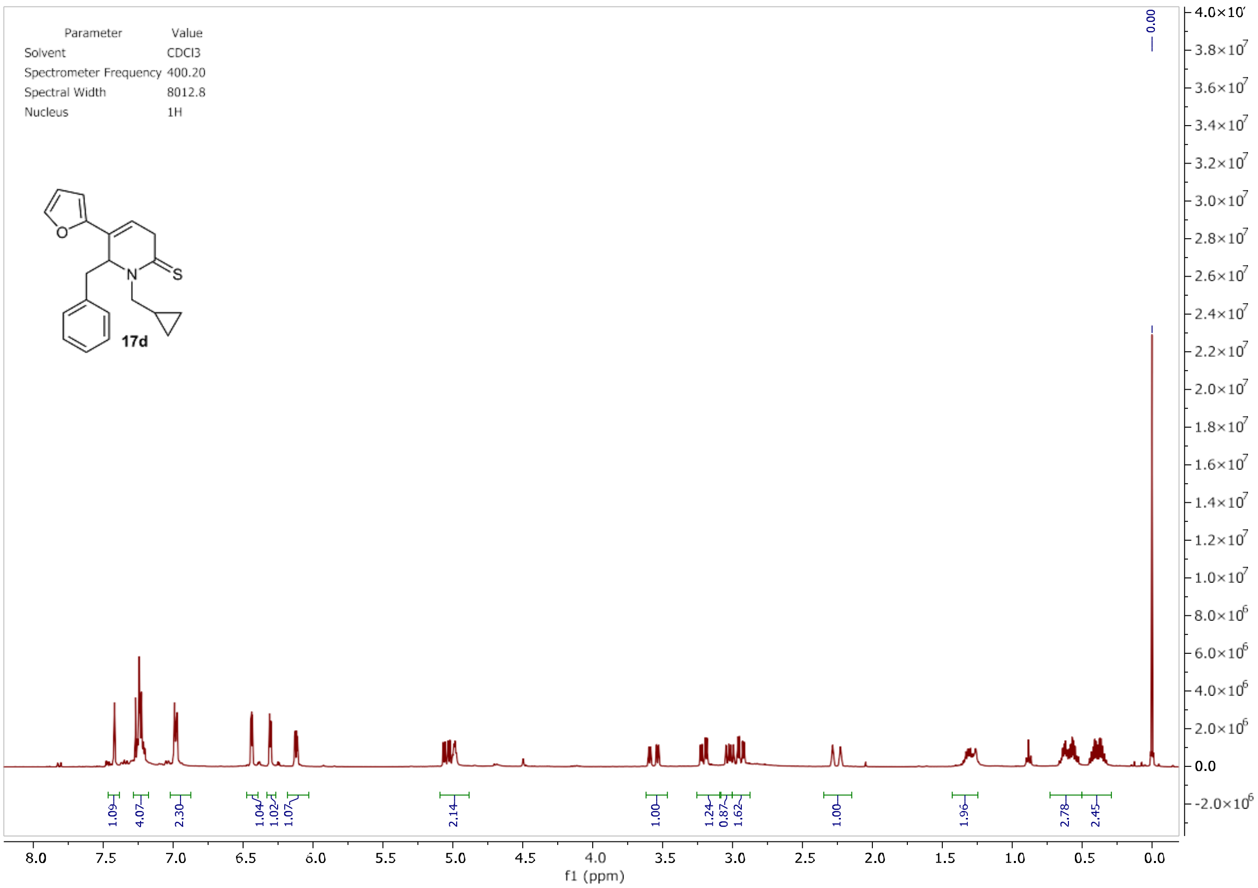

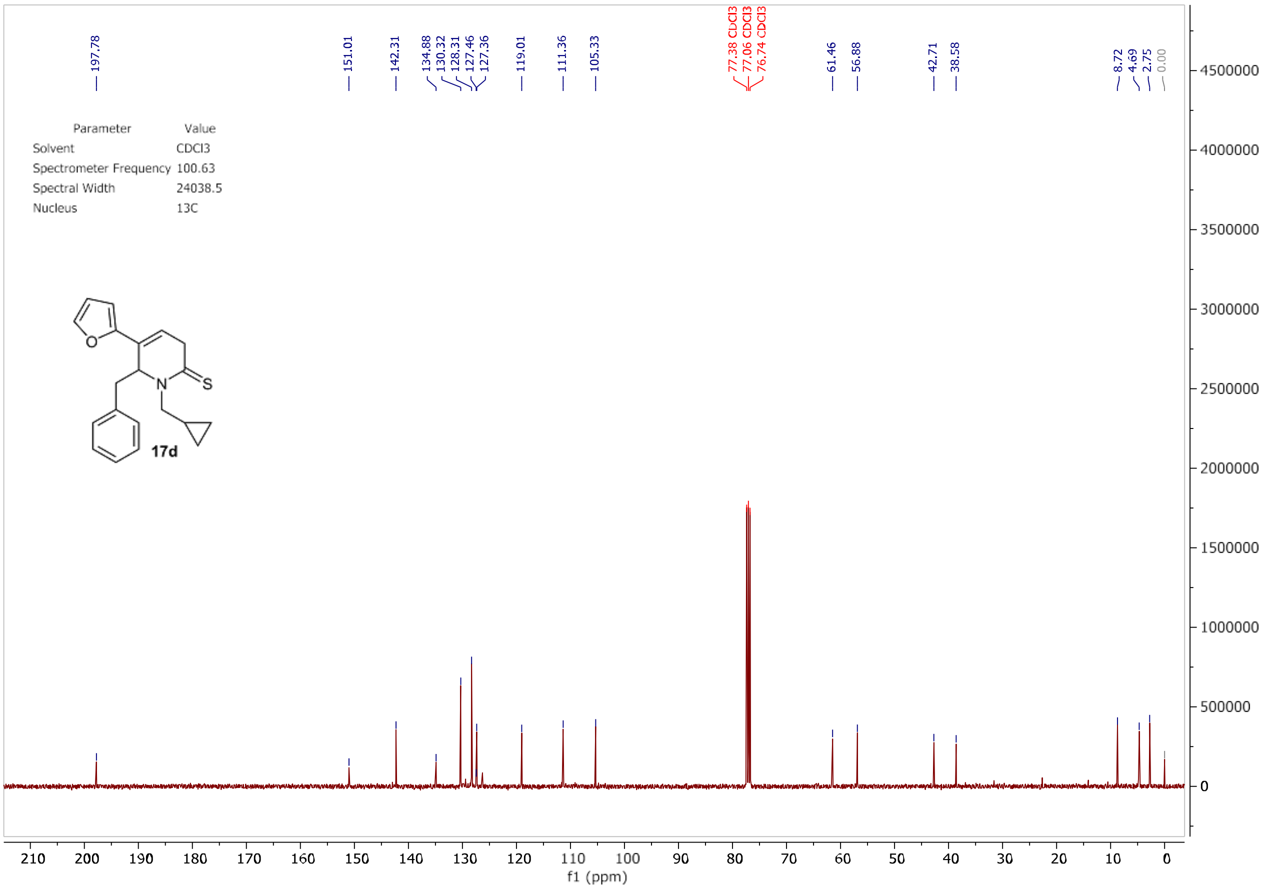

Figure S2.40. ^{1}H NMR (top) and ^{13}C NMR (bottom) spectra of compound **17d**

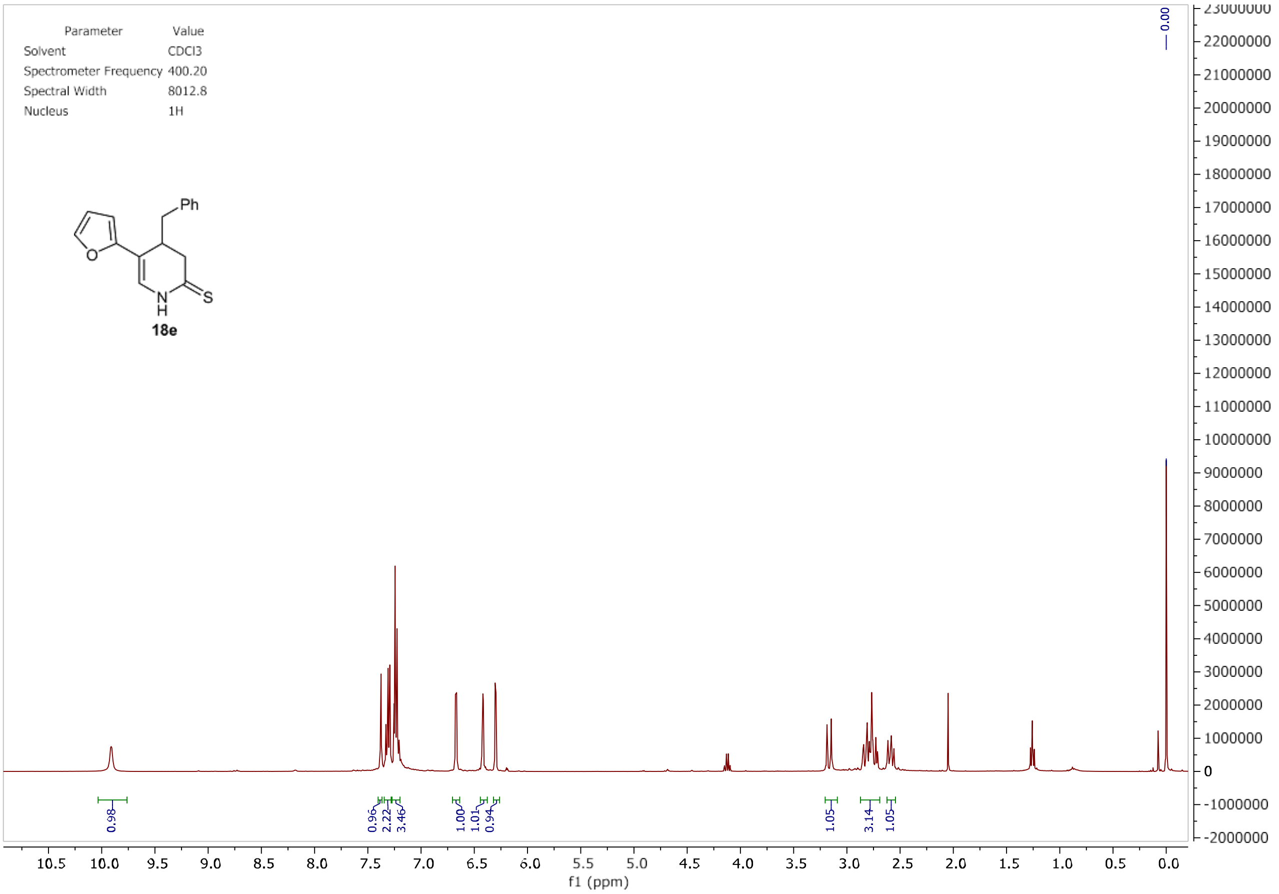

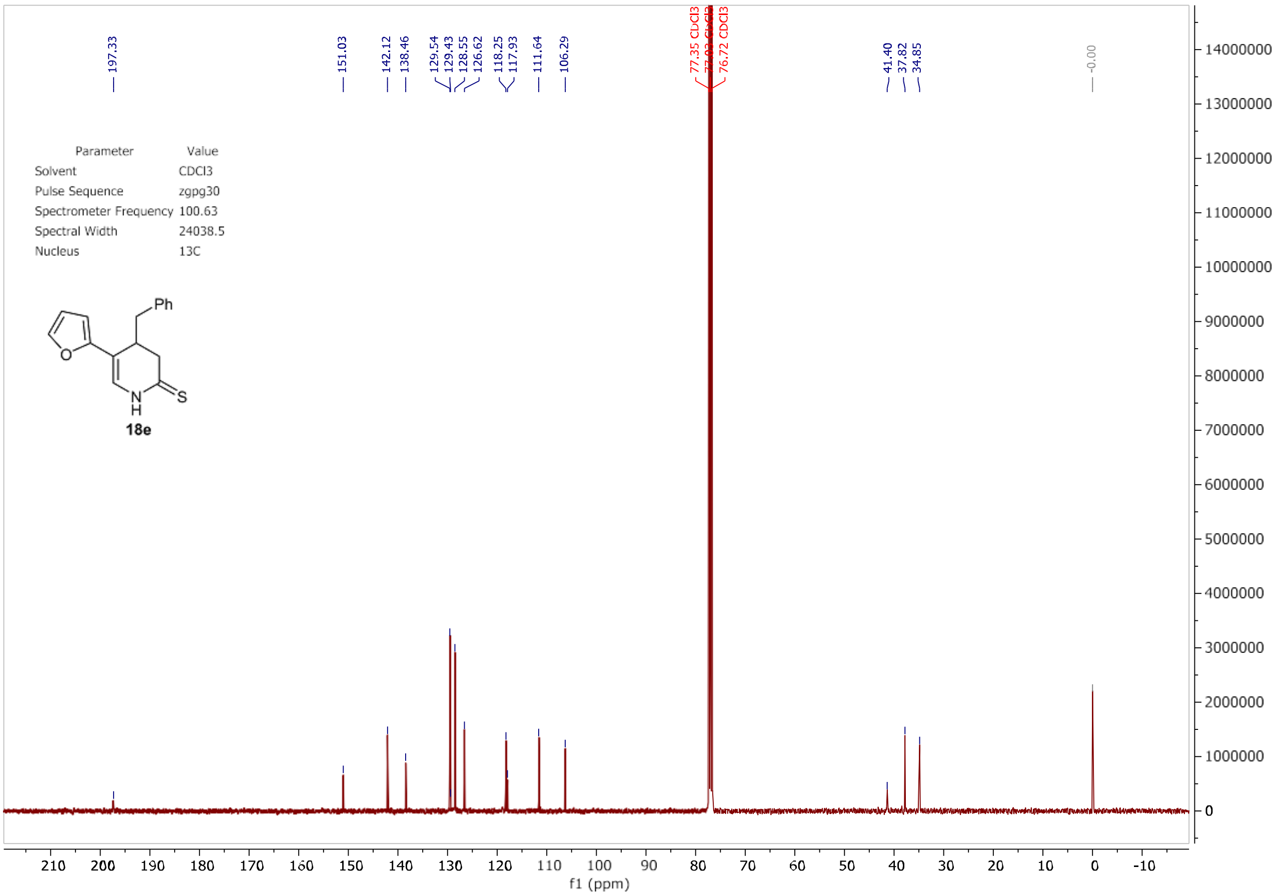

Figure S2.41. ^{1}H NMR (top) and ^{13}C NMR (bottom) spectra of compound **18e**

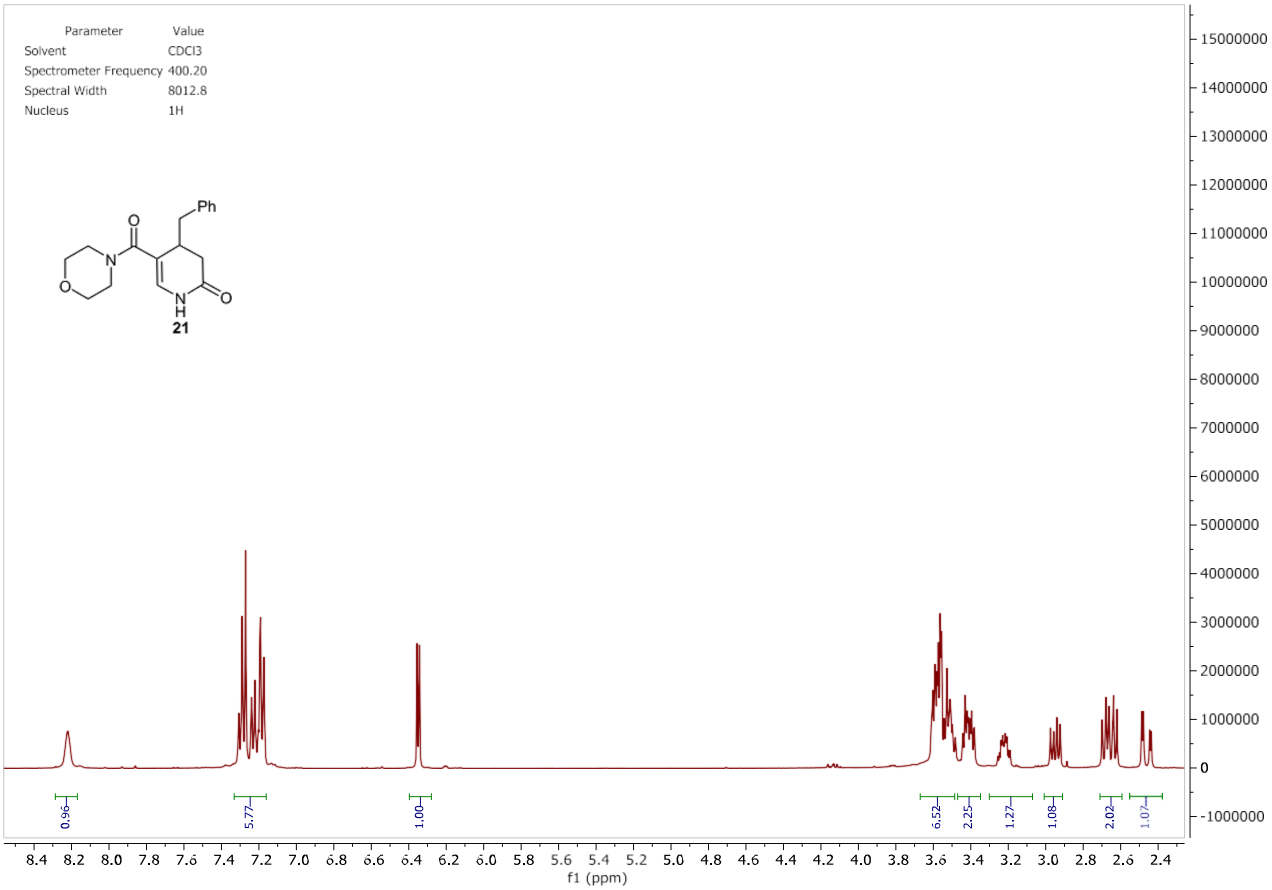

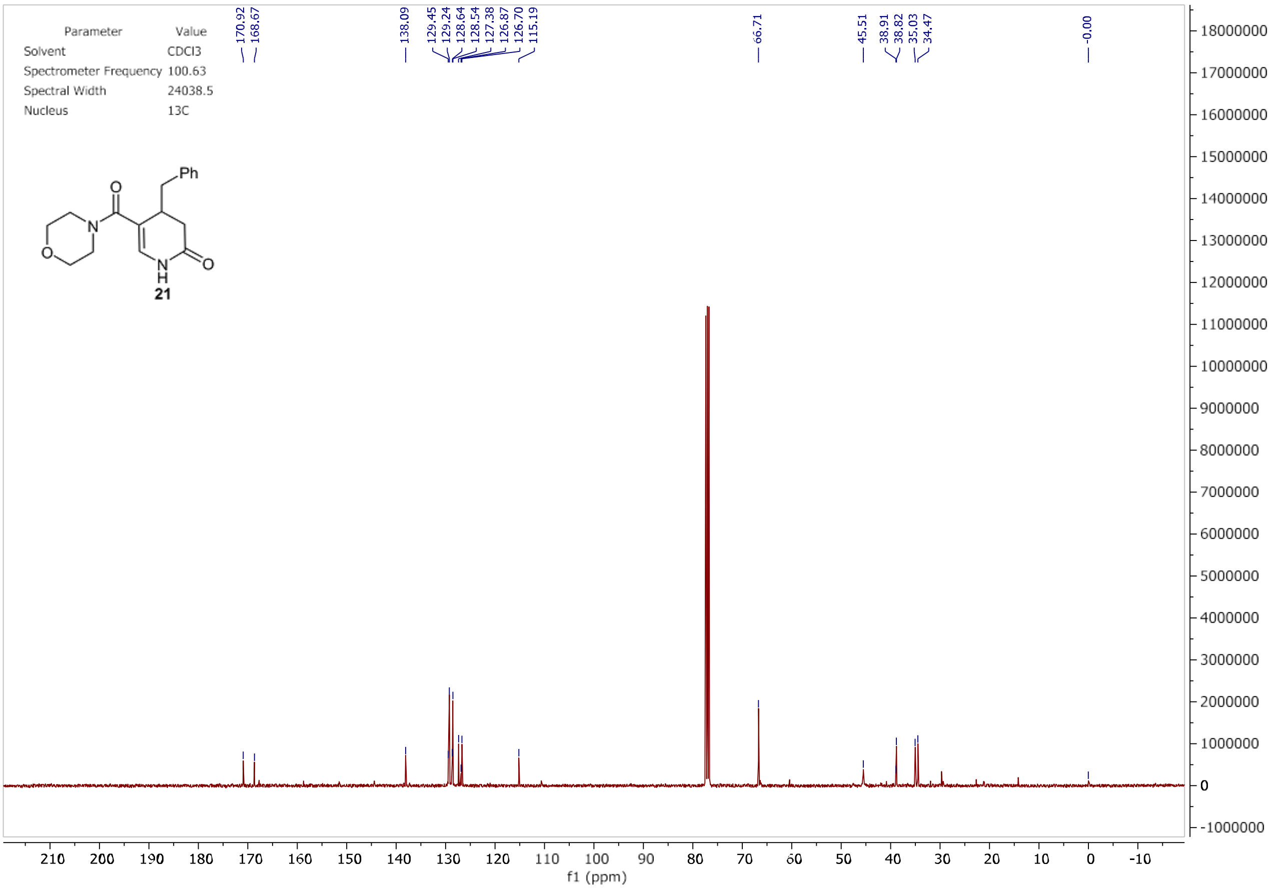

Figure S2.42. ^{1}H NMR (top) and ^{13}C NMR (bottom) spectra of compound **21**

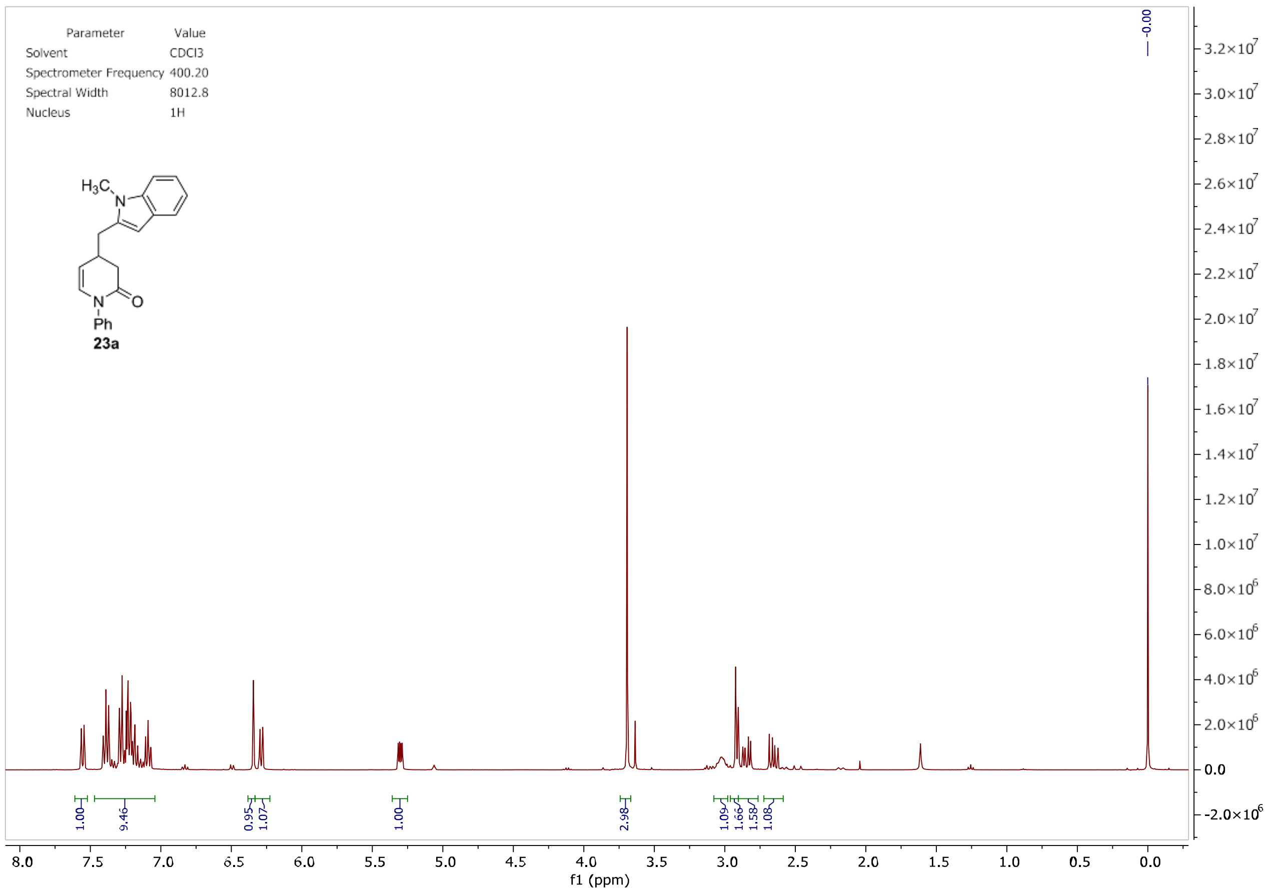

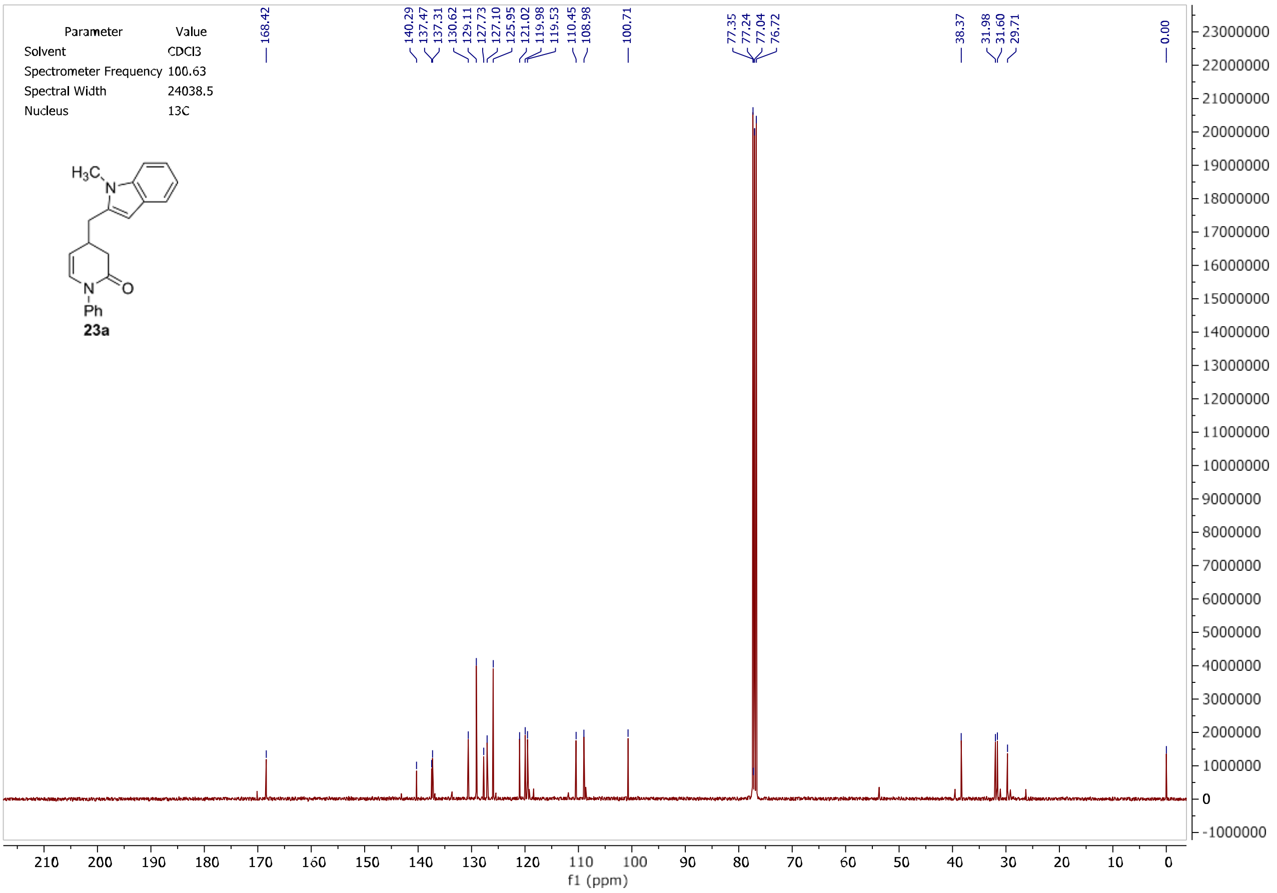

Figure S2.43. ^{1}H NMR (top) and ^{13}C NMR (bottom) spectra of compound **23a**

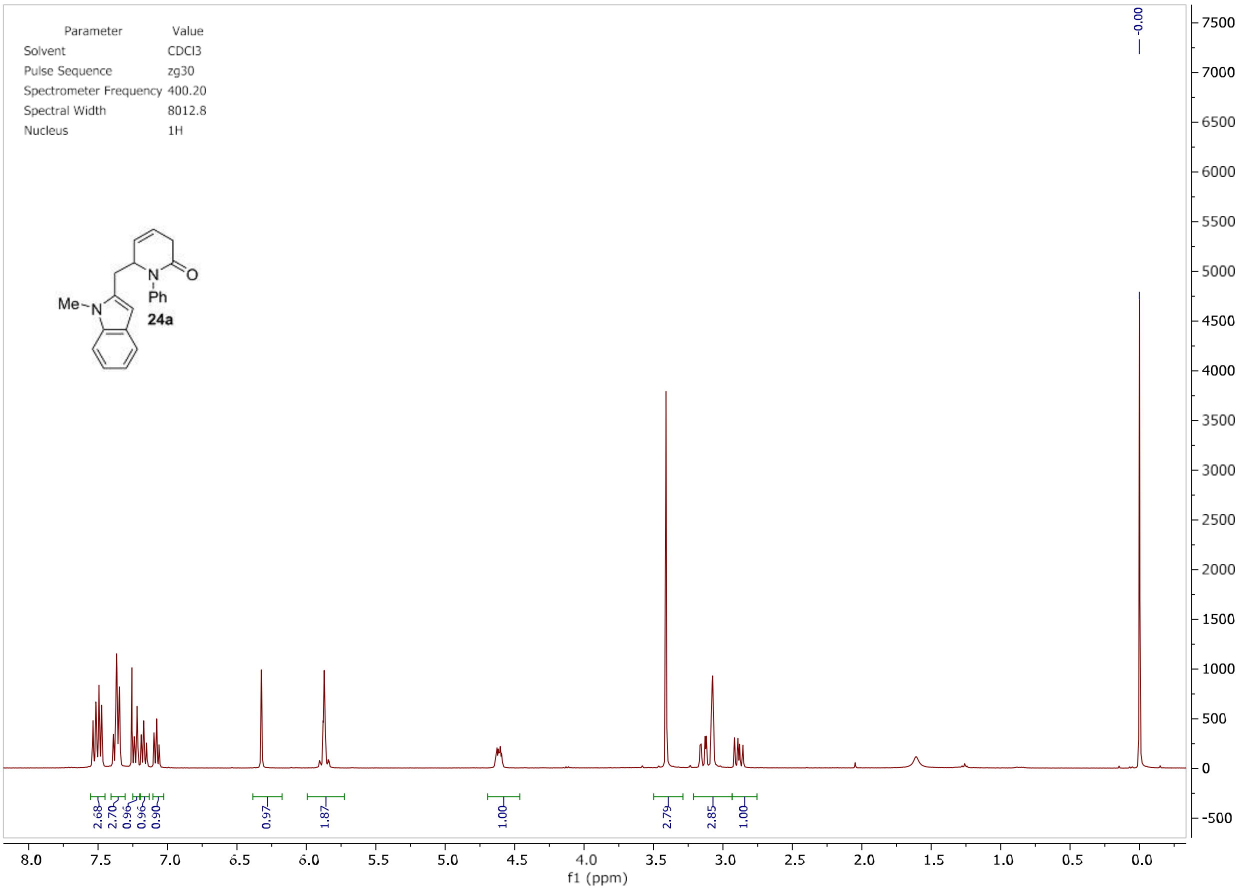

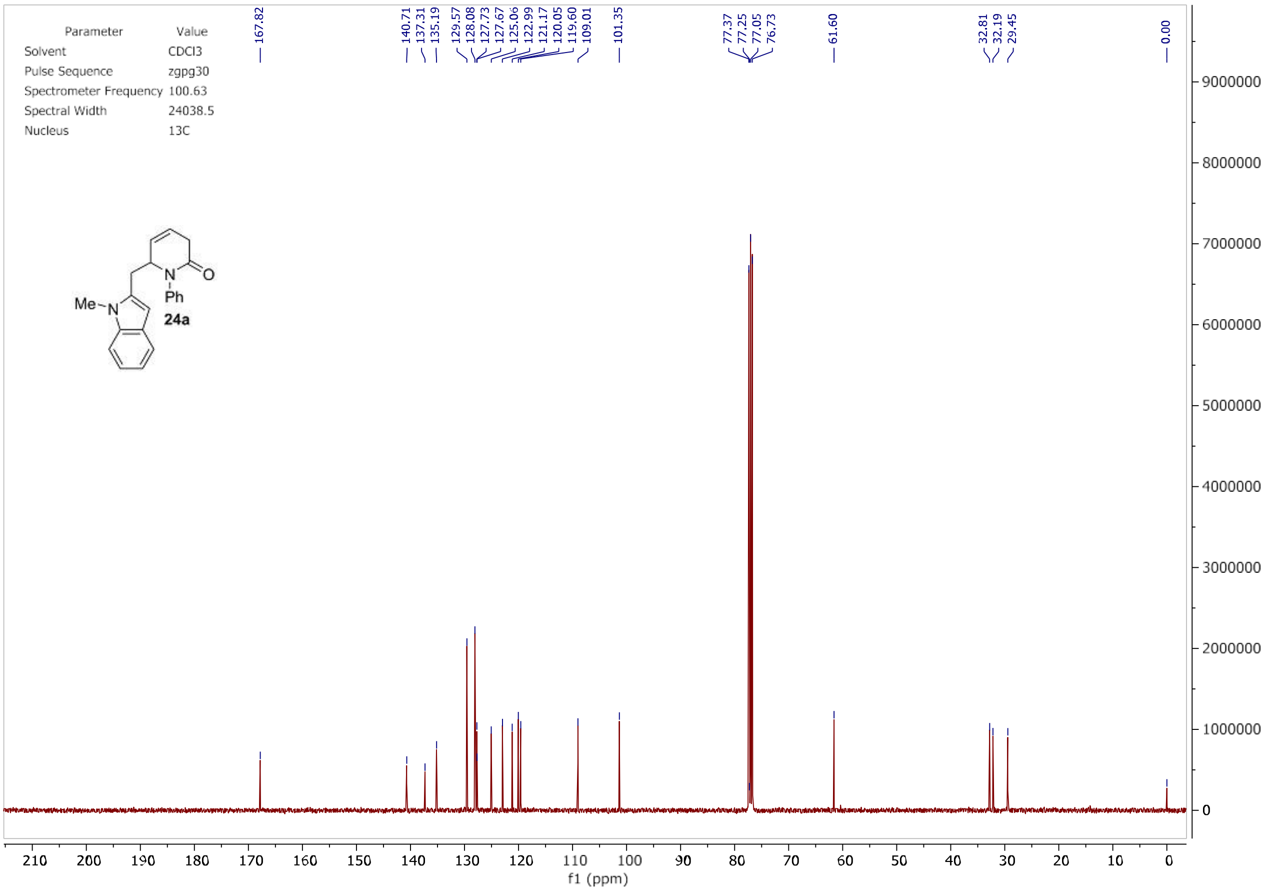

Figure S2.44. ^{1}H NMR (top) and ^{13}C NMR (bottom) spectra of compound **24a**

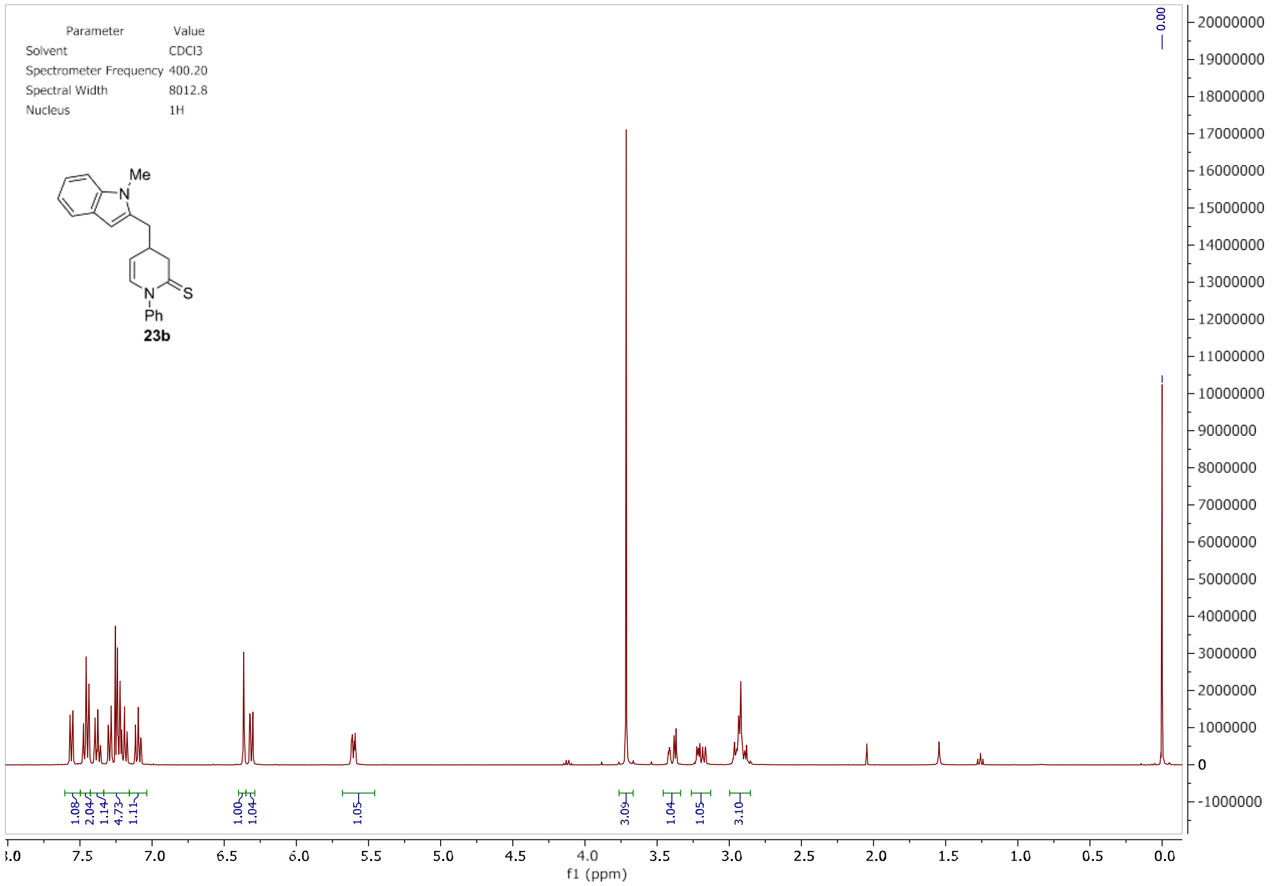

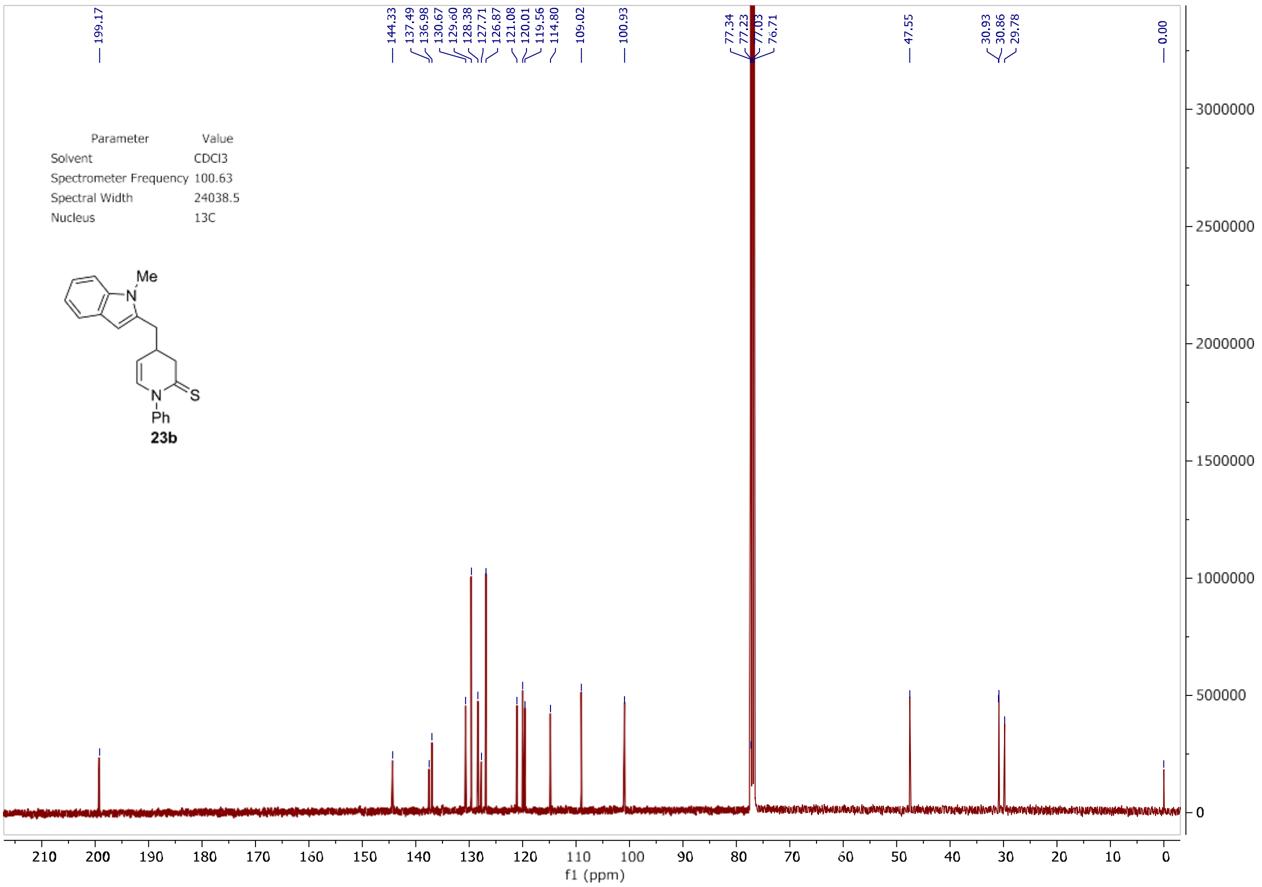

Figure S2.45. ^{1}H NMR (top) and ^{13}C NMR (bottom) spectra of compound **23b**

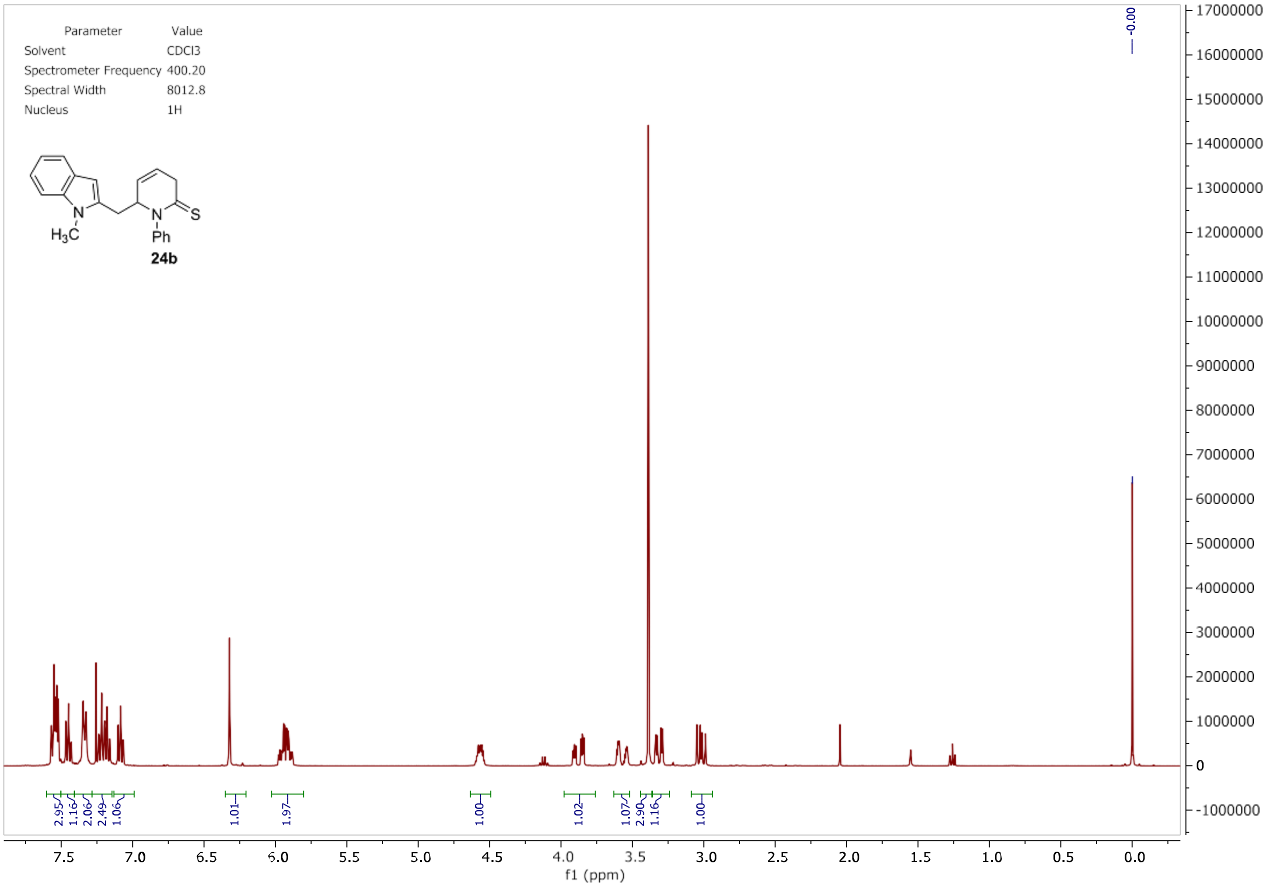

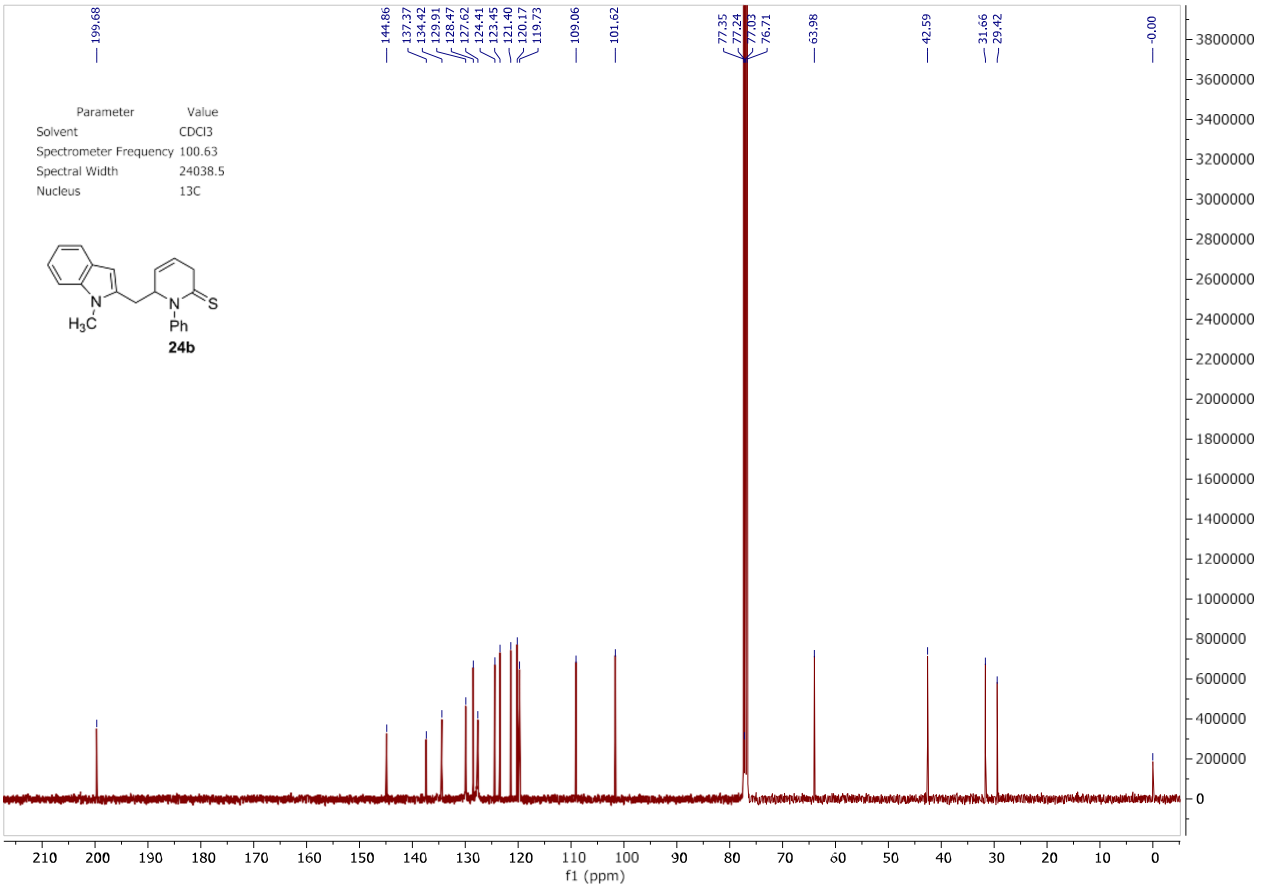

Figure S2.46. ^{1}H NMR (top) and ^{13}C NMR (bottom) spectra of compound **24b**

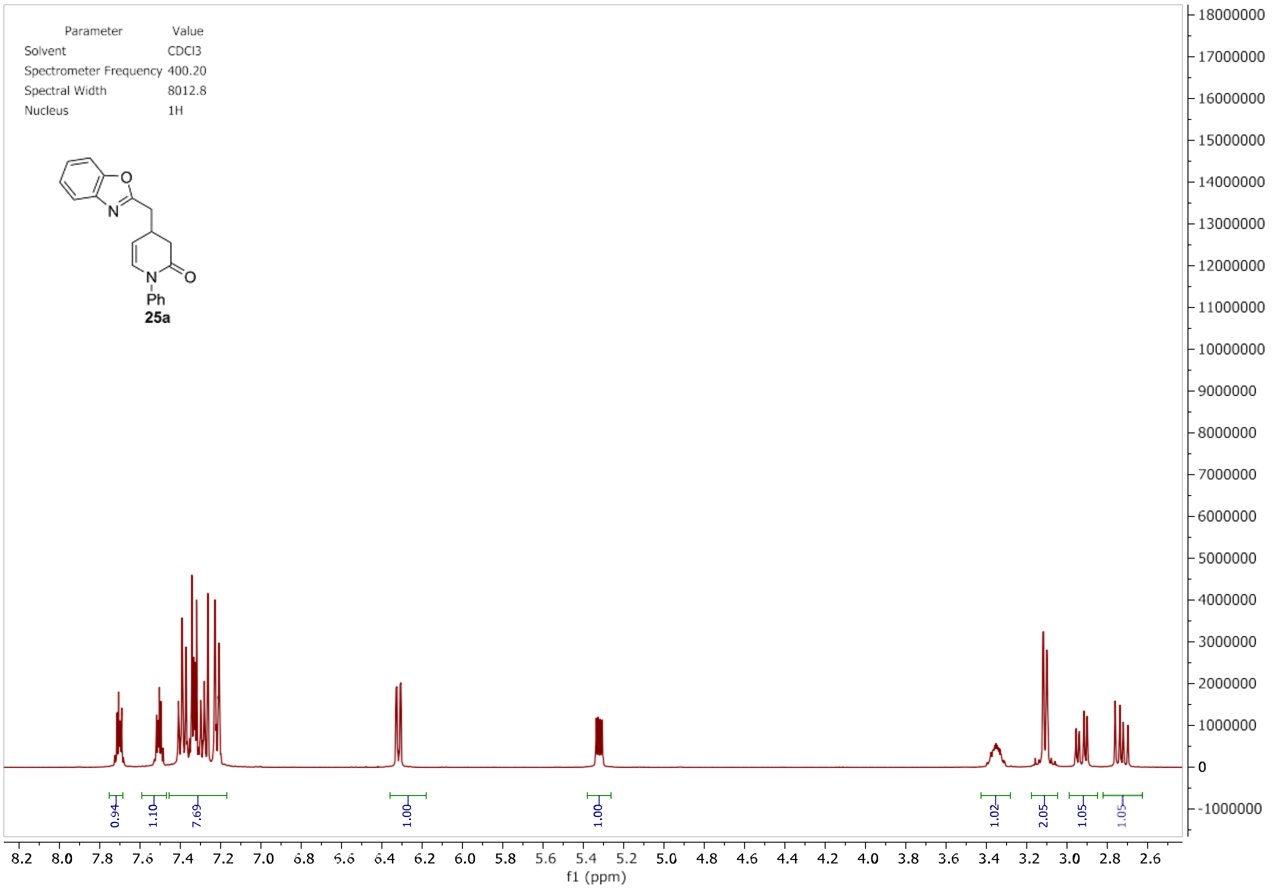

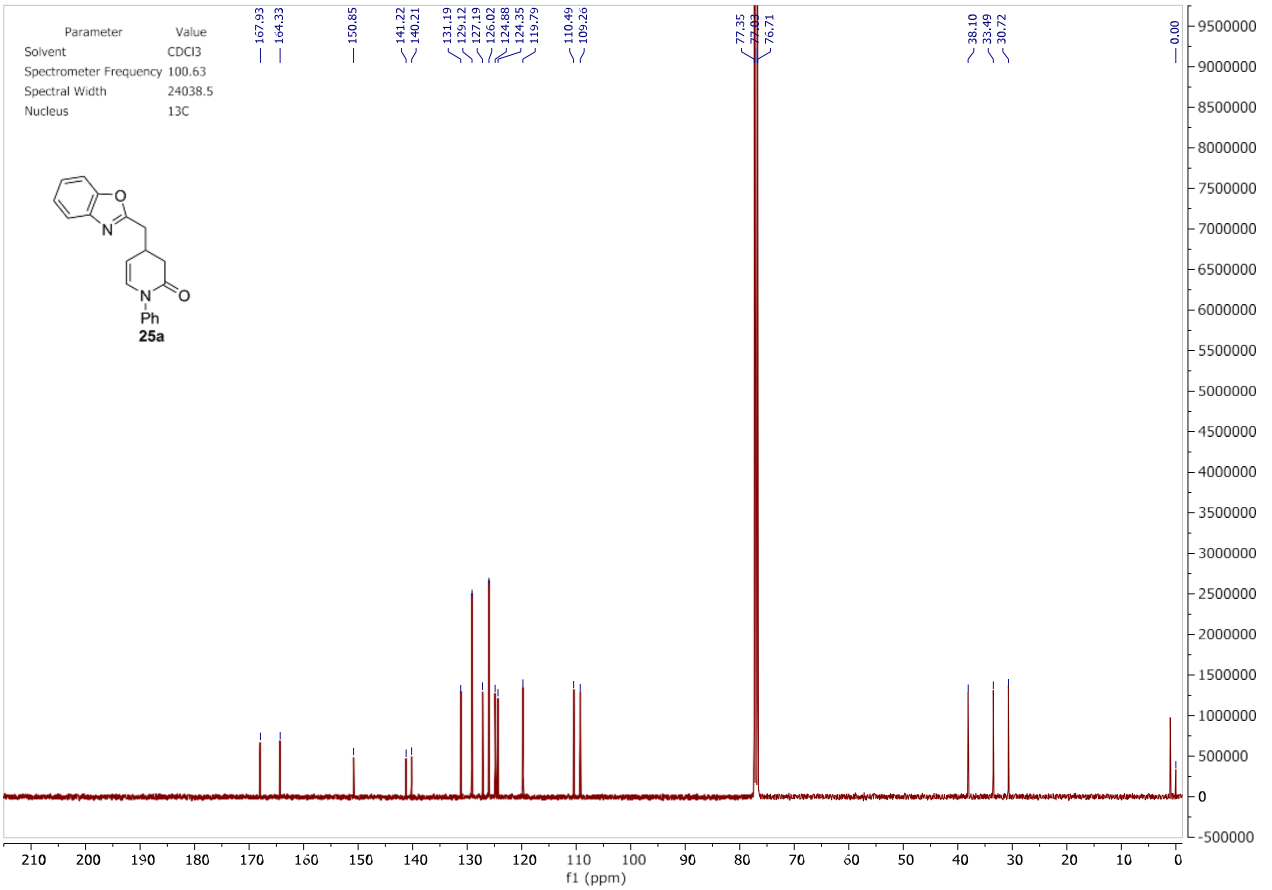

Figure S2.47. ^{1}H NMR (top) and ^{13}C NMR (bottom) spectra of compound **25a**

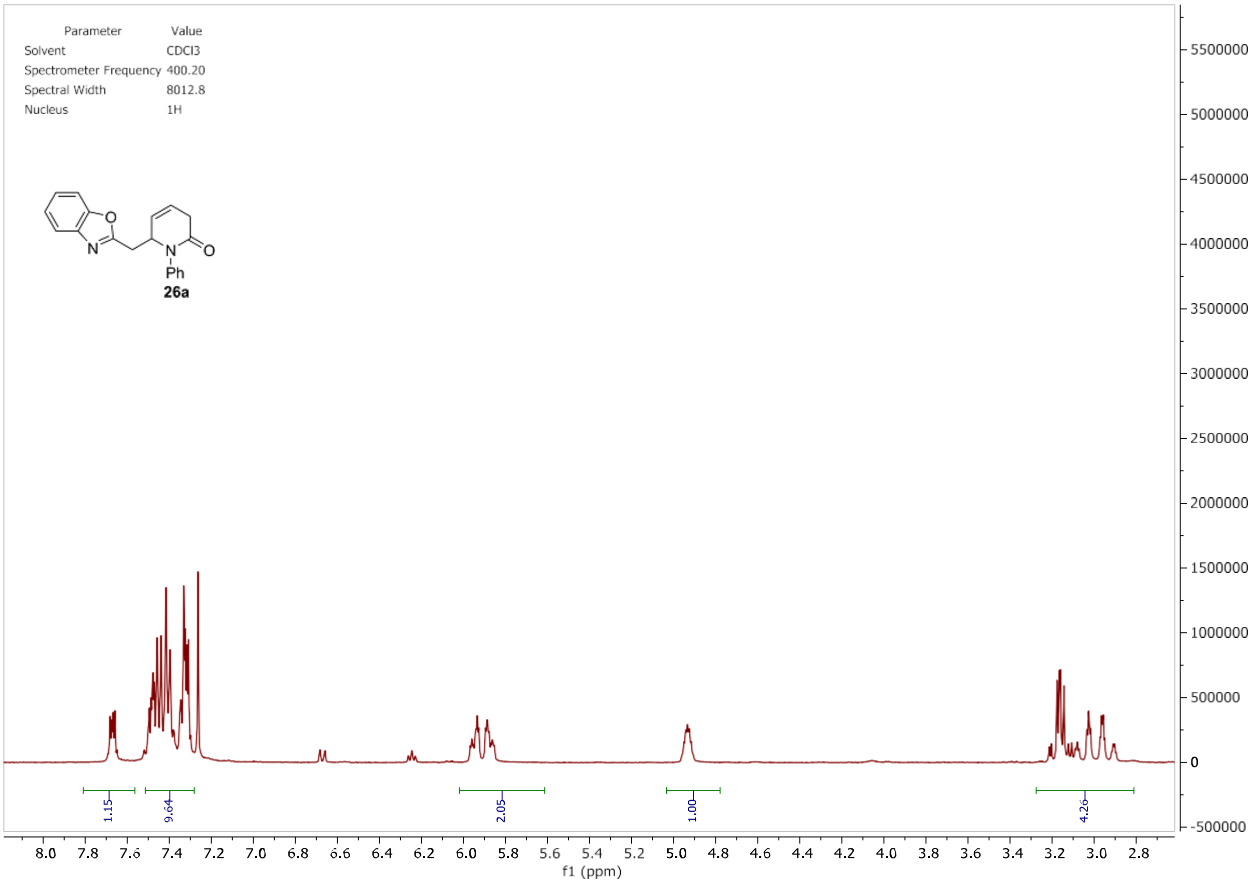

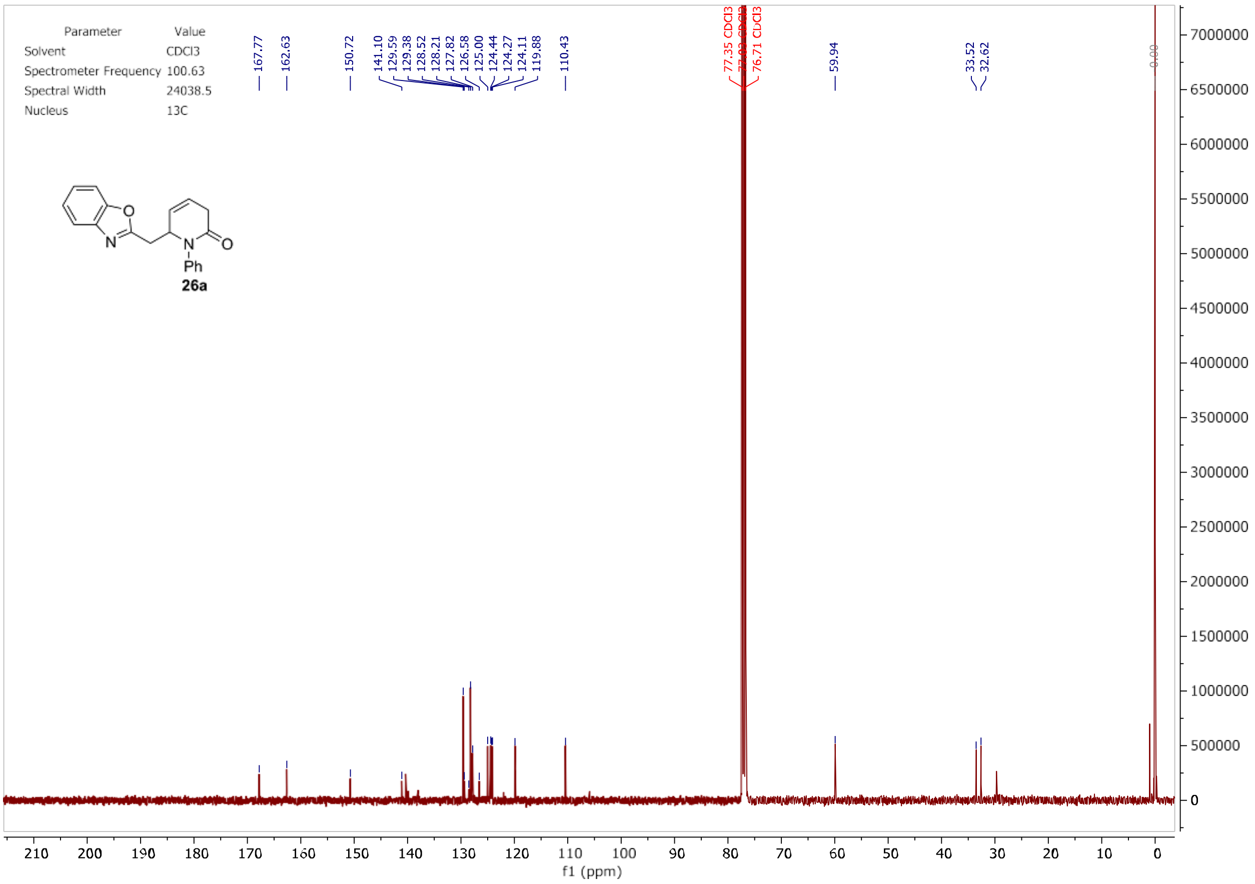

Figure S2.48. ^{1}H NMR (top) and ^{13}C NMR (bottom) spectra of compound **26a**

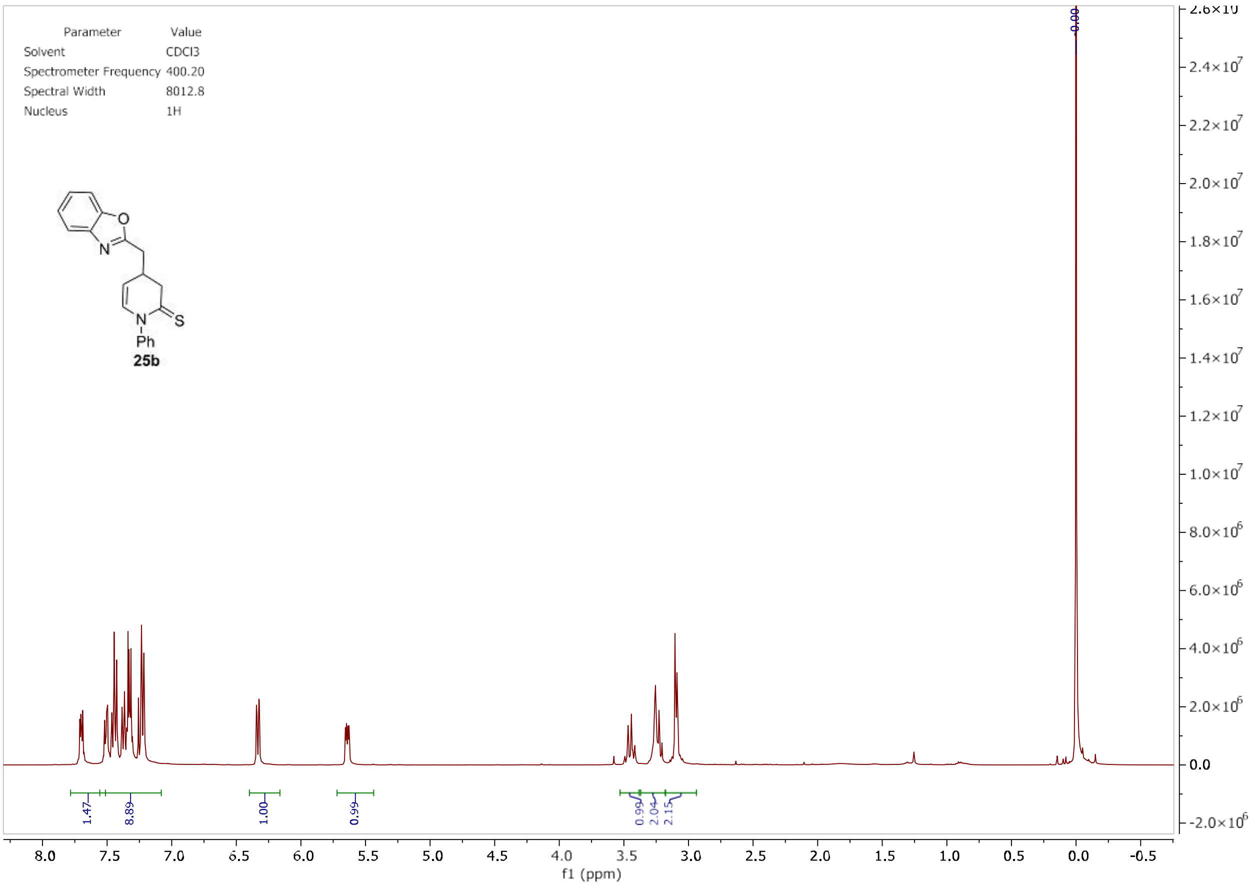

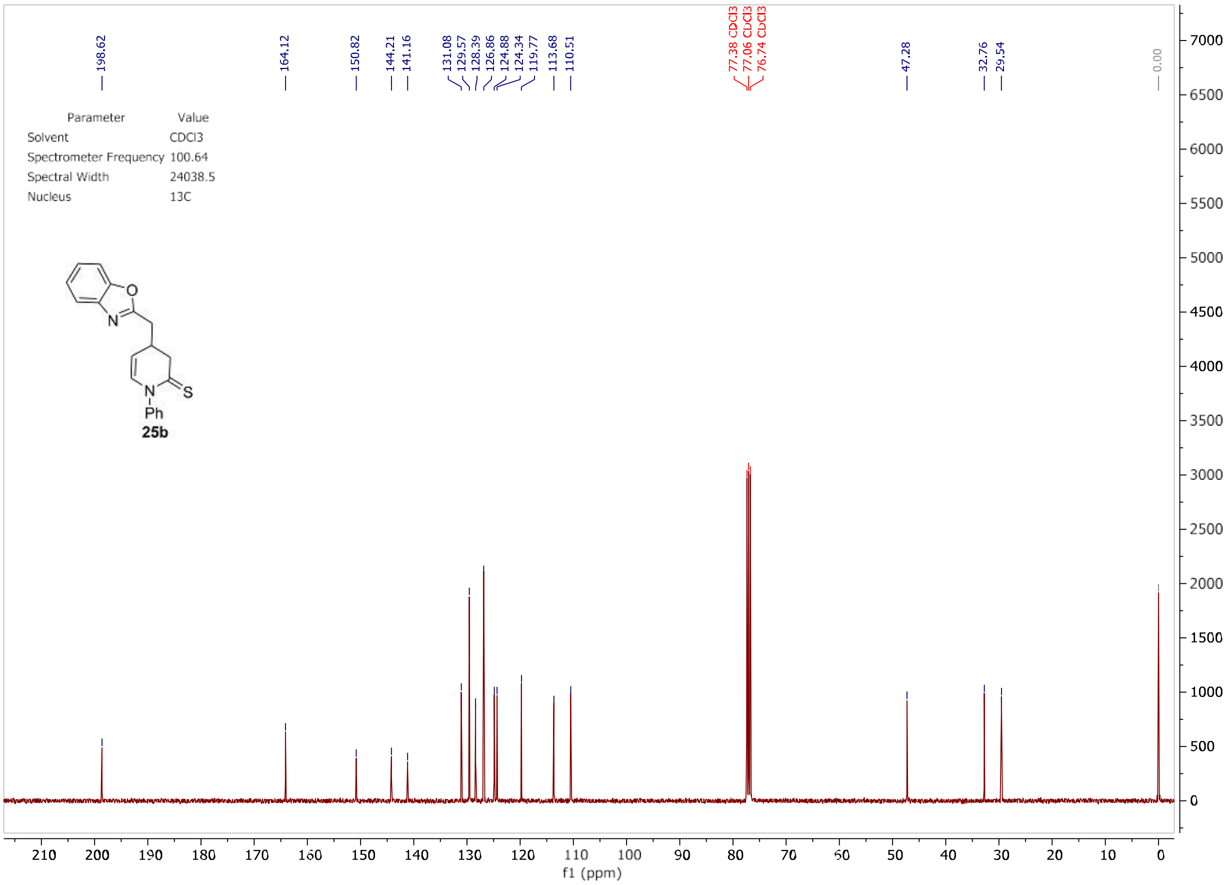

Figure S2.49. ^{1}H NMR (top) and ^{13}C NMR (bottom) spectra of compound **25b**

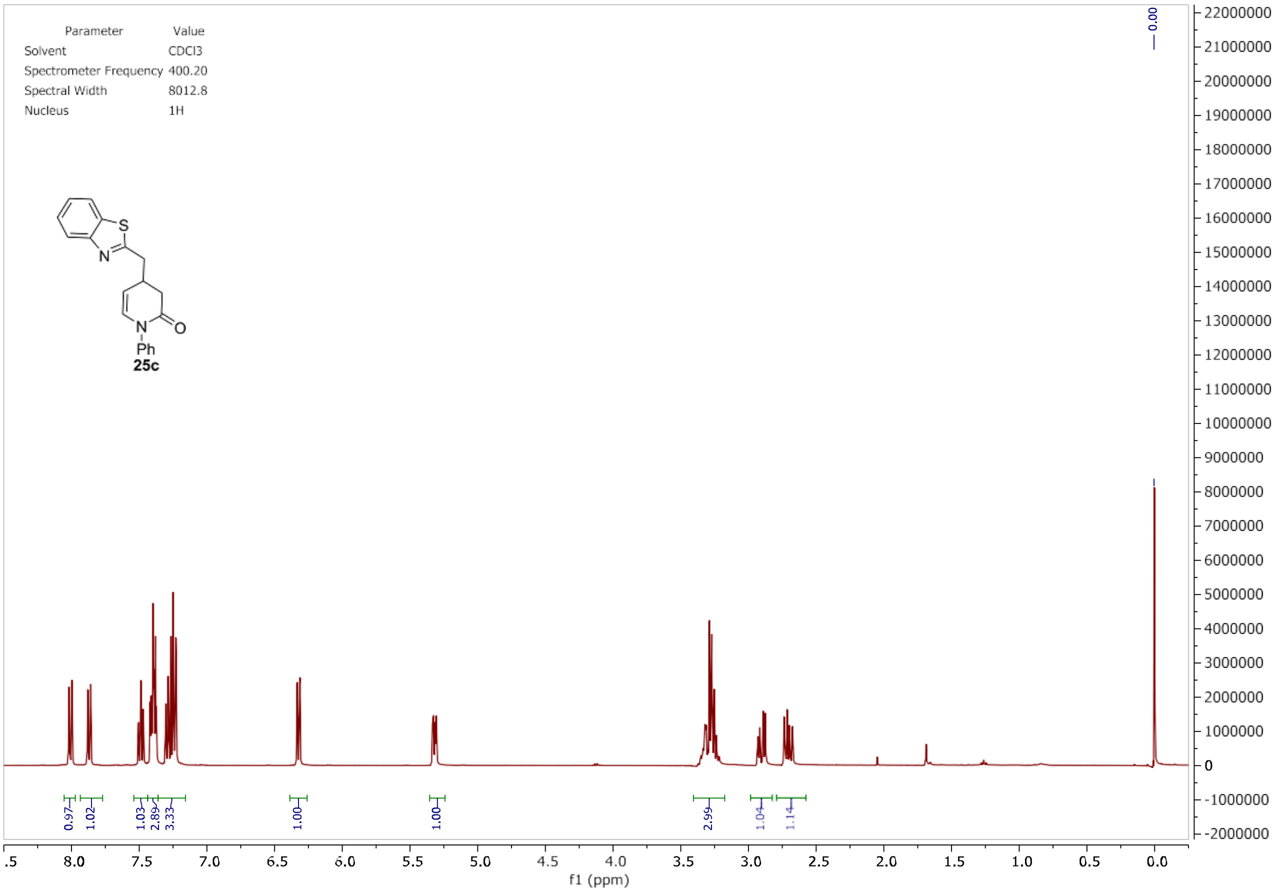

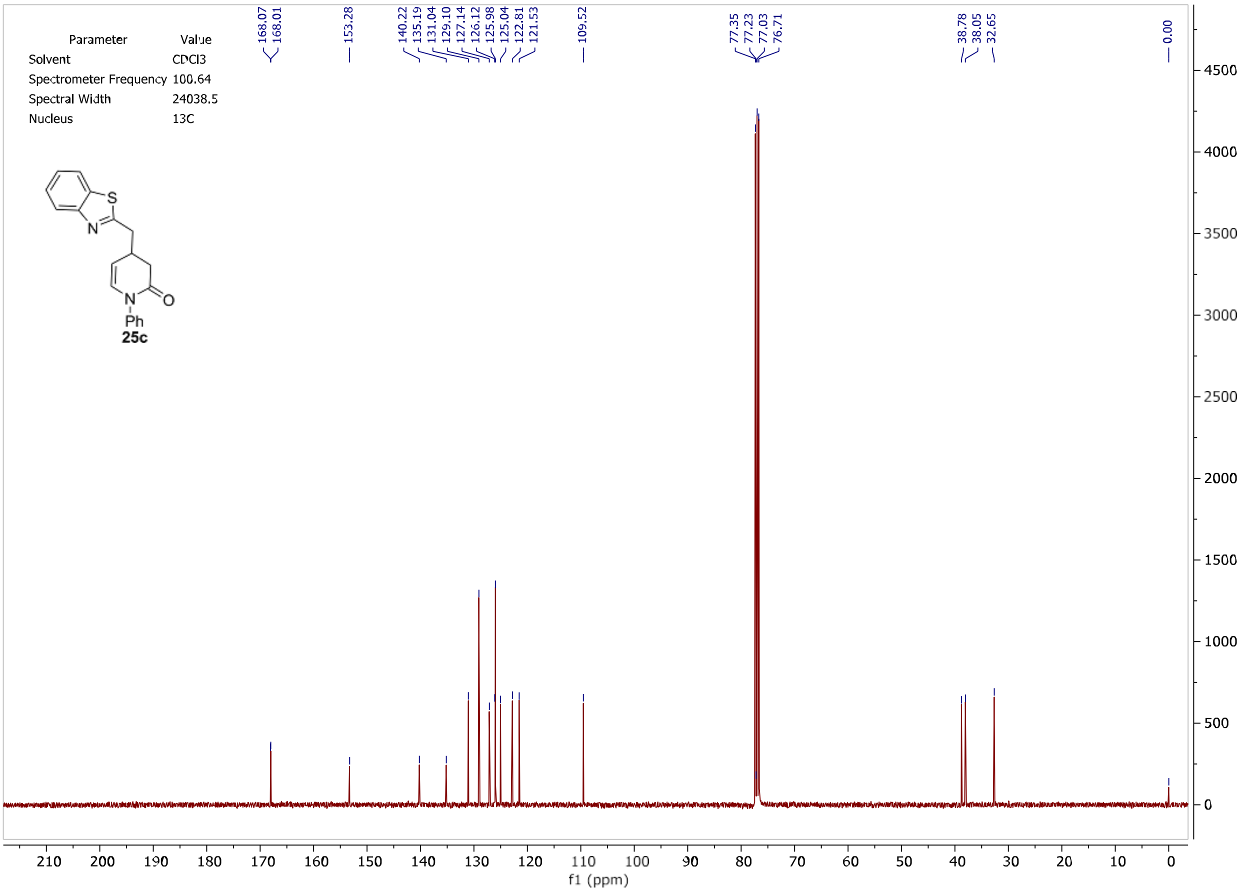

Figure S2.50. ^{1}H NMR (top) and ^{13}C NMR (bottom) spectra of compound **25c**

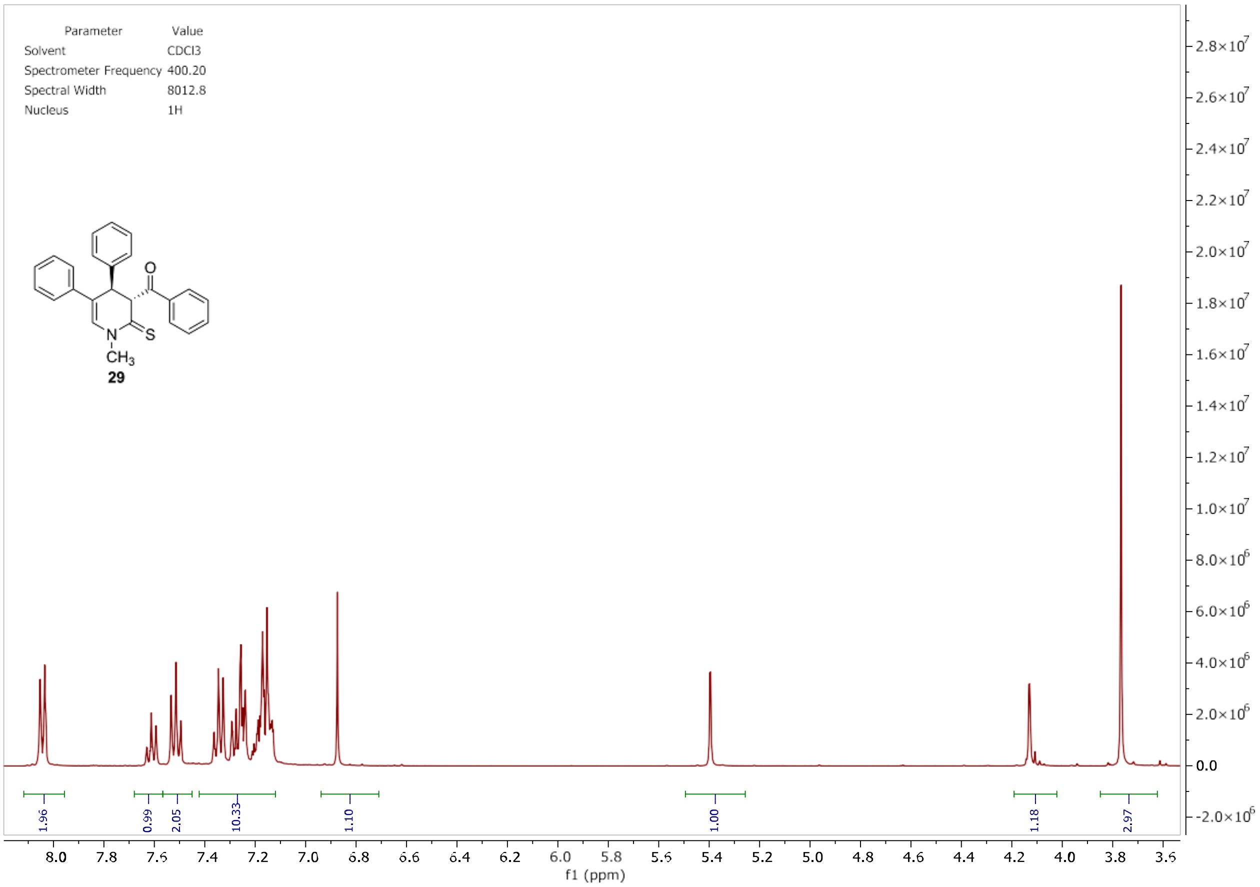

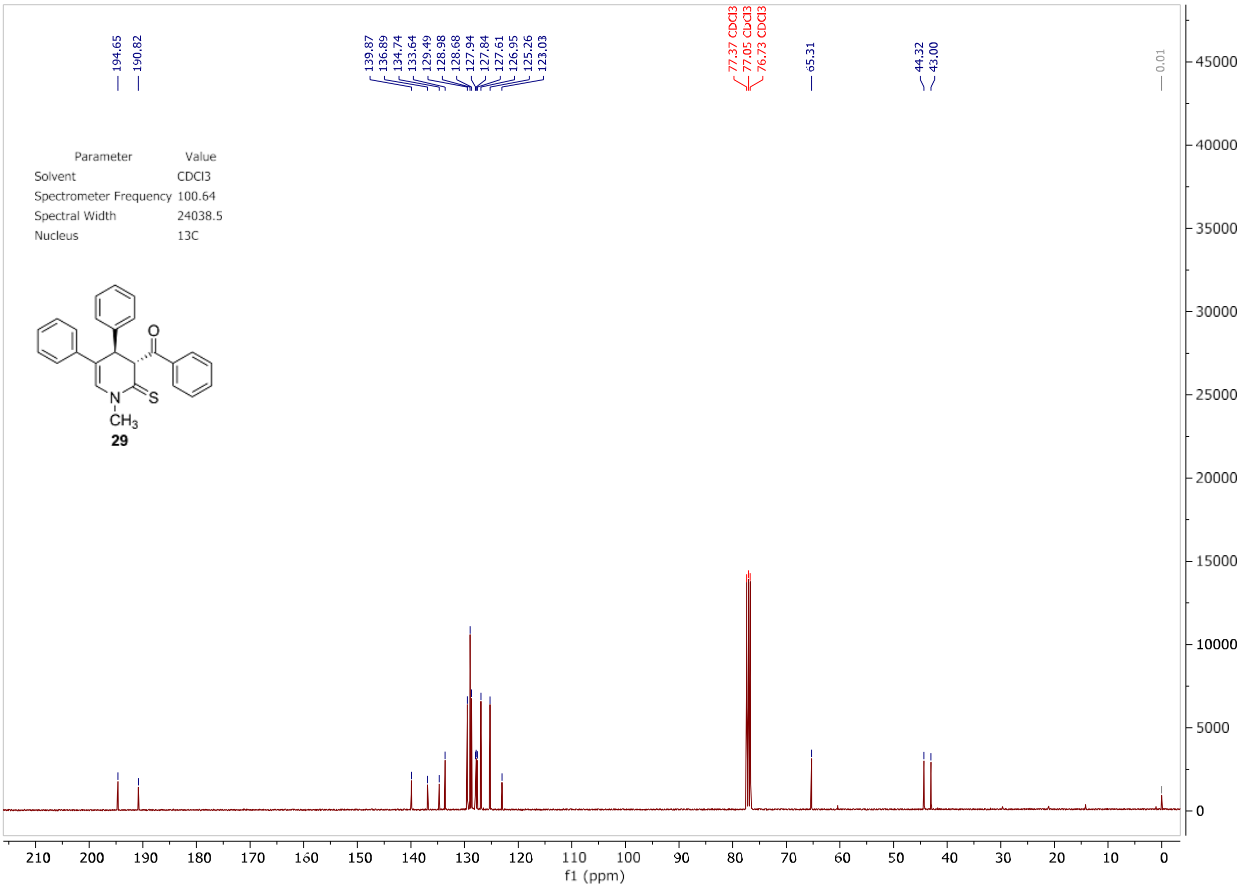

Figure S2.51. ^{1}H NMR (top) and ^{13}C NMR (bottom) spectra of compound **29**
